# Risk of Hearing Loss in Patients Treated with Exendin-4 Derivatives: A Network Meta-Analysis of Glucagon-like Peptide-1 Receptor Agonists and Sodium–Glucose Cotransporter 2 Inhibitors

**DOI:** 10.3390/ph18050735

**Published:** 2025-05-16

**Authors:** Jiann-Jy Chen, Chih-Wei Hsu, Chao-Ming Hung, Chih-Sung Liang, Kuan-Pin Su, Andre F. Carvalho, Brendon Stubbs, Yen-Wen Chen, Tien-Yu Chen, Wei-Te Lei, Bing-Yan Zeng, Ping-Tao Tseng

**Affiliations:** 1 Prospect Clinic for Otorhinolaryngology & Neurology, Kaohsiung 81166, Taiwan; jiannjy@yahoo.com.tw (J.-J.C.); kevinachen0527@gmail.com (Y.-W.C.); 2 Department of Otorhinolaryngology, E-Da Cancer Hospital, I-Shou University, Kaohsiung 82445, Taiwan; 3 Department of Psychiatry, Kaohsiung Chang Gung Memorial Hospital and Chang Gung University College of Medicine, Kaohsiung 833, Taiwan; harwicacademia@gmail.com; 4 Division of General Surgery, Department of Surgery, E-Da Cancer Hospital, I-Shou University, Kaohsiung 82445, Taiwan; ed100647@edah.org.tw; 5 School of Medicine, College of Medicine, I-Shou University, Kaohsiung 84001, Taiwan; 6 Department of Psychiatry, Beitou Branch, Tri-Service General Hospital, School of Medicine, National Defense Medical Center, Taipei 112003, Taiwan; lcsyfw@gmail.com; 7 Department of Psychiatry, National Defense Medical Center, Taipei 11490, Taiwan; 8 Department of Psychiatry & Mind-Body Interface Laboratory (MBI-Lab), China Medical University Hospital, Taichung 404328, Taiwan; cobolsu@gmail.com; 9 College of Medicine, China Medical University, Taichung 404328, Taiwan; 10 An-Nan Hospital, China Medical University, Tainan 709, Taiwan; 11 Innovation in Mental and Physical Health and Clinical Treatment (IMPACT) Strategic Research Centre, School of Medicine, Barwon Health, Deakin University, Geelong, VIC 3220, Australia; canaldasaudemental@gmail.com; 12 Department of Psychological Medicine, Institute of Psychiatry, Psychology and Neuroscience, King’s College London, London WC2R 2LS, UK; brendon.stubbs@kcl.ac.uk; 13 Department of Sport, University of Vienna, 1010 Vienna, Austria; 14 Department of Psychiatry, Tri-Service General Hospital, School of Medicine, National Defense Medical Center, Taipei 114, Taiwan; verducciwol@gmail.com; 15 Institute of Brain Science, National Yang Ming Chiao Tung University, Taipei 112, Taiwan; 16 Section of Immunology, Rheumatology, and Allergy Department of Pediatrics, Municipal MacKay Children’s Hospital, Hsinchu 300046, Taiwan; lazyleisure@gmail.com; 17 Department of Medicine, MacKay Medical College, New Taipei 25245, Taiwan; 18 Institute of Biomedical Sciences, National Sun Yat-sen University, Kaohsiung 804201, Taiwan; 19 Department of Internal Medicine, E-Da Dachang Hospital, I-Shou University, Kaohsiung 807, Taiwan; 20 Institute of Precision Medicine, National Sun Yat-sen University, Kaohsiung 804201, Taiwan

**Keywords:** network meta-analysis, GLP-1 receptor agonist, SGLT2 inhibitor, exendin-4, hearing loss, lixisenatide, efpeglenatide

## Abstract

**Background/Objectives**: Emerging evidence suggests an association between glucagon-like peptide-1 (GLP-1) receptor agonists and sodium–glucose co-transporter 2 (SGLT2) inhibitors with altered risk of damage in the inner ear system. However, limited research exists on the relationship between these medications and subsequent irreversible hearing loss. We conducted this network meta-analysis (NMA) to evaluate the comparative risk of hearing loss associated with such medications. **Methods**: In this NMA, we used a confirmatory approach to specifically focus on particular adverse effects of interest (i.e., incidence of hearing loss here) based on the Cochrane recommendation. A Bayesian-based NMA of randomized controlled trials (RCTs) of GLP-1 receptor agonists or SGLT2 inhibitors was conducted. The primary outcome was the hearing loss events. **Results**: Our NMA of 29 RCTs with 145,895 participants found that only two exendin-4 derivatives—lixisenatide and high-dose efpeglenatide (i.e., 6 mg/week)—showed increased hearing loss events compared to controls. No other GLP-1 receptor agonists or SGLT2 inhibitors demonstrated significantly elevated hearing loss risk. Lixisenatide ranked highest in risk among all investigated regimens. **Conclusions**: This comprehensive NMA identifies a significant association between exendin-4 derivatives (lixisenatide and efpeglenatide) and potential ototoxicity. Clinicians should carefully consider this potential ototoxicity when prescribing exendin-4 derivatives, particularly in patients with pre-existing hearing loss risk factors.

## 1. Introduction

Glucagon-like peptide-1 (GLP-1) receptor agonists and sodium–glucose co-transporter 2 (SGLT2) inhibitors have emerged as novel glucose-lowering agents, featuring mechanisms of action distinct from those of conventional treatments [1]. However, along with their widespread prescription, there have been more and more reports regarding irreversible end-organ damage [2]. For example, Lee et al. found a significantly increased risk of retinal vein occlusion and SGLT2 inhibitor use [3]. Similarly, the prescription of exendin-4, one type of the GLP-1 receptor agonist, would increase the risk of formation of a micro-platelet-erythrocyte clot via suppression of matrix metalloproteinase-9 activity [4,5].

Although not clearly clarified, one of the potential etiologies of hearing loss, either sudden onset or chronic course, was associated with the disturbance of microcirculation in the inner ear system. For example, in addition to the direct injury related to noise exposure, loud sounds could affect red blood cell velocity and blood vessel diameter in the cochlea and lead to disturbances in cochlear micro-circulation [6]. Furthermore, there have been several reports addressing the formation of micro-platelet-erythrocyte clots and consequent occlusion of micro-circulation in the inner ear in subjects with various autoimmune diseases, which would finally lead to irreversible sensorineural hearing loss [7,8,9]. Therefore, theoretically, the prescription of GLP-1 receptor agonists and SGLT2 inhibitors might be associated with risk of irreversible hearing loss via disturbance of micro-circulation in inner ear.

However, there are currently few reports addressing the relationship between hearing loss and GLP-1 receptor agonists/SGLT2 inhibitor use. Although there was a trend of an association between them in large-scale randomized controlled trials (RCTs) [10,11], this issue has still not caught clinicians’ attention. A well-designed network meta-analysis (NMA) of GLP-1 receptor agonists and SGLT2 inhibitors, which allows for direct comparisons among different medications, could enhance the ability to make multiple treatment efficacy comparisons and assess the potential superiority of specific interventions at various dosages [12]. This approach offers a more detailed and evidence-based framework for guiding future clinical practices. To the best of our knowledge, no NMA has yet assessed the potential risk of hearing loss related to different GLP-1 receptor agonists and SGLT2 inhibitors used in various dosages. Therefore, following the rationale in our previous NMA addressing the risk of neurodegenerative diseases related to GLP-1 receptor agonists and SGLT2 inhibitor use [13], this NMA aims to provide evidence about the comparative risk of hearing loss by GLP-1 receptor agonists and SGLT2 inhibitor use.

## 2. Methods

In this study, we used a confirmatory approach to specifically focus on particular adverse effects of interest (i.e., incidence of hearing loss here) based on the Cochrane recommendation [14] in order to repurpose drugs for new roles [15,16]. This study followed the rationales of our recent article, which addressed the risk of neurodegenerative diseases related to GLP-1 receptor agonists and SGLT2 inhibitor use [13]. This NMA adhered to the Preferred Reporting Items for Systematic Reviews and Meta-Analyses (PRISMA) guidelines, including the extension for network meta-analyses (PRISMA NMA) (Table S1) [17]. The study protocol was registered in PROSPERO under registration number CRD42025645560. The study protocol was approved by the Institutional Review Board of the Tri-Service General Hospital, National Defense Medical Center (TSGHIRB E202516007).

### 2.1. Database Searches and Study Identification

We conducted comprehensive searches across multiple databases, including PubMed, Embase, ClinicalKey, Cochrane CENTRAL, ProQuest, ScienceDirect, Web of Science, and ClinicalTrials.gov (Table S2), covering publications up to 1 February 2025. Regarding the sources of unpublished trials, we actually used several databases, including ScienceDirect (in which several important poster abstract will be posted here), ProQuest (in which some thesis would be posted here), and ClinicalTrials.gov (in which some gray literature would be uploaded here). Two independent researchers (PT Tseng and BY Zeng) carried out these searches, screened titles and abstracts, and resolved disagreements through discussion. We also manually reviewed reference lists from relevant review articles and meta-analyses for additional studies [18,19]. No language restrictions were applied.

### 2.2. Inclusion and Exclusion Criteria

This NMA was guided by the PICOS framework (Population, Intervention, Comparison, Outcome, Study Design): Population: Individuals without documented hearing loss at baseline; Intervention: Use of GLP-1 receptor agonists or SGLT2 inhibitors at any dose; Comparison: Placebo, standard care, or active comparator; Outcome: Incidence of hearing loss, either sensorineural hearing loss or conductive hearing loss; Study design: RCTs.

To minimize selective reporting bias, only RCTs with systematic adverse event assessments or targeted outcome evaluations were included [20]. Further, RCTs exclusively enrolling participants with documented hearing loss at baseline were excluded to maintain focus on causative or prophylactic evaluation. Therefore, to reduce the potential heterogeneity and bias, the inclusion criteria were strictly set as: (1) RCTs with participants free from documented hearing loss at baseline; (2) RCTs evaluating GLP-1 receptor agonists or SGLT2 inhibitors; (3) Studies involving human subjects; and (4) RCTs with systematic adverse event monitoring or direct evaluation of target outcomes.

Exclusion criteria included the following: (1) non-RCT studies; (2) RCTs specifically enrolling participants with documented hearing loss at baseline; (3) RCTs lacking direct comparisons of GLP-1 receptor agonists or SGLT2 inhibitors; (4) RCTs lacking information on target outcomes; and (5) animal studies.

### 2.3. Methodological Quality Appraisal

Two independent reviewers assessed the risk of bias using the Cochrane Risk of Bias Tool 1.0 [21], achieving an inter-rater reliability score of 0.86. Discrepancies were resolved by consulting a third reviewer.

### 2.4. Outcome Definition

Due to variations in event reporting methods, the primary outcome was defined as “total hearing loss events recorded in registry systems”. Event counts rather than individual patient numbers were considered. The hearing loss included sensorineural hearing loss or conductive hearing loss because they were frequently comorbid. Since the cut-off point between complete hearing loss and hearing impairment varied widely across different regions and years, we pooled these two diagnoses into one group but did not separate them. We calculated the dropout rates, indicating study withdrawals for any reason.

In order to provide more clinically relevant information to clinicians (i.e., the potential dose-dependent risk of hearing loss), we conducted a subgroup analysis by grouping RCTs by various dosages of individual medication, which procedures had been widely accepted in various fields of research [22,23,24]. The dose definitions followed original RCT classifications [10,11,25,26,27,28,29,30,31,32,33,34,35,36,37,38,39,40,41,42,43,44,45,46,47,48,49,50]. However, if there was only one uniform dose used in the recruited RCTs, we would not subdivide them into subgroups. Rather, we will count them as single groups, such as dapagliflozin, lixisenatide, and liraglutide.

- Canagliflozin: low: 100 mg; high: 300 mg.
- Efpeglenatide: low: 4 mg; high: 6 mg.
- Ertugliflozin: low: 5 mg; high: 15 mg.
- Injectable Semaglutide: low: 0.5 mg; Medium: 1.0 mg; high: 2.4 mg.
- Empagliflozin: low: 1–10 mg; high: 25–50 mg.
- Tirzepatide: low: 5 mg; Medium: 10 mg; high: 15 mg.
- Dulaglutide: low: 0.75 mg; high: 1.5 mg

If RCTs applied an escalating titrating dosage strategy in the study design, we would use the “highest dosage (the final dosage)” as our recording. However, if RCTs consisted of a parallel low-dose arm and high-dose arm at the same time, we would use both dosages in our recording.

### 2.5. Data Extraction, Management and Conversion

Two independent authors (PT Tseng and BY Zeng) extracted relevant data, including demographics, study design, treatment details, primary outcomes, and drop-out data. Missing information was requested by the corresponding authors. Data extraction followed protocols from the Cochrane Handbook for Systematic Reviews of Interventions and other medical literature standards [51].

### 2.6. Statistical Analyses

We conducted network meta-analyses using MetaInsight (version 4.0.2, Complex Reviews Support Unit, National Institute for Health Research, London, UK), implementing a random-effects model within a Bayesian framework. The analyses were performed using the package of gemtc, BUGSNET, and bnma in R. For categorical data management, the procedures of corrections to single-zero-event studies would be operated by the MetaInsight programs [52]. We would exclude studies with zero events in both treatment and control arms to minimize bias [53,54]. Effect sizes were calculated using odds ratios (ORs) with 95% credible intervals (95% CrIs) and presented in forest plots. We performed Bayesian analysis using four Markov Chain Monte Carlo (MCMC) chains, each with a total of 25,000 iterations, discarding the first 5000 as burn-in. A thinning interval of 1 was applied, resulting in a total of 80,000 posterior samples for inference. Similarly, the relative effects of treatments compared to placebo were assigned normal priors with a mean of zero and the same precision. Model fit and consistency were assessed using residual deviance plots, per-arm residual deviance, and leverage plots. Convergence was visually inspected using Gelman convergence assessment plots for all studies.

Treatment rankings and effect sizes for both direct and indirect comparisons were generated and systematically tabulated. We assessed the consistency between direct and indirect evidence using the ‘node-splitting’ method, which is particularly valuable for network meta-analyses with available trial-level data. To evaluate the relative superiority of individual regimens, we conducted Bayesian-based surface under the cumulative ranking (SUCRA) evaluations, visualized through Litmus Rank-O-Gram and radial SUCRA plots.

Finally, we evaluated the heterogeneity with the tau value, *I^2^*, and related *p* value. Publication bias would be assessed with a funnel plot.

### 2.7. Sensitivity Analyses

To validate the reliability and convergence of treatment estimates, we employed a deviation-model analysis [55]. Specifically, each bar represents the residual deviance for an individual study arm; larger residual deviance values indicate poorer model fit for that arm in the per-arm models. Points positioned towards the upper-right corner suggest study arms with potentially poorer model fit and/or higher influence on the network meta-analysis results in the leverage plot. Further, we evaluated the overall quality of evidence in our NMA with the GRADE, which was a recognized framework to evaluate the certainty of evidence [56]. Finally, to re-validate the results of our NMA, we re-analyze our data with risk ratio (RR) and related 95% CrIs. To reduce the potential bias related to heterogeneous baseline illnesses across the recruited RCTs, we arranged subgroup analysis focusing on those RCTs recruiting subjects with a baseline illness of diabetes, which was the major indication of GLP-1 receptor agonists and SGLT2 inhibitors.

### 2.8. General Declaration

This study complies with the principles outlined in the Declaration of Helsinki.

## 3. Results

### 3.1. Eligibility of the Studies

**Figure 1:**
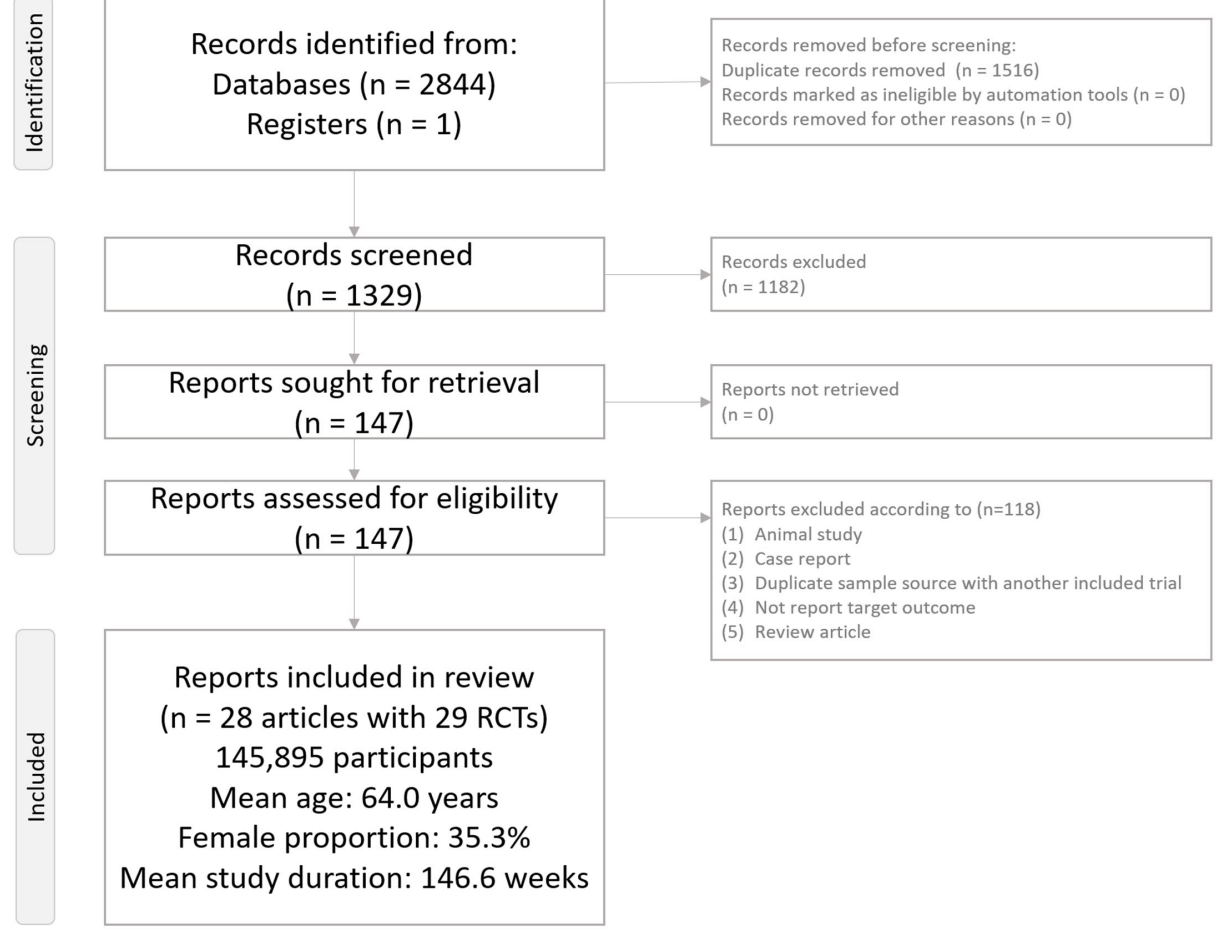
PRISMA2020 flowchart of current network meta-analysis.

Figure 1 illustrates the flowchart summarizing the literature search and screening process for this NMA. After excluding 118 articles for various reasons (Table S3), a total of 28 articles encompassing 29 RCTs were included in the analysis (Table S4) [10,11,25,26,27,28,29,30,31,32,33,34,35,36,37,38,39,40,41,42,43,44,45,46,47,48,49,50]. The selected studies involved 145,895 participants (mean age = 64.0 years, range: 50.8 to 71.9 years; mean female proportion = 35.3%, range: 20.8% to 53.6%). The average study duration was 146.6 weeks (range: 13 to 281 weeks). The investigated GLP-1 receptor agonists included tirzepatide, efpeglenatide, liraglutide, albiglutide, dulaglutide, exenatide, semaglutide, and lixisenatide. The investigated SGLT2 inhibitors included bexagliflozin, canagliflozin, empagliflozin, ertugliflozin, dapagliflozin, and sotagliflozin.

### 3.2. Primary Outcome: Incidence of Hearing Loss

**Figure 2:**
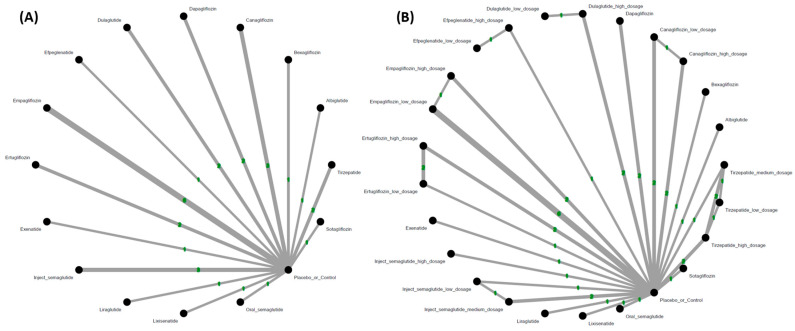
(**A**) Network structure of the primary outcome: incidence of hearing loss. The overall structure of the network meta-analysis. The lines between nodes represent direct comparisons from various trials, with the numbers over the lines indicating the number of trials providing these comparisons for each specific treatment. The thickness of the lines corresponds to the number of trials linked to the network. (**B**) Network structure of the primary outcome: incidence of hearing loss in subgroup of dosage. The overall structure of the network meta-analysis. The lines between nodes represent direct comparisons from various trials, with the numbers over the lines indicating the number of trials providing these comparisons for each specific treatment. The thickness of the lines corresponds to the number of trials linked to the network.

**Figure 3:**
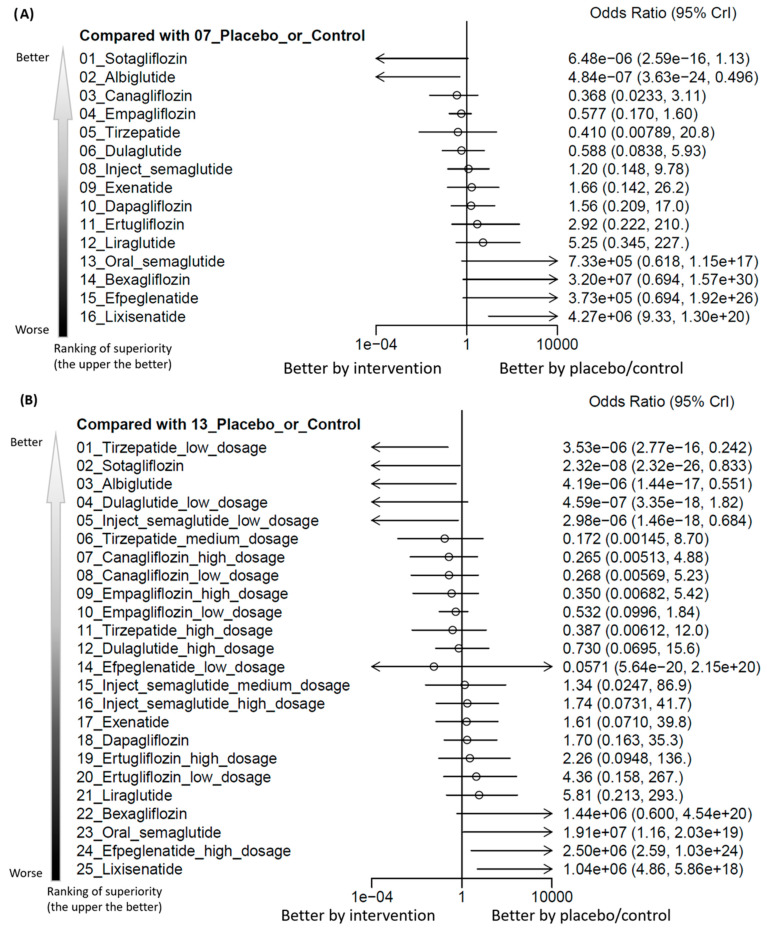
(**A**) Forest plot of primary outcome: incidence of hearing loss. When the effect size (expressed as odds ratio) is less than 1, the specified treatment is associated with fewer hearing loss events compared to placebo/controls. The arrow represents the order of ranking. (**B**) Forest plot of primary outcome: incidence of hearing loss in subgroup of dosage. When the effect size (expressed as odds ratio) is less than 1, the specified treatment is associated with fewer hearing loss events compared to placebo/controls. The arrow represents the order of ranking.

The main results of the primary outcome revealed that lixisenatide (number needed to harm = 757.75) was associated with increased events of hearing loss in comparison with the controls. In contrast, only albiglutide was associated with fewer hearing loss events than the controls. Among the investigated regimens, the risk of lixisenatide ranked the highest (Figure 2A, Figure 3A and Figure S3A, and Table 1A). There was no significant heterogeneity detected (tau < 0.001, *I^2^* = 0%, *p* value = 0.9934). The funnel plot revealed symmetry (Figure S7A).

### 3.3. Subgroup Analyses of Various Dosage

In our subgroup analysis of various dosages, lixisenatide and high-dose efpeglenatide (i.e., 6 mg/week) (number needed to harm = 757.75 and 679.00, respectively) were associated with increased events of hearing loss in comparison with the controls, whereas oral semaglutide revealed only a borderline association. In contrast, only low-dose tirzepatide, sotagliflozin, albiglutide, and low-dose injectable semaglutide were associated with fewer hearing loss events than the controls. Among the investigated regimens, the risk of lixisenatide ranked the highest (Figure 2B, Figure 3B and Figure S3B, and Table 1B). There was no significant heterogeneity detected (tau < 0.001, *I^2^* = 0%, *p* value = 0.8874). The funnel plot revealed symmetry (Figure S7B).

### 3.4. Sensitivity Analysis with Re-Validation by RR

In our sensitivity test with re-validation by RR, the main results of the current NMA would not be changed. Specifically, lixisenatide was still the only regimen associated with increased events of hearing loss in comparison with the controls (Figure S2A and Table S5A). When subgrouping according to various dosages, the lixisenatide and high-dose efpeglenatide (i.e., 6 mg/week) were still associated with increased events of hearing loss in comparison with the controls (Figure S2B and Table S5B).

### 3.5. Subgroup Analysis of RCTs Recruiting Subjects with Baseline Illness of Diabetes

In this part of the subgroup analysis, the main results of the current NMA would not be changed. Specifically, lixisenatide was still the only regimen associated with increased events of hearing loss in comparison with the controls (Figures S1A and S2C and Table S5C). When subgrouping according to various dosages, the lixisenatide and high-dose efpeglenatide (i.e., 6 mg/week) were still associated with increased events of hearing loss in comparison with the controls (Figures S1B and S2D and Table S5D).

### 3.6. Drop-Out Rate

None of the investigated regimens were associated with different drop-out rates compared to controls (Figures S1C, S2E and S3C, and Table S5E).

### 3.7. Sensitivity Analysis with Deviation-Model Evaluation

The Bayesian-based SUCRA ranking list is depicted in Table S6A–C and Figure S4A–F. The deviation-model assessment did not demonstrate significant deviation among the current NMA (Figure S5A–I).

### 3.8. Risk of Bias and Inconsistency

We identified that 81.8% (166/203 items), 17.2% (35/203 items), and 1.0% (2/203 items) of the included studies had low, unclear, and high risks of bias, respectively (Figure S6A,B). The inconsistency test, evaluating the assumption of consistency, showed no significant inconsistencies in the subgroup analysis of various dosages. In contrast, the inconsistency could not be evaluated in the main NMA because of its star-shaped in-network geometry (Table S7A–C). The overall quality of evidence of this NMA falls within the moderate-high range (Table S8A–C).

## 4. Discussion

To our knowledge, this is the first NMA addressing the potential risk of hearing loss related to GLP-1 receptor agonists and SGLT2 inhibitor use. The main results of the current NMA are summarized in Table 2. In brief, in this work, we observed that the lixisenatide and high-dose efpeglenatide, which are both exendin-4 derivatives, were associated with increased events of hearing loss in comparison with the controls.

**Figure 4:**
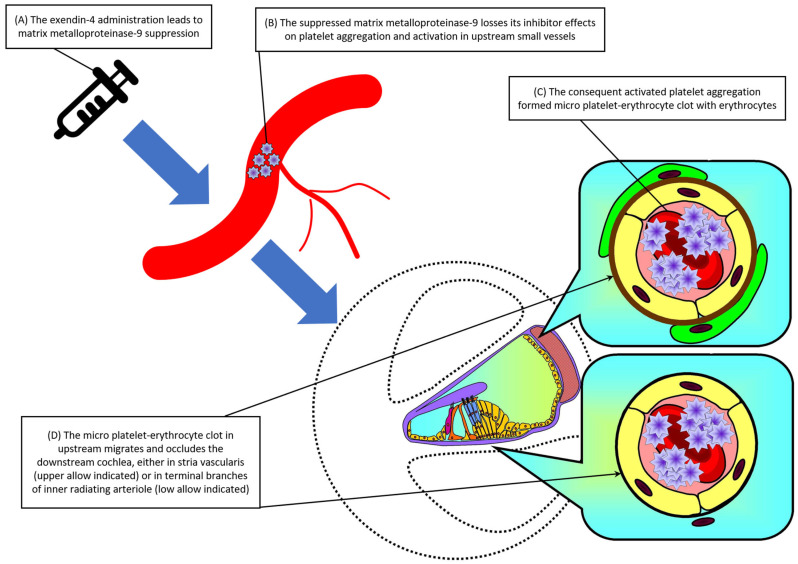
Schematic diagram of the physiopathology of exendin-4 related micro-platelet-erythrocyte clot formation. (**A**) The exendin-4 administration leads to matrix metalloproteinase-9 suppression; (**B**) the suppressed matrix metalloproteinase-9 losses its inhibitor effects on platelet aggregation and activation; (**C**) the consequent activated platelet aggregation formed micro-platelet-erythrocyte clot with erythrocytes; (**D**) finally, the upstream micro-platelet-erythrocyte clot migrates and occludes the downstream cochlea, either in stria vascularis (upper allow indicated) or in terminal branches of inner radiating arteriole (low allow indicated). Abbreviation: 95% CIs: 95% confidence intervals; GLP-1 agonist: glucagon-like peptide-1 agonist; NMA: network meta-analysis; OR: odds ratio; RCT: randomized controlled trial; SGLT2 inhibitor: sodium–glucose cotransporter 2 inhibitor.

The most important finding of the current NMA was the increased risk of hearing loss related to both exendin-4 derivative use (i.e., lixisenatide and high-dose efpeglenatide) compared to controls. Although the exendin-4 itself (i.e., exenatide) did not achieve statistical significance, it still had a trend of increased risk of hearing loss (Figure 3). This issue was clinically relevant since subjects who needed such medications had underlying risk factors of hearing loss [19]. Although there is little knowledge regarding the etiology of hearing loss, the disturbance to micro-circulation (i.e., labyrinthine artery) in the inner ear, especially the cochlea, was one of the important etiologies [57]. The disturbance to micro-circulation in the inner ear might result from the formation of a micro-platelet-erythrocyte clot, which frequently contributes to sudden sensorineural hearing loss [7,8,9]. Specifically, the formation of a micro-platelet-erythrocyte clot, either related to autoimmune diseases or medication adverse effects, would follow the blood flow and result in occlusion in the small arterioles or micro-circulation in the end organ. This thromboembolism would finally lead to ischemic damage to the affected organs, specifically the audiologic system in this NMA. There is accumulating evidence addressing the potentially increased risk of formation of a micro-platelet-erythrocyte clot related to exendin-4 derivative use. For example, Kuroki and colleagues found that exendin-4 administration would inhibit the matrix metalloproteinase-9 activation in the animal model, with the suppressing effects persisting at least 7 days [4]. Researchers have demonstrated that the matrix metalloproteinase-9 could inhibit or prolong platelet aggregation and prevent the platelet plug formation in in vitro and in vivo studies [58]. Further, matrix metalloproteinases-9 has been found to be abundant in the resistance-sized (approximately 200 µm) arteries [59]. The cochlear micro-circulation vessels (approximately diameters of 9.1 ± 0.8 µm) were in the downstream of the aforementioned resistance-sized arteries [60] so that the formation of micro-platelet-erythrocyte clots in the upstream would result in occlusion in the downstream vessels. Taken together, the suppressed matrix metalloproteinase-9 activity related to exendin-4 administration would finally result in the pro-thromboembolism environment in the micro-circulation in the inner ear [4,5]. Regardless of the potential of anti-inflammatory properties of GLP-1 receptor agonists [61] and SGLT2 inhibitors [62] to ameliorate hearing impairment [7,8,9,63], this physiopathology might not be able to reverse the potential ototoxicity effect mentioned above. We had drawn a schematic diagram to depict the physiopathology of exendin-4 related micro-platelet-erythrocyte clot formation (Figure 4). However, as addressed before, the hypothesis remained theoretical and lacked direct supporting evidence. Future direct human trials should be warranted to provide more information regarding the pro-thromboembolic effects related to exendin-4 derivative use.

### Strengths and Limitations

This NMA offers several methodological strengths that enhance the reliability and clinical utility of our findings. First, the NMA designation could enable statistical comparisons between different GLP-1 receptor agonists and SGLT2 inhibitors, providing more comprehensive evidence than RCTs or traditional pairwise meta-analyses. Our rigorous methodology included exclusively focusing on RCTs, ensuring high-quality evidence while minimizing potential bias. By specifically excluding participants with pre-existing hearing loss at baseline, we were able to isolate true causative or prophylactic effects. Furthermore, our detailed subgroup analyses across various dosages offer clinicians granular evidence to select the optimal dosage for specific patient populations who already had risk factors of hearing loss at baseline.

Some limitations warrant consideration. First, there were only a few studies addressing hearing loss related to GLP-1 receptor agonists or SGLT2 inhibitor use so that the included RCTs and recruited participants were relatively few (29 RCTs involving 145,895 participants). Second, our stringent focus on RCTs, while ensuring methodological rigor, potentially excluded valuable observational data from long-term cohort studies. Third, the variation in diagnostic approaches across multi-country trials presents a notable limitation. The lack of structural and uniform diagnostic methods to detect hearing loss may have introduced heterogeneity in case identification, potentially affecting the precision of our effect estimates. Although meta-analyses may raise concerns about “inconsistencies in diagnostic tools and baseline illnesses”, the nature of these studies is rooted in large databases or numerous clinical studies, making them more reflective of clinical experience. Therefore, many meta-analyses on drug side effects [15,16] have become a benchmark for guiding future research [64,65]. Fourth, the star-shaped network geometry in the main outcome would limit the evaluation of inconsistency. Fifth, the main results of this NMA mainly came from a few RCTs [10,11] so that the clinicians should pay special attention when interpreting the results of our study in their clinical practice. Finally, all the recruited RCTs were designed based on various underlying diseases (i.e., diabetes or obesity) but not specifically to examine hearing loss occurrence, which might lead to ignorance of the occurrence of mild hearing loss. Because adverse effects data are often handled with less rigor than the primary beneficial outcomes of a study, incomplete reporting might still exist when synthesizing data. Therefore, future studies using various audiometric tests to routinely follow patients’ hearing function should be warranted. These limitations suggest the need for large-scale studies specifically designed to assess causative or preventive effects in hearing loss.

## 5. Conclusions

This comprehensive NMA reveals a potential relationship between hearing loss and exendin-4 derivative prescription (i.e., lixisenatide and high-dose efpeglenatide). Since hearing loss is an irreversible disease and subjects who need such medications had underlying risk factors of hearing loss, this potentially ototoxicity related to exendin-4 derivative prescription would be important for clinicians to choose the most suitable regimens for patients with an underlying risk of hearing loss. Future research should prioritize large-scale studies specifically designed to assess causative or preventive effects in hearing loss.

## Figures and Tables

**Table 1:** (**A**) League table of the primary outcome: incidence of hearing loss. (**B**) League table of the primary outcome: incidence of hearing loss in subgroup of dosage.

(A)																														
01_Sotagliflozin												0.17 [0.01; 4.10]																		
0.22 (0, 2286941337189.82)		02_Albiglutide										0.20 [0.01; 4.16]																		
0 (0, 2.56)		0 (0, 2.39)		03_Canagliflozin								0.56 [0.11; 2.90]																		
0 (0, 1.05)		0 (0, 1.16)		0.65 (0.04, 7.39)		04_Empagliflozin						0.63 [0.32; 1.23]																		
0 (0, 2.87)		0 (0, 3.35)		0.81 (0.01, 56.86)		1.21 (0.02, 65.45)		05_Tirzepatide				0.41 [0.04; 3.98]																		
0 (0, 1.03)		0 (0, 1.15)		0.61 (0.02, 10.65)		0.96 (0.06, 9.25)		0.77 (0.01, 70.74)		06_Dulaglutide		0.52 [0.12; 2.26]																		
**0 (0, 0.57)**		**0 (0, 0.54)**		0.38 (0.02, 3.16)		0.57 (0.17, 1.58)		0.46 (0.01, 21.69)		0.59 (0.08, 5.9)		07_Placebo_ or_Control	0.89 [0.22; 3.62]	0.66 [0.11; 3.98]		0.80 [0.19; 3.36]		0.66 [0.10; 4.21]		0.25 [0.03; 2.23]		0.60 [0.03; 12.47]			0.34 [0.01; 8.30]		0.40 [0.02; 8.35]		0.11 [0.01; 2.06]	
**0 (0, 0.5)**		**0 (0, 0.62)**		0.3 (0.01, 5.56)		0.48 (0.04, 4.22)		0.4 (0.01, 31.86)		0.48 (0.03, 10.47)		0.84 (0.11, 5.84)	08_Inject_ semaglutide																	
**0 (0, 0.53)**		**0 (0, 0.57)**		0.24 (0.01, 5.69)		0.37 (0.02, 5.35)		0.29 (0, 33.06)		0.38 (0.02, 11.35)		0.66 (0.05, 7.81)	0.8 (0.03, 20.52)	09_Exenatide																
**0 (0, 0.5)**		**0 (0, 0.52)**		0.24 (0.01, 4.26)		0.37 (0.02, 3.22)		0.3 (0, 21.2)		0.38 (0.02, 7.12)		0.65 (0.06, 4.68)	0.8 (0.03, 11.95)	0.99 (0.03, 23.27)		10_Dapagliflozin														
**0 (0, 0.28)**		**0 (0, 0.33)**		0.12 (0, 2.92)		0.18 (0, 2.72)		0.15 (0, 14.28)		0.19 (0, 5.45)		0.33 (0.01, 3.85)	0.39 (0.01, 9.76)	0.49 (0.01, 17.46)		0.52 (0.01, 15.02)		11_Ertugliflozin												
**0 (0, 0.14)**		**0 (0, 0.16)**		0.06 (0, 1.78)		0.1 (0, 1.72)		0.07 (0, 9.52)		0.1 (0, 3.85)		0.18 (0, 2.6)	0.2 (0, 6.43)	0.25 (0, 11.63)		0.26 (0, 10.68)		0.51 (0.01, 45.3)		12_Liraglutide										
**0 (0, 0)**		**0 (0, 0)**		**0 (0, 0.33)**		**0 (0, 0.53)**		**0 (0, 0.73)**		**0 (0, 0.44)**		**0 (0, 0.93)**	0 (0, 1.29)	0 (0, 2.04)		0 (0, 1.92)		0 (0, 4.23)		0 (0, 5.95)		13_Oral_ semaglutide								
**0 (0, 0)**		**0 (0, 0)**		**0 (0, 0.41)**		**0 (0, 0.57)**		0 (0, 1.03)		**0 (0, 0.75)**		**0 (0, 0.95)**	0 (0, 1.62)	0 (0, 1.89)		0 (0, 1.97)		0 (0, 5.04)		0 (0, 10.18)		145.21 (0, 15638204434817468416)			14_Bexagliflozin					
**0 (0, 0)**		**0 (0, 0)**		**0 (0, 0.29)**		**0 (0, 0.27)**		**0 (0, 0.49)**		**0 (0, 0.39)**		**0 (0, 0.49)**	**0 (0, 0.7)**	**0 (0, 0.88)**		0 (0, 1.03)		0 (0, 3.33)		0 (0, 5.35)		1.53 (0, 39759673530850082816)			0.02 (0, 642665163278770)		15_Efpeglenatide			
**0 (0, 0)**		**0 (0, 0)**		**0 (0, 0.05)**		**0 (0, 0.06)**		**0 (0, 0.07)**		**0 (0, 0.07)**		**0 (0, 0.1)**	**0 (0, 0.18)**	**0 (0, 0.2)**		**0 (0, 0.21)**		**0 (0, 0.38)**		0 (0, 1.4)		0.52 (0, 41443130473760)			0 (0, 174818703323.2)		0.02 (0, 16044177798929.8)		16_Lixisenatide	
(**B**)																														
01_Tirzepatide_ low_dosage							0.33 [0.01; 8.14]						0.34 [0.01; 8.48]																	
174.27 (0, 10463610754211418112)	02_Sotagliflozin														0.17 [0.01; 4.10]															
0.94 (0, 23865462628940.6)	0.02 (0, 83814535670.54)		03_Albiglutide												0.20 [0.01; 4.16]															
7.18 (0, 10384045242332)	0.02 (0, 76625689170891.9)		2.79 (0, 226025279542229)	04_Dulaglutide_ low_dosage										0.33 [0.01; 8.22]																
1.17 (0, 93257222307134.8)	0 (0, 592940298063597)		1.01 (0, 131811533539368)	0.7 (0, 181440300040.18)	05_Inject_ semaglutide_ low_dosage										0.66 [0.03; 16.34]			0.34 [0.01; 8.27]												
**0 (0, 0.79)**	0 (0, 8.45)		0 (0, 25.84)	0 (0, 27.45)	0 (0, 10.56)		06_Tirzepatide_ medium_ dosage						1.01 [0.10; 9.75]		0.34 [0.01; 8.43]															
0 (0, 2.37)	0 (0, 5.31)		0 (0, 16.31)	0 (0, 16.7)	0 (0, 6.32)		0.68 (0, 146.15)	07_Canagliflozin_ high_dosage	1.00 [0.06; 15.96]						0.62 [0.08; 5.08]															
0 (0, 2.2)	0 (0, 5.17)		0 (0, 4.46)	0 (0, 17.79)	0 (0, 6.1)		0.63 (0, 154.69)	0.94 (0.01, 59.33)	08_Canagliflozin_ low_dosage						0.62 [0.08; 5.07]															
0 (0, 1.31)	0 (0, 6.04)		0 (0, 4.16)	0 (0, 12.78)	0 (0, 4.37)		0.53 (0, 117.71)	0.76 (0.01, 103.71)	0.79 (0.01, 100.39)		09_Empagliflozin _high_dosage	1.00 [0.06; 15.98]			0.62 [0.08; 5.07]															
**0 (0, 0.57)**	0 (0, 1.87)		0 (0, 1.29)	0 (0, 4.23)	0 (0, 1.53)		0.34 (0, 24.29)	0.51 (0.01, 14.49)	0.53 (0.01, 14.92)		0.69 (0.01, 13.22)	10_Empagliflozin _low_dosage			0.64 [0.33; 1.26]															
**0 (0, 0.4)**	0 (0, 3.6)		0 (0, 3.88)	0 (0, 8.59)	0 (0, 3.25)		0.43 (0.01, 11.28)	0.6 (0, 81.02)	0.69 (0, 99.71)		0.87 (0, 102.78)	1.33 (0.03, 103.38)	11_Tirzepatide_ high_dosage		0.99 [0.10; 9.57]															
**0 (0, 0.44)**	0 (0, 1.44)		**0 (0, 0.94)**	0 (0, 1.32)	0 (0, 1.14)		0.21 (0, 20.54)	0.33 (0, 16.23)	0.34 (0, 14.65)		0.45 (0, 15.63)	0.72 (0.02, 9.31)	0.48 (0, 31.08)	12_Dulaglutide_ high_dosage	0.61 [0.14; 2.61]															
**0 (0, 0.24)**	**0 (0, 0.83)**		**0 (0, 0.55)**	0 (0, 1.82)	**0 (0, 0.68)**		0.17 (0, 8.7)	0.26 (0.01, 4.88)	0.27 (0.01, 5.23)		0.35 (0.01, 5.42)	0.53 (0.1, 1.84)	0.39 (0.01, 12.01)	0.73 (0.07, 15.56)	13_Placebo_ or_Control			0.71 [0.07; 6.80]	0.67 [0.11; 3.99]	0.66 [0.11; 3.98]	0.80 [0.19; 3.36]		0.99 [0.10; 9.54]	0.43 [0.06; 2.90]	0.25 [0.03; 2.23]	0.34 [0.01; 8.30]	0.60 [0.03; 12.47]	0.20 [0.01; 4.17]		0.11 [0.01; 2.06]
0 (0, 2749665048400134)	0 (0, 13835349680732138)		0 (0, 4106605056265394)	0 (0, 416046115627.38)	0 (0, 247794060699236)		3.36 (0, 1747509744556715776)	4.57 (0, 3863746537860638720)	4.97 (0, 5275996480618600448)		5.3 (0, 4429532891571235840)	8.65 (0, 7964217336657846272)	8.04 (0, 6558691788735834112)	14.84 (0, 11731741958374318080)	17.51 (0, 17741192217616175104)		14_Efpeglenatide _low_dosage											0.20 [0.01; 4.16]		
**0 (0, 0.44)**	0 (0, 1.6)		**0 (0, 0.88)**	0 (0, 2.9)	**0 (0, 0.57)**		0.1 (0, 38.54)	0.16 (0, 29.55)	0.17 (0, 26.76)		0.23 (0, 30.52)	0.38 (0, 25.55)	0.25 (0, 60.04)	0.52 (0, 78.93)	0.75 (0.01, 40.49)		0.04 (0, 80325932675121217536)	15_Inject_ semaglutide_ medium_dosage												
**0 (0, 0.24)**	**0 (0, 0.64)**		**0 (0, 0.54)**	0 (0, 1.75)	**0 (0, 0.65)**		0.1 (0, 14.57)	0.15 (0, 10.53)	0.16 (0, 11.04)		0.2 (0, 12.12)	0.3 (0.01, 8.21)	0.22 (0, 24.01)	0.41 (0.01, 38.67)	0.57 (0.02, 13.68)		0.03 (0, 2.01122131702702e+20)	0.8 (0, 147.22)	16_Inject_ semaglutide_ high_dosage											
**0 (0, 0.26)**	**0 (0, 0.88)**		**0 (0, 0.58)**	0 (0, 1.74)	**0 (0, 0.72)**		0.09 (0, 15.53)	0.15 (0, 12.22)	0.15 (0, 11.81)		0.2 (0, 12.92)	0.32 (0.01, 8.36)	0.23 (0, 23.58)	0.46 (0.01, 39.17)	0.62 (0.03, 14.08)		0.03 (0, 1.7925843597753e+20)	0.89 (0.01, 179.63)	1.06 (0.01, 84.22)	17_Exenatide										
**0 (0, 0.2)**	**0 (0, 0.64)**		**0 (0, 0.47)**	0 (0, 1.19)	**0 (0, 0.56)**		0.09 (0, 9.19)	0.14 (0, 6.29)	0.15 (0, 6.62)		0.18 (0, 7.19)	0.31 (0.01, 3.84)	0.2 (0, 13.5)	0.43 (0.01, 19.45)	0.59 (0.03, 6.15)		0.03 (0, 1.01388022129584e+20)	0.81 (0.01, 84.95)	1.01 (0.01, 46.22)	0.96 (0.01, 45.76)	18_Dapagliflozin									
**0 (0, 0.18)**	**0 (0, 0.63)**		**0 (0, 0.37)**	0 (0, 1.15)	**0 (0, 0.54)**		0.06 (0, 13.86)	0.1 (0, 8.88)	0.1 (0, 9.66)		0.13 (0, 10.34)	0.23 (0, 6.78)	0.15 (0, 18.82)	0.29 (0, 27.46)	0.44 (0.01, 10.54)		0.02 (0, 54300857840542908416)	0.55 (0, 99.78)	0.74 (0, 63.75)	0.65 (0, 62.92)	0.73 (0.01, 60.62)		19_Ertugliflozin_ high_dosage	0.58 [0.07; 5.15]						
**0 (0, 0.09)**	**0 (0, 0.33)**		**0 (0, 0.21)**	**0 (0, 0.76)**	**0 (0, 0.27)**		0.03 (0, 6.65)	0.05 (0, 4.94)	0.05 (0, 5.09)		0.07 (0, 6.1)	0.11 (0, 3.75)	0.08 (0, 10.64)	0.17 (0, 17.44)	0.23 (0, 6.33)		0.01 (0, 27862512272519688192)	0.29 (0, 67.19)	0.37 (0, 40.33)	0.35 (0, 41.08)	0.37 (0, 39.43)		0.53 (0.03, 12.71)	20_Ertugliflozin_ low_dosage						
**0 (0, 0.09)**	**0 (0, 0.2)**		**0 (0, 0.19)**	**0 (0, 0.59)**	**0 (0, 0.23)**		0.03 (0, 5.33)	0.04 (0, 3.56)	0.04 (0, 3.63)		0.06 (0, 4.11)	0.09 (0, 2.84)	0.07 (0, 8.4)	0.13 (0, 13.58)	0.17 (0, 4.7)		0.01 (0, 17672406927695316992)	0.23 (0, 47.84)	0.29 (0, 29.33)	0.28 (0, 29.45)	0.3 (0, 29.33)		0.4 (0, 73.46)	0.74 (0, 141.71)	21_Liraglutide					
**0 (0, 0)**	**0 (0, 0)**		**0 (0, 0.01)**	**0 (0, 0.01)**	**0 (0, 0)**		**0 (0, 0.53)**	**0 (0, 0.89)**	**0 (0, 0.63)**		**0 (0, 0.58)**	**0 (0, 0.89)**	**0 (0, 0.98)**	0 (0, 1.67)	0 (0, 1.67)		0 (0, 12286429074044.5)	0 (0, 3.79)	0 (0, 4.67)	0 (0, 4.48)	0 (0, 4.1)		0 (0, 6.99)	0 (0, 14.67)	0 (0, 19.44)	22_Bexagliflozin				
**0 (0, 0)**	**0 (0, 0)**		**0 (0, 0)**	**0 (0, 0)**	**0 (0, 0)**		**0 (0, 0.35)**	**0 (0, 0.35)**	**0 (0, 0.33)**		**0 (0, 0.51)**	**0 (0, 0.46)**	**0 (0, 0.56)**	**0 (0, 0.93)**	**0 (0, 0.86)**		0 (0, 120950538640435856)	0 (0, 4.43)	0 (0, 2.1)	0 (0, 2.36)	0 (0, 2.02)		0 (0, 7.57)	0 (0, 12.28)	0 (0, 8.89)	1.21 (0, 1747737841683757)	23_Oral_ semaglutide			
**0 (0, 0)**	**0 (0, 0)**		**0 (0, 0)**	**0 (0, 0)**	**0 (0, 0.01)**		**0 (0, 0.22)**	**0 (0, 0.21)**	**0 (0, 0.2)**		**0 (0, 0.18)**	**0 (0, 0.2)**	**0 (0, 0.33)**	**0 (0, 0.46)**	**0 (0, 0.39)**		**0 (0, 0.43)**	0 (0, 1.86)	**0 (0, 0.98)**	0 (0, 1.04)	**0 (0, 0.99)**		0 (0, 1.9)	0 (0, 4)	0 (0, 5.11)	0.09 (0, 25965618197430972)	0.92 (0, 466713242374613)	24_Efpeglenatide _high_dosage		
**0 (0, 0)**	**0 (0, 0)**		**0 (0, 0)**	**0 (0, 0)**	**0 (0, 0)**		**0 (0, 0.09)**	**0 (0, 0.1)**	**0 (0, 0.09)**		**0 (0, 0.11)**	**0 (0, 0.11)**	**0 (0, 0.2)**	**0 (0, 0.25)**	**0 (0, 0.21)**		0 (0, 20563509532733)	**0 (0, 0.82)**	**0 (0, 0.56)**	**0 (0, 0.68)**	**0 (0, 0.61)**		0 (0, 1.08)	0 (0, 2.14)	0 (0, 2.52)	2.06 (0, 6625401557077732)	6.09 (0, 2608743386738966)	8.01 (0, 65039373111329704)		25_Lixisenatide

(**A**) Data presented as OR [95% CIs]. Pairwise (upper-right portion) and network (lower-left portion) meta-analysis results are presented as estimate effect sizes for the outcome of overall events of hearing loss. The gray-background indicated the interventions. Interventions are reported in order of mean ranking of beneficial prophylactic effects on overall events of hearing loss, and outcomes are expressed as odds ratio (OR) (95% confidence intervals) (95% CIs). For the pairwise meta-analyses, OR of less than 1 indicates that the treatment specified in the row achieved a more beneficial effect than that specified in the column. For the network meta-analysis (NMA), OR of less than 1 indicates that the treatment specified in the column achieved a more beneficial effect than that specified in the row. Bold results indicate statistical significance. (**B**) Data presented as OR [95% CIs]. Pairwise (upper-right portion) and network (lower-left portion) meta-analysis results are presented as estimated effect sizes for the outcome of hearing loss events in a subgroup of dosage. The gray-background indicated the interventions. Interventions are reported in order of mean ranking of beneficial prophylactic effect on events of hearing loss in a subgroup of dosage, and outcomes are expressed as odds ratio (OR) (95% confidence intervals) (95% CIs). For the pairwise meta-analyses, OR of less than 1 indicates that the treatment specified in the row achieved a more beneficial effect than that specified in the column. For the network meta-analysis (NMA), OR of less than 1 indicates that the treatment specified in the column achieved a more beneficial effect than that specified in the row. Bold results indicate statistical significance. Abbreviation: 95% CIs: 95% confidence intervals; GLP-1 agonist: glucagon-like peptide-1 agonist; NMA: network meta-analysis; OR: odds ratio; RCT: randomized controlled trial; SGLT2 inhibitor: sodium–glucose cotransporter 2 inhibitor.

**Table 2:** Summary of main findings in this network meta-analysis.

Medications	Overall	Subgroup of Dosage
Lixisenatide	Significantly increased events of hearing loss/highest risk	Significantly increased events of hearing loss/highest risk
Efpeglenatide	Not significant	High-dose efpeglenatide (i.e., 6 mg/week) significantly increased events of hearing loss

## Data Availability

The data presented in this study are available on request from the corresponding author. The data are not publicly available due to all the data of this study were extracted from an open-source website (https://clinicaltrials.gov/) (accessed on 28 October 2024).

## Supplementary Materials

The following supporting information can be downloaded at: https://www.mdpi.com/article/10.3390/ph18050735/s1, Figure S1: (A) Network structure of primary outcome: incidence of hearing loss (focus on diabetes), (B) Network structure of primary outcome: incidence of hearing loss in subgroup of dosage (focus on diabetes) (C) Network structure of NMA of drop-out rate; Figure S2: (A) Forest plot of primary outcome: incidence of hearing loss (risk ratio), (B) Forest plot of primary outcome: incidence of hearing loss in subgroup of dosage (risk ratio), (C) Forest plot of primary outcome: incidence of hearing loss (focus on diabetes), (D) Forest plot of primary outcome: incidence of hearing loss in subgroup of dosage (focus on diabetes),(E) Forest plot of NMA of drop-out rate; Figure S3: (A) Individual study result of primary outcome: incidence of hearing loss, (B) Individual study result of primary outcome: incidence of hearing loss in subgroup of dosage, (C) Individual study result of drop-out rate; Figure S4: (A) Bayesian-based Litmus Rank-O-Gram rank plot of primary outcome: incidence of hearing loss, (B) Bayesian-based radial surface under the cumulative ranking of primary outcome: incidence of hearing loss, (C) Bayesian-based Litmus Rank-O-Gram rank plot of primary outcome: incidence of hearing loss in subgroup of dosage, (D) Bayesian-based radial surface under the cumulative ranking of primary outcome: incidence of hearing loss in subgroup of dosage, (E) Bayesian-based Litmus Rank-O-Gram rank plot of drop-out rate, (F) Bayesian-based radial surface under the cumulative ranking of drop-out rate; Figure S5: (A) Bayesian-based residual deviance NMA/UME model of primary outcome: incidence of hearing loss, (B) Bayesian-based per-arm residual deviance of primary outcome: incidence of hearing loss, (C) Bayesian-based leverage plot of primary outcome: incidence of hearing loss, (D) Bayesian-based residual deviance NMA/UME model of primary outcome: incidence of hearing loss in subgroup of dosage, (E) Bayesian-based per-arm residual deviance of primary outcome: incidence of hearing loss in subgroup of dosage, (F) Bayesian-based leverage plot of primary outcome: incidence of hearing loss in subgroup of dosage, (G) Bayesian-based residual deviance NMA/UME model of drop-out rate, (H) Bayesian-based per-arm residual deviance of drop-out rate, (I) Bayesian-based leverage plot of drop-out rate; Figure S6: (A) Overview of risk of bias, (B) Detailed risk of bias in each study; Figure S7: (A) Funnel plot of the primary outcome: incidence of hearing loss, (B) Funnel plot of the primary outcome: incidence of hearing loss in subgroup of dosage; Table S1: PRISMA 2020 checklist of the current network meta-analysis; Table S2: Keyword used in each database and search results; Table S3: Excluded studies and reason; Table S4: Characteristics of the included studies; Table S5: (A) League table of NMA of primary outcome: incidence of hearing loss (risk ratio), (B) League table of NMA of primary outcome: incidence of hearing loss in subgroup of dosage (risk ratio), (C) League table of NMA of primary outcome: incidence of hearing loss (focus on diabetes), (D) League table of NMA of primary outcome: incidence of hearing loss in subgroup of dosage (focus on diabetes), (E) League table of NMA of drop-out rate; Table S6: (A) SUCRA (Surface under the cumulative ranking) of primary outcome: incidence of hearing loss, (B) SUCRA (Surface under the cumulative ranking) of primary outcome: incidence of hearing loss in subgroup of dosage, (C) SUCRA (Surface under the cumulative ranking) of drop-out rate; Table S7: (A) Inconsistency within the network meta-analysis of primary outcome: incidence of hearing loss, (B) Inconsistency within the network meta-analysis of primary outcome: incidence of hearing loss in subgroup of dosage, (C) Inconsistency within the network meta-analysis of drop-out rate; Table S8: (A) GRADE of primary outcome: incidence of hearing loss, (B) GRADE of primary outcome: incidence of hearing loss in subgroup of dosage, (C) GRADE of drop-out rate. (References in Supplementary Materials [10,11,18,19,25,26,27,28,29,30,31,32,33,34,35,36,37,38,39,40,41,42,43,44,45,46,47,48,49,50,66,67,68,69,70,71,72,73,74,75,76,77,78,79,80,81,82,83,84,85,86,87,88,89,90,91,92,93,94,95,96,97,98,99,100,101,102,103,104,105,106,107,108,109,110,111,112,113,114,115,116,117,118,119,120,121,122,123,124,125,126,127,128,129,130,131,132,133,134,135,136,137,138,139,140,141,142,143,144,145,146,147,148,149,150,151,152,153,154,155,156,157,158,159,160,161,162,163,164,165,166,167,168,169,170,171,172,173,174,175,176,177,178,179,180,181,182]).
