# Supplementary material for: Risk of Hearing Loss in Patients Treated with Exendin-4 Derivatives: A Network Meta-Analysis of Glucagon-like Peptide-1 Receptor Agonists and Sodium–Glucose Cotransporter 2 Inhibitors
Source: Pharmaceuticals (Basel). 2025 May 16;18(5):735. doi: 10.3390/ph18050735 (PMC12115298; doi:10.3390/ph18050735)
Supplement: Supplementary file 1 [file pharmaceuticals-18-00735-s001.zip › pharmaceuticals-3567879-supplementary.pdf]

List of content in supplement materials

Risk of Hearing Loss in Patients Treated with Exendin-4 Derivatives: A Network Meta-Analysis of Glucagon-Like Peptide-1 Receptor Agonists and Sodium–Glucose Cotransporter 2 Inhibitors

Figure S1	(A) Network structure of primary outcome: incidence of hearing loss (focus on diabetes)
	(B) Network structure of primary outcome: incidence of hearing loss in subgroup of dosage (focus on diabetes)
	(C) Network structure of NMA of drop-out rate
Figure S2	(A) Forest plot of primary outcome: incidence of hearing loss (risk ratio)
	(B) Forest plot of primary outcome: incidence of hearing loss in subgroup of dosage (risk ratio)
	(C) Forest plot of primary outcome: incidence of hearing loss (focus on diabetes)
	(D) Forest plot of primary outcome: incidence of hearing loss in subgroup of dosage (focus on diabetes)
	(E) Forest plot of NMA of drop-out rate
Figure S3	(A) Individual study result of primary outcome: incidence of hearing loss
	(B) Individual study result of primary outcome: incidence of hearing loss in subgroup of dosage
	(C) Individual study result of drop-out rate
Figure S4	(A) Bayesian-based Litmus Rank-O-Gram rank plot of primary outcome: incidence of hearing loss
	(B) Bayesian-based radial surface under the cumulative ranking of primary outcome: incidence of hearing loss

	(C) Bayesian-based Litmus Rank-O-Gram rank plot of primary outcome: incidence of hearing loss in subgroup of dosage
	(D) Bayesian-based radial surface under the cumulative ranking of primary outcome: incidence of hearing loss in subgroup of dosage
	(E) Bayesian-based Litmus Rank-O-Gram rank plot of drop-out rate
	(F) Bayesian-based radial surface under the cumulative ranking of drop-out rate
Figure S5	(A) Bayesian-based residual deviance NMA/UME model of primary outcome: incidence of hearing loss
	(B) Bayesian-based per-arm residual deviance of primary outcome: incidence of hearing loss
	(C) Bayesian-based leverage plot of primary outcome: incidence of hearing loss
	(D) Bayesian-based residual deviance NMA/UME model of primary outcome: incidence of hearing loss in subgroup of dosage
	(E) Bayesian-based per-arm residual deviance of primary outcome: incidence of hearing loss in subgroup of dosage
	(F) Bayesian-based leverage plot of primary outcome: incidence of hearing loss in subgroup of dosage
	(G) Bayesian-based residual deviance NMA/UME model of drop-out rate
	(H) Bayesian-based per-arm residual deviance of drop-out rate
	(I) Bayesian-based leverage plot of drop-out rate
Figure S6	(A) Overview of risk of bias
	(B) Detailed risk of bias in each study
Figure S7	(A) Funnel plot of the primary outcome: incidence of hearing loss
	(B) Funnel plot of the primary outcome: incidence of hearing loss in subgroup of dosage
Table S1	PRISMA 2020 checklist of the current network meta-analysis
Table S2	Keyword used in each database and search results
Table S3	Excluded studies and reason
Table S4	Characteristics of the included studies
Table S5	(A) League table of NMA of primary outcome: incidence of hearing loss (risk ratio)
	(B) League table of NMA of primary outcome: incidence of hearing loss in subgroup of dosage (risk ratio)
	(C) League table of NMA of primary outcome: incidence of hearing loss (focus on diabetes)

	(D) League table of NMA of primary outcome: incidence of hearing loss in subgroup of dosage (focus on diabetes)
	(E) League table of NMA of drop-out rate
Table S6	(A) SUCRA (Surface under the cumulative ranking) of primary outcome: incidence of hearing loss
	(B) SUCRA (Surface under the cumulative ranking) of primary outcome: incidence of hearing loss in subgroup of dosage
	(C) SUCRA (Surface under the cumulative ranking) of drop-out rate
Table S7	(A) Inconsistency within the network meta-analysis of primary outcome: incidence of hearing loss
	(B) Inconsistency within the network meta-analysis of primary outcome: incidence of hearing loss in subgroup of dosage
	(C) Inconsistency within the network meta-analysis of drop-out rate
Table S8	(A) GRADE of primary outcome: incidence of hearing loss
	(B) GRADE of primary outcome: incidence of hearing loss in subgroup of dosage
	(C) GRADE of drop-out rate

Figure S1A network structure of the primary outcome: incidence of hearing loss (focus on diabetes)

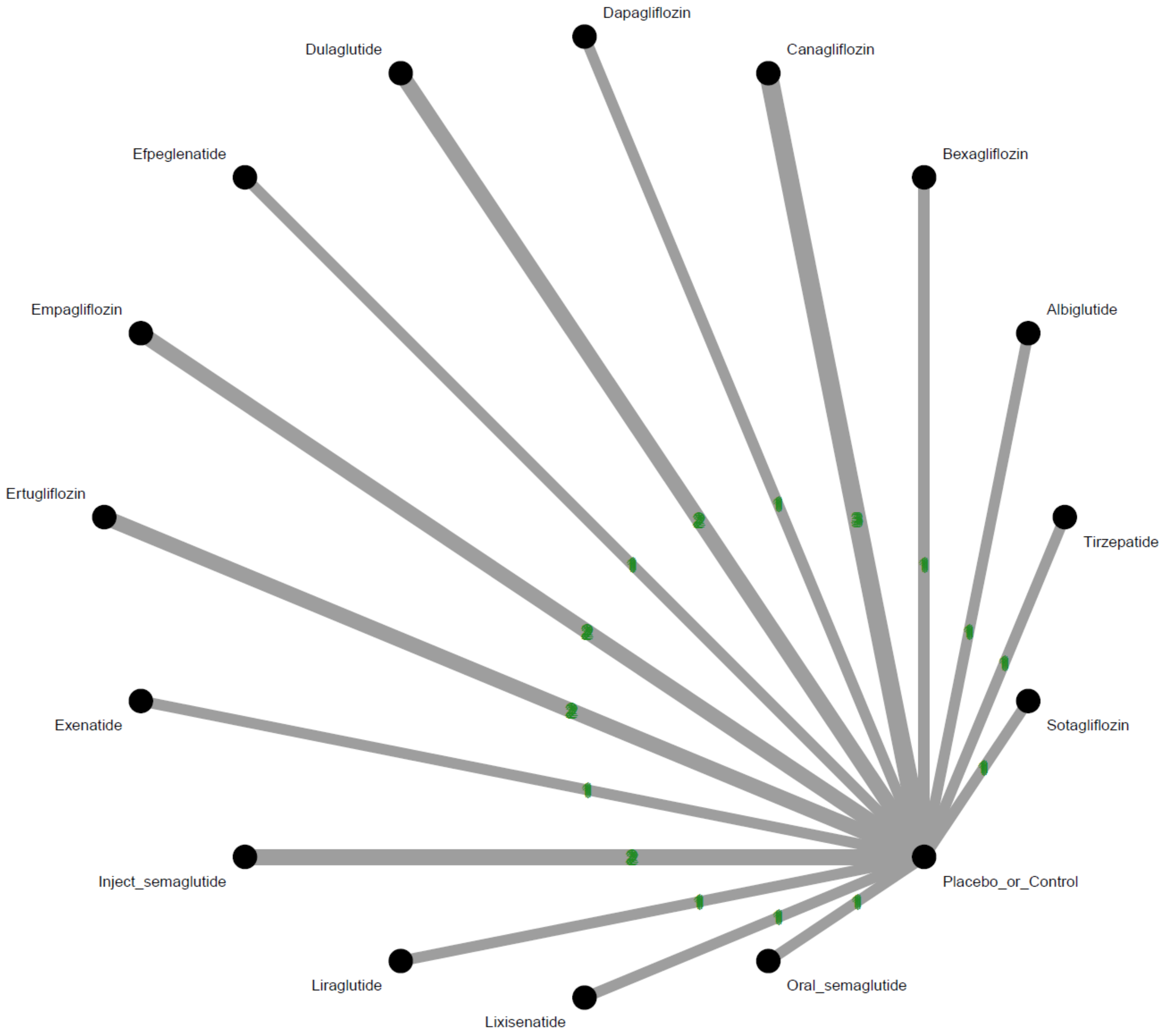

Figure S1B network structure of the primary outcome: incidence of hearing loss in subgroup of dosage (focus on diabetes)

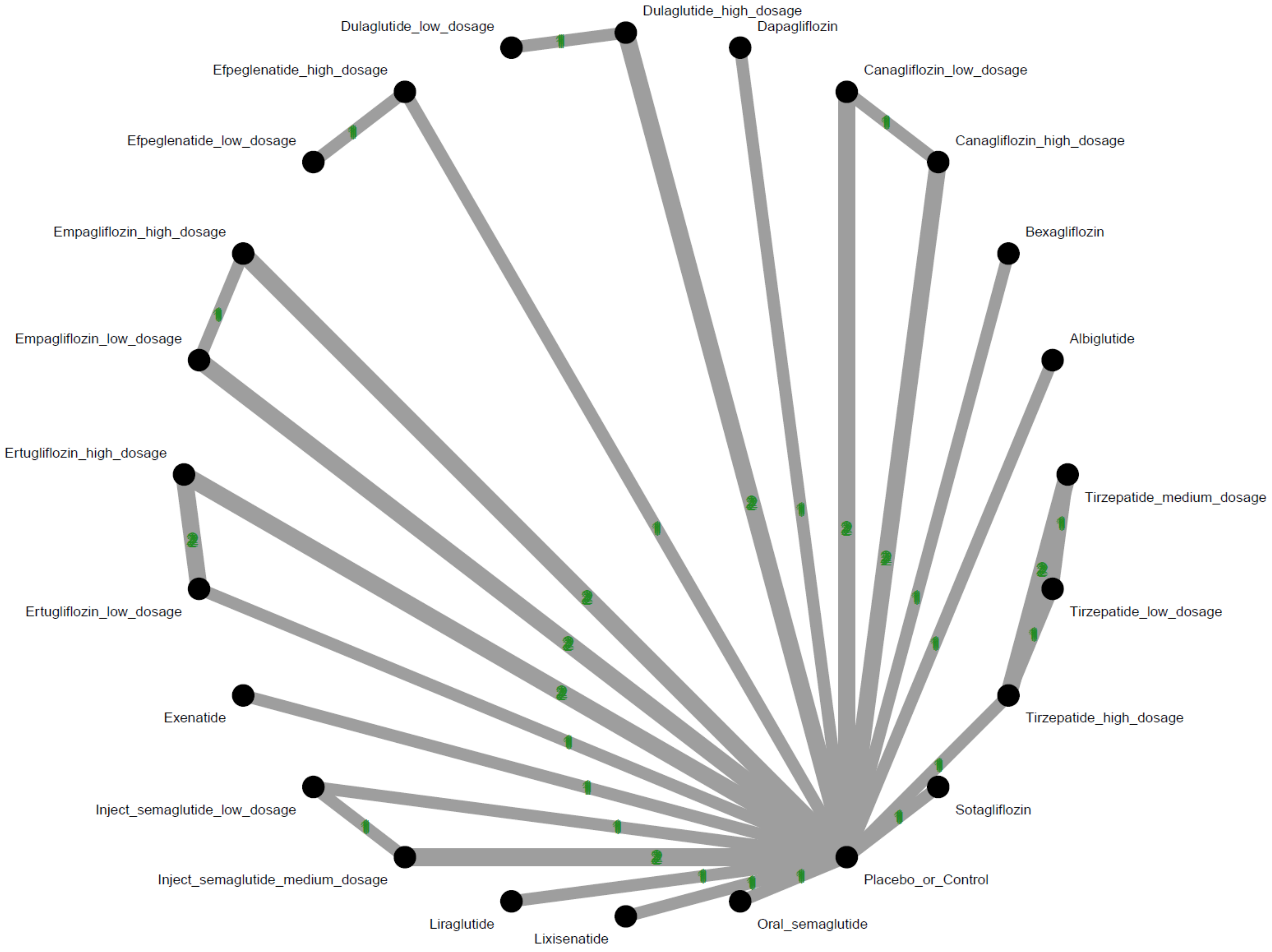

Dulaglutide_low_dosage
Dulaglutide_high_dosage
Dapagliflozin
Efpeglenatide_high_dosage
Canagliflozin_low_dosage
Efpeglenatide_low_dosage
Canagliflozin_high_dosage
Empagliflozin_high_dosage
Bexagliflozin
Empagliflozin_low_dosage
Albiglutide
Ertugliflozin_high_dosage
Tirzepatide_medium_dosage
Ertugliflozin_low_dosage
Tirzepatide_low_dosage
Exenatide
Tirzepatide_high_dosage
Inject_semaglutide_low_dosage
Sotagliflozin
Inject_semaglutide_medium_dosage
Placebo_or_Control
Liraglutide
Lixisenatide
Oral_semaglutide

Figure S1C network structure of NMA of drop-out rate

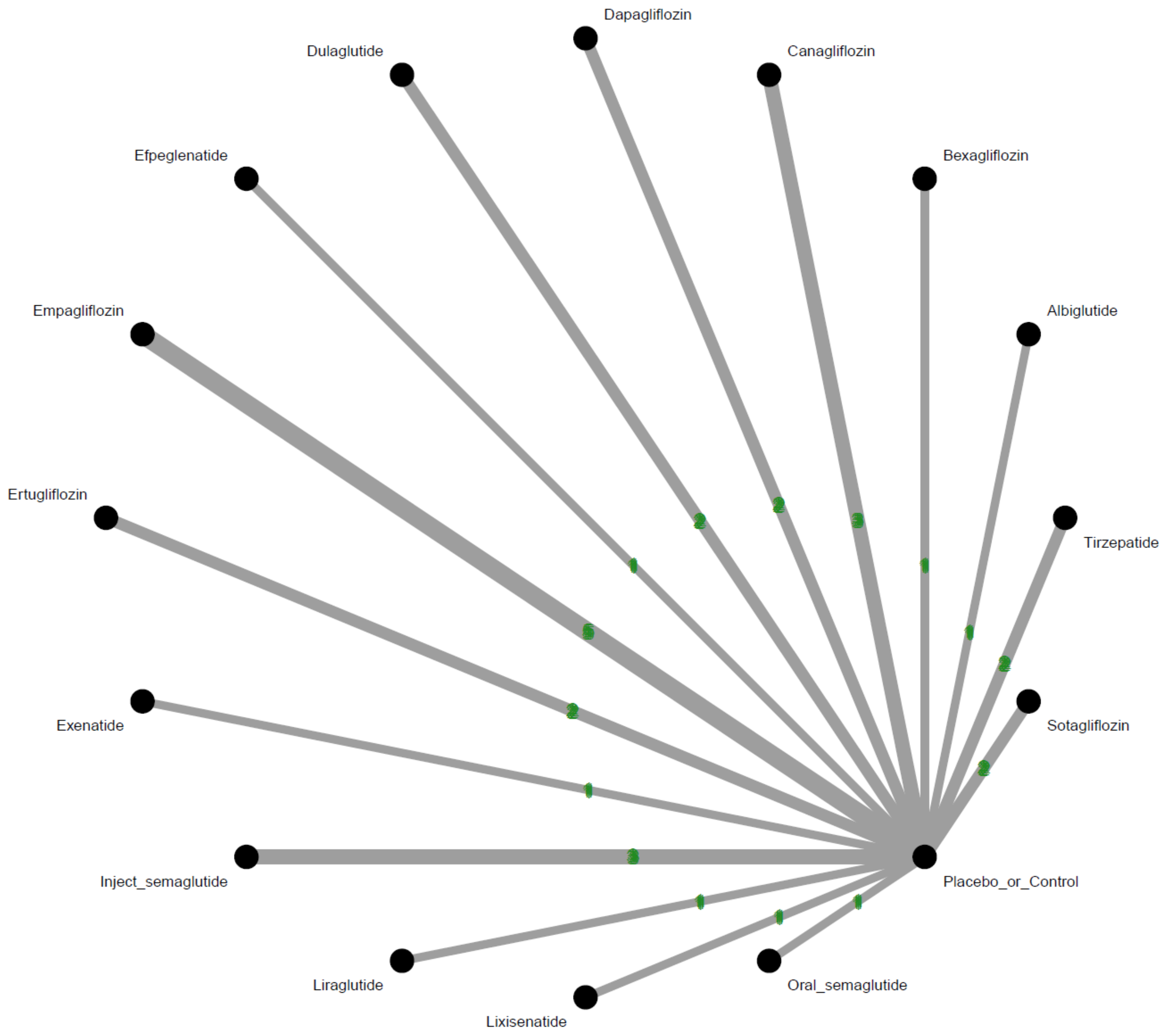

Figure legend of Figure S1A-S1C

The structure of the network meta-analysis. The lines between nodes represent direct comparisons from various trials, with the numbers over the lines indicating the number of trials providing these comparisons for each specific treatment. The thickness of the lines corresponds to the number of trials linked to the network.

Abbreviation for Figure S1A-S1C:

95%CIs: 95% confidence intervals; GLP-1 agonist: glucagon-like peptide-1 agonist; NMA: network meta-analysis; OR: odds ratio; RCT: randomized controlled trial; SGLT2 inhibitor: sodium–glucose cotransporter 2 inhibitor

Figure S2A forest plot of primary outcome: incidence of hearing loss (risk ratio)

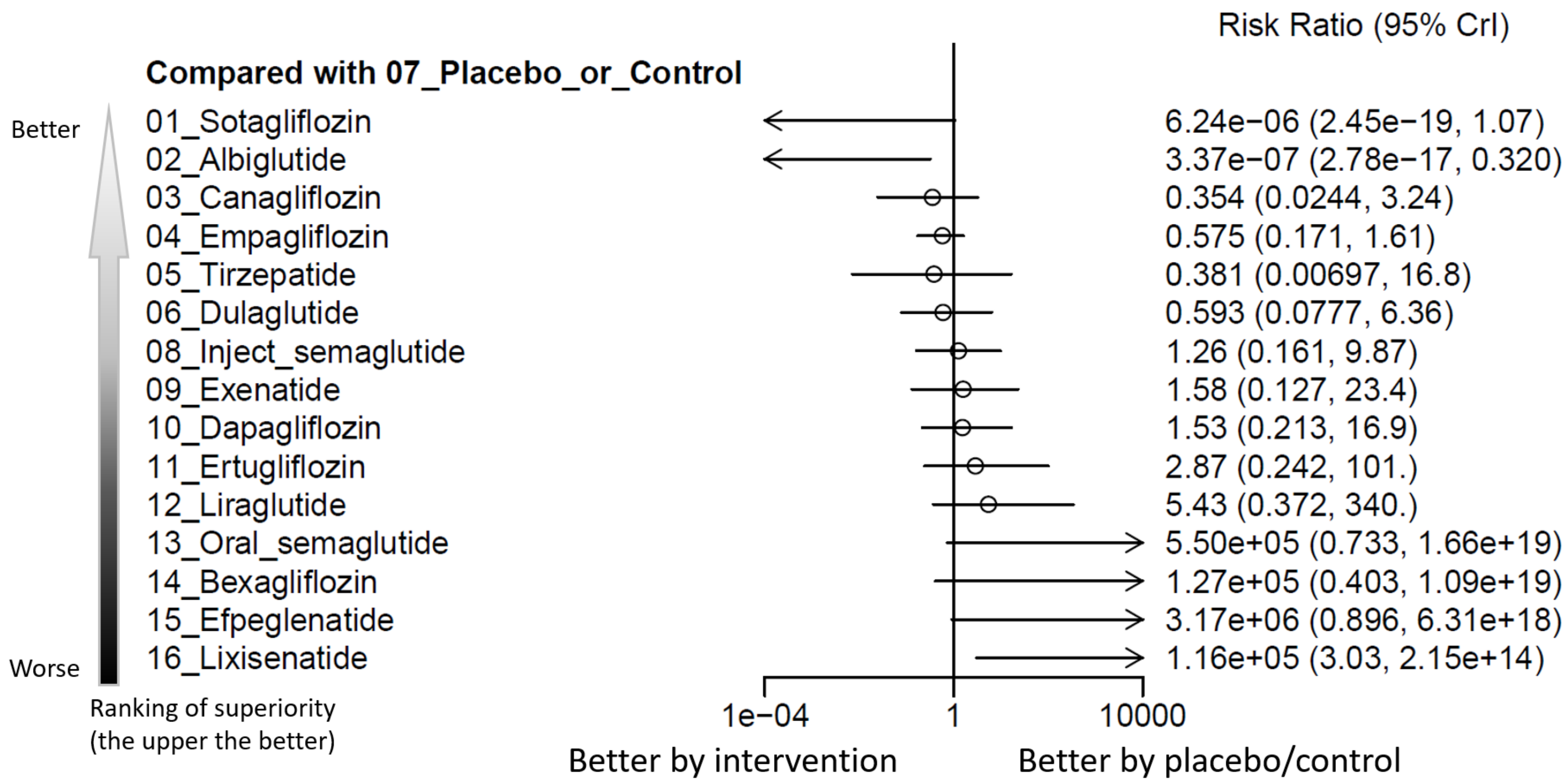

Figure S2B forest plot of primary outcome: incidence of hearing loss in subgroup of dosage (risk ratio)

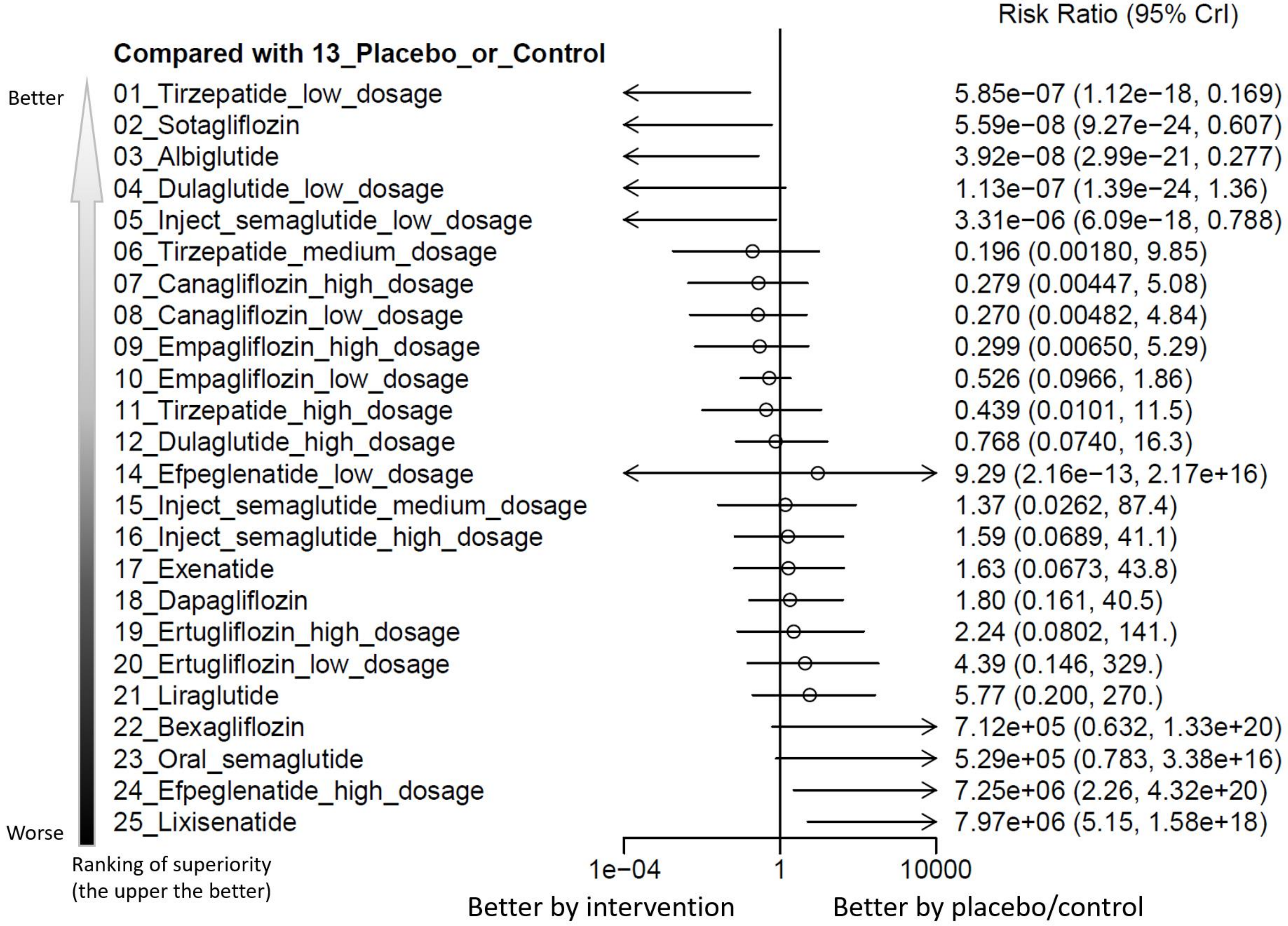

Figure S2C forest plot of primary outcome: incidence of hearing loss (focus on diabetes)

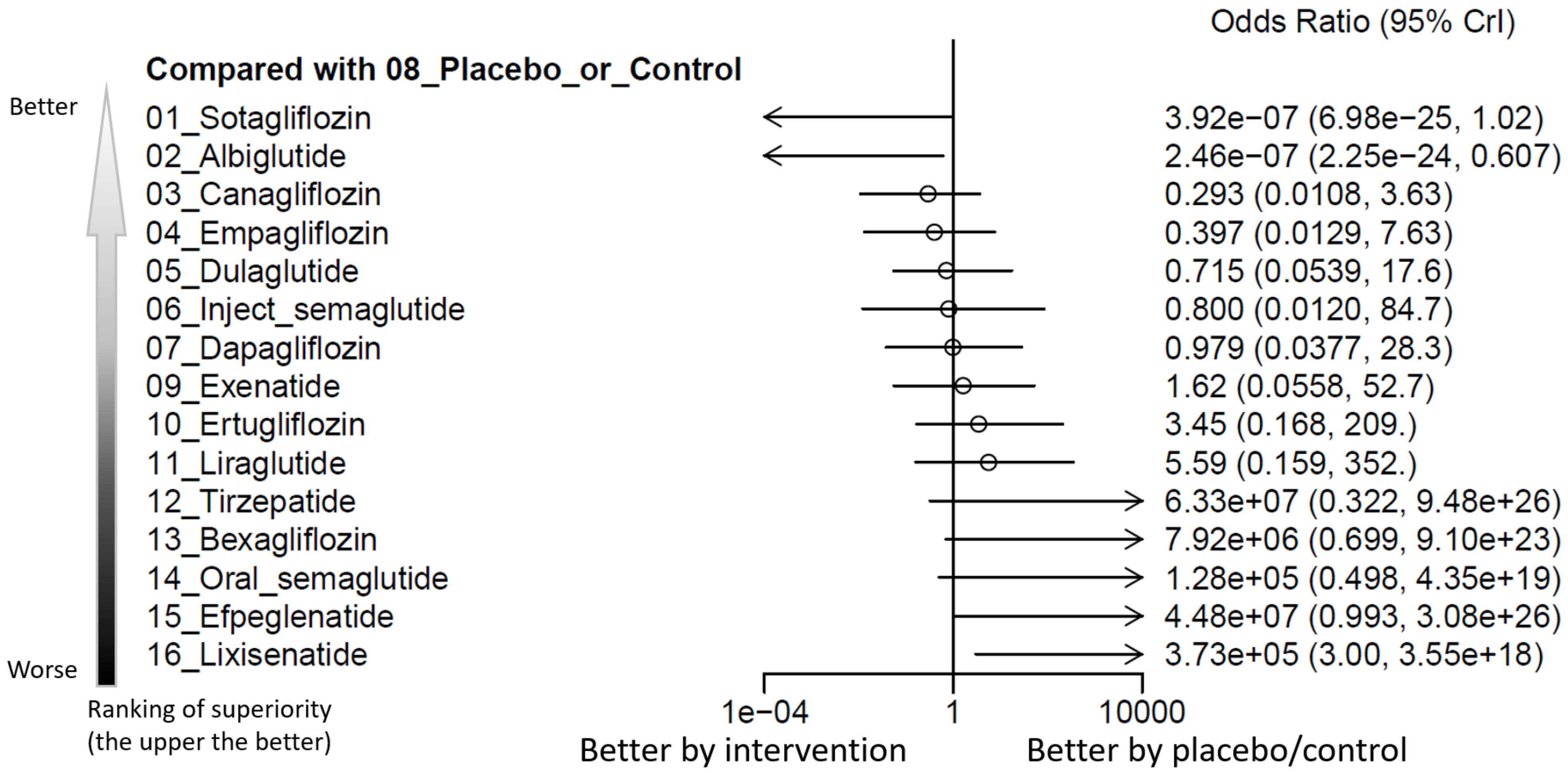

Figure S2D forest plot of primary outcome: incidence of hearing loss in subgroup of dosage (focus on diabetes)

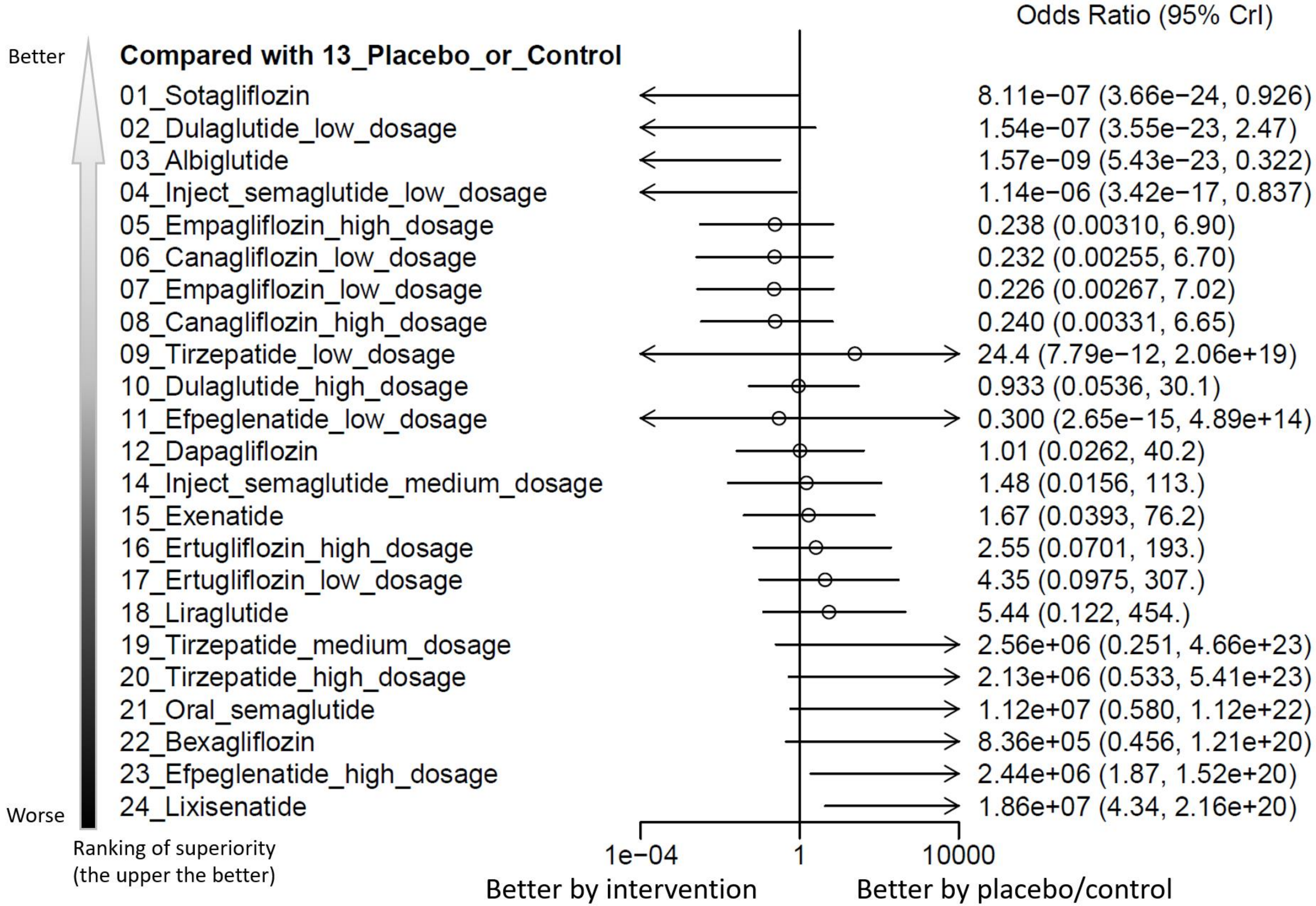

Figure S2E forest plot of NMA of drop-out rate

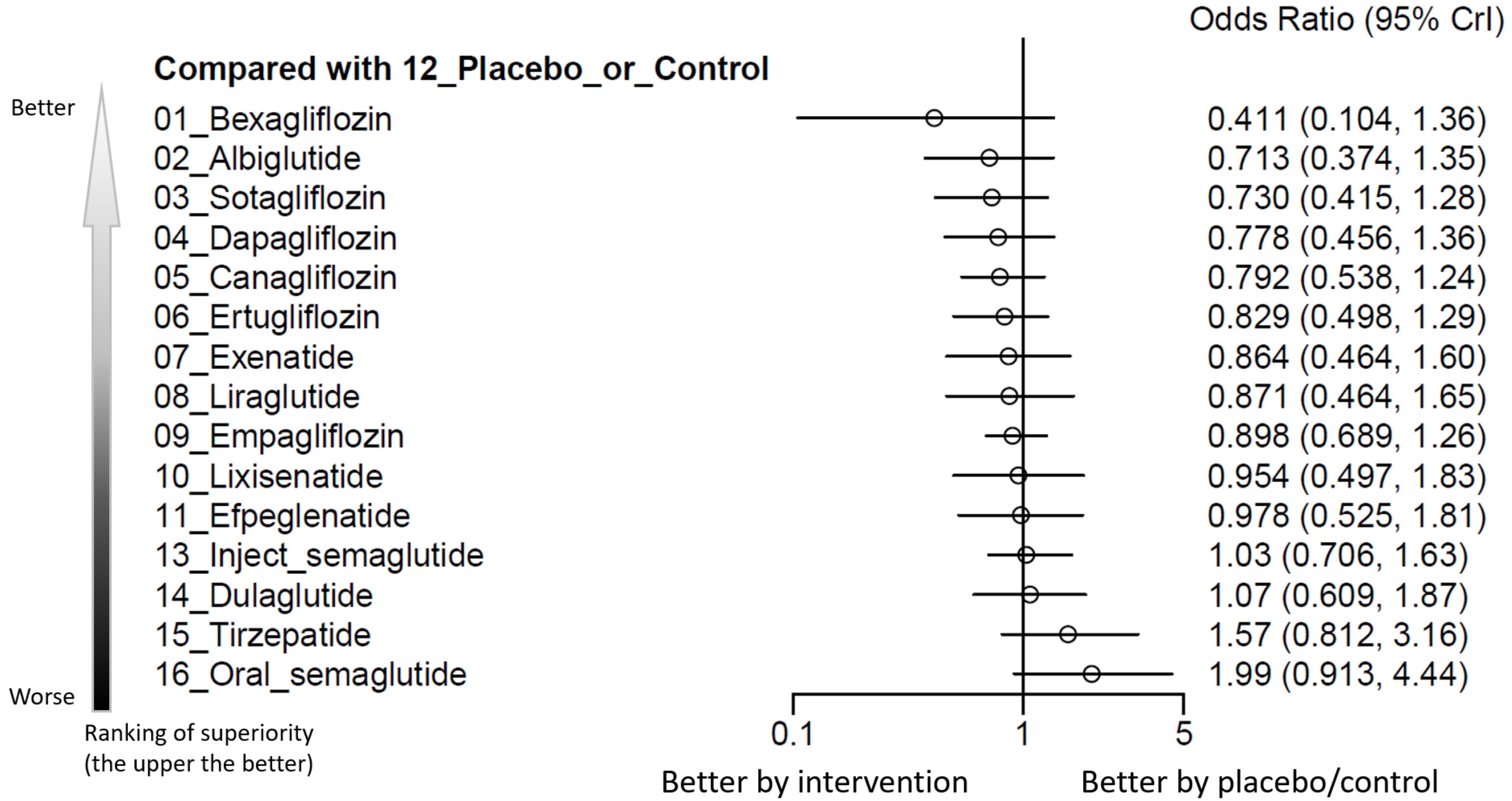

Abbreviation for Figure S2A-S2E:

95%CIs: 95% confidence intervals; GLP-1 agonist: glucagon-like peptide-1 agonist; NMA: network meta-analysis; OR: odds ratio; RCT: randomized controlled trial; SGLT2 inhibitor: sodium–glucose cotransporter 2 inhibitor

Figure S3A Individual study result of primary outcome: incidence of hearing loss

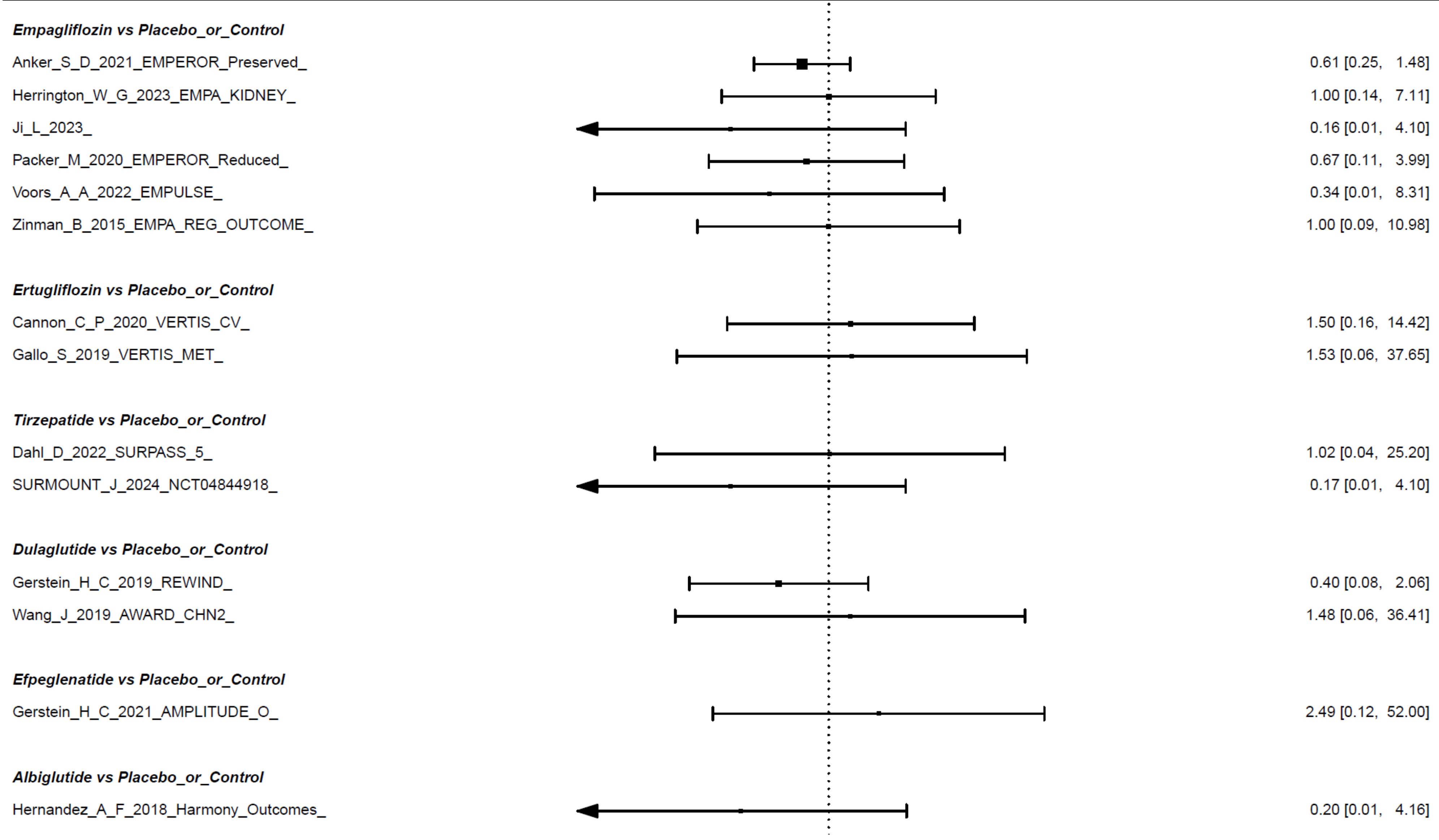

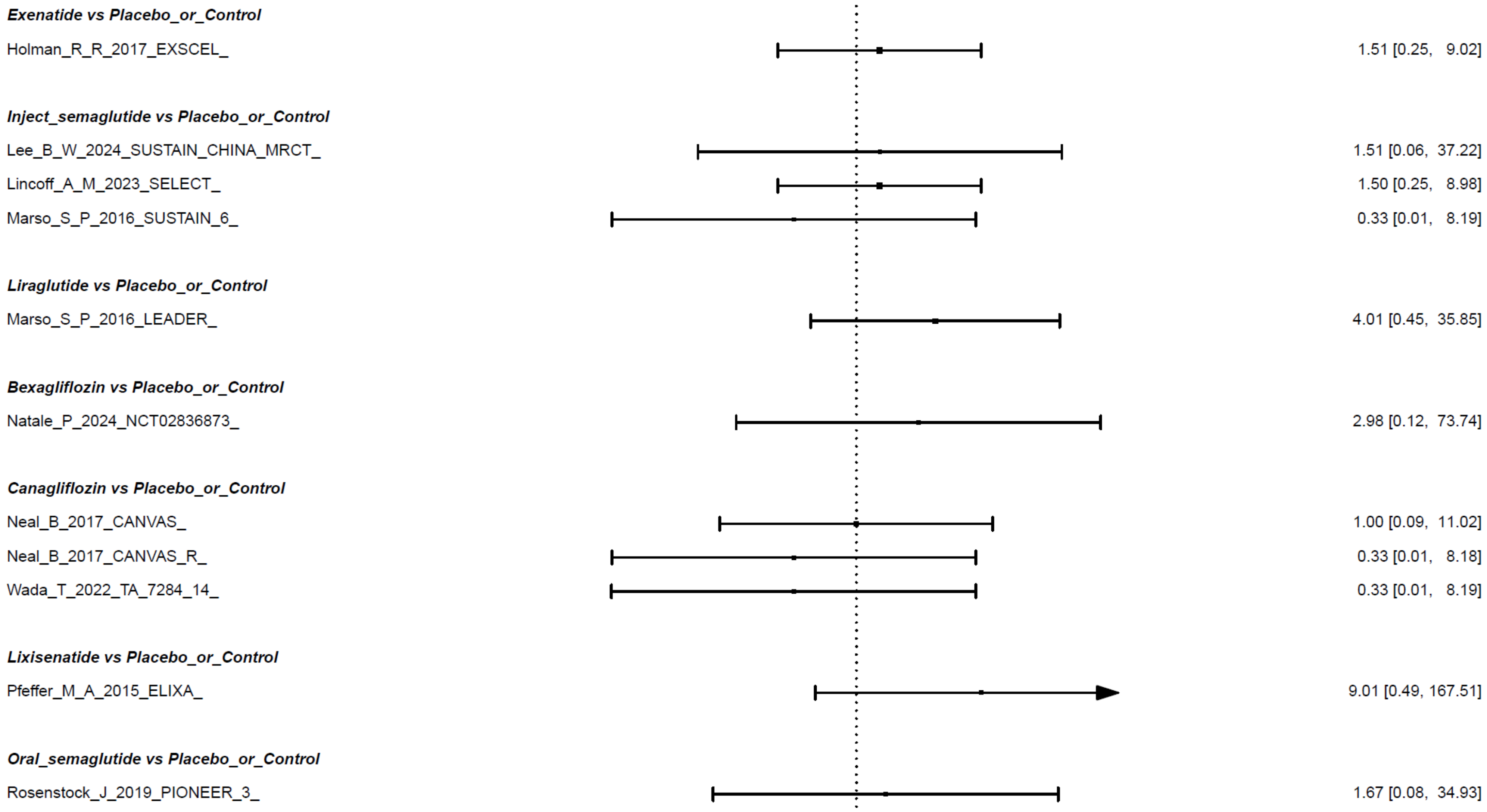
Exenatide vs Placebo_or_Control
Holman_R_R_2017_EXSCEL_
1.51 [0.25, 9.02]
Inject_semaglutide vs Placebo_or_Control
Lee_B_W_2024_SUSTAIN_CHINA_MRCT_
1.51 [0.06, 37.22]
Lincoff_A_M_2023_SELECT_
1.50 [0.25, 8.98]
Marso_S_P_2016_SUSTAIN_6_
0.33 [0.01, 8.19]
Liraglutide vs Placebo_or_Control
Marso_S_P_2016_LEADER_
4.01 [0.45, 35.85]
Bexagliflozin vs Placebo_or_Control
Natale_P_2024_NCT02836873_
2.98 [0.12, 73.74]
Canagliflozin vs Placebo_or_Control
Neal_B_2017_CANVAS_
1.00 [0.09, 11.02]
Neal_B_2017_CANVAS_R_
0.33 [0.01, 8.18]
Wada_T_2022_TA_7284_14_
0.33 [0.01, 8.19]
Lixisenatide vs Placebo_or_Control
Pfeffer_M_A_2015_ELIXA_
9.01 [0.49, 167.51]
Oral_semaglutide vs Placebo_or_Control
Rosenstock_J_2019_PIONEER_3_
1.67 [0.08, 34.93]

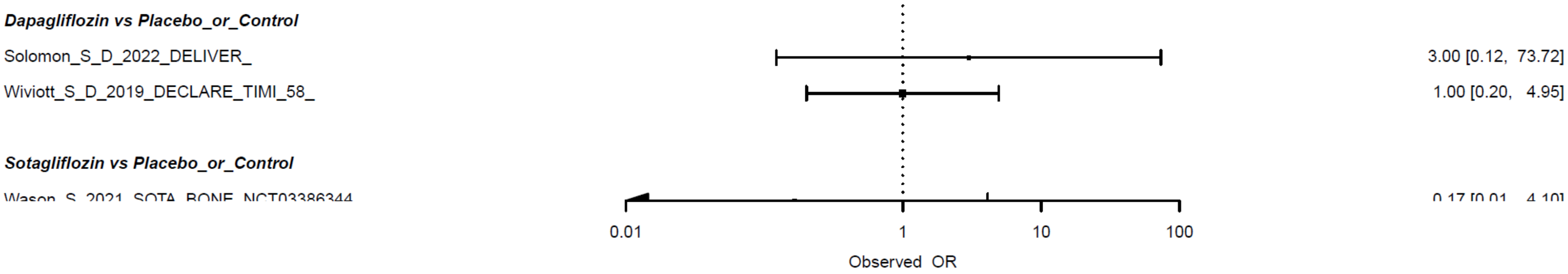
Dapagliflozin vs Placebo_or_Control
Solomon_S_D_2022_DELIVER_
3.00 [0.12, 73.72]
Wiviott_S_D_2019_DECLARE_TIMI_58_
1.00 [0.20, 4.95]
Sotagliflozin vs Placebo_or_Control
Wason_S_2021_SOTA_BONE_NCT03386344
0.17 [0.01, 4.10]
0.01
1
10
100
Observed OR

Figure S3B Individual study result of primary outcome: incidence of hearing loss in subgroup of dosage

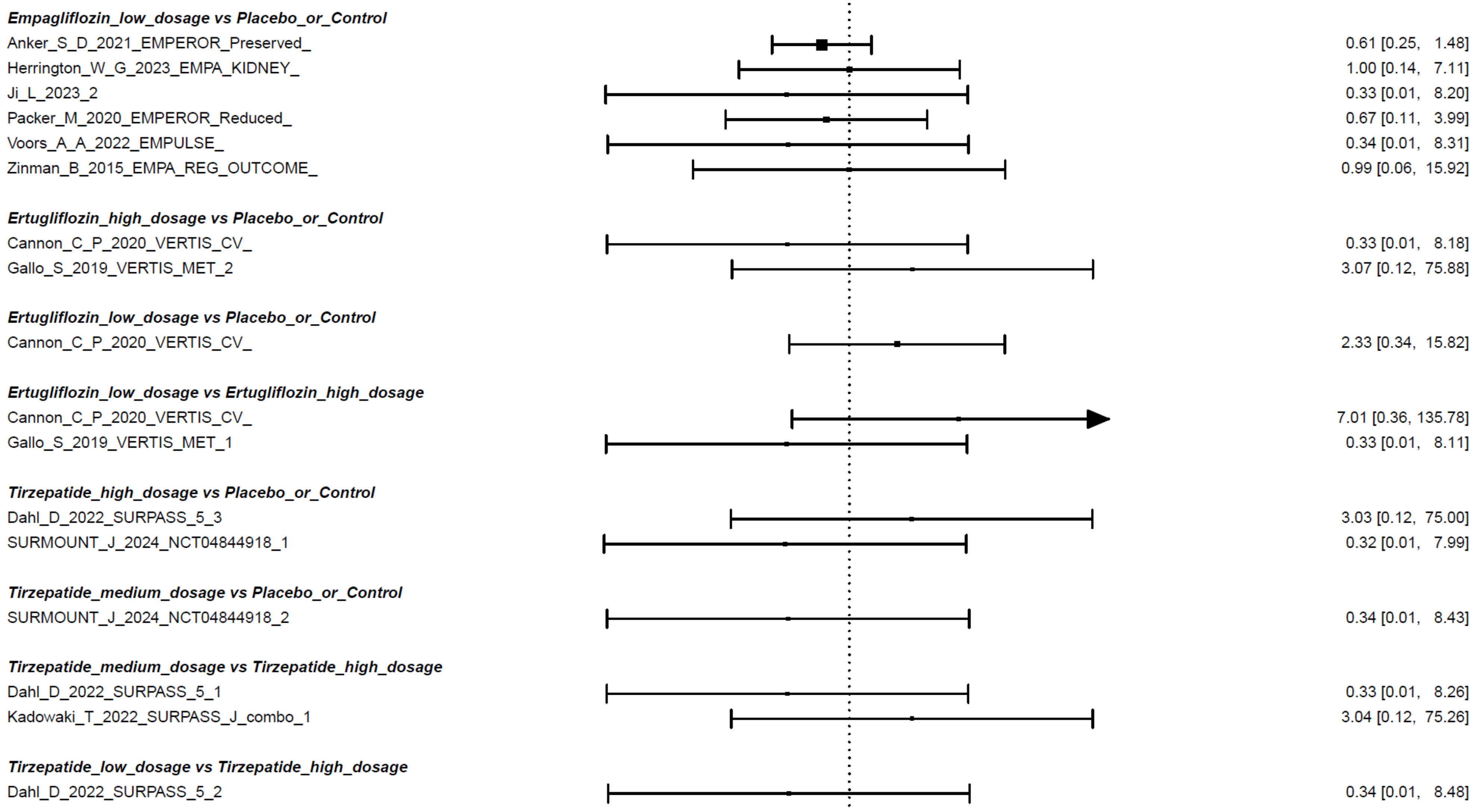

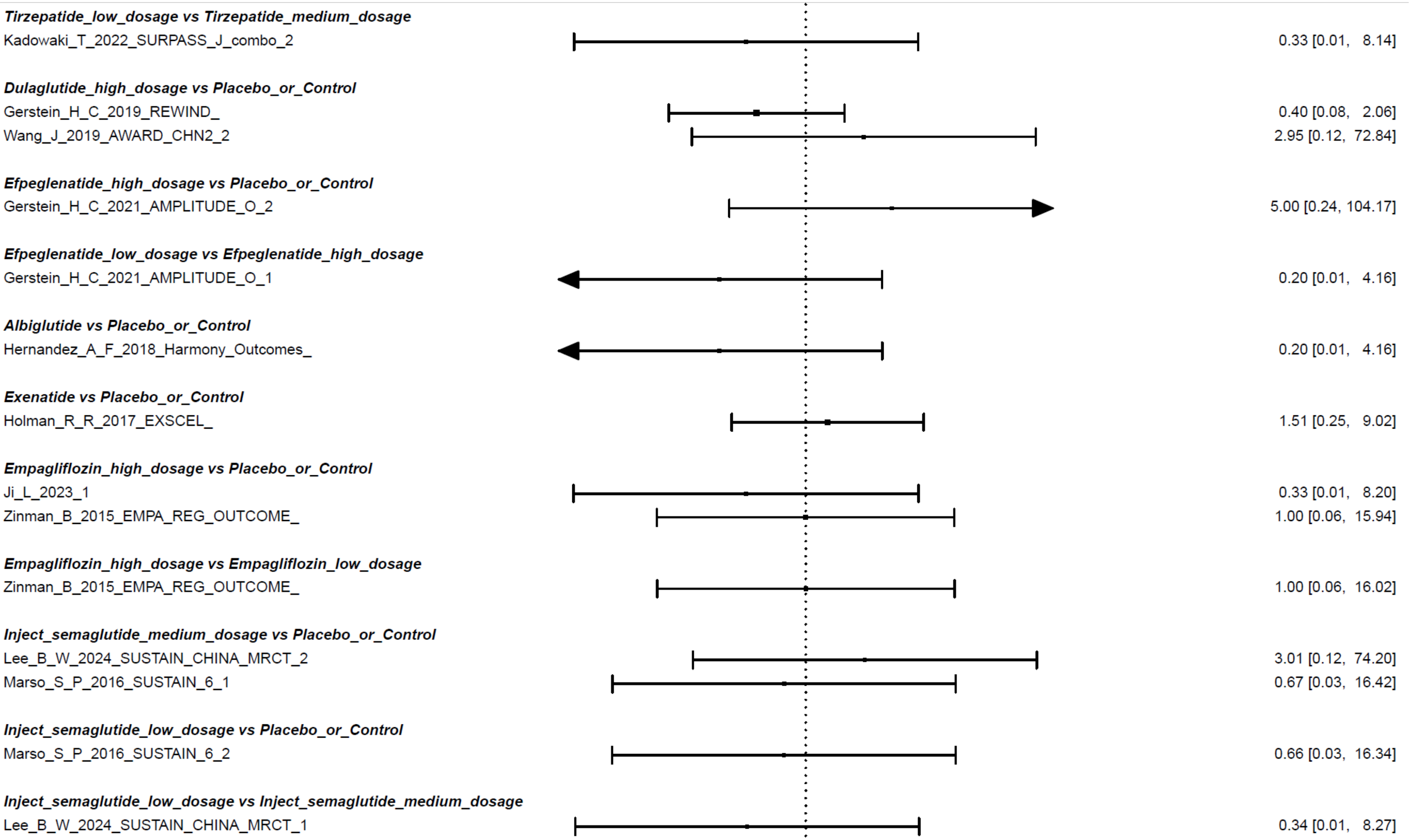

Tirzepatide_low_dosage vs Tirzepatide_medium_dosage
Kadowaki_T_2022_SURPASS_J_combo_2
0.33 [0.01, 8.14]
Dulaglutide_high_dosage vs Placebo_or_Control
Gerstein_H_C_2019_REWIND_
0.40 [0.08, 2.06]
Wang_J_2019_AWARD_CHN2_2
2.95 [0.12, 72.84]
Efpeglenatide_high_dosage vs Placebo_or_Control
Gerstein_H_C_2021_AMPLITUDE_O_2
5.00 [0.24, 104.17]
Efpeglenatide_low_dosage vs Efpeglenatide_high_dosage
Gerstein_H_C_2021_AMPLITUDE_O_1
0.20 [0.01, 4.16]
Albiglutide vs Placebo_or_Control
Hernandez_A_F_2018_Harmony_Outcomes_
0.20 [0.01, 4.16]
Exenatide vs Placebo_or_Control
Holman_R_R_2017_EXSCEL_
1.51 [0.25, 9.02]
Empagliflozin_high_dosage vs Placebo_or_Control
Ji_L_2023_1
0.33 [0.01, 8.20]
Zinman_B_2015_EMPA_REG_OUTCOME_
1.00 [0.06, 15.94]
Empagliflozin_high_dosage vs Empagliflozin_low_dosage
Zinman_B_2015_EMPA_REG_OUTCOME_
1.00 [0.06, 16.02]
Inject_semaglutide_medium_dosage vs Placebo_or_Control
Lee_B_W_2024_SUSTAIN_CHINA_MRCT_2
3.01 [0.12, 74.20]
Marso_S_P_2016_SUSTAIN_6_1
0.67 [0.03, 16.42]
Inject_semaglutide_low_dosage vs Placebo_or_Control
Marso_S_P_2016_SUSTAIN_6_2
0.66 [0.03, 16.34]
Inject_semaglutide_low_dosage vs Inject_semaglutide_medium_dosage
Lee_B_W_2024_SUSTAIN_CHINA_MRCT_1
0.34 [0.01, 8.27]

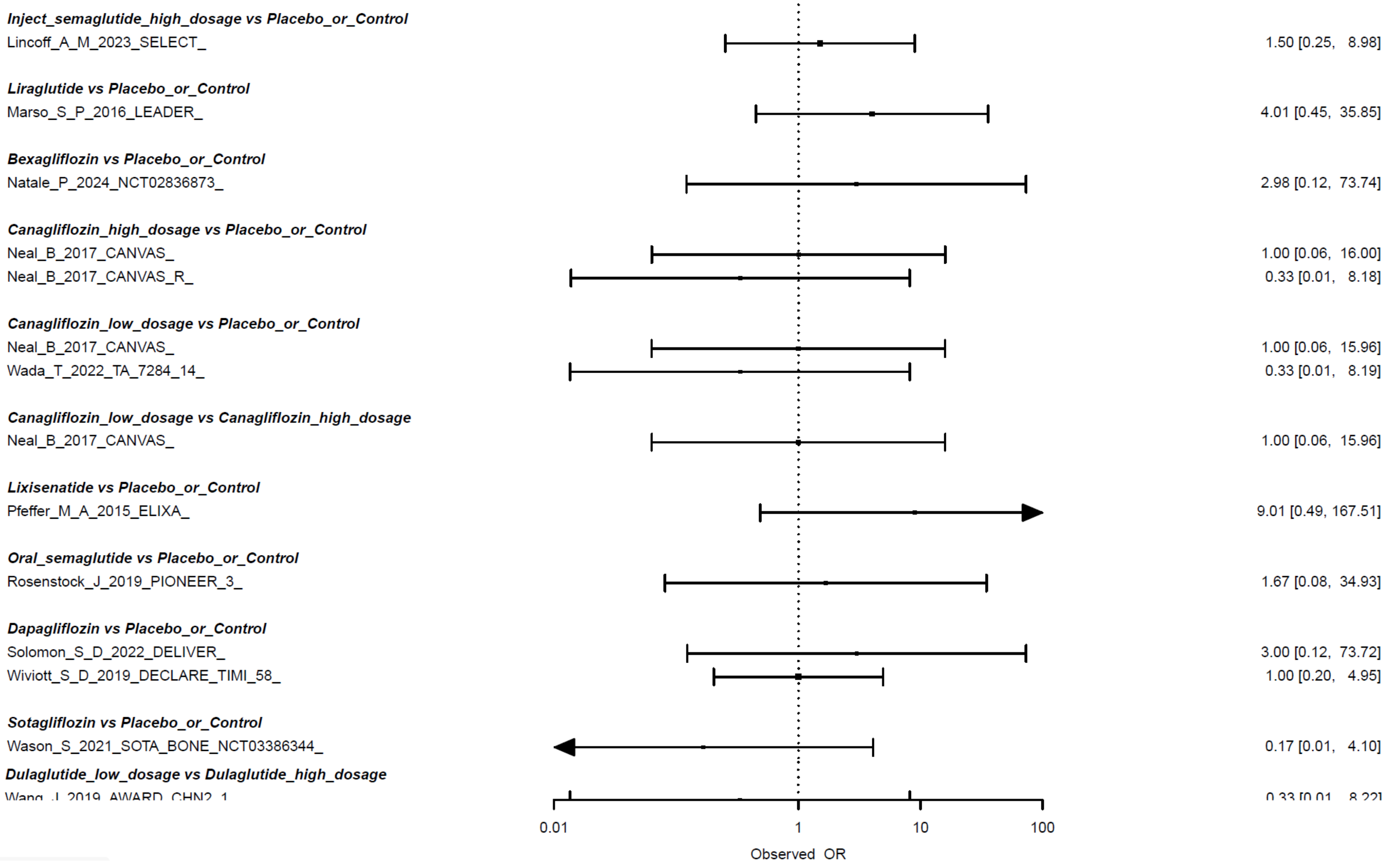
Inject_semaglutide_high_dosage vs Placebo_or_Control
Lincoff_A_M_2023_SELECT_
1.50 [0.25, 8.98]
Liraglutide vs Placebo_or_Control
Marso_S_P_2016_LEADER_
4.01 [0.45, 35.85]
Bexagliflozin vs Placebo_or_Control
Natale_P_2024_NCT02836873_
2.98 [0.12, 73.74]
Canagliflozin_high_dosage vs Placebo_or_Control
Neal_B_2017_CANVAS_
1.00 [0.06, 16.00]
Neal_B_2017_CANVAS_R_
0.33 [0.01, 8.18]
Canagliflozin_low_dosage vs Placebo_or_Control
Neal_B_2017_CANVAS_
1.00 [0.06, 15.96]
Wada_T_2022_TA_7284_14_
0.33 [0.01, 8.19]
Canagliflozin_low_dosage vs Canagliflozin_high_dosage
Neal_B_2017_CANVAS_
1.00 [0.06, 15.96]
Lixisenatide vs Placebo_or_Control
Pfeffer_M_A_2015_ELIXA_
9.01 [0.49, 167.51]
Oral_semaglutide vs Placebo_or_Control
Rosenstock_J_2019_PIONEER_3_
1.67 [0.08, 34.93]
Dapagliflozin vs Placebo_or_Control
Solomon_S_D_2022_DELIVER_
3.00 [0.12, 73.72]
Wiviott_S_D_2019_DECLARE_TIMI_58_
1.00 [0.20, 4.95]
Sotagliflozin vs Placebo_or_Control
Wason_S_2021_SOTA_BONE_NCT03386344_
0.17 [0.01, 4.10]
Dulaglutide_low_dosage vs Dulaglutide_high_dosage
0.01
1
10
100
Observed OR

Figure S3C Individual study result of drop-out rate

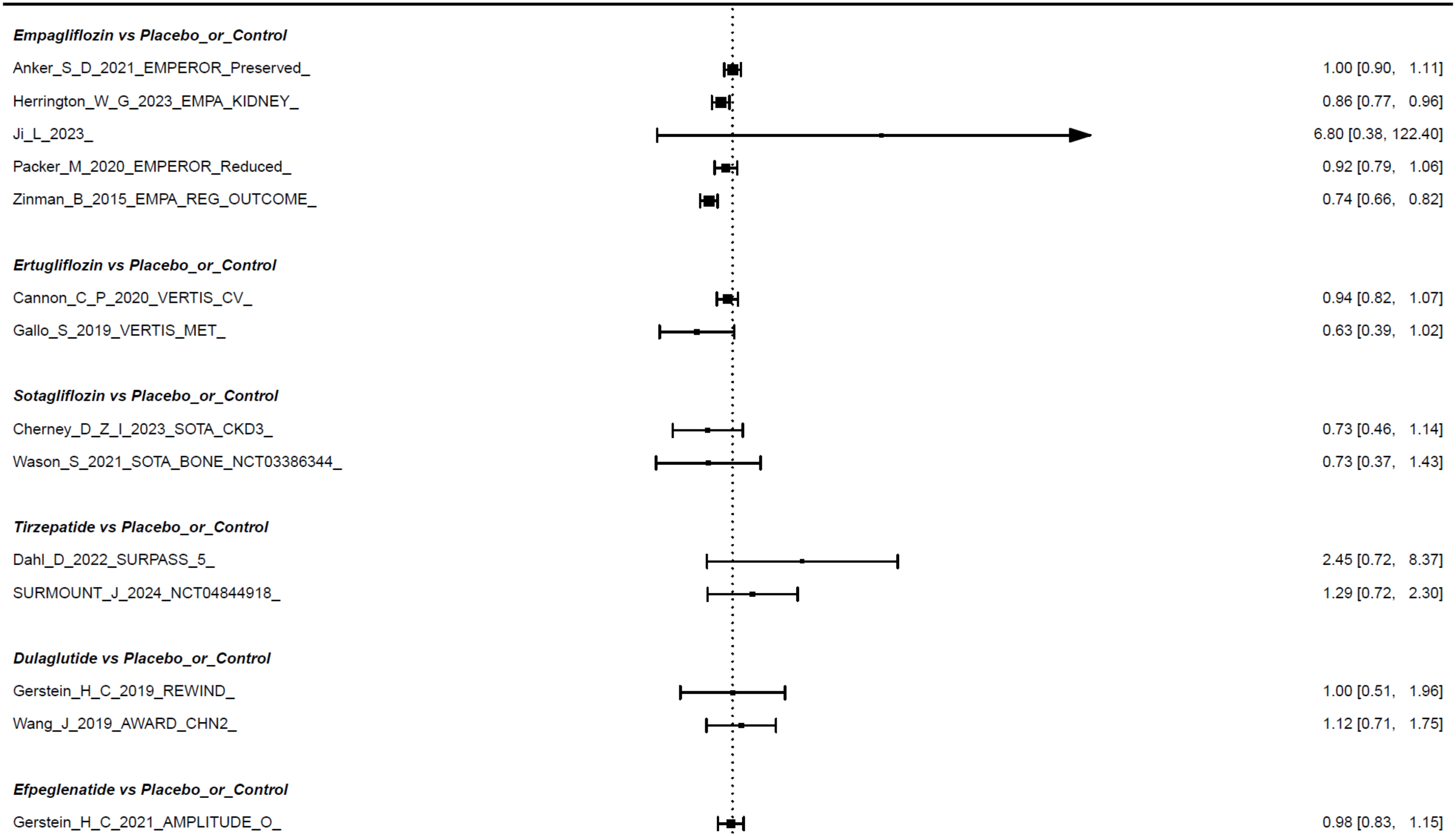

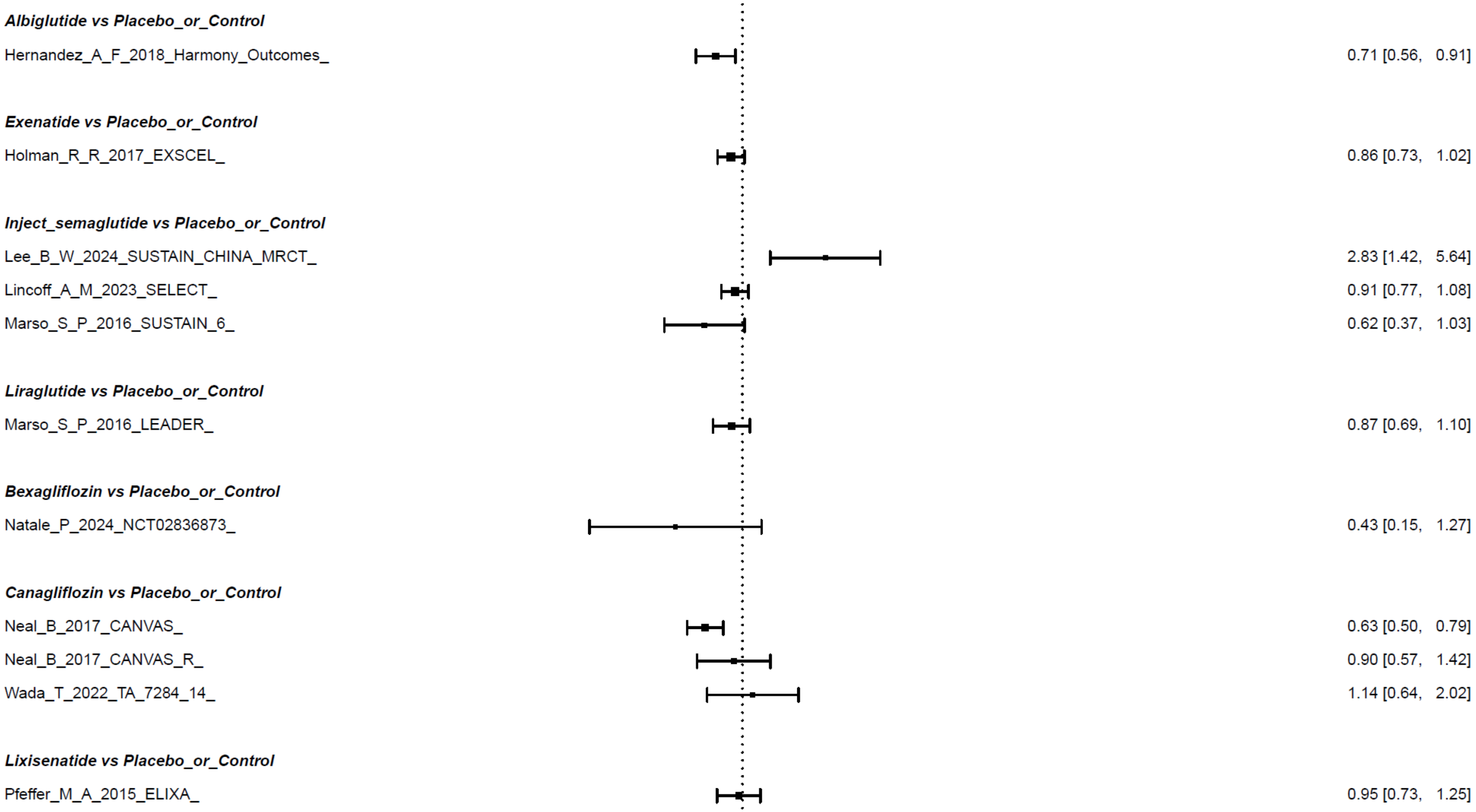

Albiglutide vs Placebo_or_Control
Hernandez_A_F_2018_Harmony_Outcomes_
0.71 [0.56, 0.91]
Exenatide vs Placebo_or_Control
Holman_R_R_2017_EXSCEL_
0.86 [0.73, 1.02]
Inject_semaglutide vs Placebo_or_Control
Lee_B_W_2024_SUSTAIN_CHINA_MRCT_
2.83 [1.42, 5.64]
Lincoff_A_M_2023_SELECT_
0.91 [0.77, 1.08]
Marso_S_P_2016_SUSTAIN_6_
0.62 [0.37, 1.03]
Liraglutide vs Placebo_or_Control
Marso_S_P_2016_LEADER_
0.87 [0.69, 1.10]
Bexagliflozin vs Placebo_or_Control
Natale_P_2024_NCT02836873_
0.43 [0.15, 1.27]
Canagliflozin vs Placebo_or_Control
Neal_B_2017_CANVAS_
0.63 [0.50, 0.79]
Neal_B_2017_CANVAS_R_
0.90 [0.57, 1.42]
Wada_T_2022_TA_7284_14_
1.14 [0.64, 2.02]
Lixisenatide vs Placebo_or_Control
Pfeffer_M_A_2015_ELIXA_
0.95 [0.73, 1.25]

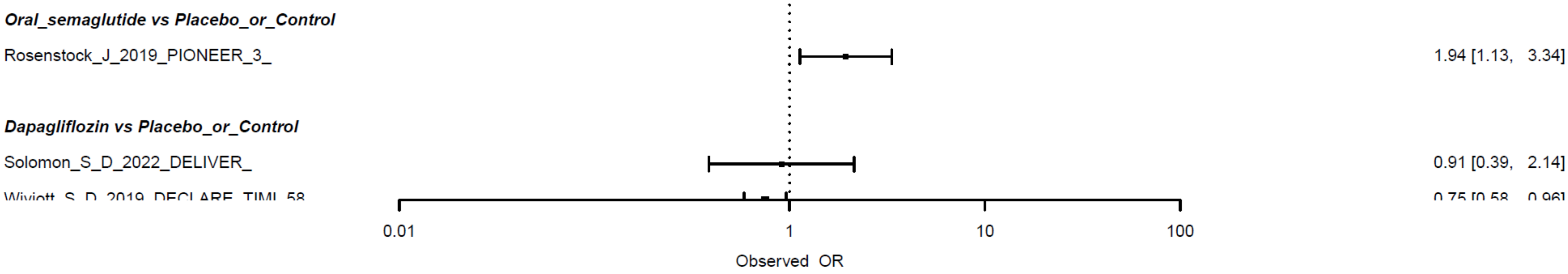
Oral_semaglutide vs Placebo_or_Control
Rosenstock_J_2019_PIONEER_3_
1.94 [1.13, 3.34]
Dapagliflozin vs Placebo_or_Control
Solomon_S_D_2022_DELIVER_
0.91 [0.39, 2.14]
0.01
1
10
100
Observed OR

Abbreviation for Figure S3A-S3C:

95%CIs: 95% confidence intervals; GLP-1 agonist: glucagon-like peptide-1 agonist; NMA: network meta-analysis; OR: odds ratio; RCT: randomized controlled trial; SGLT2 inhibitor: sodium–glucose cotransporter 2 inhibitor

Figure S4A Bayesian-based Litmus Rank-O-Gram rank plot of primary outcome: incidence of hearing loss

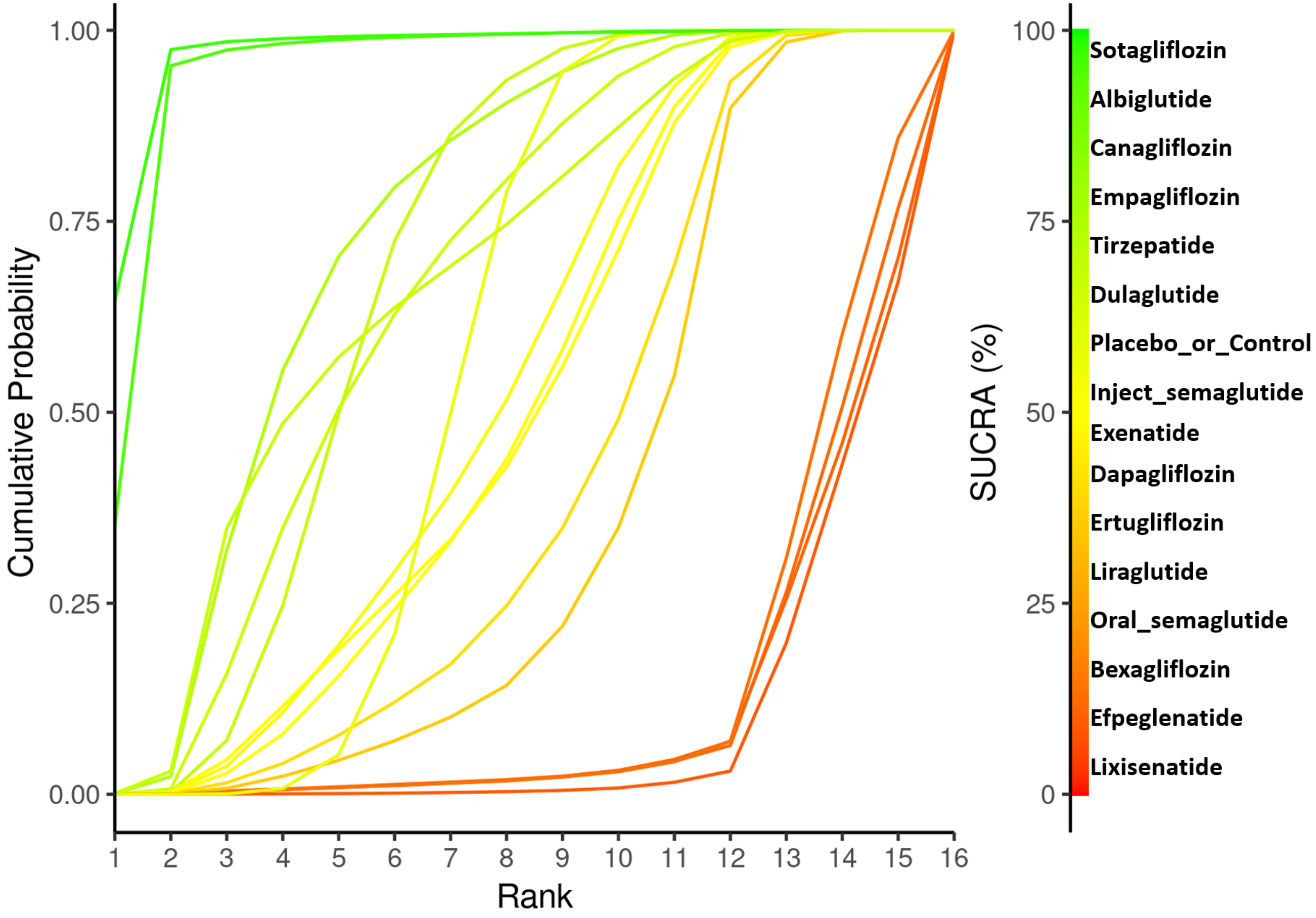

Figure S4B Bayesian-based radial surface under the cumulative ranking of primary outcome: incidence of hearing loss

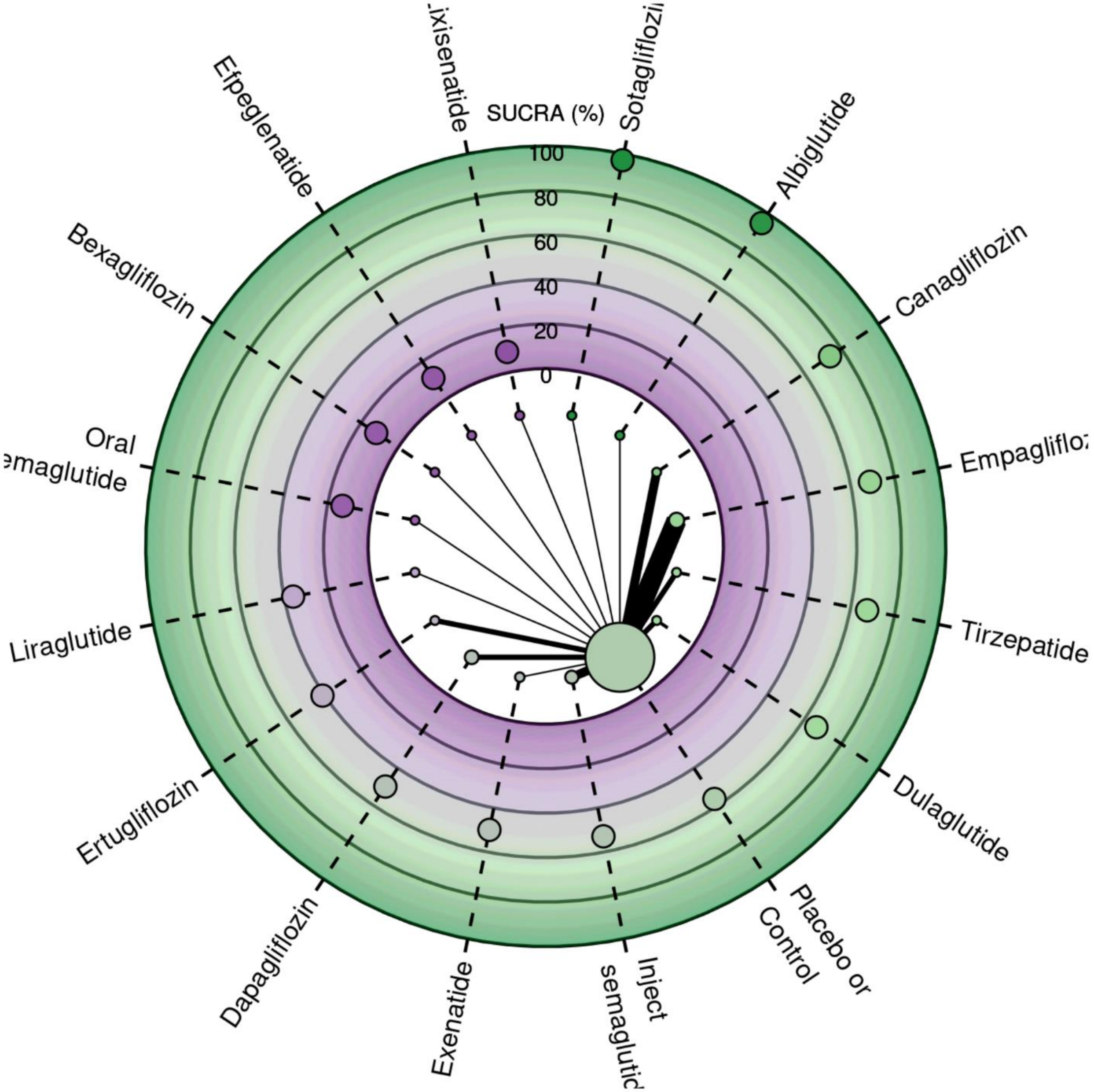

Figure S4C Bayesian-based Litmus Rank-O-Gram rank plot of primary outcome: incidence of hearing loss in subgroup of dosage

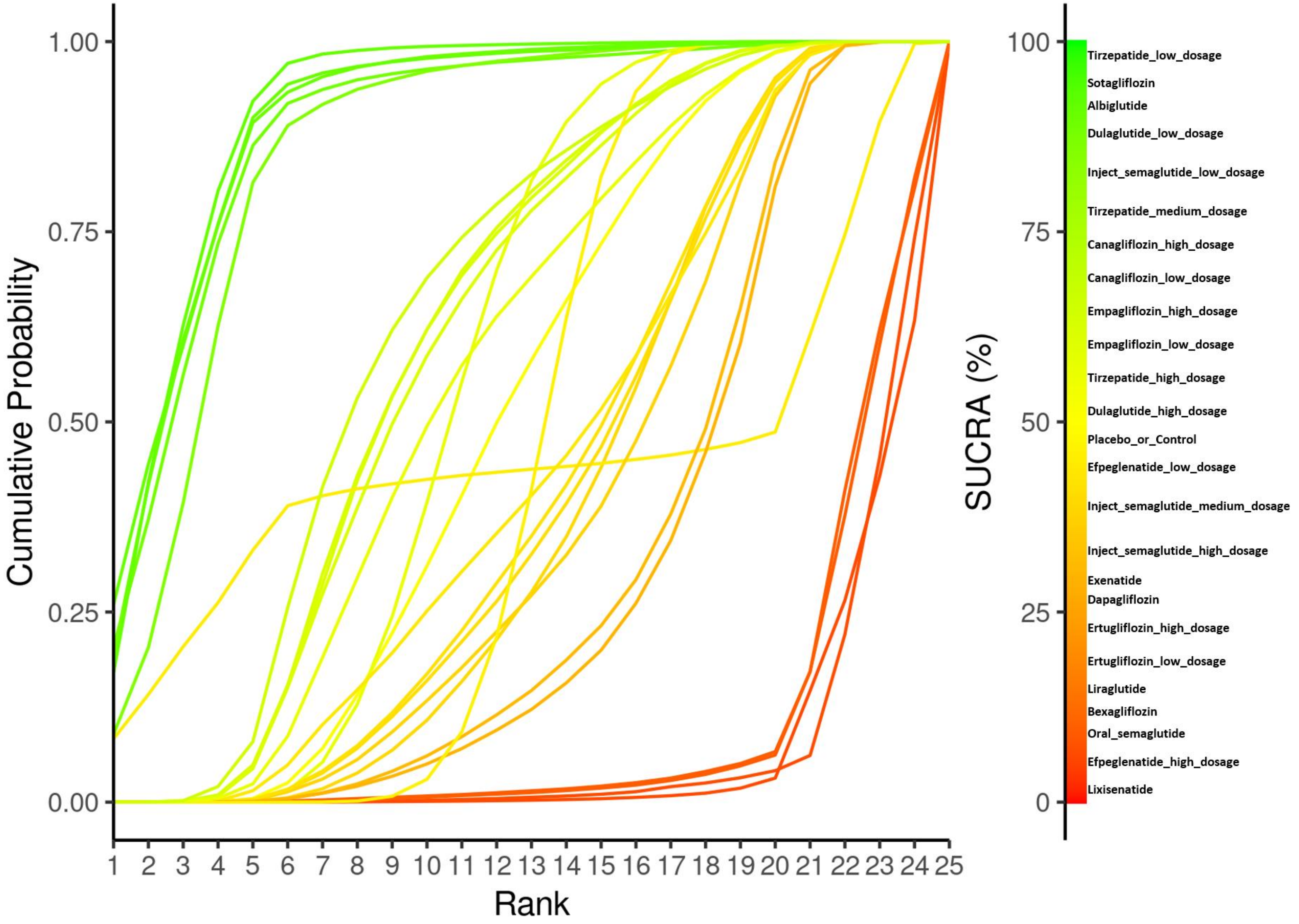

Cumulative Probability
1.00
0.75
0.50
0.25
0.00
1 2 3 4 5 6 7 8 9 10 11 12 13 14 15 16 17 18 19 20 21 22 23 24 25
Rank
SUCRA (%)
100
75
50
25
0
Tirzepatide_low_dosage
Sotagliflozin
Albiglutide
Dulaglutide_low_dosage
Inject_semaglutide_low_dosage
Tirzepatide_medium_dosage
Canagliflozin_high_dosage
Canagliflozin_low_dosage
Empagliflozin_high_dosage
Empagliflozin_low_dosage
Tirzepatide_high_dosage
Dulaglutide_high_dosage
Placebo_or_Control
Efpeglenatide_low_dosage
Inject_semaglutide_medium_dosage
Inject_semaglutide_high_dosage
Exenatide
Dapagliflozin
Ertugliflozin_high_dosage
Ertugliflozin_low_dosage
Liraglutide
Bexagliflozin
Oral_semaglutide
Efpeglenatide_high_dosage
Lixisenatide

Figure S4D Bayesian-based radial surface under the cumulative ranking of primary outcome: incidence of hearing loss in subgroup of dosage

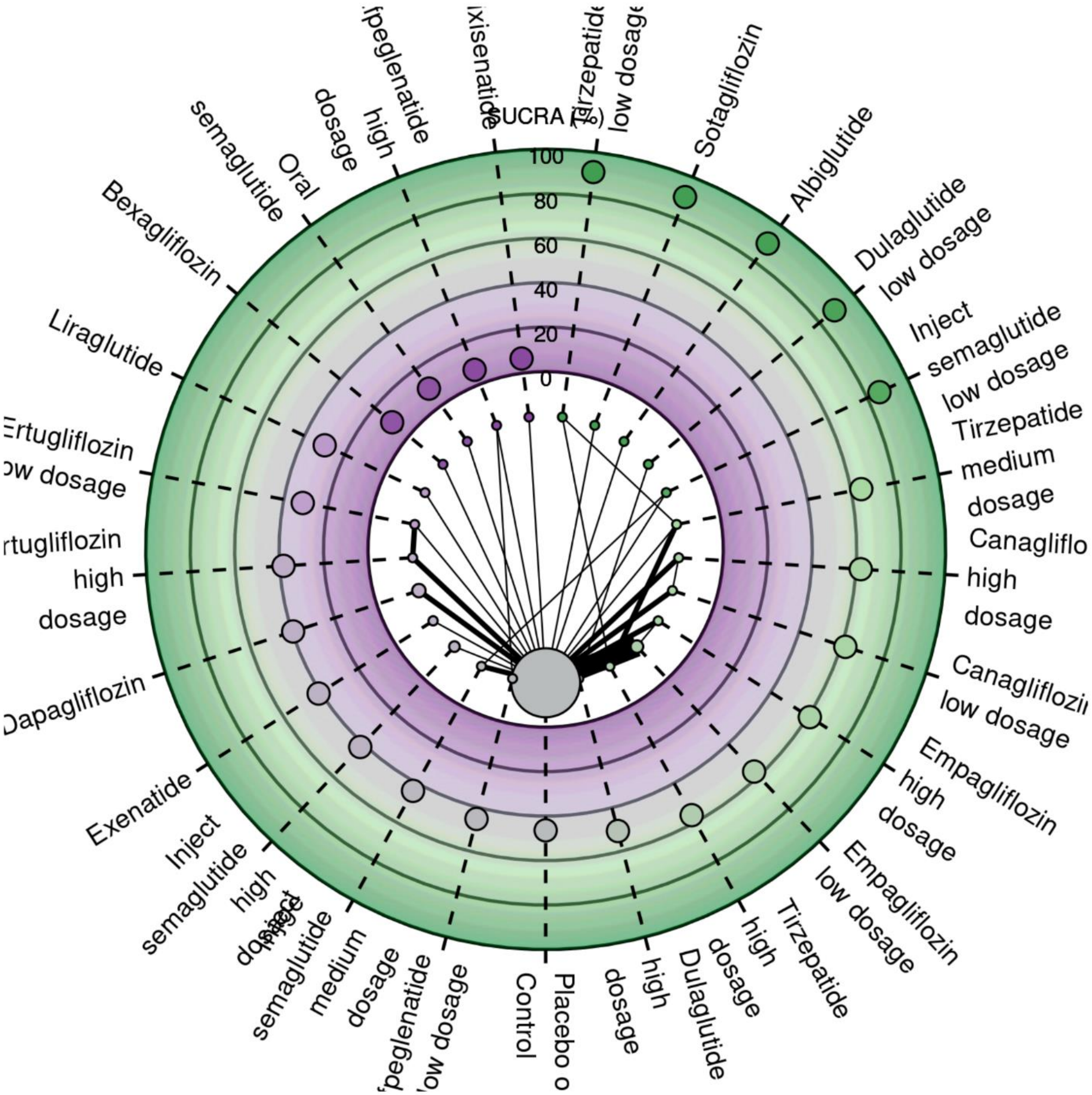

Figure S4E Bayesian-based Litmus Rank-O-Gram rank plot of drop-out rate

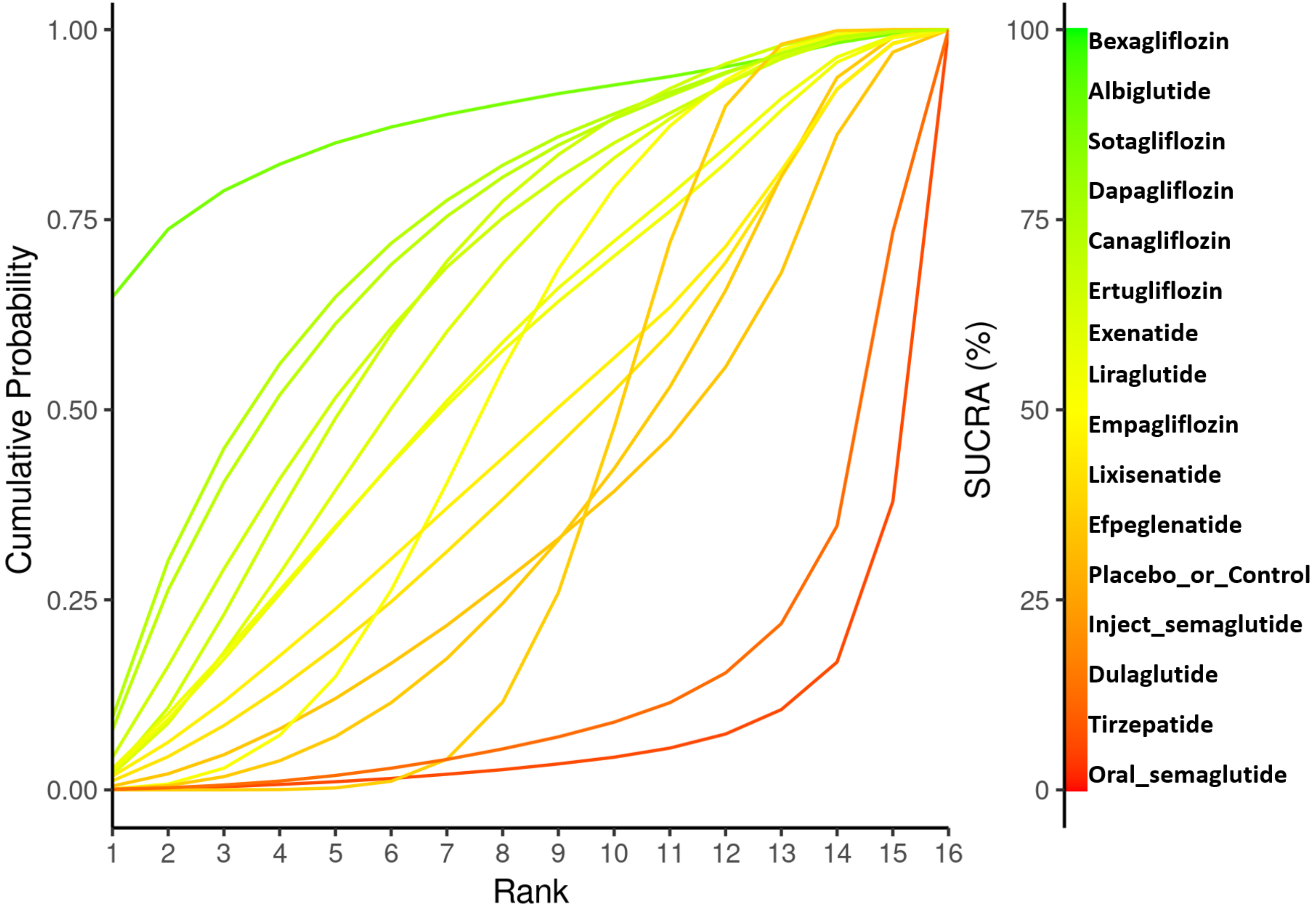

Figure S4F Bayesian-based radial surface under the cumulative ranking of drop-out rate

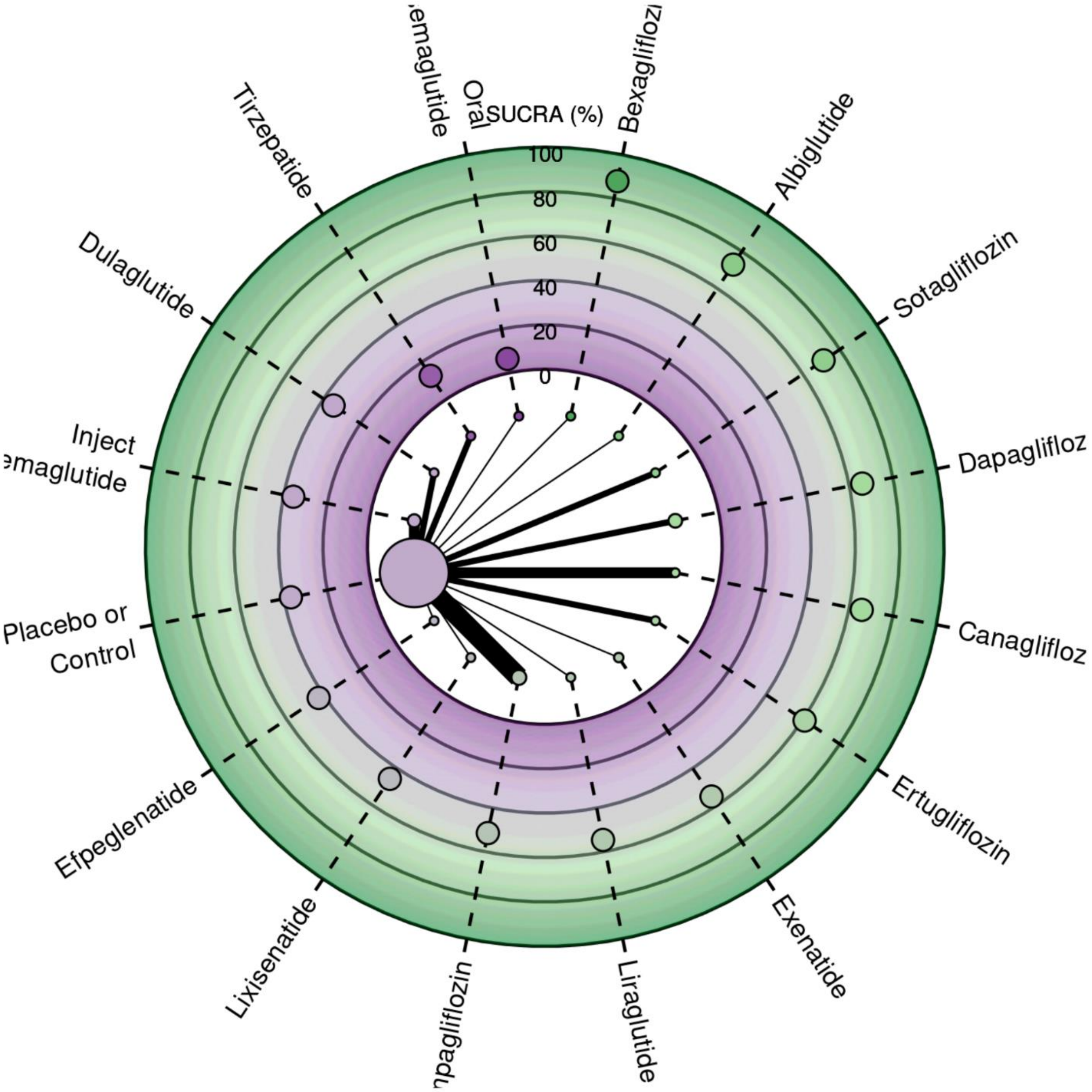

Abbreviation for Figure S4A-S4F:

95%CIs: 95% confidence intervals; GLP-1 agonist: glucagon-like peptide-1 agonist; NMA: network meta-analysis; OR: odds ratio; RCT: randomized controlled trial; SGLT2 inhibitor: sodium–glucose cotransporter 2 inhibitor

Figure S5A Bayesian-based residual deviance NMA/UME model of primary outcome: incidence of hearing loss

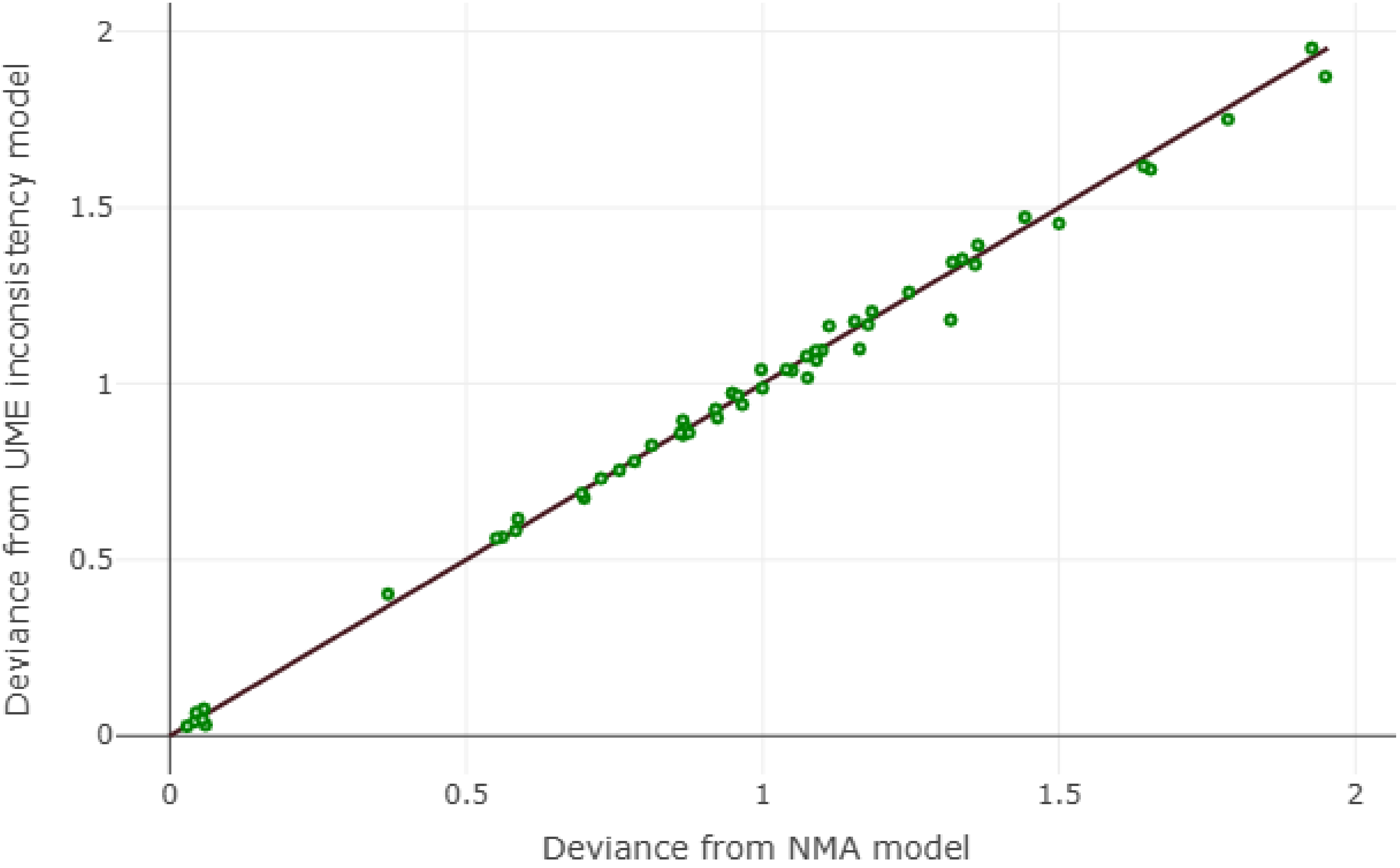

Figure S5B Bayesian-based per-arm residual deviance of primary outcome: incidence of hearing loss

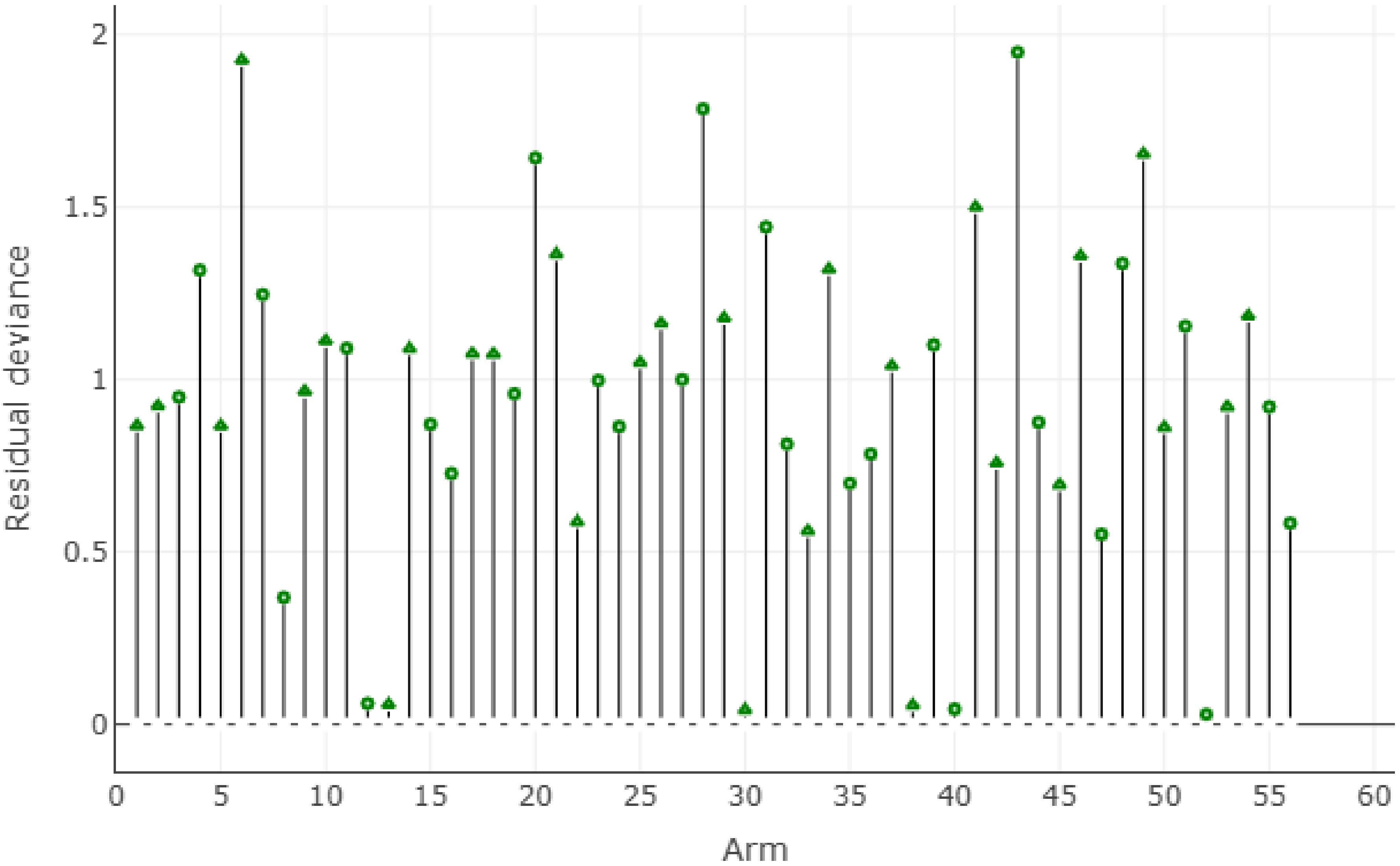

Figure S5C Bayesian-based leverage plot of primary outcome: incidence of hearing loss

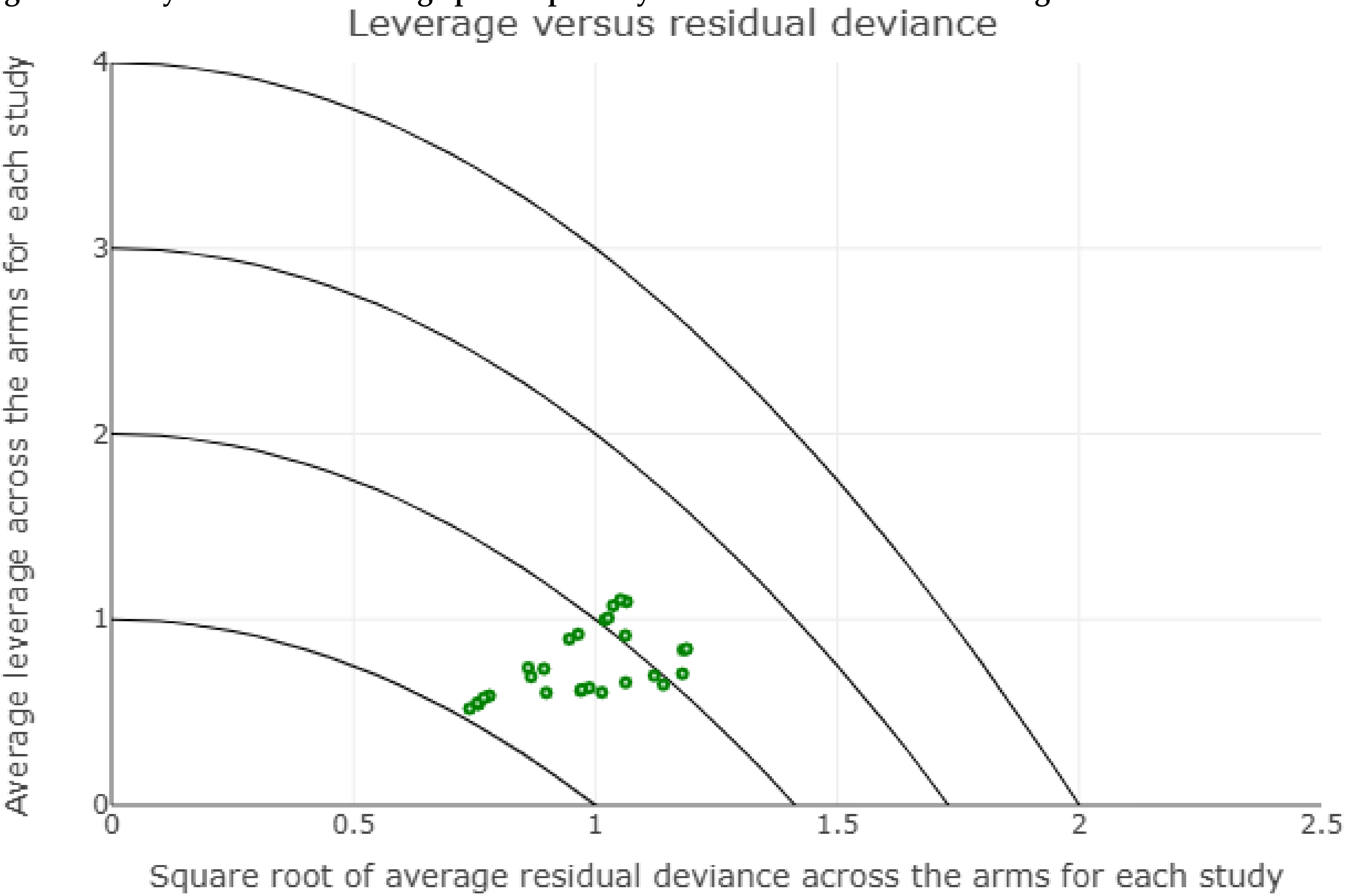

Figure S5D Bayesian-based residual deviance NMA/UME model of primary outcome: incidence of hearing loss in subgroup of dosage

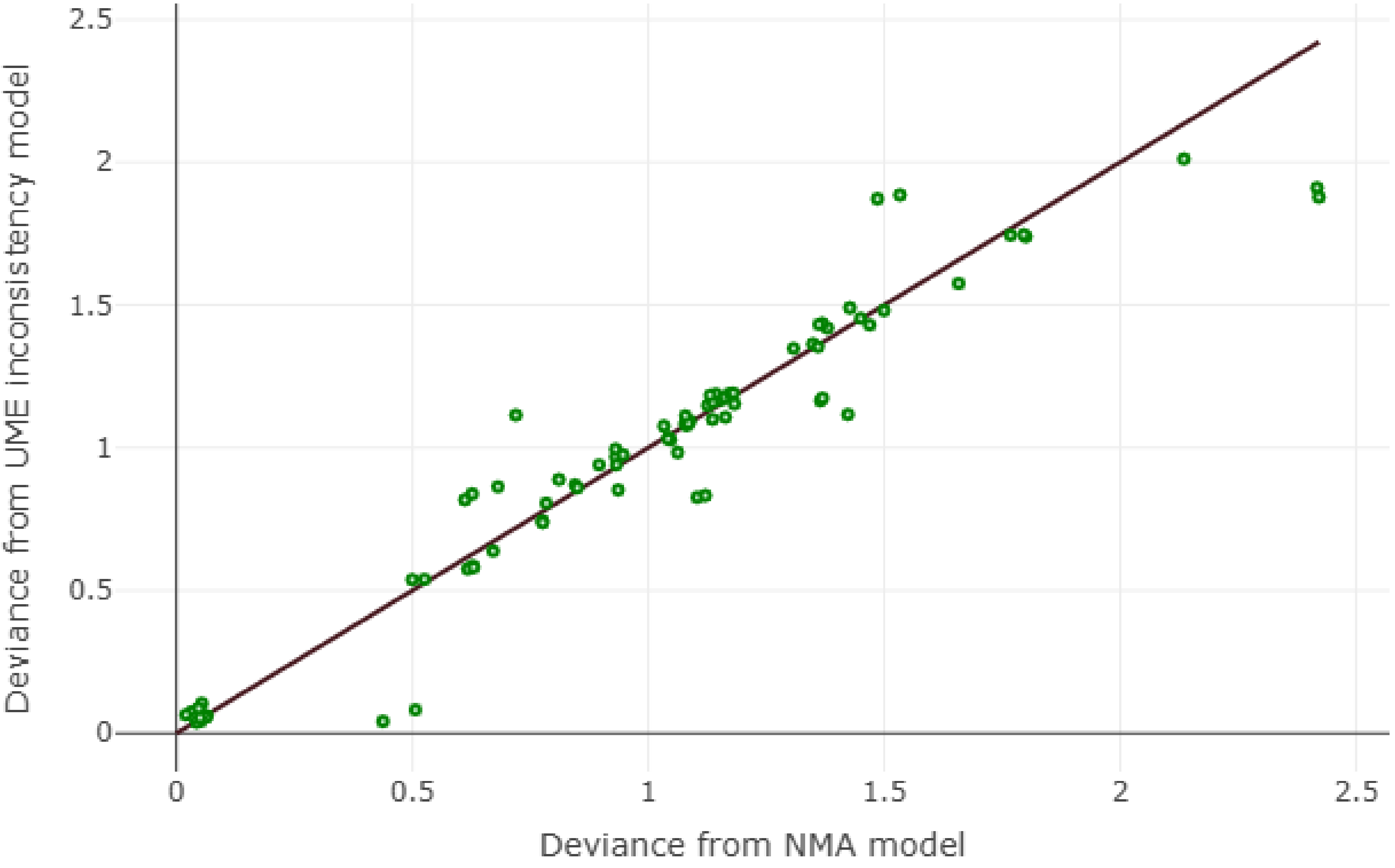

Figure S5E Bayesian-based per-arm residual deviance of primary outcome: incidence of hearing loss in subgroup of dosage

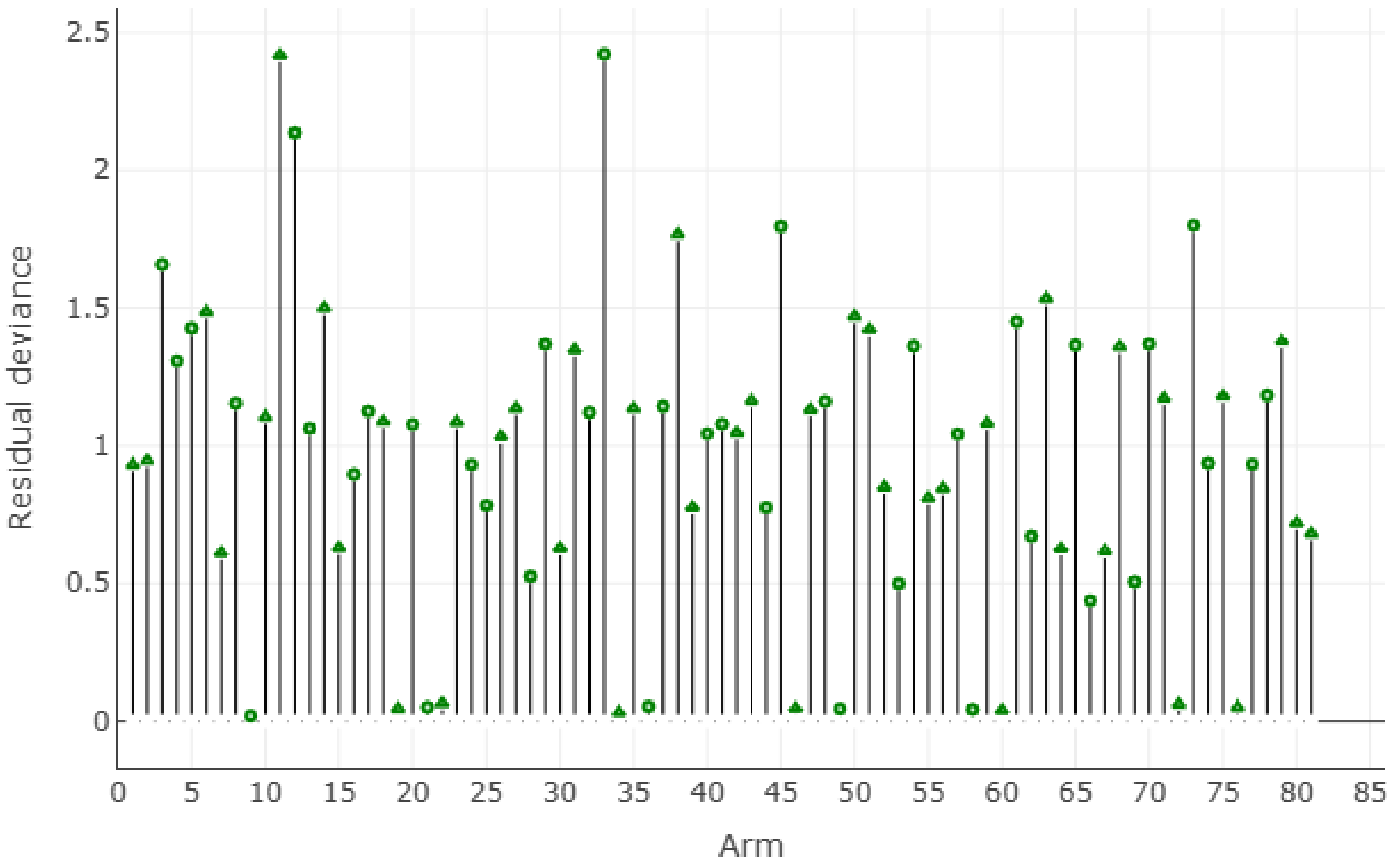

Figure S5F Bayesian-based leverage plot of primary outcome: incidence of hearing loss in subgroup of dosage

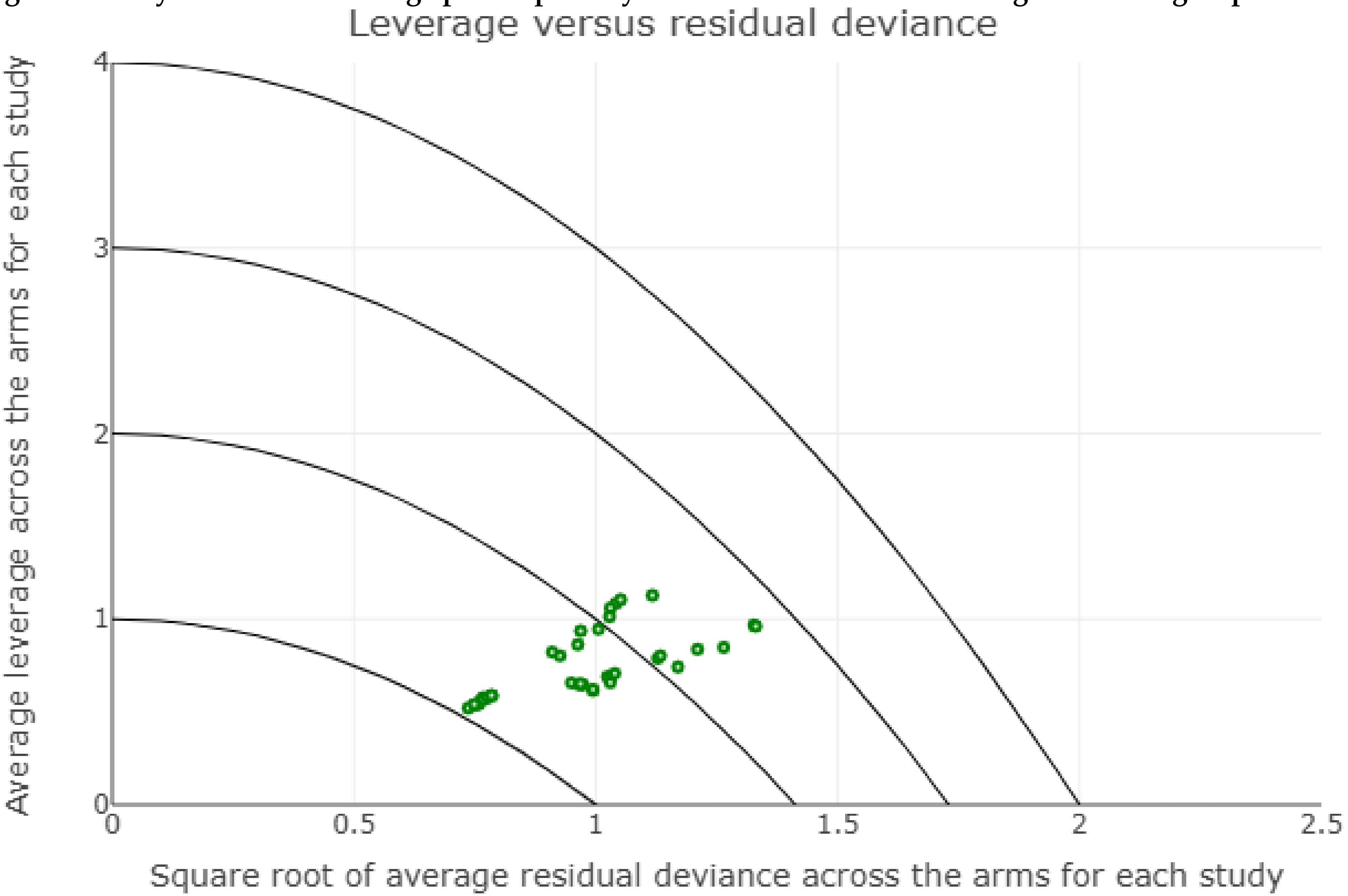

Figure S5G Bayesian-based residual deviance NMA/UME model of drop-out rate

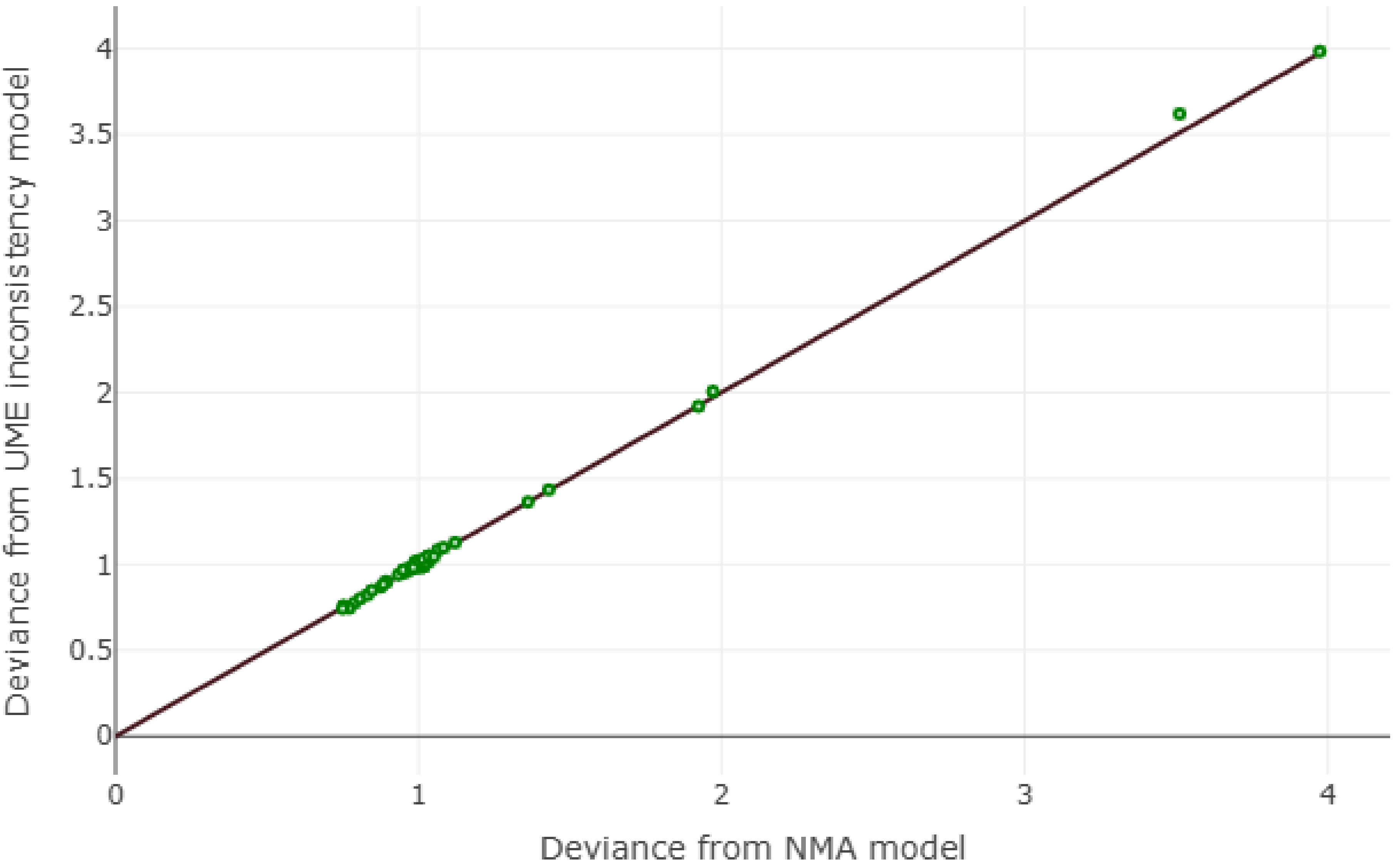

Figure S5H Bayesian-based per-arm residual deviance of drop-out rate

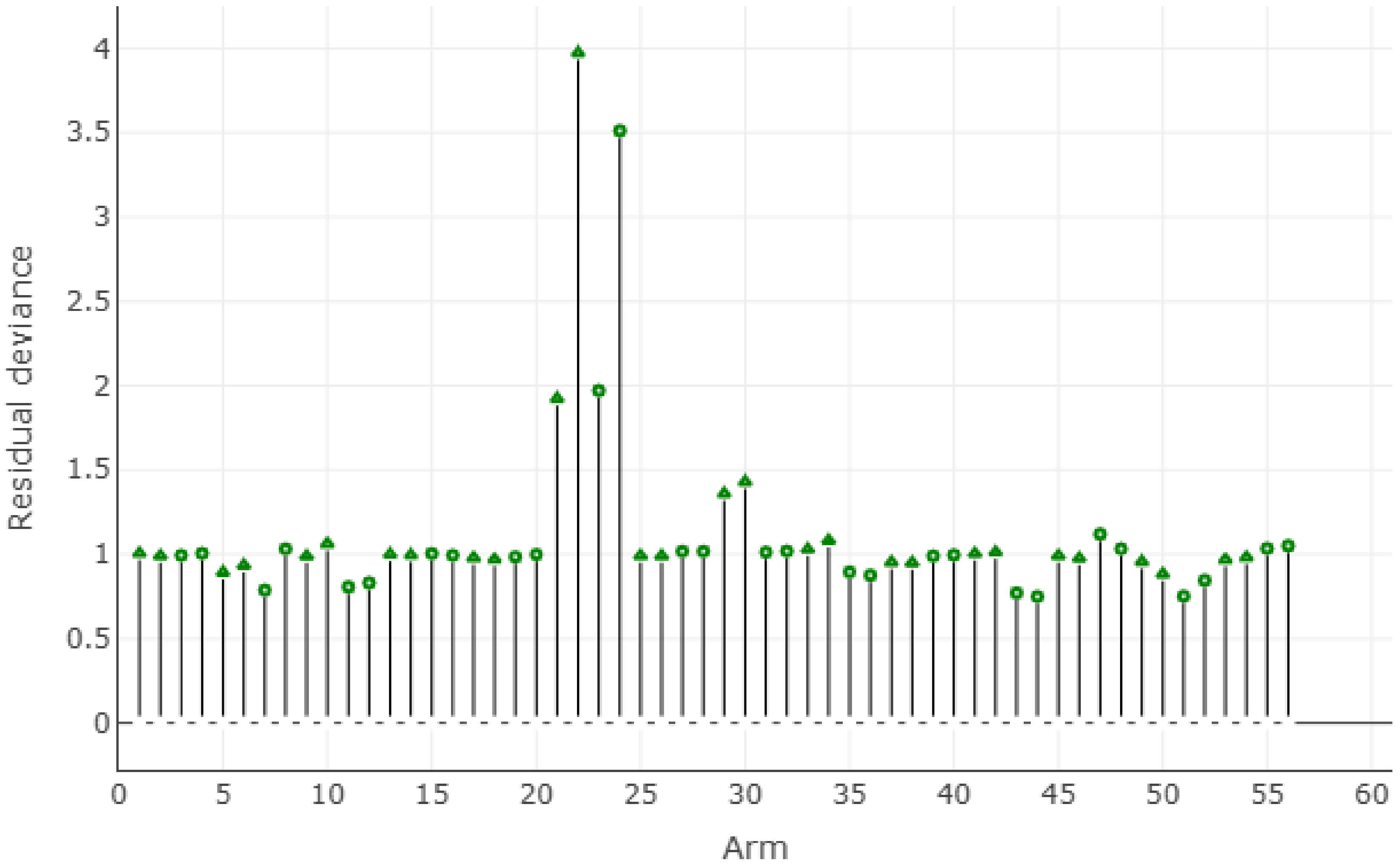

Figure S5I Bayesian-based leverage plot of drop-out rate

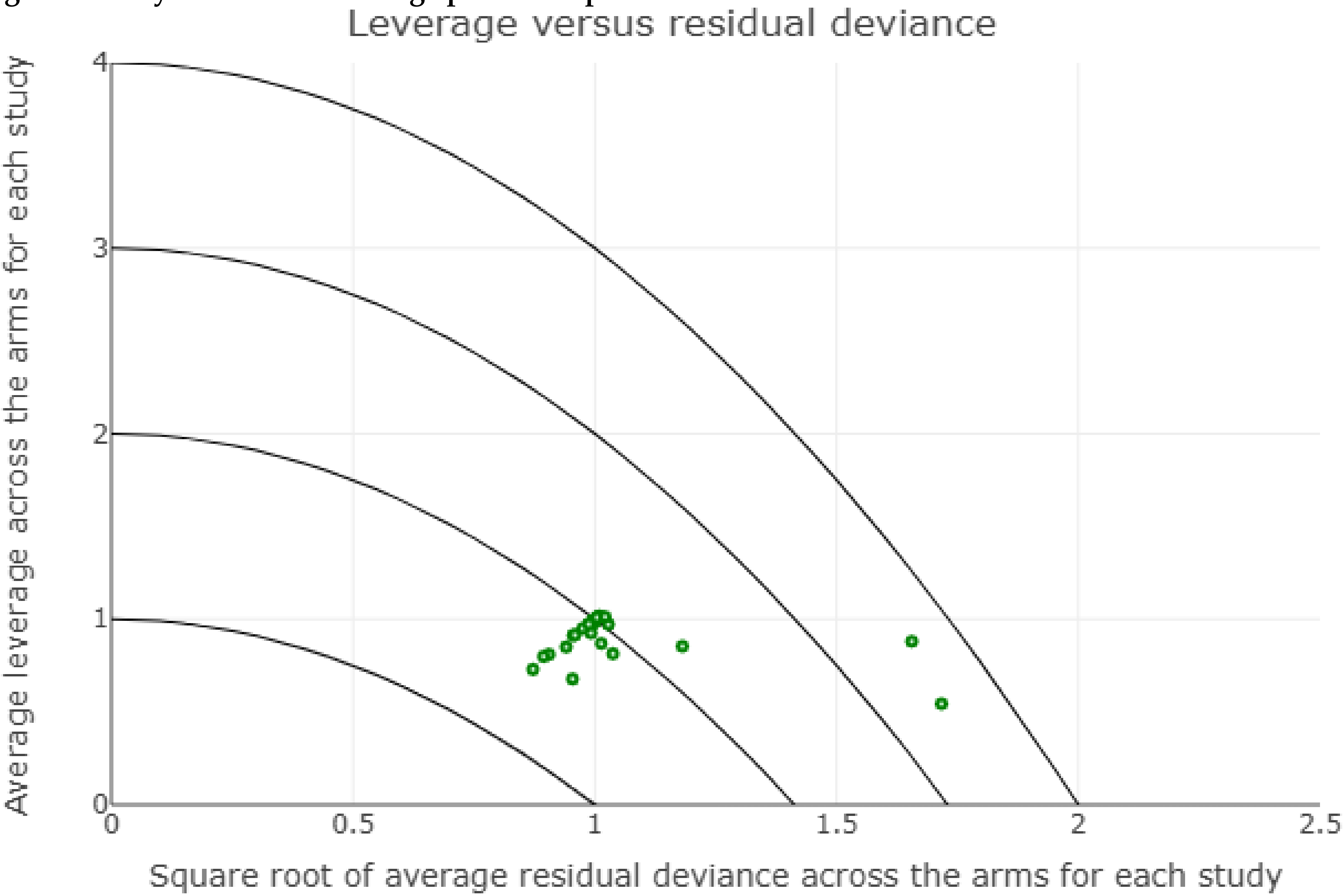

Abbreviation for Figure S5A-S5I:

95%CIs: 95% confidence intervals; GLP-1 agonist: glucagon-like peptide-1 agonist; NMA: network meta-analysis; OR: odds ratio; RCT: randomized controlled trial; SGLT2 inhibitor: sodium–glucose cotransporter 2 inhibitor

Figure S6A overview of risk of bias

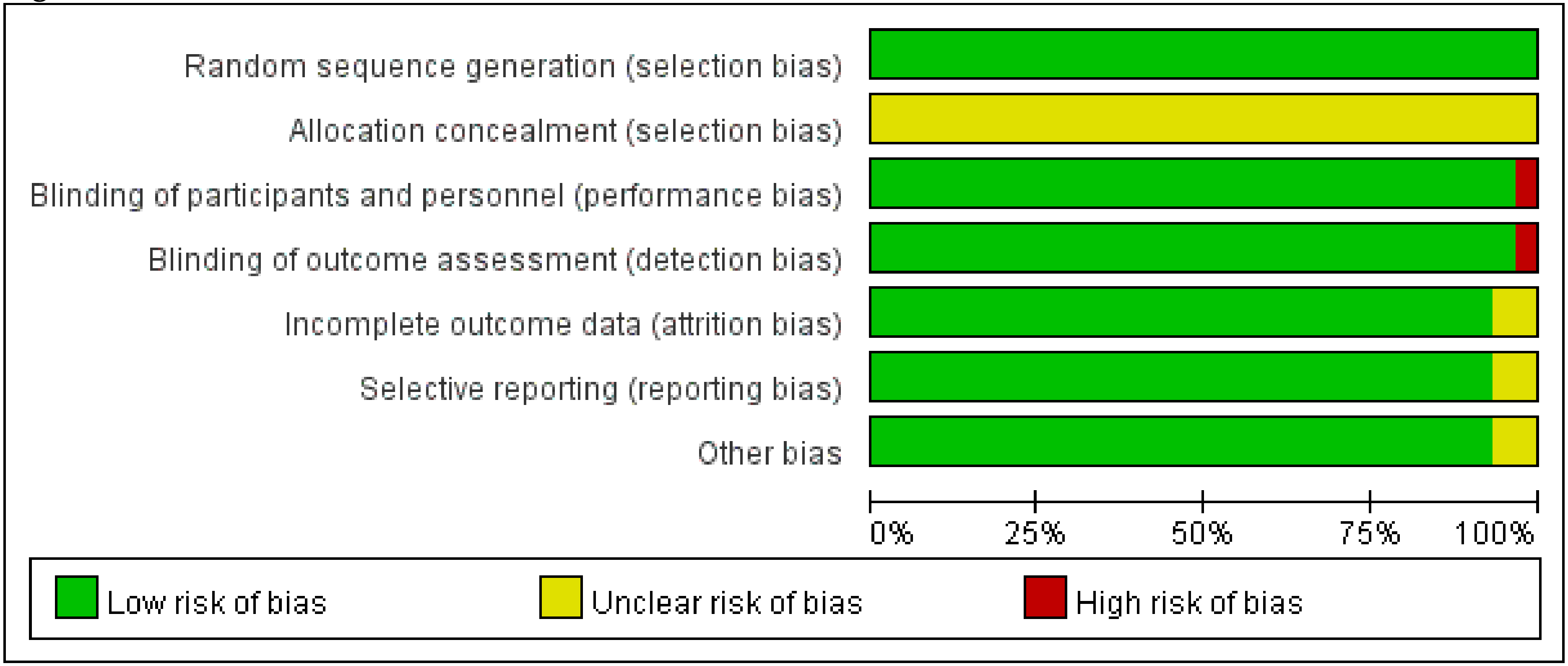

Figure S6B detailed risk of bias in each study

	Random sequence generation (selection bias)	Allocation concealment (selection bias)	Blinding of participants and personnel (performance bias)	Blinding of outcome assessment (detection bias)	Incomplete outcome data (attrition bias)	Selective reporting (reporting bias)	Other bias
Anker, S.D. (2021) (EMPEROR-Preserved)	+	?	+	+	+	+	+
Cannon, C.P. (2020) (VERTIS CV)	+	?	+	+	+	+	+
Dahl, D. (2022) (SURPASS-5)	+	?	+	+	+	+	+
Gallo, S. (2019) (VERTIS MET)	+	?	+	+	+	+	+
Gerstein, H.C. (2019) (REWIND)	+	?	+	+	+	+	+
Gerstein, H.C. (2021) (AMPLITUDE-O)	+	?	+	+	+	+	+
Hernandez, A.F. (2018) (Harmony Outcomes)	+	?	+	+	+	+	+
Herrington, W.G. (2023) (EMPA-KIDNEY)	+	?	+	+	+	+	+
Holman, R.R. (2017) (EXSCEL)	+	?	+	+	+	+	+
Ji, L. (2023)	+	?	+	+	+	+	+
Kadowaki, T. (2022) (SURPASS J-combo)	+	?	−	−	+	+	+
Lee, B.W. (2024) (SUSTAIN - CHINA MRCT)	+	?	+	+	+	+	+
Lincoff, A.M. (2023) (SELECT)	+	?	+	+	+	+	+
Marso, S.P. (2016) (LEADER)	+	?	+	+	+	+	+
Marso, S.P. (2016) (SUSTAIN-6)	+	?	+	+	+	+	+
Natale, P. (2024) (NCT02836873)	+	?	+	+	+	+	+
Neal, B. (2017) (CANVAS)	+	?	+	+	+	+	+
Neal, B. (2017) (CANVAS-R)	+	?	+	+	+	+	+
Packer, M. (2020) (EMPEROR-Reduced)	+	?	+	+	+	+	+
Pfeffer, M.A. (2015) (ELIXA)	+	?	+	+	+	+	+
Rosenstock, J. (2019) (PIONEER 3)	+	?	+	+	+	+	+
Solomon, S.D. (2022) (DELIVER)	+	?	+	+	+	+	+
SURMOUNT-J (2024) (NCT04844918)	+	?	+	+	?	?	?
Voors, A.A. (2022) (EMPULSE)	+	?	+	+	+	+	+
Wada, T. (2022) (TA-7284-14)	+	?	+	+	+	+	+
Wang, J. (2019) (AWARD-CHN2)	+	?	+	+	+	+	+
Wason, S. (2021) (SOTA-BONE) (NCT03386344)	+	?	+	+	?	?	?
Wiviott, S.D. (2019) (DECLARE-TIMI 58)	+	?	+	+	+	+	+
Zinman, B. (2015) (EMPA-REG OUTCOME)	+	?	+	+	+	+	+

Figure S7A Funnel plot of the primary outcome: incidence of hearing loss

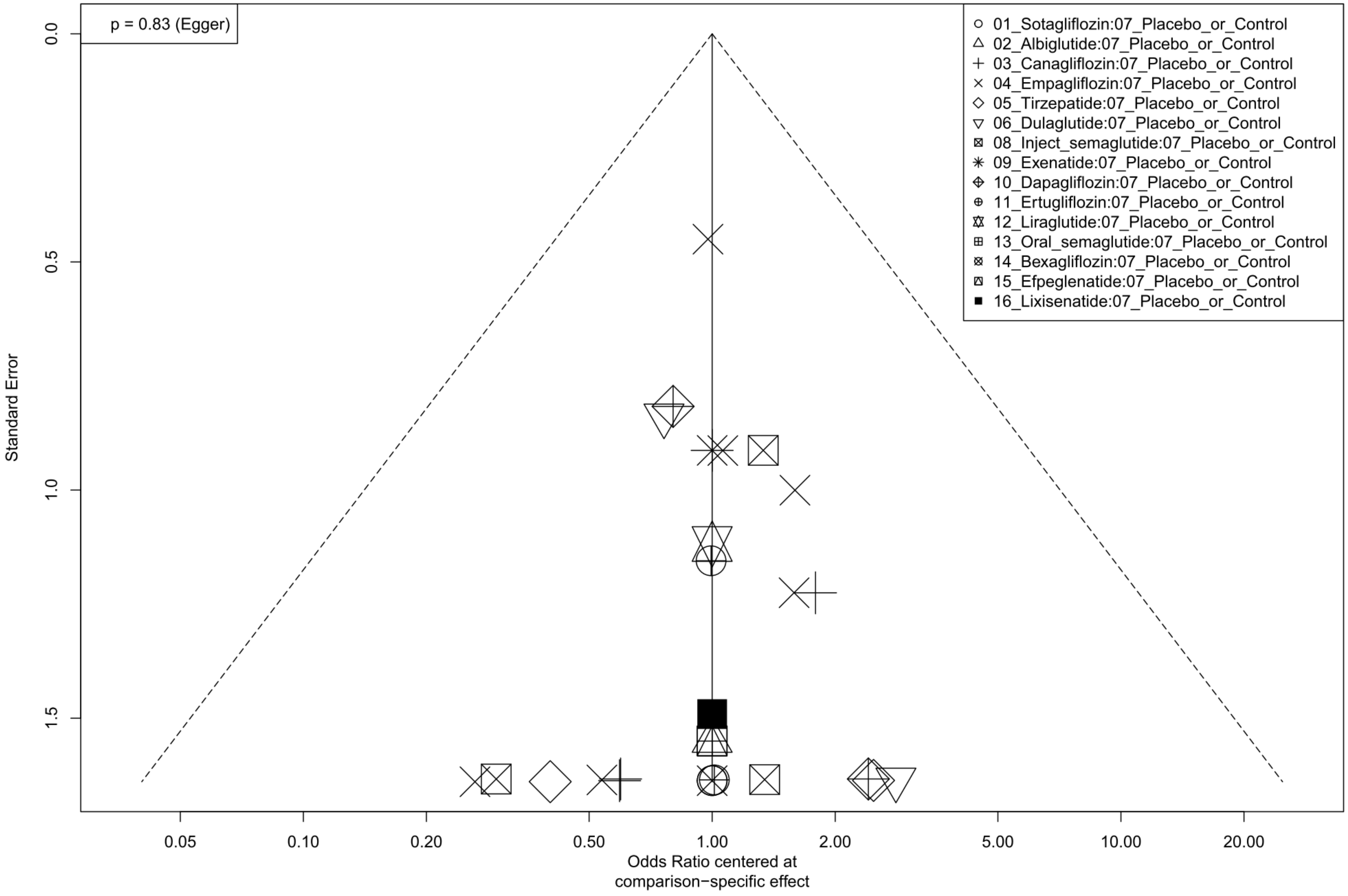

Figure S7B Funnel plot of the primary outcome: incidence of hearing loss in subgroup of dosage

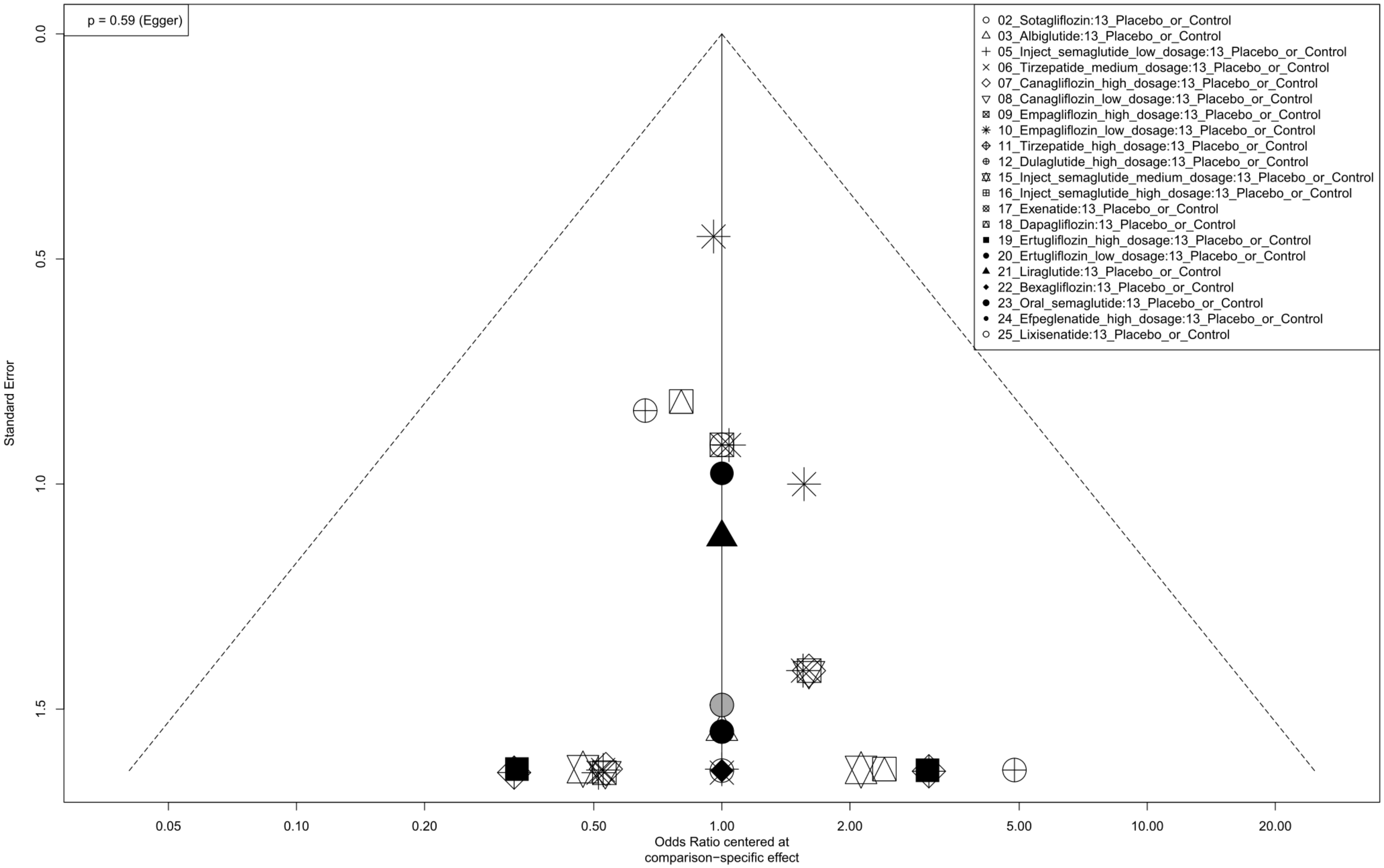

Table S1: PRISMA 2020 checklist of the current network meta-analysis

Section and Topic	Item #	Checklist item	Page where item is reported
**TITLE**			
Title	1	Identify the report as a systematic review.	1
**ABSTRACT**			
Abstract	2	See the PRISMA 2020 for Abstracts checklist.	7-8
**INTRODUCTION**			
Rationale	3	Describe the rationale for the review in the context of existing knowledge.	9-10
Objectives	4	Provide an explicit statement of the objective(s) or question(s) the review addresses.	9-10
**METHODS**			
Eligibility criteria	5	Specify the inclusion and exclusion criteria for the review and how studies were grouped for the syntheses.	11-12
Information sources	6	Specify all databases, registers, websites, organisations, reference lists and other sources searched or consulted to identify studies. Specify the date when each source was last searched or consulted.	11-12
Search strategy	7	Present the full search strategies for all databases, registers and websites, including any filters and limits used.	11-12
Selection process	8	Specify the methods used to decide whether a study met the inclusion criteria of the review, including how many reviewers screened each record and each report retrieved, whether they worked independently, and if applicable, details of automation tools used in the process.	11-12
Data collection process	9	Specify the methods used to collect data from reports, including how many reviewers collected data from each report, whether they worked independently, any processes for obtaining or confirming data from study investigators, and if applicable, details of automation tools used in the process.	11-12
Data items	10a	List and define all outcomes for which data were sought. Specify whether all results that were compatible with each outcome domain in each study were sought (e.g. for all measures, time points, analyses), and if not, the methods used to decide which results to collect.	12-13
	10b	List and define all other variables for which data were sought (e.g. participant and intervention characteristics, funding sources). Describe any assumptions made about any missing or unclear information.	12-13
Study risk of bias assessment	11	Specify the methods used to assess risk of bias in the included studies, including details of the tool(s) used, how many reviewers assessed each study and whether they worked independently, and if applicable, details of automation tools used in the process.	12-13
Effect measures	12	Specify for each outcome the effect measure(s) (e.g. risk ratio, mean difference) used in the synthesis or presentation of results.	12-13
Synthesis methods	13a	Describe the processes used to decide which studies were eligible for each synthesis (e.g. tabulating the study intervention characteristics and comparing against the planned groups for each synthesis (item #5)).	12-13
	13b	Describe any methods required to prepare the data for presentation or synthesis, such as handling of missing summary statistics, or data conversions.	13-15
	13c	Describe any methods used to tabulate or visually display results of individual studies and syntheses.	13-15
	13d	Describe any methods used to synthesize results and provide a rationale for the choice(s). If meta-analysis was performed, describe the	13-15

Section and Topic	Item #	Checklist item	Page where item is reported
		model(s), method(s) to identify the presence and extent of statistical heterogeneity, and software package(s) used.	
	13e	Describe any methods used to explore possible causes of heterogeneity among study results (e.g. subgroup analysis, meta-regression).	13-15
	13f	Describe any sensitivity analyses conducted to assess robustness of the synthesized results.	13-15
Reporting bias assessment	14	Describe any methods used to assess risk of bias due to missing results in a synthesis (arising from reporting biases).	13-15
Certainty assessment	15	Describe any methods used to assess certainty (or confidence) in the body of evidence for an outcome.	13-15
**RESULTS**			
Study selection	16a	Describe the results of the search and selection process, from the number of records identified in the search to the number of studies included in the review, ideally using a flow diagram.	16-17, Fig 1, Tab S2
	16b	Cite studies that might appear to meet the inclusion criteria, but which were excluded, and explain why they were excluded.	16-17, Tab S3
Study characteristics	17	Cite each included study and present its characteristics.	16-17, Tab S4
Risk of bias in studies	18	Present assessments of risk of bias for each included study.	16-17, Fig S6
Results of individual studies	19	For all outcomes, present, for each study: (a) summary statistics for each group (where appropriate) and (b) an effect estimate and its precision (e.g. confidence/credible interval), ideally using structured tables or plots.	16-17, Fig S3
Results of syntheses	20a	For each synthesis, briefly summarise the characteristics and risk of bias among contributing studies.	17-18, Fig 2
	20b	Present results of all statistical syntheses conducted. If meta-analysis was done, present for each the summary estimate and its precision (e.g. confidence/credible interval) and measures of statistical heterogeneity. If comparing groups, describe the direction of the effect.	17-18, Fig 3
	20c	Present results of all investigations of possible causes of heterogeneity among study results.	17-18, Tab S7
	20d	Present results of all sensitivity analyses conducted to assess the robustness of the synthesized results.	17-18
Reporting biases	21	Present assessments of risk of bias due to missing results (arising from reporting biases) for each synthesis assessed.	17-18, Fig S6
Certainty of evidence	22	Present assessments of certainty (or confidence) in the body of evidence for each outcome assessed.	17-18
**DISCUSSION**			
Discussion	23a	Provide a general interpretation of the results in the context of other evidence.	19-21
	23b	Discuss any limitations of the evidence included in the review.	21-22
	23c	Discuss any limitations of the review processes used.	21-22
	23d	Discuss implications of the results for practice, policy, and future research.	23
**OTHER INFORMATION**			
Registration and protocol	24a	Provide registration information for the review, including register name and registration number, or state that the review was not registered.	8

Section and Topic	Item #	Checklist item	Page where item is reported
	24b	Indicate where the review protocol can be accessed, or state that a protocol was not prepared.	8
	24c	Describe and explain any amendments to information provided at registration or in the protocol.	8
Support	25	Describe sources of financial or non-financial support for the review, and the role of the funders or sponsors in the review.	24
Competing interests	26	Declare any competing interests of review authors.	24
Availability of data, code and other materials	27	Report which of the following are publicly available and where they can be found: template data collection forms; data extracted from included studies; data used for all analyses; analytic code; any other materials used in the review.	24

The current checklist followed the latest PRISMA 2020 guideline [1].

Table S2: Keyword used in each database and search results

Database	Keyword	Filter	Date	Result
PubMed	(deafness OR deaf OR hearing impairment OR hearing loss OR sensorineural hearing loss OR conductive hearing loss OR hypoacusis) AND (glucagon-like peptide-1 receptor agonist OR Sodium Glucose Cotransporter 2 Inhibitor OR lixisenatide OR orforglipron OR exenatide OR semaglutide OR liraglutide OR albiglutide OR dulaglutide OR tirzepatide OR bexagliflozin OR canagliflozin OR dapagliflozin OR empagliflozin OR ertugliflozin OR ipragliflozin OR luseogliflozin OR remogliflozin OR sergliflozin OR sotagliflozin OR tofogliflozin OR henagliflozin OR janagliflozin OR mizagliflozin OR velagliflozin OR enavogliflozin OR licogliflozin OR rongliflozin) AND (random OR randomized OR randomised)	N/A	2025/02/01	0
ClinicalKey	(deafness OR deaf OR hearing impairment OR hearing loss OR sensorineural hearing loss OR conductive hearing loss OR hypoacusis) AND (glucagon-like peptide-1 receptor agonist OR Sodium Glucose Cotransporter 2 Inhibitor OR lixisenatide OR orforglipron OR exenatide OR semaglutide OR liraglutide OR albiglutide OR dulaglutide OR tirzepatide OR bexagliflozin OR canagliflozin OR dapagliflozin OR empagliflozin OR ertugliflozin OR ipragliflozin OR luseogliflozin OR remogliflozin OR sergliflozin OR sotagliflozin OR tofogliflozin OR henagliflozin OR janagliflozin OR mizagliflozin OR velagliflozin OR enavogliflozin OR licogliflozin OR rongliflozin) AND (random OR randomized	N/A	2025/02/01	893

	OR randomised)			
Cochrane CENTRAL	(deafness OR deaf OR hearing impairment OR hearing loss OR sensorineural hearing loss OR conductive hearing loss OR hypoacusis) AND (glucagon-like peptide-1 receptor agonist OR Sodium Glucose Cotransporter 2 Inhibitor OR lixisenatide OR orforglipron OR exenatide OR semaglutide OR liraglutide OR albiglutide OR dulaglutide OR tirzepatide OR bexagliflozin OR canagliflozin OR dapagliflozin OR empagliflozin OR ertugliflozin OR ipragliflozin OR luseogliflozin OR remogliflozin OR sergliflozin OR sotagliflozin OR tofogliflozin OR henagliflozin OR janagliflozin OR mizagliflozin OR velagliflozin OR enavogliflozin OR licogliflozin OR rongliflozin) AND (random OR randomized OR randomised)	N/A	2025/02/01	3
Embase	(deafness OR deaf OR hearing impairment OR hearing loss OR sensorineural hearing loss OR conductive hearing loss OR hypoacusis) AND (glucagon-like peptide-1 receptor agonist OR Sodium Glucose Cotransporter 2 Inhibitor) AND (random OR randomized OR randomised)	N/A	2025/02/01	26
ProQuest	(deafness OR deaf OR hearing impairment OR hearing loss OR sensorineural hearing loss OR conductive hearing loss OR hypoacusis) AND (glucagon-like peptide-1 receptor agonist OR Sodium Glucose Cotransporter 2 Inhibitor) AND (random OR randomized OR randomised)	N/A	2025/02/01	659
ScienceDirect	(deafness OR deaf OR hearing impairment OR hearing loss OR sensorineural hearing loss OR conductive hearing loss OR hypoacusis) AND (glucagon-like peptide-1 receptor agonist OR Sodium Glucose Cotransporter 2 Inhibitor)	N/A	2025/02/01	1249

Web of Science	(deafness OR deaf OR hearing impairment OR hearing loss OR sensorineural hearing loss OR conductive hearing loss OR hypoacusis) AND (glucagon-like peptide-1 receptor agonist OR Sodium Glucose Cotransporter 2 Inhibitor)	N/A	2025/02/01	14
ClinicalTrials.gov	(deafness OR deaf OR hearing impairment OR hearing loss OR sensorineural hearing loss OR conductive hearing loss OR hypoacusis) AND (glucagon-like peptide-1 receptor agonist OR Sodium Glucose Cotransporter 2 Inhibitor)	N/A	2025/02/01	1

Abbreviation: N/A: not applied

Table S3: Excluded studies and reason

Reason	Numbers	References
Animal study	1	[2]
Case report	1	[3]
Duplicate sample source with another included trial	6	[4-9]
Not report target outcome	108	[10-117]
Review article	2	[118,119]

Table S4: Characteristics of the included studies

Study name	Baseline illness	Comparison	Subjects	Mean age (year)	Female (%)	Treatment duration	Route	Category	ClinicalTrials.gov	Country
Lee, B.W. (2024) (SUSTAIN - CHINA MRCT)[120]	patients with type 2 diabetes mellitus	Inject semaglutide 0.5 mg<br>Inject semaglutide 1.0 mg<br>Control with Sitagliptin	35<br>40<br>35	54.3±12.9<br>55.7±10.3<br>56.2±11.3	51.4<br>50.0<br>40.0	30 weeks	injection	GLP-1 agonist	NCT03061214	Multiple countries
Natale, P. (2024) (NCT02836873)[121]	patients with type 2 diabetes mellitus and chronic kidney disease	Bexagliflozin 20mg<br>Placebo	157<br>155	69.3±8.4<br>69.9±8.3	41.4<br>32.9	24 weeks	oral	SGLT2 inhibitor	NCT02836873	Multiple countries
SURMOUNT-J (2024) (NCT04844918)[122]	patients with obesity	Tirzepatide 10mg<br>Tirzepatide 15mg<br>Placebo	73<br>77<br>75	49.0±10.9<br>51.1±10.3<br>52.3±10.9	41.1<br>41.6<br>40.0	72 weeks	injection	GLP-1 agonist	NCT04844918	Japan
Herrington, W.G. (2023) (EMPA-KIDNEY)[123]	patients with renal failure	Empagliflozin 10mg<br>Placebo	3304<br>3305	63.9±13.9<br>63.8±13.9	33.2<br>33.1	104 weeks	oral	SGLT2 inhibitor	NCT03594110	Multiple countries
Ji, L. (2023)[124]	patients with type 2 diabetes mellitus	Empagliflozin 10mg<br>Empagliflozin 25mg<br>Placebo	73<br>73<br>73	59.9±7.7<br>60.7±9.1<br>60.1±8.0	41.1<br>45.2<br>50.7	24 weeks	oral	SGLT2 inhibitor	NCT04233801	China
Lincoff, A.M. (2023) (SELECT)[125]	patients with obesity	Inject Semaglutide 2.4mg<br>Placebo	8803<br>8801	61.6±8.9<br>61.6±8.8	27.8<br>27.5	104 weeks	injection	GLP-1 agonist	NCT03574597	Multiple countries
Dahl, D. (2022) (SURPASS-5)[126]	patients with type 2 diabetes mellitus	Tirzepatide 5mg<br>Tirzepatide 10mg<br>Tirzepatide 15mg<br>Placebo	116<br>119<br>120<br>120	62.0±10.0<br>60.0±10.0<br>61.0±10.0<br>60.0±10.0	47.4<br>39.5<br>45.8<br>45.0	40 weeks	injection	GLP-1 agonist	NCT04039503	Multiple countries
Kadowaki, T. (2022) (SURPASS J-combo)[127]	patients with type 2 diabetes mellitus	Tirzepatide 5mg<br>Tirzepatide 10mg<br>Tirzepatide 15mg	148<br>147<br>148	57.7±11.0<br>56.9±11.2<br>56.5±10.4	19.6<br>23.1<br>29.7	56 weeks	injection	GLP-1 agonist	NCT03861039	Japan
Solomon, S.D. (2022) (DELIVER)[128]	patients with stabilized heart failure	Dapagliflozin 10mg<br>Placebo	3131<br>3132	71.8±9.6<br>71.5±9.5	43.6<br>44.2	120 weeks	oral	SGLT2 inhibitor	NCT03619213	Multiple countries

Voors, A.A. (2022) (EMPULSE)[129]	patients with acute heart failure and dyspnea	Empagliflozin 10mg<br>Placebo	265<br>265	71.0<br>70.0	32.5<br>35.1	13 weeks	oral	SGLT2 inhibitor	NCT04157751	Multiple countries
Wada, T. (2022) (TA-7284-14)[130]	patients with type 2 diabetes mellitus and chronic kidney disease	Canagliflozin 100 mg<br>Placebo	154<br>154	62.5±10.5<br>62.4±11.1	25.3<br>16.2	104 weeks	oral	SGLT2 inhibitor	NCT03436693	Japan
Anker, S.D. (2021) (EMPEROR-Preserved)[131]	patients with heart failure with preserved ejection fraction	Empagliflozin 10mg<br>Placebo	2997<br>2991	71.8±9.3<br>71.9±9.6	44.6<br>44.7	156 weeks	oral	SGLT2 inhibitor	NCT03057951	Multiple countries
Gerstein, H.C. (2021) (AMPLITUDE-O)[132]	patients with type 2 diabetes mellitus	Efpeglenatide 4 mg<br>Efpeglenatide 6 mg<br>Placebo	1359<br>1358<br>1359	64.6±8.2<br>64.7±8.2<br>64.4±8.3	32.5<br>35.6<br>30.8	104 weeks	injection	GLP-1 agonist	NCT03496298	Multiple countries
Wason, S. (2021) (SOTA-BONE) (NCT03386344)[133]	patients with type 2 diabetes mellitus	Sotagliflozin 200-400mg<br>Placebo	250<br>126	66.3±6.8<br>66.3±5.7	44.4<br>44.4	110 weeks	oral	SGLT2 inhibitor	NCT03386344	Multiple countries
Cannon, C.P. (2020) (VERTIS CV)[134]	patients with type 2 diabetes mellitus	Ertugliflozin 5 mg<br>Ertugliflozin 15 mg<br>Placebo	2752<br>2747<br>2747	64.3±8.2<br>64.4±8.0<br>64.4±8.0	29.1<br>30.3<br>30.7	182 weeks	oral	SGLT2 inhibitor	NCT01986881	Multiple countries
Packer, M. (2020) (EMPEROR-Reduced)[135]	patients with chronic heart failure	Empagliflozin 10mg<br>Placebo	1863<br>1867	67.2±10.8<br>66.5±11.2	23.5<br>24.4	64 weeks	oral	SGLT2 inhibitor	NCT03057977	Multiple countries
Gallo, S. (2019) (VERTIS MET)[136]	patients with type 2 diabetes mellitus	Ertugliflozin 5 mg<br>Ertugliflozin 15 mg<br>Placebo with glimepiride	207<br>205<br>209	56.6±8.2<br>56.9±9.4<br>56.5±8.7	53.1<br>54.6<br>53.1	104 weeks	oral	SGLT2 inhibitor	NCT02033889	Multiple countries
Gerstein, H.C. (2019) (REWIND)[137]	patients with type 2 diabetes mellitus	Dulaglutide 1.5 mg<br>Placebo	4949<br>4952	66.2±6.5<br>66.2±6.5	46.6<br>46.1	281 weeks	injection	GLP-1 agonist	NCT01394952	Multiple countries
Rosenstock, J. (2019) (PIONEER 3)[138]	patients with type 2 diabetes mellitus	Oral semaglutide 3-14mg<br>Control with sitagliptin	1396<br>466	57.7±10.0<br>58.0±10.0	46.6<br>49.0	78 weeks	oral	GLP-1 agonist	NCT02607865	Multiple countries
Wang, J. (2019) (AWARD-CHN2)[139]	patients with type 2 diabetes mellitus	Dulaglutide 1.5 mg<br>Dulaglutide 0.75 mg<br>Placebo with insulin glargine	253<br>252<br>250	55.0±9.6<br>54.5±10.0<br>55.4±9.2	46.6<br>43.3<br>44.4	52 weeks	injection	GLP-1 agonist	NCT01648582	Multiple countries
Wiviott, S.D. (2019) (DECLARE-TIMI 58)[140]	patients with atherosclerotic vascular disease	Dapagliflozin 10mg<br>Placebo	8582<br>8578	63.9±6.8<br>64.0±6.8	36.9<br>37.9	206 weeks	oral	SGLT2 inhibitor	NCT01730534	Multiple countries

Hernandez, A.F. (2018) (Harmony Outcomes)[141]	patients with type 2 diabetes mellitus	Albiglutide 50 mg<br>Placebo	4731<br>4732	64.1±8.7<br>64.2±8.7	30.2<br>31.0	86 weeks	injection	GLP-1 agonist	NCT02465515	Multiple countries
Holman, R.R. (2017) (EXSCEL)[142]	patients with type 2 diabetes mellitus	Exenatide 2mg<br>Placebo	7356<br>7396	61.8±9.4<br>61.9±9.4	38.0<br>38.0	166 weeks	injection	GLP-1 agonist	NCT01144338	Multiple countries
Neal, B. (2017) (CANVAS)[143]	patients with type 2 diabetes mellitus	Canagliflozin 100 mg<br>Canagliflozin 300 mg<br>Placebo	1445<br>1443<br>1442	62.2±8.0<br>62.8±8.1<br>62.3±7.9	33.5<br>34.6<br>33.7	126 weeks	oral	SGLT2 inhibitor	NCT01032629	Multiple countries
Neal, B. (2017) (CANVAS-R)[143]	patients with type 2 diabetes mellitus	Canagliflozin 300 mg<br>Placebo	2907<br>2905	63.9±8.4<br>64.0±8.3	36.2<br>38.2	126 weeks	oral	SGLT2 inhibitor	NCT01989754	Multiple countries
Marso, S.P. (2016) (LEADER)[144]	patients with type 2 diabetes mellitus	Liraglutide 1.8mg<br>Placebo	4668<br>4672	64.2±7.2<br>64.4±7.2	35.5<br>36.0	198 weeks	injection	GLP-1 agonist	NCT01179048	Multiple countries
Marso, S.P. (2016) (SUSTAIN-6)[145]	patients with type 2 diabetes mellitus	Inject semaglutide 0.5 mg<br>Inject semaglutide 1.0 mg<br>Placebo	826<br>822<br>1649	NA	40.1<br>37.0<br>40.0	109 weeks	injection	GLP-1 agonist	NCT01720446	Multiple countries
Pfeffer, M.A. (2015) (ELIXA)[146]	patients with type 2 diabetes mellitus and recent acute coronary syndrome	Lixisenatide 20ug<br>Placebo	3034<br>3034	59.9±9.7<br>60.6±9.6	30.4<br>30.9	100 weeks	injection	GLP-1 agonist	NCT01147250	Multiple countries
Zinman, B. (2015) (EMPA-REG OUTCOME)[147]	patients with type 2 diabetes mellitus	Empagliflozin 10mg<br>Empagliflozin 25mg<br>Placebo	2345<br>2342<br>2333	63.0±8.6<br>63.2±8.6<br>63.2±8.8	29.5<br>28.1<br>28.0	135 weeks	oral	SGLT2 inhibitor	NCT01131676	Multiple countries

Abbreviations: GLP-1 agonist: glucagon-like peptide-1 agonist; NA: not available; SGLT2 inhibitor: sodium–glucose cotransporter 2 inhibitor

Table S5A: League table of NMA of primary outcome: incidence of hearing loss (risk ratio)

01_Sotagliflozin						0.17 [0.01; 4.07]									
10.59 (0, 9390489232299.15)	02_Albiglutide					0.20 [0.01; 4.16]									
0 (0, 4.94)	0 (0, 1.26)	03_Canagliflozin				0.56 [0.11; 2.89]									
0 (0, 2.03)	***0 (0, 0.64)**	0.63 (0.04, 7.77)	04_Empagliflozin			0.63 [0.32; 1.23]									
0 (0, 5.64)	0 (0, 1.43)	0.94 (0.01, 88.88)	1.49 (0.03, 94.28)	05_Tirzepatide		0.41 [0.04; 3.93]									
0 (0, 2.49)	***0 (0, 0.7)**	0.59 (0.01, 12.91)	0.97 (0.06, 9.47)	0.61 (0.01, 51.53)	06_Dulaglutide	0.53 [0.12; 2.26]									
0 (0, 1.07)	***0 (0, 0.32)**	0.35 (0.02, 3.24)	0.57 (0.17, 1.61)	0.38 (0.01, 16.83)	0.59 (0.08, 6.36)	07_Placebo_ or_Control	0.89 [0.22; 3.62]	0.66 [0.11; 3.97]	0.80 [0.19; 3.36]	0.66 [0.10; 4.21]	0.25 [0.03; 2.23]	0.60 [0.03; 12.44]	0.34 [0.01; 8.22]	0.40 [0.02; 8.35]	0.11 [0.01; 2.06]
0 (0, 1.2)	***0 (0, 0.36)**	0.27 (0.01, 5.2)	0.45 (0.04, 4.42)	0.3 (0, 21.2)	0.47 (0.03, 11.35)	0.79 (0.1, 6.22)	08_Inject_ semaglutide								
***0 (0, 0.98)**	***0 (0, 0.29)**	0.23 (0, 6.22)	0.36 (0.02, 5.38)	0.21 (0, 23.67)	0.36 (0.01, 12.03)	0.63 (0.04, 7.86)	0.79 (0.03, 19.36)	09_Exenatide							
***0 (0, 0.87)**	***0 (0, 0.26)**	0.23 (0.01, 4.1)	0.37 (0.02, 3.33)	0.22 (0, 17.37)	0.38 (0.02, 7.65)	0.65 (0.06, 4.69)	0.81 (0.03, 13.01)	1.03 (0.03, 25.86)	10_Dapagliflozin						
***0 (0, 0.52)**	***0 (0, 0.13)**	0.12 (0, 3.51)	0.2 (0, 2.86)	0.11 (0, 12.2)	0.2 (0, 5.47)	0.35 (0.01, 4.13)	0.41 (0.01, 12.29)	0.53 (0.01, 18.66)	0.5 (0.01, 15.94)	11_Ertugliflozin					
***0 (0, 0.34)**	***0 (0, 0.09)**	0.06 (0, 1.84)	0.11 (0, 1.76)	0.06 (0, 6.84)	0.1 (0, 3.97)	0.18 (0, 2.69)	0.23 (0, 6.3)	0.29 (0, 12.7)	0.28 (0, 10.83)	0.53 (0.01, 41.36)	12_Liraglutide				
***0 (0, 0.01)**	***0 (0, 0)**	***0 (0, 0.81)**	***0 (0, 0.82)**	0 (0, 1.43)	0 (0, 1.03)	0 (0, 1.36)	0 (0, 2.06)	0 (0, 3.05)	0 (0, 2.73)	0 (0, 7.04)	0 (0, 21.46)	13_Oral_ semaglutide			
***0 (0, 0.02)**	***0 (0, 0.01)**	***0 (0, 0.96)**	0 (0, 1.54)	0 (0, 1.98)	0 (0, 1.77)	0 (0, 2.48)	0 (0, 4.05)	0 (0, 5.55)	0 (0, 5.42)	0 (0, 12.18)	0 (0, 41.37)	4.7 (0, 857048281282911)	14_Bexagliflozin		
***0 (0, 0.01)**	***0 (0, 0)**	***0 (0, 0.51)**	***0 (0, 0.66)**	***0 (0, 0.97)**	***0 (0, 0.93)**	0 (0, 1.12)	0 (0, 1.95)	0 (0, 2.24)	0 (0, 1.69)	0 (0, 5.96)	0 (0, 15.48)	0.66 (0, 22845261856380.6)	0.15 (0, 27022192959235.1)	15_Efpeglenatide	
***0 (0, 0)**	***0 (0, 0)**	***0 (0, 0.2)**	***0 (0, 0.2)**	***0 (0, 0.18)**	***0 (0, 0.21)**	***0 (0, 0.33)**	***0 (0, 0.54)**	***0 (0, 0.64)**	***0 (0, 0.54)**	0 (0, 1.75)	0 (0, 2.98)	5.77 (0, 247978669688448)	0.78 (0, 273864502625991)	10.83 (0, 298889784842481)	16_Lixisenatide

Data presented as RR [95%CIs]. Pairwise (upper-right portion) and network (lower-left portion) meta-analysis results are presented as estimate effect sizes for the outcome of overall events of hearing loss. Interventions are reported in order of mean ranking of

beneficially prophylactic effect on overall events of hearing loss, and outcomes are expressed as risk ratio (RR) (95% confidence intervals) (95%CIs). For the pairwise meta-analyses, RR of less than 1 indicates that the treatment specified in the row got more beneficial effect than that specified in the column. For the network meta-analysis (NMA), RR of less than 1 indicates that the treatment specified in the column got more beneficial effect than that specified in the row. Bold results indicate statistical significance.

Table S5B: League table of NMA of primary outcome: incidence of hearing loss in subgroup of dosage (risk ratio)

01_Tirzepatide _low_dosage					0.33 [0.01; 8.06]																			
9.08 (0, 5891225398624785)	02_Sotagliflozin											0.17 [0.01; 4.07]												
11.15 (0, 23348979466865644)	3.85 (0, 22280407584710284)	03_Albiglutide										0.20 [0.01; 4.16]												
3.23 (0, 2061780217436191232)	0.3 (0, 2607400040445676032)	0.28 (0, 375661120095617)	04_Dulaglutide _low_dosage								0.33 [0.01; 8.18]													
0.17 (0, 3456104678347.27)	0.04 (0, 3948730756245.19)	0.03 (0, 1123315179887.12)	0.04 (0, 8259307785814.53)	05_Inject_ semaglutide_ low_dosage								0.67 [0.03; 16.31]		0.34 [0.01; 8.23]										
0 (0, 0.59)	0 (0, 5.86)	0 (0, 4.73)	0 (0, 21.16)	0 (0, 13.85)	06_Tirzepatide _medium_dosage					1.01 [0.11; 9.62]		0.34 [0.01; 8.27]												
0 (0, 1.72)	0 (0, 4.39)	0 (0, 2.33)	0 (0, 11.82)	0 (0, 5.18)	0.78 (0, 192.65)	07_Canagliflozin _high_dosage	1.00 [0.06; 15.93]					0.62 [0.08; 5.07]												
0 (0, 1.56)	0 (0, 5.38)	0 (0, 2.43)	0 (0, 13.83)	0 (0, 6.92)	0.75 (0, 210.47)	1 (0.02, 67.97)	08_Canagliflozin _low_dosage					0.62 [0.08; 5.05]												
0 (0, 1.29)	0 (0, 4.36)	0 (0, 2.07)	0 (0, 8.77)	0 (0, 5.37)	0.69 (0, 160.8)	0.92 (0.01, 122.35)	0.88 (0.01, 105.41)	09_Empagliflozin_ high_dosage	1.00 [0.06; 15.96]			0.62 [0.08; 5.03]												
***0 (0, 0.39)**	0 (0, 1.23)	***0 (0, 0.62)**	0 (0, 3.44)	0 (0, 1.94)	0.39 (0, 28.62)	0.54 (0.01, 15.19)	0.52 (0.01, 14.56)	0.6 (0.01, 12.75)	10_Empagliflozin_ low_dosage			0.64 [0.33; 1.26]												
***0 (0, 0.28)**	0 (0, 2.58)	0 (0, 1.39)	0 (0, 6.65)	0 (0, 3.43)	0.46 (0.01, 13.17)	0.65 (0, 82.49)	0.59 (0, 77.38)	0.69 (0, 69.49)	1.18 (0.03, 59.18)	11_Tirzepatide _high_dosage		0.99 [0.10; 9.38]												
***0 (0, 0.29)**	0 (0, 1.01)	***0 (0, 0.43)**	0 (0, 1.04)	0 (0, 1.31)	0.24 (0, 22.77)	0.34 (0, 14.88)	0.33 (0, 14.65)	0.35 (0, 14.66)	0.68 (0.02, 9.05)	0.53 (0, 30.67)	12_Dulaglutide _high_dosage	0.61 [0.14; 2.61]												
***0 (0, 0.17)**	***0 (0, 0.61)**	***0 (0, 0.28)**	0 (0, 1.36)	***0 (0, 0.79)**	0.2 (0, 9.85)	0.28 (0, 5.08)	0.27 (0, 4.84)	0.3 (0.01, 5.29)	0.53 (0.1, 1.86)	0.44 (0.01, 11.53)	0.77 (0.07, 16.3)	13_Placebo_ or_Control		0.71 [0.07; 6.77]	0.67 [0.11; 3.99]	0.66 [0.11; 3.97]	0.80 [0.19; 3.36]	0.99 [0.10; 9.49]	0.43 [0.06; 2.90]	0.25 [0.03; 2.23]	0.34 [0.01; 8.22]	0.60 [0.03; 12.44]	0.20 [0.01; 4.17]	0.11 [0.01; 2.06]
0 (0, 60321605.77)	0 (0, 98506891.96)	0 (0, 104448534.99)	0 (0, 4277802.71)	0 (0, 431568952.13)	0.02 (0, 1145188228303.21)	0.02 (0, 1436377860390.49)	0.02 (0, 1600112518908.68)	0.02 (0, 1659583500268.39)	0.05 (0, 2499529163022.11)	0.04 (0, 2312084056366.8)	0.09 (0, 5677768590870.85)	0.11 (0, 4628957527654.09)	14_Efpeglenatide _low_dosage										0.20 [0.01; 4.16]	
***0 (0, 0.29)**	***0 (0, 0.92)**	***0 (0, 0.38)**	0 (0, 2.07)	***0 (0, 0.58)**	0.13 (0, 35.12)	0.19 (0, 25.34)	0.18 (0, 28.85)	0.2 (0, 30.17)	0.37 (0, 21.38)	0.31 (0, 49.57)	0.59 (0.01, 85.38)	0.73 (0.01, 38.19)	6.79 (0, 16632986170509376)	15_Inject_ semaglutide_ medium_dosage										
***0 (0, 0.18)**	***0 (0, 0.54)**	***0 (0, 0.3)**	0 (0, 1.33)	***0 (0, 0.89)**	0.11 (0, 18.01)	0.17 (0, 12.25)	0.16 (0, 11.37)	0.18 (0, 12.38)	0.33 (0.01, 8.71)	0.27 (0, 25.29)	0.49 (0.01, 43.71)	0.63 (0.02, 14.52)	6.04 (0, 12195342249663256)	0.83 (0.01, 154.88)	16_Inject_ semaglutide_ high_dosage									
***0 (0, 0.18)**	***0 (0, 0.52)**	***0 (0, 0.27)**	0 (0, 1.34)	***0 (0, 0.86)**	0.11 (0, 18.98)	0.17 (0, 12.32)	0.16 (0, 11.31)	0.18 (0, 12.51)	0.32 (0.01, 8.71)	0.26 (0, 26.72)	0.48 (0.01, 42.96)	0.61 (0.02, 14.87)	6.17 (0, 18649227861573440)	0.84 (0, 163.14)	0.98 (0.01, 91.56)	17_Exenatide								
***0 (0, 0.12)**	***0 (0, 0.48)**	***0 (0, 0.19)**	***0 (0, 0.91)**	***0 (0, 0.58)**	0.1 (0, 10.83)	0.15 (0, 6.89)	0.14 (0, 5.82)	0.16 (0, 7.08)	0.28 (0.01, 4.09)	0.23 (0, 13.36)	0.43 (0.01, 19.72)	0.56 (0.02, 6.22)	4.66 (0, 12004104504080320)	0.71 (0, 88.03)	0.87 (0.01, 45.06)	0.89 (0.01, 47.69)	18_Dapagliflozin							
***0 (0, 0.14)**	***0 (0, 0.54)**	***0 (0, 0.2)**	***0 (0, 0.98)**	***0 (0, 0.6)**	0.09 (0, 13.72)	0.11 (0, 10.88)	0.11 (0, 9.68)	0.12 (0, 9.97)	0.23 (0, 7.83)	0.18 (0, 20.43)	0.34 (0, 28.06)	0.45 (0.01, 12.47)	4.09 (0, 12863660541627128)	0.61 (0, 100.51)	0.69 (0, 69.35)	0.7 (0, 71.98)	0.8 (0.01, 70.93)	19_Ertugliflozin_ high_dosage	0.59 [0.07; 5.15]					
***0 (0, 0.06)**	***0 (0, 0.23)**	***0 (0, 0.1)**	***0 (0, 0.5)**	***0 (0, 0.34)**	0.04 (0, 8.58)	0.06 (0, 5.16)	0.06 (0, 5.05)	0.06 (0, 5.61)	0.11 (0, 3.94)	0.09 (0, 11.5)	0.17 (0, 18.86)	0.23 (0, 6.84)	2.09 (0, 7561339130889109)	0.29 (0, 63.45)	0.34 (0, 40.42)	0.37 (0, 41.42)	0.41 (0, 43.5)	0.5 (0.03, 12.24)	20_Ertugliflozin_ low_dosage					
***0 (0, 0.05)**	***0 (0, 0.18)**	***0 (0, 0.09)**	***0 (0, 0.4)**	***0 (0, 0.25)**	0.03 (0, 6.49)	0.05 (0, 3.97)	0.04 (0, 3.67)	0.05 (0, 4.17)	0.09 (0, 3.02)	0.07 (0, 9.28)	0.13 (0, 13.95)	0.17 (0, 5.01)	1.63 (0, 4948528679711714)	0.23 (0, 52.98)	0.26 (0, 29.69)	0.27 (0, 30.54)	0.31 (0, 33.64)	0.39 (0, 83.51)	0.79 (0, 143.86)	21_Liraglutide				
***0 (0, 0)**	***0 (0, 0)**	***0 (0, 0)**	***0 (0, 0)**	***0 (0, 0.01)**	***0 (0, 0.56)**	***0 (0, 0.68)**	***0 (0, 0.7)**	***0 (0, 0.66)**	***0 (0, 0.78)**	0 (0, 1.13)	0 (0, 1.68)	0 (0, 1.58)	0 (0, 2051217010247.24)	0 (0, 5.54)	0 (0, 4.07)	0 (0, 4.1)	0 (0, 3.79)	0 (0, 9.4)	0 (0, 19.35)	0 (0, 20.27)	22_Bexagliflozin			
***0 (0, 0)**	***0 (0, 0)**	***0 (0, 0)**	***0 (0, 0)**	***0 (0, 0.01)**	***0 (0, 0.44)**	***0 (0, 0.52)**	***0 (0, 0.49)**	***0 (0, 0.7)**	***0 (0, 0.72)**	0 (0, 1.01)	0 (0, 1.69)	0 (0, 1.28)	0 (0, 4482603966737.91)	0 (0, 3.85)	0 (0, 3.17)	0 (0, 3.7)	0 (0, 3.35)	0 (0, 7.01)	0 (0, 12.75)	0 (0, 14.67)	1.56 (0, 3868578609925050)	23_Oral_ semaglutide		
***0 (0, 0)**	***0 (0, 0)**	***0 (0, 0)**	***0 (0, 0)**	***0 (0, 0)**	***0 (0, 0.19)**	***0 (0, 0.19)**	***0 (0, 0.18)**	***0 (0, 0.21)**	***0 (0, 0.23)**	***0 (0, 0.29)**	***0 (0, 0.63)**	***0 (0, 0.44)**	***0 (0, 0.55)**	0 (0, 1.73)	0 (0, 1.14)	0 (0, 1.22)	0 (0, 1.43)	0 (0, 2.65)	0 (0, 4.68)	0 (0, 5.73)	0.17 (0, 810625511869478)	0.04 (0, 524603668869.44)	24_Efpeglenatide_ high_dosage	
***0 (0, 0)**	***0 (0, 0)**	***0 (0, 0)**	***0 (0, 0)**	***0 (0, 0)**	***0 (0, 0.08)**	***0 (0, 0.09)**	***0 (0, 0.09)**	***0 (0, 0.11)**	***0 (0, 0.1)**	***0 (0, 0.15)**	***0 (0, 0.23)**	***0 (0, 0.19)**	0 (0, 5340103228.15)	***0 (0, 0.55)**	***0 (0, 0.45)**	***0 (0, 0.56)**	***0 (0, 0.61)**	0 (0, 1.15)	0 (0, 2.22)	0 (0, 2.62)	0.07 (0, 98308616628675744)	0.07 (0, 338459319388.11)	2.03 (0, 77054297409542.6)	25_Lixisenatide

Data presented as RR [95%CIs]. Pairwise (upper-right portion) and network (lower-left portion) meta-analysis results are presented as estimate effect sizes for the outcome of events of hearing loss in subgroup of dosage. Interventions are reported in order of mean

ranking of beneficially prophylactic effect on events of hearing loss in subgroup of dosage, and outcomes are expressed as risk ratio (RR) (95% confidence intervals) (95%CIs). For the pairwise meta-analyses, RR of less than 1 indicates that the treatment specified in the row got more beneficial effect than that specified in the column. For the network meta-analysis (NMA), RR of less than 1 indicates that the treatment specified in the column got more beneficial effect than that specified in the row. Bold results indicate statistical significance.

Table S5C: League table of NMA of primary outcome: incidence of hearing loss (focus on diabetes)

01_Sotagliflozin							0.17 [0.01; 4.10]								
2.47 (0, 2122238777788886016)	02_Albiglutide						0.20 [0.01; 4.16]								
0 (0, 6.44)	0 (0, 3.83)	03_Canagliflozin					0.56 [0.11; 2.90]								
0 (0, 5.06)	0 (0, 2.79)	0.71 (0.01, 46.34)	04_Empagliflozin				0.52 [0.08; 3.58]								
0 (0, 1.99)	0 (0, 1.18)	0.39 (0, 14.68)	0.57 (0, 25.59)	05_Dulaglutide			0.52 [0.12; 2.26]								
0 (0, 3.43)	0 (0, 2.57)	0.34 (0, 56.26)	0.5 (0, 76.68)	0.91 (0.01, 181.47)	06_Inject_ semaglutide		0.71 [0.07; 6.83]								
0 (0, 1.6)	0 (0, 1.09)	0.29 (0, 15.91)	0.4 (0, 28.42)	0.74 (0.01, 75.37)	0.81 (0, 211.61)	07_Dapagliflozin	1.00 [0.20; 4.95]								
0 (0, 1.02)	*0 (0, 0.61)	0.29 (0.01, 3.63)	0.4 (0.01, 7.63)	0.72 (0.05, 17.62)	0.8 (0.01, 84.67)	0.98 (0.04, 28.28)	08_Placebo_ or_Control	0.66 [0.11; 3.98]	0.66 [0.10; 4.21]	0.25 [0.03; 2.23]	0.98 [0.04; 25.20]	0.34 [0.01; 8.30]	0.60 [0.03; 12.47]	0.40 [0.02; 8.35]	0.11 [0.01; 2.06]
0 (0, 1.03)	*0 (0, 0.7)	0.18 (0, 11.01)	0.25 (0, 19.16)	0.44 (0.01, 48.93)	0.51 (0, 157.23)	0.61 (0.01, 69.4)	0.62 (0.02, 17.93)	09_Exenatide							
*0 (0, 0.5)	*0 (0, 0.28)	0.08 (0, 4.22)	0.11 (0, 7.75)	0.2 (0, 15.84)	0.22 (0, 51)	0.28 (0, 23.81)	0.29 (0, 5.96)	0.45 (0, 44.01)	10_Ertugliflozin						
*0 (0, 0.3)	*0 (0, 0.2)	0.05 (0, 3.64)	0.07 (0, 6.66)	0.12 (0, 16.62)	0.14 (0, 41.73)	0.17 (0, 22.71)	0.18 (0, 6.29)	0.29 (0, 40.06)	0.62 (0, 141.36)	11_Liraglutide					
*0 (0, 0.01)	*0 (0, 0)	0 (0, 1.03)	0 (0, 1.58)	0 (0, 3.48)	0 (0, 6.32)	0 (0, 4.26)	0 (0, 3.11)	0 (0, 7.88)	0 (0, 20.34)	0 (0, 39.76)	12_Tirzepatide				
*0 (0, 0.01)	*0 (0, 0)	*0 (0, 0.72)	0 (0, 1)	0 (0, 1.76)	0 (0, 2.44)	0 (0, 2.26)	0 (0, 1.43)	0 (0, 4.04)	0 (0, 12.63)	0 (0, 21.29)	16.52 (0, 1.23048545652618e+22)	13_Bexagliflozin			
*0 (0, 0.01)	*0 (0, 0.01)	*0 (0, 0.81)	0 (0, 1.37)	0 (0, 2.48)	0 (0, 5.56)	0 (0, 3.74)	0 (0, 2.01)	0 (0, 6.49)	0 (0, 16.82)	0 (0, 30.91)	504.83 (0, 6.57108414695688e+23)	12.2 (0, 3.3841999950902e+20)	14_Oral_ semaglutide		
*0 (0, 0)	*0 (0, 0)	*0 (0, 0.34)	*0 (0, 0.62)	0 (0, 1.19)	0 (0, 1.92)	0 (0, 1.52)	0 (0, 1.01)	0 (0, 3.05)	0 (0, 6.74)	0 (0, 11.31)	3.97 (0, 371174953816719040)	0.1 (0, 55278842106634696)	0.01 (0, 360132055918163)	15_Efpeglenatide	
*0 (0, 0)	*0 (0, 0)	*0 (0, 0.13)	*0 (0, 0.23)	*0 (0, 0.45)	*0 (0, 0.91)	*0 (0, 0.7)	*0 (0, 0.33)	0 (0, 1.06)	0 (0, 2.72)	0 (0, 6.23)	62.03 (0, 9.52706471994347e+22)	9.21 (0, 380186840537805696)	0.39 (0, 45183755699111.8)	16.84 (0, 1.78905299487425e+22)	16_Lixisenatide

Data presented as OR [95%CIs]. Pairwise (upper-right portion) and network (lower-left portion) meta-analysis results are presented

as estimate effect sizes for the outcome of overall events of hearing loss. Interventions are reported in order of mean ranking of beneficially prophylactic effect on overall events of hearing loss, and outcomes are expressed as odds ratio (OR) (95% confidence intervals) (95%CIs). For the pairwise meta-analyses, OR of less than 1 indicates that the treatment specified in the row got more beneficial effect than that specified in the column. For the network meta-analysis (NMA), OR of less than 1 indicates that the treatment specified in the column got more beneficial effect than that specified in the row. Bold results indicate statistical significance.

Table S5D: League table of NMA of primary outcome: incidence of hearing loss in subgroup of dosage (focus on diabetes)

01_Sotagliflozin												0.17 [0.01; 4.10]											
2.09 (0, 318679639578509952)	02_Dulaglutide_ low_dosage									0.33 [0.01; 8.22]													
65.83 (0, 302947997740808192)	106.2 (0, 215981963803103744)	03_Albiglutide										0.20 [0.01; 4.16]											
0.29 (0, 1417258178379.72)	0.09 (0, 1309410154737.98)	0.01 (0, 62990208813.5)	04_Inject_ semaglutide_ low_dosage									0.66 [0.03; 16.34]	0.34 [0.01; 8.27]										
0 (0, 8.54)	0 (0, 24.38)	0 (0, 3.36)	0 (0, 8.28)	05_Empagliflozin_ high_dosage		1.00 [0.06; 15.98]						0.62 [0.08; 5.07]											
0 (0, 9.14)	0 (0, 24.6)	0 (0, 3.29)	0 (0, 8.16)	1.05 (0, 273.95)	06_Canagliflozin_ low_dosage		1.00 [0.06; 15.96]					0.62 [0.08; 5.07]											
0 (0, 8.74)	0 (0, 25.44)	0 (0, 3.31)	0 (0, 8.28)	0.99 (0.01, 111.77)	0.98 (0, 266.22)	07_Empagliflozin_ low_dosage						0.62 [0.08; 5.07]											
0 (0, 8.39)	0 (0, 27.99)	0 (0, 2.74)	0 (0, 7.6)	0.99 (0, 231.81)	0.92 (0.01, 93.57)	0.98 (0, 196.22)	08_Canagliflozin_ high_dosage					0.62 [0.08; 5.08]											
0 (0, 10997731.54)	0 (0, 2726844.52)	0 (0, 56022.22)	0 (0, 1467276.61)	0.01 (0, 20854927203.95)	0.01 (0, 27245205919.07)	0.01 (0, 26482439363.25)	0.01 (0, 32809840139.01)	09_Tirzepatide_ low_dosage										0.33 [0.01; 8.14]	0.34 [0.01; 8.48]				
0 (0, 1.69)	0 (0, 1.41)	*0 (0, 0.45)	0 (0, 1.22)	0.23 (0, 19.87)	0.23 (0, 19.66)	0.22 (0, 20.44)	0.24 (0, 19.15)	22.42 (0, 35028063296017367040)	10_Dulaglutide_ high_dosage			0.61 [0.14; 2.61]											
0 (0, 2737247323.15)	0 (0, 166467197801.65)	0 (0, 171968606.94)	0 (0, 53345228827.14)	0.7 (0, 105754474223745)	0.73 (0, 92620609897793.7)	0.74 (0, 80144434371706.5)	0.8 (0, 95569160614378.1)	82.4 (0, 2.24545299015127e+25)	3.49 (0, 453718005125628)	11_Efpeglenatide_ low_dosage												0.20 [0.01; 4.16]	
0 (0, 1.49)	0 (0, 4.24)	*0 (0, 0.54)	0 (0, 1.45)	0.23 (0, 31.5)	0.22 (0, 32.18)	0.22 (0, 29.31)	0.23 (0, 31.46)	24.65 (0, 14929837176839278592)	0.96 (0.01, 152.33)	0.29 (0, 646672983743489)	12_Dapagliflozin	1.00 [0.20; 4.96]											
*0 (0, 0.93)	0 (0, 2.47)	*0 (0, 0.32)	*0 (0, 0.84)	0.24 (0, 6.9)	0.23 (0, 6.7)	0.23 (0, 7.02)	0.24 (0, 6.65)	24.43 (0, 20583156940281937920)	0.93 (0.05, 30.09)	0.3 (0, 489205959767217)	1.01 (0.03, 40.25)	13_Placebo_ or_Control	0.71 [0.07; 6.80]	0.66 [0.11; 3.98]	0.99 [0.10; 9.54]	0.43 [0.06; 2.90]	0.25 [0.03; 2.23]		0.33 [0.01; 8.20]	0.60 [0.03; 12.47]	0.34 [0.01; 8.30]	0.20 [0.01; 4.17]	0.11 [0.01; 2.06]
0 (0, 1.44)	0 (0, 3.9)	*0 (0, 0.44)	*0 (0, 0.46)	0.15 (0, 45.97)	0.14 (0, 41.43)	0.15 (0, 43.35)	0.16 (0, 42.71)	15.87 (0, 1.9744437178517e+19)	0.66 (0, 199.75)	0.19 (0, 365168461432397)	0.7 (0, 237.63)	0.68 (0.01, 64.09)	14_Inject_ semaglutide_ medium_dosage										
*0 (0, 0.99)	0 (0, 2.79)	*0 (0, 0.3)	*0 (0, 0.83)	0.14 (0, 19.75)	0.13 (0, 20.3)	0.13 (0, 19.46)	0.14 (0, 20.4)	14.9 (0, 10643265465437739008)	0.57 (0.01, 100.64)	0.17 (0, 348448061826236)	0.61 (0, 112.79)	0.6 (0.01, 25.47)	0.88 (0, 275.85)	15_Exenatide									
*0 (0, 0.65)	0 (0, 1.89)	*0 (0, 0.21)	*0 (0, 0.65)	0.08 (0, 13.69)	0.08 (0, 11.73)	0.08 (0, 13.62)	0.09 (0, 12.99)	8.46 (0, 5334059590660868096)	0.37 (0, 55.76)	0.1 (0, 286149237192867)	0.39 (0, 65.45)	0.39 (0.01, 14.26)	0.54 (0, 148.65)	0.65 (0, 120.36)	16_Ertugliflozin_ high_dosage	0.58 [0.07; 5.15]							
*0 (0, 0.37)	0 (0, 1.21)	*0 (0, 0.13)	*0 (0, 0.39)	0.05 (0, 8.13)	0.05 (0, 8.23)	0.05 (0, 8.15)	0.05 (0, 7.92)	4.96 (0, 4274140862899236352)	0.21 (0, 39.86)	0.06 (0, 184430299998978)	0.22 (0, 46.23)	0.23 (0, 10.26)	0.33 (0, 110.24)	0.37 (0, 89.15)	0.6 (0.02, 22.45)	17_Ertugliflozin_ low_dosage							
*0 (0, 0.3)	*0 (0, 0.8)	*0 (0, 0.1)	*0 (0, 0.34)	0.04 (0, 6.2)	0.04 (0, 6.64)	0.04 (0, 6.18)	0.04 (0, 6.64)	4.07 (0, 1996938509722690560)	0.17 (0, 32.31)	0.04 (0, 129932193776580)	0.18 (0, 37.19)	0.18 (0, 8.22)	0.25 (0, 88.54)	0.3 (0, 67.85)	0.46 (0, 150.11)	0.79 (0, 217.5)	18_Liraglutide						
*0 (0, 0)	*0 (0, 0.02)	*0 (0, 0)	*0 (0, 0.01)	0 (0, 1.41)	0 (0, 1.41)	0 (0, 1.38)	0 (0, 1.52)	*0 (0, 0.64)	0 (0, 6.41)	0 (0, 34409584014.07)	0 (0, 7.16)	0 (0, 3.98)	0 (0, 11.74)	0 (0, 11.77)	0 (0, 23.5)	0 (0, 38.2)	0 (0, 40.99)	19_Tirzepatide_ medium_dosage	1.01 [0.10; 9.75]				
*0 (0, 0)	*0 (0, 0.01)	*0 (0, 0)	*0 (0, 0.01)	*0 (0, 0.69)	*0 (0, 0.75)	*0 (0, 0.7)	*0 (0, 0.83)	*0 (0, 0.59)	0 (0, 3.12)	0 (0, 37919455953.32)	0 (0, 3.33)	0 (0, 1.88)	0 (0, 7.02)	0 (0, 5.9)	0 (0, 12.05)	0 (0, 19.93)	0 (0, 21.89)	1.09 (0.01, 90.9)	20_Tirzepatide_ high_dosage				
*0 (0, 0.02)	*0 (0, 0.01)	*0 (0, 0)	*0 (0, 0)	*0 (0, 0.92)	*0 (0, 0.68)	*0 (0, 0.86)	0 (0, 1.03)	0 (0, 94704164398439.3)	0 (0, 1.97)	0 (0, 15565378709.97)	0 (0, 3.19)	0 (0, 1.72)	0 (0, 5.53)	0 (0, 4.7)	0 (0, 8.87)	0 (0, 13.05)	0 (0, 21.55)	1.19 (0, 1223191932944810752)	1.14 (0, 642943096931709824)	21_Oral_ semaglutide			
*0 (0, 0.01)	*0 (0, 0.01)	*0 (0, 0)	*0 (0, 0.01)	*0 (0, 0.89)	*0 (0, 0.82)	*0 (0, 0.82)	*0 (0, 0.97)	0 (0, 67407871957541.6)	0 (0, 3.85)	0 (0, 15186541816.36)	0 (0, 4.32)	0 (0, 2.19)	0 (0, 10.13)	0 (0, 7.58)	0 (0, 15.93)	0 (0, 25.98)	0 (0, 33.27)	4.45 (0, 1215235608392165888)	4.18 (0, 1638494836222807552)	26.5 (0, 553157232212981056)	22_Bexagliflozin		
*0 (0, 0)	*0 (0, 0)	*0 (0, 0)	*0 (0, 0)	*0 (0, 0.25)	*0 (0, 0.2)	*0 (0, 0.3)	*0 (0, 0.23)	0 (0, 1661383496346434)	0 (0, 1.02)	*0 (0, 0.56)	0 (0, 1.07)	*0 (0, 0.53)	0 (0, 2.23)	0 (0, 1.9)	0 (0, 3.22)	0 (0, 5.26)	0 (0, 8.68)	1.01 (0, 24523824230707306496)	0.8 (0, 22996670333404631040)	1.41 (0, 1216365062732060928)	0.26 (0, 4919770682926172)	23_Efpeglenatide_ high_dosage	
*0 (0, 0)	*0 (0, 0)	*0 (0, 0)	*0 (0, 0)	*0 (0, 0.1)	*0 (0, 0.09)	*0 (0, 0.09)	*0 (0, 0.11)	0 (0, 41541388548403.3)	*0 (0, 0.41)	0 (0, 854185744.85)	*0 (0, 0.46)	*0 (0, 0.23)	*0 (0, 0.92)	*0 (0, 0.81)	0 (0, 1.26)	0 (0, 2.24)	0 (0, 3.22)	0.51 (0, 3175620563482272 64)	0.51 (0, 2419534646189748 48)	0.52 (0, 6319610393409708 80)	0.05 (0, 76392667219863.6)	0.2 (0, 66098980421613.5)	24_Lixisenatide

Data presented as OR [95%CIs]. Pairwise (upper-right portion) and network (lower-left portion) meta-analysis results are presented as estimate effect sizes for the outcome of events of hearing loss in subgroup of dosage. Interventions are reported in order of mean ranking of beneficially prophylactic effect on events of hearing loss in subgroup of dosage, and outcomes are expressed as odds ratio (OR) (95% confidence intervals) (95%CIs). For the pairwise meta-analyses, OR of less than 1 indicates that the treatment specified in the row got more beneficial effect than that specified in the column. For the network meta-analysis (NMA), OR of less than 1 indicates that the treatment specified in the column got more beneficial effect than that specified in the row. Bold results indicate statistical significance.

Table S5E: League table of NMA of drop-out rate

01_Bexagliflozin											0.43 [0.14; 1.33]				
0.57 (0.13, 2.25)	02_Albiglutide										0.71 [0.48; 1.06]				
0.56 (0.13, 2.12)	0.98 (0.42, 2.3)	03_Sotagliflozin									0.73 [0.47; 1.13]				
0.53 (0.12, 1.95)	0.92 (0.39, 2.1)	0.94 (0.43, 2.02)	04_Dapagliflozin								0.77 [0.54; 1.11]				
0.52 (0.12, 1.8)	0.9 (0.41, 1.87)	0.92 (0.45, 1.81)	0.98 (0.49, 1.92)	05_Canagliflozin							0.77 [0.58; 1.02]				
0.5 (0.12, 1.82)	0.86 (0.4, 1.98)	0.88 (0.43, 1.89)	0.94 (0.47, 2.03)	0.95 (0.54, 1.94)	06_Ertugliflozin						0.84 [0.63; 1.13]				
0.48 (0.11, 1.85)	0.83 (0.34, 2.01)	0.85 (0.37, 1.95)	0.9 (0.4, 2.08)	0.91 (0.45, 2)	0.96 (0.42, 2.03)	07_Exenatide					0.86 [0.61; 1.23]				
0.47 (0.11, 1.8)	0.82 (0.33, 2.01)	0.84 (0.36, 1.95)	0.89 (0.39, 2.07)	0.91 (0.44, 2.01)	0.95 (0.41, 2.02)	0.99 (0.4, 2.38)	08_Liraglutide				0.87 [0.59; 1.28]				
0.45 (0.11, 1.54)	0.79 (0.37, 1.55)	0.81 (0.42, 1.49)	0.86 (0.45, 1.58)	0.88 (0.53, 1.47)	0.92 (0.49, 1.52)	0.96 (0.46, 1.84)	0.97 (0.46, 1.89)	09_Empagliflozin			0.88 [0.74; 1.04]				
0.43 (0.09, 1.68)	0.75 (0.3, 1.88)	0.77 (0.32, 1.81)	0.81 (0.35, 1.94)	0.83 (0.4, 1.85)	0.87 (0.38, 1.88)	0.91 (0.37, 2.21)	0.91 (0.37, 2.28)	0.94 (0.48, 2.01)	10_Lixisenatide		0.95 [0.63; 1.44]				
0.42 (0.09, 1.61)	0.73 (0.3, 1.77)	0.75 (0.32, 1.73)	0.8 (0.35, 1.85)	0.81 (0.4, 1.77)	0.85 (0.37, 1.79)	0.88 (0.37, 2.13)	0.89 (0.36, 2.18)	0.92 (0.48, 1.9)	0.97 (0.4, 2.41)	11_Efpeglenatide	0.98 [0.69; 1.39]				
0.41 (0.1, 1.36)	0.71 (0.37, 1.35)	0.73 (0.41, 1.28)	0.78 (0.46, 1.36)	0.79 (0.54, 1.24)	0.83 (0.5, 1.29)	0.86 (0.46, 1.6)	0.87 (0.46, 1.65)	0.9 (0.69, 1.26)	0.95 (0.5, 1.83)	0.98 (0.52, 1.81)	12_Placebo_ or_Control	1.02 [0.77; 1.35]	0.93 [0.60; 1.45]	0.68 [0.38; 1.21]	0.52 [0.28; 0.96]
0.4 (0.09, 1.39)	0.69 (0.31, 1.42)	0.71 (0.34, 1.38)	0.76 (0.37, 1.47)	0.76 (0.43, 1.37)	0.8 (0.39, 1.41)	0.84 (0.38, 1.69)	0.85 (0.38, 1.74)	0.87 (0.52, 1.44)	0.93 (0.41, 1.92)	0.95 (0.43, 1.92)	0.97 (0.61, 1.42)	13_Inject_ semaglutide			
0.38 (0.09, 1.43)	0.67 (0.29, 1.57)	0.68 (0.31, 1.52)	0.73 (0.34, 1.62)	0.74 (0.38, 1.54)	0.77 (0.36, 1.57)	0.81 (0.35, 1.86)	0.82 (0.35, 1.9)	0.84 (0.46, 1.65)	0.89 (0.38, 2.12)	0.91 (0.4, 2.12)	0.93 (0.53, 1.64)	0.96 (0.49, 2.03)	14_Dulaglutide		
0.26 (0.06, 1.04)	0.46 (0.17, 1.13)	0.47 (0.19, 1.11)	0.5 (0.21, 1.16)	0.51 (0.23, 1.12)	0.53 (0.22, 1.16)	0.55 (0.21, 1.33)	0.56 (0.22, 1.37)	0.58 (0.28, 1.2)	0.61 (0.23, 1.51)	0.63 (0.24, 1.52)	0.64 (0.32, 1.23)	0.66 (0.3, 1.47)	0.68 (0.28, 1.63)	15_Tirzepatide	
**0.21 (0.04, 0.87)**	**0.36 (0.13, 0.99)**	**0.37 (0.14, 0.96)**	0.39 (0.15, 1.02)	**0.4 (0.16, 0.98)**	0.41 (0.16, 1)	0.43 (0.16, 1.17)	0.44 (0.16, 1.19)	0.45 (0.2, 1.07)	0.48 (0.17, 1.32)	0.49 (0.18, 1.32)	0.5 (0.23, 1.09)	0.52 (0.21, 1.3)	0.54 (0.2, 1.4)	0.79 (0.28, 2.24)	16_Oral_ semaglutide

Data presented as OR [95%CIs]. Pairwise (upper-right portion) and network (lower-left portion) meta-analysis results are presented as estimate effect sizes for the outcome of safety profile (drop-out rate). Interventions are reported in order of mean ranking of safety, and outcomes are expressed as odds ratio (OR) (95%

confidence intervals) (95%CIs). For the pairwise meta-analyses, OR of less than 1 indicates that the treatment specified in the row got more safety than that specified in the column. For the network meta-analysis (NMA), OR of less than 1 indicates that the treatment specified in the column got more safety than that specified in the row. Bold results indicate statistical significance.

Abbreviation: 95%CIs: 95% confidence intervals; GLP-1 agonist: glucagon-like peptide-1 agonist; NMA: network meta-analysis; OR: odds ratio; RCT: randomized controlled trial; RR: risk ratio; SGLT2 inhibitor: sodium–glucose cotransporter 2 inhibitor

Table S6A: SUCRA (Surface under the cumulative ranking) of primary outcome: incidence of hearing loss

Treatment	Rank 1	Rank 2	Rank 3	Rank 4	Rank 5	Rank 6	Rank 7	Rank 8	Rank 9	Rank 10	Rank 11	Rank 12	Rank 13	Rank 14	Rank 15	Rank 16	SUCRA
Sotagliflozin	0.6454375	0.3293375	0.0103375	0.0040375	0.0024875	0.001675	0.001125	0.00095	0.0011625	0.00105	0.0010875	0.000825	0.00045	2.50E-05	1.25E-05	0	97.077
Albiglutide	0.3522875	0.6012875	0.020725	0.0085375	0.005025	0.0029375	0.0022875	0.00195	0.0018375	0.00155	0.0009875	0.00055	2.50E-05	1.25E-05	0	0	94.82966667
Canagliflozin	0.001	0.0221375	0.2966375	0.2352	0.149025	0.0904625	0.0619875	0.04875	0.04075	0.0305125	0.0175375	0.0055125	0.0004875	0	0	0	73.83283333
Empagliflozin	5.00E-05	0.002425	0.0680625	0.176325	0.251725	0.2257625	0.1398875	0.070575	0.0415	0.0179625	0.0049	0.00075	7.50E-05	0	0	0	68.744
Tirzepatide	0.0008625	0.02955	0.3179625	0.1369375	0.0870125	0.0647625	0.0542125	0.0548	0.0627875	0.06435	0.06385	0.0495125	0.012275	0.00105	7.50E-05	0	67.44258333
Dulaglutide	8.75E-05	0.006875	0.1509	0.1905125	0.1563125	0.123825	0.096575	0.0792625	0.0739625	0.062075	0.0381125	0.0176875	0.0034875	0.000325	0	0	66.45991667
Placebo_or_Control	0	1.25E-05	5.00E-04	0.007	0.044175	0.1577125	0.289275	0.2901	0.157725	0.0461	0.00685	0.00055	0	0	0	0	56.63416667
Inject_semaglutide	3.75E-05	0.0023625	0.0344	0.0703625	0.0880625	0.09625	0.10255	0.1219375	0.149925	0.1563625	0.10485	0.06415	0.008275	0.000475	0	0	52.994
Exenatide	0.00015	0.0029125	0.0423	0.069925	0.073925	0.0717	0.0725875	0.0950625	0.131225	0.152975	0.1664375	0.0989875	0.0198875	0.001825	1.00E-04	0	50.02641667
Dapagliflozin	1.25E-05	0.001325	0.0261875	0.051225	0.0758375	0.0872375	0.0889625	0.10795	0.143525	0.1696375	0.1480375	0.0843375	0.014625	0.001025	6.25E-05	1.25E-05	49.93833333
Ertugliflozin	2.50E-05	0.000925	0.01415	0.025175	0.036875	0.043475	0.049425	0.0765875	0.1018875	0.142475	0.201475	0.240675	0.0600875	0.0063125	0.00045	0	40.85833333
Liraglutide	3.75E-05	5.00E-04	0.007275	0.015775	0.0208875	0.0255125	0.031075	0.0414125	0.077525	0.129025	0.1989125	0.3503375	0.0863375	0.0146625	0.000675	5.00E-05	35.927
Oral_semaglutide	0	1.25E-05	0.002825	0.003225	0.0028375	0.0026125	0.003025	0.0028875	0.0050875	0.006975	0.0129875	0.0271375	0.239475	0.29325	0.2569375	0.140725	13.30716667
Bexagliflozin	0	0.00015	0.00335	0.0028375	0.0026875	0.002525	0.0030875	0.0034625	0.0048	0.008225	0.0121	0.0206125	0.2006	0.2427375	0.2602125	0.2326125	11.75591667
Efpeglenatide	1.25E-05	0.0001875	0.0041375	0.0026125	0.00275	0.00305	0.003075	0.0032375	0.004575	0.0076125	0.0143875	0.0236875	0.1861375	0.2043	0.2424125	0.297825	11.04058333
Lixisenatide	0	0	0.00025	0.0003125	0.000375	5.00E-04	0.0008625	0.001075	0.001725	0.0031125	0.0074875	0.0146875	0.167775	0.234	0.2390625	0.328775	9.132083333

Table S6B: SUCRA (Surface under the cumulative ranking) of primary outcome: incidence of hearing loss in subgroup of dosage

Treatment	Rank 1	Rank 2	Rank 3	Rank 4	Rank 5	Rank 6	Rank 7	Rank 8	Rank 9	Rank 10	Rank 11	Rank 12	Rank 13	Rank 14	Rank 15	Rank 16	Rank 17	Rank 18	Rank 19	Rank 20	Rank 21	Rank 22	Rank 23	Rank 24	Rank 25	SUCRA
Tirzepatide_low_dosage	0.1702	0.250675	0.20615	0.176575	0.1179875	0.0498	0.0122375	0.0047875	0.0032875	0.002025	0.001125	0.001075	0.000875	0.000725	0.0006875	0.0005625	0.0004375	0.0003375	0.000225	0.0001375	6.25E-05	2.50E-05	0	0	0	91.05270833
Sotagliflozin	0.2599375	0.1847375	0.1555875	0.15985	0.139925	0.043825	0.0148875	0.008575	0.0060125	0.004725	0.0035375	0.00305	0.002425	0.0016625	0.0021	0.0020375	0.00215	0.0018	0.0013	0.0013375	0.0004625	6.25E-05	1.25E-05	0	0	90.42286458
Albiglutide	0.1932125	0.224075	0.1906625	0.15075	0.134625	0.0401	0.0203125	0.0123875	0.00805	0.00595	0.00365	0.0030125	0.0029375	0.0022	0.001725	0.0015875	0.0016125	0.0012625	0.000825	0.0007375	0.0002125	6.25E-05	3.75E-05	1.25E-05	0	90.05234375
Dulaglutide_low_dosage	0.2040875	0.1678625	0.189475	0.1730375	0.1289375	0.0553125	0.0182375	0.0128125	0.007975	0.006075	0.0046625	0.0043875	0.00305	0.0032375	0.002725	0.0030625	0.003125	0.0028375	0.0033125	0.003	0.002225	0.000475	8.75E-05	0	0	88.73020833
Inject_semaglutide_low_dosage	0.0893125	0.114175	0.1899375	0.2327125	0.1885125	0.074875	0.02785	0.01995	0.0126	0.0111	0.00735	0.00585	0.004525	0.0045625	0.0043625	0.0036875	0.003125	0.001925	0.0016	0.001425	5.00E-04	6.25E-05	0	0	0	86.0590625
Tirzepatide_medium_dosage	0	0	0.00195	0.0186375	0.0589125	0.176175	0.1588125	0.1171625	0.08905	0.0687125	0.0530125	0.043825	0.0392875	0.0316	0.0307	0.0277875	0.0253375	0.0229625	0.017825	0.0114125	0.005575	0.0011875	7.50E-05	0	0	64.61348958
Canagliflozin_high_dosage	0	0.0001125	0.000925	0.008475	0.0394375	0.1043375	0.1474	0.1289	0.104525	0.0873125	0.0769125	0.056975	0.047575	0.0411875	0.0383	0.033875	0.0324875	0.023075	0.0166	0.0078375	0.00305	0.0006625	3.75E-05	0	0	62.0934375
Canagliflozin_low_dosage	0	2.50E-05	0.0014125	0.0067625	0.0361875	0.1097875	0.131925	0.1367875	0.110125	0.08835	0.0712625	0.0551625	0.0466875	0.042	0.043875	0.0379375	0.0301	0.0227	0.0167625	0.0081625	0.003375	6.00E-04	1.25E-05	0	0	61.85125
Empagliflozin_high_dosage	0	0	0.0006125	0.006625	0.037025	0.1062375	0.12305	0.113725	0.10995	0.089775	0.0744875	0.0642125	0.05215	0.043025	0.0425625	0.0420625	0.038	0.027575	0.0160375	0.00895	0.0034875	0.000425	2.50E-05	0	0	60.83244792
Empagliflozin_low_dosage	0	0	0	2.00E-04	0.0019375	0.0125375	0.037875	0.0762375	0.115325	0.1501625	0.158525	0.146625	0.11775	0.0773375	0.0496875	0.0287	0.015625	0.007875	0.0026625	0.000825	1.00E-04	1.25E-05	0	0	0	57.09005208
Tirzepatide_high_dosage	0	0	0.00025	0.00465	0.0191625	0.0626	0.103525	0.1049125	0.1054	0.0938375	0.079825	0.06445	0.0523625	0.050975	0.05175	0.0484875	0.046675	0.0414	0.0325875	0.023925	0.0113	0.00165	0.0002625	1.25E-05	0	56.43385417
Dulaglutide_high_dosage	0	0	0	0.0004625	0.0043125	0.0206375	0.045175	0.0702125	0.08145	0.0883375	0.0928625	0.0954125	0.082225	0.0782125	0.0748125	0.07235	0.0636875	0.0515875	0.0386125	0.02495	0.011375	0.002875	0.000425	2.50E-05	0	50.79953125
Placebo_or_Control	0	0	0	0	0	3.75E-05	0.000125	0.001325	0.0063125	0.0222375	0.0624	0.1248	0.1943	0.224875	0.1863125	0.1119	0.04865	0.0133125	0.002975	0.0003625	7.50E-05	0	0	0	0	46.39104167
Efpeglenatide_low_dosage	0.08325	0.0583375	0.0628875	0.0578875	0.0690375	0.058225	0.0131875	0.0091875	0.0064875	0.005875	0.0052125	0.0041	0.0040625	0.0037	0.0040625	0.0051125	0.005725	0.007425	0.008975	0.0138625	0.1279625	0.131525	0.148725	0.102575	0.0026125	45.17161458
Inject_semaglutide_medium_dosage	0	0	3.75E-05	0.001525	0.013475	0.0338625	0.05305	0.0458125	0.0483875	0.0551875	0.0519375	0.050175	0.0497875	0.0525	0.0609625	0.0700875	0.0810375	0.0803125	0.0858375	0.1021875	0.04625	0.0154875	0.0019625	0.0001375	0	43.95083333
Inject_semaglutide_high_dosage	0	0	0	0.0005375	0.0028875	0.0137125	0.023925	0.0332375	0.0433875	0.05095	0.056875	0.062675	0.0617375	0.06705	0.0773625	0.091425	0.097825	0.10055	0.091625	0.0732625	0.0400125	0.0095625	0.0013125	8.75E-05	0	41.93145833

Exenatide	0	0	7.50E-05	0.00065	0.0029125	0.0122625	0.02245	0.0325875	0.041575	0.0473375	0.0512625	0.0529	0.0620375	0.0666125	0.07425	0.0925875	0.1008	0.1076875	0.098	0.0803125	0.0421875	0.0104875	0.001	2.50E-05	0	41.04270833
Dapagliflozin	0	0	0	1.00E-04	0.0007875	0.0049125	0.0118	0.02075	0.0299375	0.0395	0.05095	0.0551625	0.062375	0.072625	0.0910875	0.1052625	0.115625	0.1210875	0.0954625	0.0749125	0.0389875	0.007675	0.000925	7.50E-05	0	39.51963542
Ertugliflozin_high_dosage	0	0	0	0.000175	0.0020125	0.0114625	0.0166625	0.025675	0.036275	0.041275	0.04345	0.0470875	0.0478375	0.053025	0.0643375	0.085725	0.098275	0.111025	0.1323	0.1117875	0.05665	0.013375	0.0015125	7.50E-05	0	38.23432292
Ertugliflozin_low_dosage	0	0	0	1.00E-04	0.0007125	0.0036375	0.0078375	0.011725	0.0166125	0.020225	0.025125	0.0288	0.031975	0.0400875	0.0452125	0.060475	0.08635	0.11215	0.15835	0.1919625	0.121325	0.0319625	0.0047375	0.0006375	0	31.33046875
Liraglutide	0	0	2.50E-05	0.0001375	0.00085	0.0038125	0.0068	0.009375	0.012975	0.0162875	0.020175	0.0242625	0.027175	0.0349375	0.043	0.0617125	0.082425	0.1161125	0.1431125	0.206225	0.135575	0.0498	0.0048	0.000425	0	29.93317708
Bexagliflozin	0	0	1.25E-05	1.00E-04	2.00E-04	0.0011875	0.0014	0.0018125	0.0017125	0.001725	0.00185	0.0023625	0.0025375	0.00265	0.0035375	0.0044875	0.0060375	0.0087875	0.010425	0.015625	0.10495	0.2384375	0.2125125	0.1849	0.19275	9.690520833
Oral_semaglutide	0	0	0	3.75E-05	0.00015	0.00055	0.0009375	0.001425	0.00165	0.00185	0.0020625	0.00185	0.0020375	0.0027	0.003225	0.0039625	0.006025	0.00805	0.01115	0.01425	0.1097125	0.205375	0.2247625	0.2189125	0.179325	9.377447917
Efpeglenatide_high_dosage	0	0	0	0	1.25E-05	5.00E-05	0.0004125	0.0004375	0.00065	0.00085	0.001125	0.00125	0.0017125	0.0016375	0.00225	0.0033875	0.0065875	0.004825	0.0068875	0.0093625	0.0199	0.15925	0.233175	0.28795	0.2582875	6.8709375
Lixisenatide	0	0	0	1.25E-05	0	6.25E-05	0.000125	2.00E-04	0.0002875	0.0003375	0.0003625	0.0005375	0.000575	0.000875	0.0011125	0.0017375	0.002275	0.0033375	0.00655	0.0131875	0.1146875	0.1189625	0.1636	0.20415	0.367025	6.524583333

Table S6C: SUCRA (Surface under the cumulative ranking) of drop-out rate

Treatment	Rank 1	Rank 2	Rank 3	Rank 4	Rank 5	Rank 6	Rank 7	Rank 8	Rank 9	Rank 10	Rank 11	Rank 12	Rank 13	Rank 14	Rank 15	Rank 16	SUCRA
Bexagliflozin	0.6482375	0.0893125	0.050675	0.0346875	0.02805	0.0209375	0.0165	0.014025	0.0134	0.0113	0.011075	0.0129625	0.0142625	0.017125	0.0118875	0.0055625	87.90225
Albiglutide	0.0950375	0.206725	0.1473875	0.1110375	0.0882	0.0706125	0.0565375	0.0460875	0.037625	0.0300375	0.0280875	0.026425	0.0233625	0.0192625	0.0097375	0.0038375	72.86766667
Sotagliflozin	0.0789875	0.18405	0.141925	0.1145	0.09365	0.0779625	0.06335	0.05155	0.042225	0.0345875	0.0302125	0.029275	0.0268	0.0201375	0.0086	0.0021875	71.156
Dapagliflozin	0.0427375	0.120275	0.1277125	0.118225	0.1068625	0.0916375	0.0804125	0.064375	0.0528875	0.0459	0.0392125	0.03775	0.0336375	0.0249875	0.010725	0.0026625	65.92491667
Canagliflozin	0.022325	0.0859875	0.122525	0.1334375	0.1245125	0.1113125	0.09525	0.0789125	0.061375	0.0485375	0.038275	0.0326625	0.02415	0.0146625	0.0050625	0.0010125	65.692
Ertugliflozin	0.0206625	0.066875	0.093525	0.1036	0.10945	0.1078	0.1008625	0.089725	0.077125	0.061575	0.0518375	0.0454875	0.0378875	0.024125	0.00805	0.0014125	60.88766667
Exenatide	0.0256125	0.0673125	0.0792	0.0857125	0.0866875	0.0852	0.0822125	0.0768625	0.0714375	0.0616375	0.060275	0.06265	0.0645625	0.0545625	0.0275625	0.0085125	55.3155
Liraglutide	0.0281125	0.072075	0.078825	0.0836	0.0842125	0.08165	0.077175	0.072075	0.064775	0.059725	0.0589	0.0632125	0.0696625	0.06315	0.0322375	0.0106125	54.66216667
Empagliflozin	0.001125	0.00655	0.0209	0.043325	0.07705	0.1139125	0.1408125	0.1489	0.1324375	0.1074	0.080775	0.0601125	0.040375	0.01975	0.0056875	0.0008875	51.51641667
Lixisenatide	0.0180625	0.0447	0.053475	0.0601	0.06175	0.06575	0.066775	0.065725	0.0667875	0.0651125	0.06655	0.0799625	0.1	0.10665	0.0599	0.0187	45.7375
Efpeglenatide	0.01185	0.031975	0.040825	0.048625	0.0546125	0.0595625	0.066025	0.0684625	0.0714625	0.0717375	0.0760375	0.0926	0.1128	0.115425	0.0602125	0.0177875	42.59083333
Placebo_or_Control	0	0	3.75E-05	3.00E-04	0.0022	0.0088375	0.0290125	0.0749125	0.144	0.2177625	0.2426875	0.1797625	0.081375	0.0177625	0.0013375	1.25E-05	36.70083333
Inject_semaglutide	0.0010375	0.0045125	0.0119	0.020875	0.0318375	0.0445625	0.0581125	0.0724375	0.083175	0.09415	0.1073875	0.1269625	0.150125	0.1299125	0.0529375	0.010075	35.58225
Dulaglutide	0.005025	0.0161625	0.0251375	0.0339	0.039925	0.046375	0.049875	0.0560625	0.0579	0.062375	0.071125	0.0924375	0.124	0.181475	0.1085125	0.0297125	34.5595
Tirzepatide	0.0006625	0.0019875	0.0038	0.0051125	0.0074125	0.0095625	0.01155	0.0138125	0.01585	0.0193375	0.0255625	0.03925	0.0649	0.1284875	0.3865	0.2662125	12.60058333
Oral_semaglutide	0.000525	0.0015	0.00215	0.0029625	0.0035875	0.004325	0.0055375	0.006075	0.0075375	0.008825	0.012	0.0184875	0.0321	0.062525	0.21105	0.6208125	6.303916667

Table S7A: inconsistency within the network meta-analysis of primary outcome: incidence of hearing loss

	Comparison	No.Studies	NMA	Direct	Indirect	Difference	Diff_95CI_lower	Diff_95CI_upper	*p* value
1	Albiglutide:Bexagliflozin	0	-2.702488189	NA	-2.702488189	NA	NA	NA	NA
2	Albiglutide:Canagliflozin	0	-1.026627714	NA	-1.026627714	NA	NA	NA	NA
3	Albiglutide:Dapagliflozin	0	-1.829689142	NA	-1.829689142	NA	NA	NA	NA
4	Albiglutide:Dulaglutide	0	-0.965893446	NA	-0.965893446	NA	NA	NA	NA
5	Albiglutide:Efpeglenatide	0	-2.524550194	NA	-2.524550194	NA	NA	NA	NA
6	Albiglutide:Empagliflozin	0	-1.144270447	NA	-1.144270447	NA	NA	NA	NA
7	Albiglutide:Ertugliflozin	0	-2.021519646	NA	-2.021519646	NA	NA	NA	NA
8	Albiglutide:Exenatide	0	-2.01969392	NA	-2.01969392	NA	NA	NA	NA
9	Albiglutide:Inject_semaglutide	0	-1.727878885	NA	-1.727878885	NA	NA	NA	NA
10	Albiglutide:Liraglutide	0	-2.998080272	NA	-2.998080272	NA	NA	NA	NA
11	Albiglutide:Lixisenatide	0	-3.809160922	NA	-3.809160922	NA	NA	NA	NA
12	Albiglutide:Oral_semaglutide	0	-2.125408265	NA	-2.125408265	NA	NA	NA	NA
13	Albiglutide:Placebo_or_Control	1	-1.610286179	-1.610286179	NA	NA	NA	NA	NA
14	Albiglutide:Sotagliflozin	0	0.187479314	NA	0.187479314	NA	NA	NA	NA
15	Albiglutide:Tirzepatide	0	-0.720693839	NA	-0.720693839	NA	NA	NA	NA
16	Bexagliflozin:Canagliflozin	0	1.675860475	NA	1.675860475	NA	NA	NA	NA
17	Bexagliflozin:Dapagliflozin	0	0.872799047	NA	0.872799047	NA	NA	NA	NA
18	Bexagliflozin:Dulaglutide	0	1.736594743	NA	1.736594743	NA	NA	NA	NA
19	Bexagliflozin:Efpeglenatide	0	0.177937995	NA	0.177937995	NA	NA	NA	NA

20	Bexagliflozin:Empagliflozin	0	1.558217742	NA	1.558217742	NA	NA	NA	NA
21	Bexagliflozin:Ertugliflozin	0	0.680968543	NA	0.680968543	NA	NA	NA	NA
22	Bexagliflozin:Exenatide	0	0.682794269	NA	0.682794269	NA	NA	NA	NA
23	Bexagliflozin:Inject_semaglutide	0	0.974609304	NA	0.974609304	NA	NA	NA	NA
24	Bexagliflozin:Liraglutide	0	-0.295592083	NA	-0.295592083	NA	NA	NA	NA
25	Bexagliflozin:Lixisenatide	0	-1.106672732	NA	-1.106672732	NA	NA	NA	NA
26	Bexagliflozin:Oral_semaglutide	0	0.577079925	NA	0.577079925	NA	NA	NA	NA
27	Bexagliflozin:Placebo_or_Control	1	1.09220201	1.09220201	NA	NA	NA	NA	NA
28	Bexagliflozin:Sotagliflozin	0	2.889967504	NA	2.889967504	NA	NA	NA	NA
29	Bexagliflozin:Tirzepatide	0	1.98179435	NA	1.98179435	NA	NA	NA	NA
30	Canagliflozin:Dapagliflozin	0	-0.803061429	NA	-0.803061429	NA	NA	NA	NA
31	Canagliflozin:Dulaglutide	0	0.060734268	NA	0.060734268	NA	NA	NA	NA
32	Canagliflozin:Efpeglenatide	0	-1.49792248	NA	-1.49792248	NA	NA	NA	NA
33	Canagliflozin:Empagliflozin	0	-0.117642734	NA	-0.117642734	NA	NA	NA	NA
34	Canagliflozin:Ertugliflozin	0	-0.994891932	NA	-0.994891932	NA	NA	NA	NA
35	Canagliflozin:Exenatide	0	-0.993066206	NA	-0.993066206	NA	NA	NA	NA
36	Canagliflozin:Inject_semaglutide	0	-0.701251171	NA	-0.701251171	NA	NA	NA	NA
37	Canagliflozin:Liraglutide	0	-1.971452559	NA	-1.971452559	NA	NA	NA	NA
38	Canagliflozin:Lixisenatide	0	-2.782533208	NA	-2.782533208	NA	NA	NA	NA
39	Canagliflozin:Oral_semaglutide	0	-1.098780551	NA	-1.098780551	NA	NA	NA	NA
40	Canagliflozin:Placebo_or_Control	3	-0.583658465	-0.583658465	NA	NA	NA	NA	NA
41	Canagliflozin:Sotagliflozin	0	1.214107028	NA	1.214107028	NA	NA	NA	NA

42	Canagliflozin:Tirzepatide	0	0.305933875	NA	0.305933875	NA	NA	NA	NA
43	Dapagliflozin:Dulaglutide	0	0.863795696	NA	0.863795696	NA	NA	NA	NA
44	Dapagliflozin:Efpeglenatide	0	-0.694861052	NA	-0.694861052	NA	NA	NA	NA
45	Dapagliflozin:Empagliflozin	0	0.685418695	NA	0.685418695	NA	NA	NA	NA
46	Dapagliflozin:Ertugliflozin	0	-0.191830504	NA	-0.191830504	NA	NA	NA	NA
47	Dapagliflozin:Exenatide	0	-0.190004778	NA	-0.190004778	NA	NA	NA	NA
48	Dapagliflozin:Inject_semaglutide	0	0.101810257	NA	0.101810257	NA	NA	NA	NA
49	Dapagliflozin:Liraglutide	0	-1.16839113	NA	-1.16839113	NA	NA	NA	NA
50	Dapagliflozin:Lixisenatide	0	-1.979471779	NA	-1.979471779	NA	NA	NA	NA
51	Dapagliflozin:Oral_semaglutide	0	-0.295719122	NA	-0.295719122	NA	NA	NA	NA
52	Dapagliflozin:Placebo_or_Control	2	0.219402963	0.219402963	NA	NA	NA	NA	NA
53	Dapagliflozin:Sotagliflozin	0	2.017168457	NA	2.017168457	NA	NA	NA	NA
54	Dapagliflozin:Tirzepatide	0	1.108995303	NA	1.108995303	NA	NA	NA	NA
55	Dulaglutide:Efpeglenatide	0	-1.558656748	NA	-1.558656748	NA	NA	NA	NA
56	Dulaglutide:Empagliflozin	0	-0.178377001	NA	-0.178377001	NA	NA	NA	NA
57	Dulaglutide:Ertugliflozin	0	-1.0556262	NA	-1.0556262	NA	NA	NA	NA
58	Dulaglutide:Exenatide	0	-1.053800474	NA	-1.053800474	NA	NA	NA	NA
59	Dulaglutide:Inject_semaglutide	0	-0.761985439	NA	-0.761985439	NA	NA	NA	NA
60	Dulaglutide:Liraglutide	0	-2.032186826	NA	-2.032186826	NA	NA	NA	NA
61	Dulaglutide:Lixisenatide	0	-2.843267475	NA	-2.843267475	NA	NA	NA	NA
62	Dulaglutide:Oral_semaglutide	0	-1.159514819	NA	-1.159514819	NA	NA	NA	NA
63	Dulaglutide:Placebo_or_Control	2	-0.644392733	-0.644392733	NA	NA	NA	NA	NA

64	Dulaglutide:Sotagliflozin	0	1.153372761	NA	1.153372761	NA	NA	NA	NA
65	Dulaglutide:Tirzepatide	0	0.245199607	NA	0.245199607	NA	NA	NA	NA
66	Efpeglenatide:Empagliflozin	0	1.380279747	NA	1.380279747	NA	NA	NA	NA
67	Efpeglenatide:Ertugliflozin	0	0.503030548	NA	0.503030548	NA	NA	NA	NA
68	Efpeglenatide:Exenatide	0	0.504856274	NA	0.504856274	NA	NA	NA	NA
69	Efpeglenatide:Inject_semaglutide	0	0.796671309	NA	0.796671309	NA	NA	NA	NA
70	Efpeglenatide:Liraglutide	0	-0.473530078	NA	-0.473530078	NA	NA	NA	NA
71	Efpeglenatide:Lixisenatide	0	-1.284610727	NA	-1.284610727	NA	NA	NA	NA
72	Efpeglenatide:Oral_semaglutide	0	0.39914193	NA	0.39914193	NA	NA	NA	NA
73	Efpeglenatide:Placebo_or_Control	1	0.914264015	0.914264015	NA	NA	NA	NA	NA
74	Efpeglenatide:Sotagliflozin	0	2.712029509	NA	2.712029509	NA	NA	NA	NA
75	Efpeglenatide:Tirzepatide	0	1.803856355	NA	1.803856355	NA	NA	NA	NA
76	Empagliflozin:Ertugliflozin	0	-0.877249199	NA	-0.877249199	NA	NA	NA	NA
77	Empagliflozin:Exenatide	0	-0.875423473	NA	-0.875423473	NA	NA	NA	NA
78	Empagliflozin:Inject_semaglutide	0	-0.583608438	NA	-0.583608438	NA	NA	NA	NA
79	Empagliflozin:Liraglutide	0	-1.853809825	NA	-1.853809825	NA	NA	NA	NA
80	Empagliflozin:Lixisenatide	0	-2.664890474	NA	-2.664890474	NA	NA	NA	NA
81	Empagliflozin:Oral_semaglutide	0	-0.981137817	NA	-0.981137817	NA	NA	NA	NA
82	Empagliflozin:Placebo_or_Control	6	-0.466015732	-0.466015732	NA	NA	NA	NA	NA
83	Empagliflozin:Sotagliflozin	0	1.331749762	NA	1.331749762	NA	NA	NA	NA
84	Empagliflozin:Tirzepatide	0	0.423576608	NA	0.423576608	NA	NA	NA	NA
85	Ertugliflozin:Exenatide	0	0.001825726	NA	0.001825726	NA	NA	NA	NA

86	Ertugliflozin:Inject_semaglutide	0	0.293640761	NA	0.293640761	NA	NA	NA	NA
87	Ertugliflozin:Liraglutide	0	-0.976560627	NA	-0.976560627	NA	NA	NA	NA
88	Ertugliflozin:Lixisenatide	0	-1.787641276	NA	-1.787641276	NA	NA	NA	NA
89	Ertugliflozin:Oral_semaglutide	0	-0.103888619	NA	-0.103888619	NA	NA	NA	NA
90	Ertugliflozin:Placebo_or_Control	2	0.411233467	0.411233467	NA	NA	NA	NA	NA
91	Ertugliflozin:Sotagliflozin	0	2.20899896	NA	2.20899896	NA	NA	NA	NA
92	Ertugliflozin:Tirzepatide	0	1.300825807	NA	1.300825807	NA	NA	NA	NA
93	Exenatide:Inject_semaglutide	0	0.291815035	NA	0.291815035	NA	NA	NA	NA
94	Exenatide:Liraglutide	0	-0.978386352	NA	-0.978386352	NA	NA	NA	NA
95	Exenatide:Lixisenatide	0	-1.789467002	NA	-1.789467002	NA	NA	NA	NA
96	Exenatide:Oral_semaglutide	0	-0.105714345	NA	-0.105714345	NA	NA	NA	NA
97	Exenatide:Placebo_or_Control	1	0.409407741	0.409407741	NA	NA	NA	NA	NA
98	Exenatide:Sotagliflozin	0	2.207173234	NA	2.207173234	NA	NA	NA	NA
99	Exenatide:Tirzepatide	0	1.299000081	NA	1.299000081	NA	NA	NA	NA
100	Inject_semaglutide:Liraglutide	0	-1.270201388	NA	-1.270201388	NA	NA	NA	NA
101	Inject_semaglutide:Lixisenatide	0	-2.081282037	NA	-2.081282037	NA	NA	NA	NA
102	Inject_semaglutide:Oral_semaglutide	0	-0.39752938	NA	-0.39752938	NA	NA	NA	NA
103	Inject_semaglutide:Placebo_or_Control	3	0.117592706	0.117592706	NA	NA	NA	NA	NA
104	Inject_semaglutide:Sotagliflozin	0	1.915358199	NA	1.915358199	NA	NA	NA	NA
105	Inject_semaglutide:Tirzepatide	0	1.007185046	NA	1.007185046	NA	NA	NA	NA
106	Liraglutide:Lixisenatide	0	-0.811080649	NA	-0.811080649	NA	NA	NA	NA
107	Liraglutide:Oral_semaglutide	0	0.872672008	NA	0.872672008	NA	NA	NA	NA

108	Liraglutide:Placebo_or_Control	1	1.387794094	1.387794094	NA	NA	NA	NA	NA
109	Liraglutide:Sotagliflozin	0	3.185559587	NA	3.185559587	NA	NA	NA	NA
110	Liraglutide:Tirzepatide	0	2.277386433	NA	2.277386433	NA	NA	NA	NA
111	Lixisenatide:Oral_semaglutide	0	1.683752657	NA	1.683752657	NA	NA	NA	NA
112	Lixisenatide:Placebo_or_Control	1	2.198874743	2.198874743	NA	NA	NA	NA	NA
113	Lixisenatide:Sotagliflozin	0	3.996640236	NA	3.996640236	NA	NA	NA	NA
114	Lixisenatide:Tirzepatide	0	3.088467083	NA	3.088467083	NA	NA	NA	NA
115	Oral_semaglutide:Placebo_or_Control	1	0.515122086	0.515122086	NA	NA	NA	NA	NA
116	Oral_semaglutide:Sotagliflozin	0	2.312887579	NA	2.312887579	NA	NA	NA	NA
117	Oral_semaglutide:Tirzepatide	0	1.404714426	NA	1.404714426	NA	NA	NA	NA
118	Sotagliflozin:Placebo_or_Control	1	-1.797765493	-1.797765493	NA	NA	NA	NA	NA
119	Tirzepatide:Placebo_or_Control	2	-0.88959234	-0.88959234	NA	NA	NA	NA	NA
120	Sotagliflozin:Tirzepatide	0	-0.908173153	NA	-0.908173153	NA	NA	NA	NA

Table S7B: inconsistency within the network meta-analysis of primary outcome: incidence of hearing loss in subgroup of dosage

	Comparison	No.Studies	NMA	Direct	Indirect	Difference	Diff_95CI_lower	Diff_95CI_upper	*p* value
1	Albiglutide:Bexagliflozin	0	-2.702488189	NA	-2.702488189	NA	NA	NA	NA
2	Albiglutide:Canagliflozin_high_dosage	0	-1.028101095	NA	-1.028101095	NA	NA	NA	NA
3	Albiglutide:Canagliflozin_low_dosage	0	-1.02515165	NA	-1.02515165	NA	NA	NA	NA
4	Albiglutide:Dapagliflozin	0	-1.829689142	NA	-1.829689142	NA	NA	NA	NA
5	Albiglutide:Dulaglutide_high_dosage	0	-1.10938717	NA	-1.10938717	NA	NA	NA	NA
6	Albiglutide:Dulaglutide_low_dosage	0	-0.010774881	NA	-0.010774881	NA	NA	NA	NA
7	Albiglutide:Efpeglenatide_high_dosage	0	-3.218986628	NA	-3.218986628	NA	NA	NA	NA
8	Albiglutide:Efpeglenatide_low_dosage	0	-1.60660429	NA	-1.60660429	NA	NA	NA	NA
9	Albiglutide:Empagliflozin_high_dosage	0	-1.064754584	NA	-1.064754584	NA	NA	NA	NA
10	Albiglutide:Empagliflozin_low_dosage	0	-1.153206779	NA	-1.153206779	NA	NA	NA	NA
11	Albiglutide:Ertugliflozin_high_dosage	0	-2.149792434	NA	-2.149792434	NA	NA	NA	NA
12	Albiglutide:Ertugliflozin_low_dosage	0	-2.452023508	NA	-2.452023508	NA	NA	NA	NA
13	Albiglutide:Exenatide	0	-2.01969392	NA	-2.01969392	NA	NA	NA	NA
14	Albiglutide:Inject_semaglutide_high_dosage	0	-2.015637644	NA	-2.015637644	NA	NA	NA	NA
15	Albiglutide:Inject_semaglutide_low_dosage	0	-1.068285836	NA	-1.068285836	NA	NA	NA	NA
16	Albiglutide:Inject_semaglutide_medium_dosage	0	-2.025899952	NA	-2.025899952	NA	NA	NA	NA
17	Albiglutide:Liraglutide	0	-2.998080272	NA	-2.998080272	NA	NA	NA	NA

18	Albiglutide:Lixisenatide	0	-3.809160922	NA	-3.809160922	NA	NA	NA	NA
19	Albiglutide:Oral_semaglutide	0	-2.125408265	NA	-2.125408265	NA	NA	NA	NA
20	Albiglutide:Placebo_or_Control	1	-1.610286179	-1.610286179	NA	NA	NA	NA	NA
21	Albiglutide:Sotagliflozin	0	0.187479314	NA	0.187479314	NA	NA	NA	NA
22	Albiglutide:Tirzepatide_high_dosage	0	-1.311296761	NA	-1.311296761	NA	NA	NA	NA
23	Albiglutide:Tirzepatide_low_dosage	0	-0.11150438	NA	-0.11150438	NA	NA	NA	NA
24	Albiglutide:Tirzepatide_medium_dosage	0	-1.097231029	NA	-1.097231029	NA	NA	NA	NA
25	Bexagliflozin:Canagliflozin_high_dosage	0	1.674387094	NA	1.674387094	NA	NA	NA	NA
26	Bexagliflozin:Canagliflozin_low_dosage	0	1.677336539	NA	1.677336539	NA	NA	NA	NA
27	Bexagliflozin:Dapagliflozin	0	0.872799047	NA	0.872799047	NA	NA	NA	NA
28	Bexagliflozin:Dulaglutide_high_dosage	0	1.593101019	NA	1.593101019	NA	NA	NA	NA
29	Bexagliflozin:Dulaglutide_low_dosage	0	2.691713308	NA	2.691713308	NA	NA	NA	NA
30	Bexagliflozin:Efpeglenatide_high_dosage	0	-0.516498439	NA	-0.516498439	NA	NA	NA	NA
31	Bexagliflozin:Efpeglenatide_low_dosage	0	1.0958839	NA	1.0958839	NA	NA	NA	NA
32	Bexagliflozin:Empagliflozin_high_dosage	0	1.637733605	NA	1.637733605	NA	NA	NA	NA
33	Bexagliflozin:Empagliflozin_low_dosage	0	1.54928141	NA	1.54928141	NA	NA	NA	NA
34	Bexagliflozin:Ertugliflozin_high_dosage	0	0.552695755	NA	0.552695755	NA	NA	NA	NA
35	Bexagliflozin:Ertugliflozin_low_dosage	0	0.250464681	NA	0.250464681	NA	NA	NA	NA
36	Bexagliflozin:Exenatide	0	0.682794269	NA	0.682794269	NA	NA	NA	NA
37	Bexagliflozin:Inject_semaglutide_high_dosage	0	0.686850545	NA	0.686850545	NA	NA	NA	NA
38	Bexagliflozin:Inject_semaglutide_low_dosage	0	1.634202353	NA	1.634202353	NA	NA	NA	NA
39	Bexagliflozin:Inject_semaglutide_medium_dosage	0	0.676588237	NA	0.676588237	NA	NA	NA	NA

40	Bexagliflozin:Liraglutide	0	-0.295592083	NA	-0.295592083	NA	NA	NA	NA
41	Bexagliflozin:Lixisenatide	0	-1.106672732	NA	-1.106672732	NA	NA	NA	NA
42	Bexagliflozin:Oral_semaglutide	0	0.577079925	NA	0.577079925	NA	NA	NA	NA
43	Bexagliflozin:Placebo_or_Control	1	1.09220201	1.09220201	NA	NA	NA	NA	NA
44	Bexagliflozin:Sotagliflozin	0	2.889967504	NA	2.889967504	NA	NA	NA	NA
45	Bexagliflozin:Tirzepatide_high_dosage	0	1.391191428	NA	1.391191428	NA	NA	NA	NA
46	Bexagliflozin:Tirzepatide_low_dosage	0	2.590983809	NA	2.590983809	NA	NA	NA	NA
47	Bexagliflozin:Tirzepatide_medium_dosage	0	1.60525716	NA	1.60525716	NA	NA	NA	NA
48	Canagliflozin_high_dosage:Canagliflozin_low_dosage	1	0.002949446	0.002773927	0.003418369	-0.000644443	-5.313695398	5.312406512	0.999810317
49	Canagliflozin_high_dosage:Dapagliflozin	0	-0.801588047	NA	-0.801588047	NA	NA	NA	NA
50	Canagliflozin_high_dosage:Dulaglutide_high_dosage	0	-0.081286074	NA	-0.081286074	NA	NA	NA	NA
51	Canagliflozin_high_dosage:Dulaglutide_low_dosage	0	1.017326214	NA	1.017326214	NA	NA	NA	NA
52	Canagliflozin_high_dosage:Efpeglenatide_high_dosage	0	-2.190885533	NA	-2.190885533	NA	NA	NA	NA
53	Canagliflozin_high_dosage:Efpeglenatide_low_dosage	0	-0.578503194	NA	-0.578503194	NA	NA	NA	NA
54	Canagliflozin_high_dosage:Empagliflozin_high_dosage	0	-0.036653489	NA	-0.036653489	NA	NA	NA	NA
55	Canagliflozin_high_dosage:Empagliflozin_low_dosage	0	-0.125105684	NA	-0.125105684	NA	NA	NA	NA
56	Canagliflozin_high_dosage:Ertugliflozin_high_dosage	0	-1.121691339	NA	-1.121691339	NA	NA	NA	NA
57	Canagliflozin_high_dosage:Ertugliflozin_low_dosage	0	-1.423922412	NA	-1.423922412	NA	NA	NA	NA
58	Canagliflozin_high_dosage:Exenatide	0	-0.991592825	NA	-0.991592825	NA	NA	NA	NA
59	Canagliflozin_high_dosage:Inject_semaglutide_high_dosage	0	-0.987536549	NA	-0.987536549	NA	NA	NA	NA
60	Canagliflozin_high_dosage:Inject_semaglutide_low_dosage	0	-0.040184741	NA	-0.040184741	NA	NA	NA	NA
61	Canagliflozin_high_dosage:Inject_semaglutide_medium_dosage	0	-0.997798857	NA	-0.997798857	NA	NA	NA	NA

62	Canagliflozin_high_dosage:Liraglutide	0	-1.969979177	NA	-1.969979177	NA	NA	NA	NA
63	Canagliflozin_high_dosage:Lixisenatide	0	-2.781059826	NA	-2.781059826	NA	NA	NA	NA
64	Canagliflozin_high_dosage:Oral_semaglutide	0	-1.097307169	NA	-1.097307169	NA	NA	NA	NA
65	Canagliflozin_high_dosage:Placebo_or_Control	2	-0.582185083	-0.471246411	-2.204663782	1.733417371	-6.551044906	10.01787965	0.681734573
66	Canagliflozin_high_dosage:Sotagliflozin	0	1.21558041	NA	1.21558041	NA	NA	NA	NA
67	Canagliflozin_high_dosage:Tirzepatide_high_dosage	0	-0.283195666	NA	-0.283195666	NA	NA	NA	NA
68	Canagliflozin_high_dosage:Tirzepatide_low_dosage	0	0.916596716	NA	0.916596716	NA	NA	NA	NA
69	Canagliflozin_high_dosage:Tirzepatide_medium_dosage	0	-0.069129934	NA	-0.069129934	NA	NA	NA	NA
70	Canagliflozin_low_dosage:Dapagliflozin	0	-0.804537493	NA	-0.804537493	NA	NA	NA	NA
71	Canagliflozin_low_dosage:Dulaglutide_high_dosage	0	-0.08423552	NA	-0.08423552	NA	NA	NA	NA
72	Canagliflozin_low_dosage:Dulaglutide_low_dosage	0	1.014376769	NA	1.014376769	NA	NA	NA	NA
73	Canagliflozin_low_dosage:Efpeglenatide_high_dosage	0	-2.193834978	NA	-2.193834978	NA	NA	NA	NA
74	Canagliflozin_low_dosage:Efpeglenatide_low_dosage	0	-0.58145264	NA	-0.58145264	NA	NA	NA	NA
75	Canagliflozin_low_dosage:Empagliflozin_high_dosage	0	-0.039602934	NA	-0.039602934	NA	NA	NA	NA
76	Canagliflozin_low_dosage:Empagliflozin_low_dosage	0	-0.12805513	NA	-0.12805513	NA	NA	NA	NA
77	Canagliflozin_low_dosage:Ertugliflozin_high_dosage	0	-1.124640784	NA	-1.124640784	NA	NA	NA	NA
78	Canagliflozin_low_dosage:Ertugliflozin_low_dosage	0	-1.426871858	NA	-1.426871858	NA	NA	NA	NA
79	Canagliflozin_low_dosage:Exenatide	0	-0.99454227	NA	-0.99454227	NA	NA	NA	NA
80	Canagliflozin_low_dosage:Inject_semaglutide_high_dosage	0	-0.990485994	NA	-0.990485994	NA	NA	NA	NA
81	Canagliflozin_low_dosage:Inject_semaglutide_low_dosage	0	-0.043134186	NA	-0.043134186	NA	NA	NA	NA
82	Canagliflozin_low_dosage:Inject_semaglutide_medium_dosage	0	-1.000748302	NA	-1.000748302	NA	NA	NA	NA
83	Canagliflozin_low_dosage:Liraglutide	0	-1.972928623	NA	-1.972928623	NA	NA	NA	NA

84	Canagliflozin_low_dosage:Lixisenatide	0	-2.784009272	NA	-2.784009272	NA	NA	NA	NA
85	Canagliflozin_low_dosage:Oral_semaglutide	0	-1.100256615	NA	-1.100256615	NA	NA	NA	NA
86	Canagliflozin_low_dosage:Placebo_or_Control	2	-0.585134529	-0.474077806	-2.201374039	1.727296233	-6.546269908	10.00086237	0.682402195
87	Canagliflozin_low_dosage:Sotagliflozin	0	1.212630964	NA	1.212630964	NA	NA	NA	NA
88	Canagliflozin_low_dosage:Tirzepatide_high_dosage	0	-0.286145111	NA	-0.286145111	NA	NA	NA	NA
89	Canagliflozin_low_dosage:Tirzepatide_low_dosage	0	0.91364727	NA	0.91364727	NA	NA	NA	NA
90	Canagliflozin_low_dosage:Tirzepatide_medium_dosage	0	-0.07207938	NA	-0.07207938	NA	NA	NA	NA
91	Dapagliflozin:Dulaglutide_high_dosage	0	0.720301972	NA	0.720301972	NA	NA	NA	NA
92	Dapagliflozin:Dulaglutide_low_dosage	0	1.818914261	NA	1.818914261	NA	NA	NA	NA
93	Dapagliflozin:Efpeglenatide_high_dosage	0	-1.389297486	NA	-1.389297486	NA	NA	NA	NA
94	Dapagliflozin:Efpeglenatide_low_dosage	0	0.223084853	NA	0.223084853	NA	NA	NA	NA
95	Dapagliflozin:Empagliflozin_high_dosage	0	0.764934558	NA	0.764934558	NA	NA	NA	NA
96	Dapagliflozin:Empagliflozin_low_dosage	0	0.676482363	NA	0.676482363	NA	NA	NA	NA
97	Dapagliflozin:Ertugliflozin_high_dosage	0	-0.320103292	NA	-0.320103292	NA	NA	NA	NA
98	Dapagliflozin:Ertugliflozin_low_dosage	0	-0.622334365	NA	-0.622334365	NA	NA	NA	NA
99	Dapagliflozin:Exenatide	0	-0.190004778	NA	-0.190004778	NA	NA	NA	NA
100	Dapagliflozin:Inject_semaglutide_high_dosage	0	-0.185948502	NA	-0.185948502	NA	NA	NA	NA
101	Dapagliflozin:Inject_semaglutide_low_dosage	0	0.761403306	NA	0.761403306	NA	NA	NA	NA
102	Dapagliflozin:Inject_semaglutide_medium_dosage	0	-0.19621081	NA	-0.19621081	NA	NA	NA	NA
103	Dapagliflozin:Liraglutide	0	-1.16839113	NA	-1.16839113	NA	NA	NA	NA
104	Dapagliflozin:Lixisenatide	0	-1.979471779	NA	-1.979471779	NA	NA	NA	NA
105	Dapagliflozin:Oral_semaglutide	0	-0.295719122	NA	-0.295719122	NA	NA	NA	NA

106	Dapagliflozin:Placebo_or_Control	2	0.219402963	0.219402963	NA	NA	NA	NA	NA
107	Dapagliflozin:Sotagliflozin	0	2.017168457	NA	2.017168457	NA	NA	NA	NA
108	Dapagliflozin:Tirzepatide_high_dosage	0	0.518392381	NA	0.518392381	NA	NA	NA	NA
109	Dapagliflozin:Tirzepatide_low_dosage	0	1.718184763	NA	1.718184763	NA	NA	NA	NA
110	Dapagliflozin:Tirzepatide_medium_dosage	0	0.732458113	NA	0.732458113	NA	NA	NA	NA
111	Dulaglutide_high_dosage:Dulaglutide_low_dosage	1	1.098612289	1.098612289	NA	NA	NA	NA	NA
112	Dulaglutide_high_dosage:Efpeglenatide_high_dosage	0	-2.109599458	NA	-2.109599458	NA	NA	NA	NA
113	Dulaglutide_high_dosage:Efpeglenatide_low_dosage	0	-0.49721712	NA	-0.49721712	NA	NA	NA	NA
114	Dulaglutide_high_dosage:Empagliflozin_high_dosage	0	0.044632586	NA	0.044632586	NA	NA	NA	NA
115	Dulaglutide_high_dosage:Empagliflozin_low_dosage	0	-0.043819609	NA	-0.043819609	NA	NA	NA	NA
116	Dulaglutide_high_dosage:Ertugliflozin_high_dosage	0	-1.040405264	NA	-1.040405264	NA	NA	NA	NA
117	Dulaglutide_high_dosage:Ertugliflozin_low_dosage	0	-1.342636338	NA	-1.342636338	NA	NA	NA	NA
118	Dulaglutide_high_dosage:Exenatide	0	-0.91030675	NA	-0.91030675	NA	NA	NA	NA
119	Dulaglutide_high_dosage:Inject_semaglutide_high_dosage	0	-0.906250474	NA	-0.906250474	NA	NA	NA	NA
120	Dulaglutide_high_dosage:Inject_semaglutide_low_dosage	0	0.041101334	NA	0.041101334	NA	NA	NA	NA
121	Dulaglutide_high_dosage:Inject_semaglutide_medium_dosage	0	-0.916512782	NA	-0.916512782	NA	NA	NA	NA
122	Dulaglutide_high_dosage:Liraglutide	0	-1.888693103	NA	-1.888693103	NA	NA	NA	NA
123	Dulaglutide_high_dosage:Lixisenatide	0	-2.699773752	NA	-2.699773752	NA	NA	NA	NA
124	Dulaglutide_high_dosage:Oral_semaglutide	0	-1.016021095	NA	-1.016021095	NA	NA	NA	NA
125	Dulaglutide_high_dosage:Placebo_or_Control	2	-0.500899009	-0.500899009	NA	NA	NA	NA	NA
126	Dulaglutide_high_dosage:Sotagliflozin	0	1.296866484	NA	1.296866484	NA	NA	NA	NA
127	Dulaglutide_high_dosage:Tirzepatide_high_dosage	0	-0.201909591	NA	-0.201909591	NA	NA	NA	NA

128	Dulaglutide_high_dosage:Tirzepatide_low_dosage	0	0.99788279	NA	0.99788279	NA	NA	NA	NA
129	Dulaglutide_high_dosage:Tirzepatide_medium_dosage	0	0.012156141	NA	0.012156141	NA	NA	NA	NA
130	Dulaglutide_low_dosage:Efpeglenatide_high_dosage	0	-3.208211747	NA	-3.208211747	NA	NA	NA	NA
131	Dulaglutide_low_dosage:Efpeglenatide_low_dosage	0	-1.595829408	NA	-1.595829408	NA	NA	NA	NA
132	Dulaglutide_low_dosage:Empagliflozin_high_dosage	0	-1.053979703	NA	-1.053979703	NA	NA	NA	NA
133	Dulaglutide_low_dosage:Empagliflozin_low_dosage	0	-1.142431898	NA	-1.142431898	NA	NA	NA	NA
134	Dulaglutide_low_dosage:Ertugliflozin_high_dosage	0	-2.139017553	NA	-2.139017553	NA	NA	NA	NA
135	Dulaglutide_low_dosage:Ertugliflozin_low_dosage	0	-2.441248627	NA	-2.441248627	NA	NA	NA	NA
136	Dulaglutide_low_dosage:Exenatide	0	-2.008919039	NA	-2.008919039	NA	NA	NA	NA
137	Dulaglutide_low_dosage:Inject_semaglutide_high_dosage	0	-2.004862763	NA	-2.004862763	NA	NA	NA	NA
138	Dulaglutide_low_dosage:Inject_semaglutide_low_dosage	0	-1.057510955	NA	-1.057510955	NA	NA	NA	NA
139	Dulaglutide_low_dosage:Inject_semaglutide_medium_dosage	0	-2.015125071	NA	-2.015125071	NA	NA	NA	NA
140	Dulaglutide_low_dosage:Liraglutide	0	-2.987305391	NA	-2.987305391	NA	NA	NA	NA
141	Dulaglutide_low_dosage:Lixisenatide	0	-3.79838604	NA	-3.79838604	NA	NA	NA	NA
142	Dulaglutide_low_dosage:Oral_semaglutide	0	-2.114633383	NA	-2.114633383	NA	NA	NA	NA
143	Dulaglutide_low_dosage:Placebo_or_Control	0	-1.599511298	NA	-1.599511298	NA	NA	NA	NA
144	Dulaglutide_low_dosage:Sotagliflozin	0	0.198254196	NA	0.198254196	NA	NA	NA	NA
145	Dulaglutide_low_dosage:Tirzepatide_high_dosage	0	-1.30052188	NA	-1.30052188	NA	NA	NA	NA
146	Dulaglutide_low_dosage:Tirzepatide_low_dosage	0	-0.100729499	NA	-0.100729499	NA	NA	NA	NA
147	Dulaglutide_low_dosage:Tirzepatide_medium_dosage	0	-1.086456148	NA	-1.086456148	NA	NA	NA	NA
148	Efpeglenatide_high_dosage:Efpeglenatide_low_dosage	1	1.612382339	1.612382339	NA	NA	NA	NA	NA
149	Efpeglenatide_high_dosage:Empagliflozin_high_dosage	0	2.154232044	NA	2.154232044	NA	NA	NA	NA

150	Efpeglenatide_high_dosage:Empagliflozin_low_dosage	0	2.065779849	NA	2.065779849	NA	NA	NA	NA
151	Efpeglenatide_high_dosage:Ertugliflozin_high_dosage	0	1.069194194	NA	1.069194194	NA	NA	NA	NA
152	Efpeglenatide_high_dosage:Ertugliflozin_low_dosage	0	0.76696312	NA	0.76696312	NA	NA	NA	NA
153	Efpeglenatide_high_dosage:Exenatide	0	1.199292708	NA	1.199292708	NA	NA	NA	NA
154	Efpeglenatide_high_dosage:Inject_semaglutide_high_dosage	0	1.203348984	NA	1.203348984	NA	NA	NA	NA
155	Efpeglenatide_high_dosage:Inject_semaglutide_low_dosage	0	2.150700792	NA	2.150700792	NA	NA	NA	NA
156	Efpeglenatide_high_dosage:Inject_semaglutide_medium_dosage	0	1.193086676	NA	1.193086676	NA	NA	NA	NA
157	Efpeglenatide_high_dosage:Liraglutide	0	0.220906356	NA	0.220906356	NA	NA	NA	NA
158	Efpeglenatide_high_dosage:Lixisenatide	0	-0.590174293	NA	-0.590174293	NA	NA	NA	NA
159	Efpeglenatide_high_dosage:Oral_semaglutide	0	1.093578363	NA	1.093578363	NA	NA	NA	NA
160	Efpeglenatide_high_dosage:Placebo_or_Control	1	1.608700449	1.608700449	NA	NA	NA	NA	NA
161	Efpeglenatide_high_dosage:Sotagliflozin	0	3.406465943	NA	3.406465943	NA	NA	NA	NA
162	Efpeglenatide_high_dosage:Tirzepatide_high_dosage	0	1.907689867	NA	1.907689867	NA	NA	NA	NA
163	Efpeglenatide_high_dosage:Tirzepatide_low_dosage	0	3.107482248	NA	3.107482248	NA	NA	NA	NA
164	Efpeglenatide_high_dosage:Tirzepatide_medium_dosage	0	2.121755599	NA	2.121755599	NA	NA	NA	NA
165	Efpeglenatide_low_dosage:Empagliflozin_high_dosage	0	0.541849705	NA	0.541849705	NA	NA	NA	NA
166	Efpeglenatide_low_dosage:Empagliflozin_low_dosage	0	0.45339751	NA	0.45339751	NA	NA	NA	NA
167	Efpeglenatide_low_dosage:Ertugliflozin_high_dosage	0	-0.543188145	NA	-0.543188145	NA	NA	NA	NA
168	Efpeglenatide_low_dosage:Ertugliflozin_low_dosage	0	-0.845419218	NA	-0.845419218	NA	NA	NA	NA
169	Efpeglenatide_low_dosage:Exenatide	0	-0.41308963	NA	-0.41308963	NA	NA	NA	NA
170	Efpeglenatide_low_dosage:Inject_semaglutide_high_dosage	0	-0.409033355	NA	-0.409033355	NA	NA	NA	NA
171	Efpeglenatide_low_dosage:Inject_semaglutide_low_dosage	0	0.538318454	NA	0.538318454	NA	NA	NA	NA

172	Efpeglenatide_low_dosage:Inject_semaglutide_medium_dosage	0	-0.419295663	NA	-0.419295663	NA	NA	NA	NA
173	Efpeglenatide_low_dosage:Liraglutide	0	-1.391475983	NA	-1.391475983	NA	NA	NA	NA
174	Efpeglenatide_low_dosage:Lixisenatide	0	-2.202556632	NA	-2.202556632	NA	NA	NA	NA
175	Efpeglenatide_low_dosage:Oral_semaglutide	0	-0.518803975	NA	-0.518803975	NA	NA	NA	NA
176	Efpeglenatide_low_dosage:Placebo_or_Control	0	-0.003681889	NA	-0.003681889	NA	NA	NA	NA
177	Efpeglenatide_low_dosage:Sotagliflozin	0	1.794083604	NA	1.794083604	NA	NA	NA	NA
178	Efpeglenatide_low_dosage:Tirzepatide_high_dosage	0	0.295307529	NA	0.295307529	NA	NA	NA	NA
179	Efpeglenatide_low_dosage:Tirzepatide_low_dosage	0	1.49509991	NA	1.49509991	NA	NA	NA	NA
180	Efpeglenatide_low_dosage:Tirzepatide_medium_dosage	0	0.50937326	NA	0.50937326	NA	NA	NA	NA
181	Empagliflozin_high_dosage:Empagliflozin_low_dosage	1	-0.088452195	0.001280683	-0.181469751	0.182750434	-3.77373141	4.139232278	0.927865258
182	Empagliflozin_high_dosage:Ertugliflozin_high_dosage	0	-1.08503785	NA	-1.08503785	NA	NA	NA	NA
183	Empagliflozin_high_dosage:Ertugliflozin_low_dosage	0	-1.387268924	NA	-1.387268924	NA	NA	NA	NA
184	Empagliflozin_high_dosage:Exenatide	0	-0.954939336	NA	-0.954939336	NA	NA	NA	NA
185	Empagliflozin_high_dosage:Inject_semaglutide_high_dosage	0	-0.95088306	NA	-0.95088306	NA	NA	NA	NA
186	Empagliflozin_high_dosage:Inject_semaglutide_low_dosage	0	-0.003531252	NA	-0.003531252	NA	NA	NA	NA
187	Empagliflozin_high_dosage:Inject_semaglutide_medium_dosage	0	-0.961145368	NA	-0.961145368	NA	NA	NA	NA
188	Empagliflozin_high_dosage:Liraglutide	0	-1.933325688	NA	-1.933325688	NA	NA	NA	NA
189	Empagliflozin_high_dosage:Lixisenatide	0	-2.744406337	NA	-2.744406337	NA	NA	NA	NA
190	Empagliflozin_high_dosage:Oral_semaglutide	0	-1.06065368	NA	-1.06065368	NA	NA	NA	NA
191	Empagliflozin_high_dosage:Placebo_or_Control	2	-0.545531595	-0.47624904	-0.938379859	0.462130819	-4.961828493	5.88609013	0.867376061
192	Empagliflozin_high_dosage:Sotagliflozin	0	1.252233899	NA	1.252233899	NA	NA	NA	NA
193	Empagliflozin_high_dosage:Tirzepatide_high_dosage	0	-0.246542177	NA	-0.246542177	NA	NA	NA	NA

194	Empagliflozin_high_dosage:Tirzepatide_low_dosage	0	0.953250204	NA	0.953250204	NA	NA	NA	NA
195	Empagliflozin_high_dosage:Tirzepatide_medium_dosage	0	-0.032476445	NA	-0.032476445	NA	NA	NA	NA
196	Empagliflozin_low_dosage:Ertugliflozin_high_dosage	0	-0.996585655	NA	-0.996585655	NA	NA	NA	NA
197	Empagliflozin_low_dosage:Ertugliflozin_low_dosage	0	-1.298816728	NA	-1.298816728	NA	NA	NA	NA
198	Empagliflozin_low_dosage:Exenatide	0	-0.866487141	NA	-0.866487141	NA	NA	NA	NA
199	Empagliflozin_low_dosage:Inject_semaglutide_high_dosage	0	-0.862430865	NA	-0.862430865	NA	NA	NA	NA
200	Empagliflozin_low_dosage:Inject_semaglutide_low_dosage	0	0.084920943	NA	0.084920943	NA	NA	NA	NA
201	Empagliflozin_low_dosage:Inject_semaglutide_medium_dosage	0	-0.872693173	NA	-0.872693173	NA	NA	NA	NA
202	Empagliflozin_low_dosage:Liraglutide	0	-1.844873493	NA	-1.844873493	NA	NA	NA	NA
203	Empagliflozin_low_dosage:Lixisenatide	0	-2.655954142	NA	-2.655954142	NA	NA	NA	NA
204	Empagliflozin_low_dosage:Oral_semaglutide	0	-0.972201485	NA	-0.972201485	NA	NA	NA	NA
205	Empagliflozin_low_dosage:Placebo_or_Control	6	-0.4570794	-0.444524941	-2.222048597	1.777523656	-6.279367853	9.834415165	0.665443281
206	Empagliflozin_low_dosage:Sotagliflozin	0	1.340686094	NA	1.340686094	NA	NA	NA	NA
207	Empagliflozin_low_dosage:Tirzepatide_high_dosage	0	-0.158089982	NA	-0.158089982	NA	NA	NA	NA
208	Empagliflozin_low_dosage:Tirzepatide_low_dosage	0	1.0417024	NA	1.0417024	NA	NA	NA	NA
209	Empagliflozin_low_dosage:Tirzepatide_medium_dosage	0	0.05597575	NA	0.05597575	NA	NA	NA	NA
210	Ertugliflozin_high_dosage:Ertugliflozin_low_dosage	2	-0.302231074	-0.537330523	0.592949082	-1.130279605	-5.902327009	3.641767799	0.642486009
211	Ertugliflozin_high_dosage:Exenatide	0	0.130098514	NA	0.130098514	NA	NA	NA	NA
212	Ertugliflozin_high_dosage:Inject_semaglutide_high_dosage	0	0.13415479	NA	0.13415479	NA	NA	NA	NA
213	Ertugliflozin_high_dosage:Inject_semaglutide_low_dosage	0	1.081506598	NA	1.081506598	NA	NA	NA	NA
214	Ertugliflozin_high_dosage:Inject_semaglutide_medium_dosage	0	0.123892482	NA	0.123892482	NA	NA	NA	NA
215	Ertugliflozin_high_dosage:Liraglutide	0	-0.848287838	NA	-0.848287838	NA	NA	NA	NA

216	Ertugliflozin_high_dosage:Lixisenatide	0	-1.659368487	NA	-1.659368487	NA	NA	NA	NA
217	Ertugliflozin_high_dosage:Oral_semaglutide	0	0.02438417	NA	0.02438417	NA	NA	NA	NA
218	Ertugliflozin_high_dosage:Placebo_or_Control	2	0.539506255	0.009673603	2.981390709	-2.971717106	-8.336768754	2.393334542	0.27764293
219	Ertugliflozin_high_dosage:Sotagliflozin	0	2.337271749	NA	2.337271749	NA	NA	NA	NA
220	Ertugliflozin_high_dosage:Tirzepatide_high_dosage	0	0.838495673	NA	0.838495673	NA	NA	NA	NA
221	Ertugliflozin_high_dosage:Tirzepatide_low_dosage	0	2.038288054	NA	2.038288054	NA	NA	NA	NA
222	Ertugliflozin_high_dosage:Tirzepatide_medium_dosage	0	1.052561405	NA	1.052561405	NA	NA	NA	NA
223	Ertugliflozin_low_dosage:Exenatide	0	0.432329588	NA	0.432329588	NA	NA	NA	NA
224	Ertugliflozin_low_dosage:Inject_semaglutide_high_dosage	0	0.436385864	NA	0.436385864	NA	NA	NA	NA
225	Ertugliflozin_low_dosage:Inject_semaglutide_low_dosage	0	1.383737672	NA	1.383737672	NA	NA	NA	NA
226	Ertugliflozin_low_dosage:Inject_semaglutide_medium_dosage	0	0.426123556	NA	0.426123556	NA	NA	NA	NA
227	Ertugliflozin_low_dosage:Liraglutide	0	-0.546056765	NA	-0.546056765	NA	NA	NA	NA
228	Ertugliflozin_low_dosage:Lixisenatide	0	-1.357137414	NA	-1.357137414	NA	NA	NA	NA
229	Ertugliflozin_low_dosage:Oral_semaglutide	0	0.326615243	NA	0.326615243	NA	NA	NA	NA
230	Ertugliflozin_low_dosage:Placebo_or_Control	1	0.841737329	0.847662292	0.810358217	0.037304075	-4.763952096	4.838560246	0.987850098
231	Ertugliflozin_low_dosage:Sotagliflozin	0	2.639502822	NA	2.639502822	NA	NA	NA	NA
232	Ertugliflozin_low_dosage:Tirzepatide_high_dosage	0	1.140726747	NA	1.140726747	NA	NA	NA	NA
233	Ertugliflozin_low_dosage:Tirzepatide_low_dosage	0	2.340519128	NA	2.340519128	NA	NA	NA	NA
234	Ertugliflozin_low_dosage:Tirzepatide_medium_dosage	0	1.354792478	NA	1.354792478	NA	NA	NA	NA
235	Exenatide:Inject_semaglutide_high_dosage	0	0.004056276	NA	0.004056276	NA	NA	NA	NA
236	Exenatide:Inject_semaglutide_low_dosage	0	0.951408084	NA	0.951408084	NA	NA	NA	NA
237	Exenatide:Inject_semaglutide_medium_dosage	0	-0.006206032	NA	-0.006206032	NA	NA	NA	NA

238	Exenatide:Liraglutide	0	-0.978386352	NA	-0.978386352	NA	NA	NA	NA
239	Exenatide:Lixisenatide	0	-1.789467002	NA	-1.789467002	NA	NA	NA	NA
240	Exenatide:Oral_semaglutide	0	-0.105714345	NA	-0.105714345	NA	NA	NA	NA
241	Exenatide:Placebo_or_Control	1	0.409407741	0.409407741	NA	NA	NA	NA	NA
242	Exenatide:Sotagliflozin	0	2.207173234	NA	2.207173234	NA	NA	NA	NA
243	Exenatide:Tirzepatide_high_dosage	0	0.708397159	NA	0.708397159	NA	NA	NA	NA
244	Exenatide:Tirzepatide_low_dosage	0	1.90818954	NA	1.90818954	NA	NA	NA	NA
245	Exenatide:Tirzepatide_medium_dosage	0	0.922462891	NA	0.922462891	NA	NA	NA	NA
246	Inject_semaglutide_high_dosage:Inject_semaglutide_low_dosage	0	0.947351808	NA	0.947351808	NA	NA	NA	NA
247	Inject_semaglutide_high_dosage:Inject_semaglutide_medium_dosage	0	-0.010262308	NA	-0.010262308	NA	NA	NA	NA
248	Inject_semaglutide_high_dosage:Liraglutide	0	-0.982442628	NA	-0.982442628	NA	NA	NA	NA
249	Inject_semaglutide_high_dosage:Lixisenatide	0	-1.793523277	NA	-1.793523277	NA	NA	NA	NA
250	Inject_semaglutide_high_dosage:Oral_semaglutide	0	-0.109770621	NA	-0.109770621	NA	NA	NA	NA
251	Inject_semaglutide_high_dosage:Placebo_or_Control	1	0.405351465	0.405351465	NA	NA	NA	NA	NA
252	Inject_semaglutide_high_dosage:Sotagliflozin	0	2.203116959	NA	2.203116959	NA	NA	NA	NA
253	Inject_semaglutide_high_dosage:Tirzepatide_high_dosage	0	0.704340883	NA	0.704340883	NA	NA	NA	NA
254	Inject_semaglutide_high_dosage:Tirzepatide_low_dosage	0	1.904133264	NA	1.904133264	NA	NA	NA	NA
255	Inject_semaglutide_high_dosage:Tirzepatide_medium_dosage	0	0.918406615	NA	0.918406615	NA	NA	NA	NA
256	Inject_semaglutide_low_dosage:Inject_semaglutide_medium_dosage	1	-0.957614116	-1.091679852	-0.75683662	-0.334843232	-5.39959429	4.729907826	0.896900253
257	Inject_semaglutide_low_dosage:Liraglutide	0	-1.929794437	NA	-1.929794437	NA	NA	NA	NA
258	Inject_semaglutide_low_dosage:Lixisenatide	0	-2.740875086	NA	-2.740875086	NA	NA	NA	NA
259	Inject_semaglutide_low_dosage:Oral_semaglutide	0	-1.057122429	NA	-1.057122429	NA	NA	NA	NA

260	Inject_semaglutide_low_dosage:Placebo_or_Control	1	-0.542000343	-0.408191143	-0.743034375	0.334843232	-4.729907826	5.39959429	0.896900253
261	Inject_semaglutide_low_dosage:Sotagliflozin	0	1.25576515	NA	1.25576515	NA	NA	NA	NA
262	Inject_semaglutide_low_dosage:Tirzepatide_high_dosage	0	-0.243010925	NA	-0.243010925	NA	NA	NA	NA
263	Inject_semaglutide_low_dosage:Tirzepatide_low_dosage	0	0.956781456	NA	0.956781456	NA	NA	NA	NA
264	Inject_semaglutide_low_dosage:Tirzepatide_medium_dosage	0	-0.028945193	NA	-0.028945193	NA	NA	NA	NA
265	Inject_semaglutide_medium_dosage:Liraglutide	0	-0.97218032	NA	-0.97218032	NA	NA	NA	NA
266	Inject_semaglutide_medium_dosage:Lixisenatide	0	-1.783260969	NA	-1.783260969	NA	NA	NA	NA
267	Inject_semaglutide_medium_dosage:Oral_semaglutide	0	-0.099508312	NA	-0.099508312	NA	NA	NA	NA
268	Inject_semaglutide_medium_dosage:Placebo_or_Control	2	0.415613773	0.348645476	0.683488708	-0.334843232	-5.39959429	4.729907826	0.896900253
269	Inject_semaglutide_medium_dosage:Sotagliflozin	0	2.213379267	NA	2.213379267	NA	NA	NA	NA
270	Inject_semaglutide_medium_dosage:Tirzepatide_high_dosage	0	0.714603191	NA	0.714603191	NA	NA	NA	NA
271	Inject_semaglutide_medium_dosage:Tirzepatide_low_dosage	0	1.914395572	NA	1.914395572	NA	NA	NA	NA
272	Inject_semaglutide_medium_dosage:Tirzepatide_medium_dosage	0	0.928668923	NA	0.928668923	NA	NA	NA	NA
273	Liraglutide:Lixisenatide	0	-0.811080649	NA	-0.811080649	NA	NA	NA	NA
274	Liraglutide:Oral_semaglutide	0	0.872672008	NA	0.872672008	NA	NA	NA	NA
275	Liraglutide:Placebo_or_Control	1	1.387794094	1.387794094	NA	NA	NA	NA	NA
276	Liraglutide:Sotagliflozin	0	3.185559587	NA	3.185559587	NA	NA	NA	NA
277	Liraglutide:Tirzepatide_high_dosage	0	1.686783511	NA	1.686783511	NA	NA	NA	NA
278	Liraglutide:Tirzepatide_low_dosage	0	2.886575893	NA	2.886575893	NA	NA	NA	NA
279	Liraglutide:Tirzepatide_medium_dosage	0	1.900849243	NA	1.900849243	NA	NA	NA	NA
280	Lixisenatide:Oral_semaglutide	0	1.683752657	NA	1.683752657	NA	NA	NA	NA
281	Lixisenatide:Placebo_or_Control	1	2.198874743	2.198874743	NA	NA	NA	NA	NA

282	Lixisenatide:Sotagliflozin	0	3.996640236	NA	3.996640236	NA	NA	NA	NA
283	Lixisenatide:Tirzepatide_high_dosage	0	2.497864161	NA	2.497864161	NA	NA	NA	NA
284	Lixisenatide:Tirzepatide_low_dosage	0	3.697656542	NA	3.697656542	NA	NA	NA	NA
285	Lixisenatide:Tirzepatide_medium_dosage	0	2.711929892	NA	2.711929892	NA	NA	NA	NA
286	Oral_semaglutide:Placebo_or_Control	1	0.515122086	0.515122086	NA	NA	NA	NA	NA
287	Oral_semaglutide:Sotagliflozin	0	2.312887579	NA	2.312887579	NA	NA	NA	NA
288	Oral_semaglutide:Tirzepatide_high_dosage	0	0.814111504	NA	0.814111504	NA	NA	NA	NA
289	Oral_semaglutide:Tirzepatide_low_dosage	0	2.013903885	NA	2.013903885	NA	NA	NA	NA
290	Oral_semaglutide:Tirzepatide_medium_dosage	0	1.028177235	NA	1.028177235	NA	NA	NA	NA
291	Sotagliflozin:Placebo_or_Control	1	-1.797765493	-1.797765493	NA	NA	NA	NA	NA
292	Tirzepatide_high_dosage:Placebo_or_Control	2	-0.298989418	-0.013556002	-1.098842812	1.08528681	-3.345491902	5.516065522	0.631171341
293	Tirzepatide_low_dosage:Placebo_or_Control	0	-1.498781799	NA	-1.498781799	NA	NA	NA	NA
294	Tirzepatide_medium_dosage:Placebo_or_Control	1	-0.51305515	-1.08509857	0.00018824	-1.08528681	-5.516065522	3.345491902	0.631171341
295	Sotagliflozin:Tirzepatide_high_dosage	0	-1.498776075	NA	-1.498776075	NA	NA	NA	NA
296	Sotagliflozin:Tirzepatide_low_dosage	0	-0.298983694	NA	-0.298983694	NA	NA	NA	NA
297	Sotagliflozin:Tirzepatide_medium_dosage	0	-1.284710344	NA	-1.284710344	NA	NA	NA	NA
298	Tirzepatide_high_dosage:Tirzepatide_low_dosage	1	1.199792381	1.07318719	1.373726825	-0.300539635	-5.247445036	4.646365766	0.905216882
299	Tirzepatide_high_dosage:Tirzepatide_medium_dosage	2	0.214065732	-0.007434402	0.594597416	-0.602031818	-4.343741934	3.139678299	0.752493411
300	Tirzepatide_low_dosage:Tirzepatide_medium_dosage	1	-0.98572665	-1.112171818	-0.811632183	-0.300539635	-5.247445036	4.646365766	0.905216882

Table S7C: inconsistency within the network meta-analysis of drop-out rate

	Comparison	No.Studies	NMA	Direct	Indirect	Difference	Diff_95CI_lower	Diff_95CI_upper	*p* value
1	Albiglutide:Bexagliflozin	0	0.505969697	NA	0.505969697	NA	NA	NA	NA
2	Albiglutide:Canagliflozin	0	-0.076095453	NA	-0.076095453	NA	NA	NA	NA
3	Albiglutide:Dapagliflozin	0	-0.077821705	NA	-0.077821705	NA	NA	NA	NA
4	Albiglutide:Dulaglutide	0	-0.407927888	NA	-0.407927888	NA	NA	NA	NA
5	Albiglutide:Efpeglenatide	0	-0.313538318	NA	-0.313538318	NA	NA	NA	NA
6	Albiglutide:Empagliflozin	0	-0.205741398	NA	-0.205741398	NA	NA	NA	NA
7	Albiglutide:Ertugliflozin	0	-0.166820364	NA	-0.166820364	NA	NA	NA	NA
8	Albiglutide:Exenatide	0	-0.191025609	NA	-0.191025609	NA	NA	NA	NA
9	Albiglutide:Inject_semaglutide	0	-0.312015396	NA	-0.312015396	NA	NA	NA	NA
10	Albiglutide:Liraglutide	0	-0.198585572	NA	-0.198585572	NA	NA	NA	NA
11	Albiglutide:Lixisenatide	0	-0.288326343	NA	-0.288326343	NA	NA	NA	NA
12	Albiglutide:Oral_semaglutide	0	-0.999753459	NA	-0.999753459	NA	NA	NA	NA
13	Albiglutide:Placebo_or_Control	1	-0.336554885	-0.336554885	NA	NA	NA	NA	NA
14	Albiglutide:Sotagliflozin	0	-0.017479214	NA	-0.017479214	NA	NA	NA	NA
15	Albiglutide:Tirzepatide	0	-0.728314087	NA	-0.728314087	NA	NA	NA	NA
16	Bexagliflozin:Canagliflozin	0	-0.58206515	NA	-0.58206515	NA	NA	NA	NA
17	Bexagliflozin:Dapagliflozin	0	-0.583791402	NA	-0.583791402	NA	NA	NA	NA
18	Bexagliflozin:Dulaglutide	0	-0.913897585	NA	-0.913897585	NA	NA	NA	NA

19	Bexagliflozin:Efpeglenatide	0	-0.819508015	NA	-0.819508015	NA	NA	NA	NA
20	Bexagliflozin:Empagliflozin	0	-0.711711095	NA	-0.711711095	NA	NA	NA	NA
21	Bexagliflozin:Ertugliflozin	0	-0.672790061	NA	-0.672790061	NA	NA	NA	NA
22	Bexagliflozin:Exenatide	0	-0.696995306	NA	-0.696995306	NA	NA	NA	NA
23	Bexagliflozin:Inject_semaglutide	0	-0.817985093	NA	-0.817985093	NA	NA	NA	NA
24	Bexagliflozin:Liraglutide	0	-0.704555269	NA	-0.704555269	NA	NA	NA	NA
25	Bexagliflozin:Lixisenatide	0	-0.79429604	NA	-0.79429604	NA	NA	NA	NA
26	Bexagliflozin:Oral_semaglutide	0	-1.505723156	NA	-1.505723156	NA	NA	NA	NA
27	Bexagliflozin:Placebo_or_Control	1	-0.842524582	-0.842524582	NA	NA	NA	NA	NA
28	Bexagliflozin:Sotagliflozin	0	-0.523448911	NA	-0.523448911	NA	NA	NA	NA
29	Bexagliflozin:Tirzepatide	0	-1.234283784	NA	-1.234283784	NA	NA	NA	NA
30	Canagliflozin:Dapagliflozin	0	-0.001726252	NA	-0.001726252	NA	NA	NA	NA
31	Canagliflozin:Dulaglutide	0	-0.331832434	NA	-0.331832434	NA	NA	NA	NA
32	Canagliflozin:Efpeglenatide	0	-0.237442865	NA	-0.237442865	NA	NA	NA	NA
33	Canagliflozin:Empagliflozin	0	-0.129645945	NA	-0.129645945	NA	NA	NA	NA
34	Canagliflozin:Ertugliflozin	0	-0.090724911	NA	-0.090724911	NA	NA	NA	NA
35	Canagliflozin:Exenatide	0	-0.114930156	NA	-0.114930156	NA	NA	NA	NA
36	Canagliflozin:Inject_semaglutide	0	-0.235919942	NA	-0.235919942	NA	NA	NA	NA
37	Canagliflozin:Liraglutide	0	-0.122490119	NA	-0.122490119	NA	NA	NA	NA
38	Canagliflozin:Lixisenatide	0	-0.21223089	NA	-0.21223089	NA	NA	NA	NA
39	Canagliflozin:Oral_semaglutide	0	-0.923658005	NA	-0.923658005	NA	NA	NA	NA
40	Canagliflozin:Placebo_or_Control	3	-0.260459432	-0.260459432	NA	NA	NA	NA	NA

41	Canagliflozin:Sotagliflozin	0	0.058616239	NA	0.058616239	NA	NA	NA	NA
42	Canagliflozin:Tirzepatide	0	-0.652218634	NA	-0.652218634	NA	NA	NA	NA
43	Dapagliflozin:Dulaglutide	0	-0.330106183	NA	-0.330106183	NA	NA	NA	NA
44	Dapagliflozin:Efpeglenatide	0	-0.235716614	NA	-0.235716614	NA	NA	NA	NA
45	Dapagliflozin:Empagliflozin	0	-0.127919693	NA	-0.127919693	NA	NA	NA	NA
46	Dapagliflozin:Ertugliflozin	0	-0.088998659	NA	-0.088998659	NA	NA	NA	NA
47	Dapagliflozin:Exenatide	0	-0.113203904	NA	-0.113203904	NA	NA	NA	NA
48	Dapagliflozin:Inject_semaglutide	0	-0.234193691	NA	-0.234193691	NA	NA	NA	NA
49	Dapagliflozin:Liraglutide	0	-0.120763867	NA	-0.120763867	NA	NA	NA	NA
50	Dapagliflozin:Lixisenatide	0	-0.210504638	NA	-0.210504638	NA	NA	NA	NA
51	Dapagliflozin:Oral_semaglutide	0	-0.921931754	NA	-0.921931754	NA	NA	NA	NA
52	Dapagliflozin:Placebo_or_Control	2	-0.25873318	-0.25873318	NA	NA	NA	NA	NA
53	Dapagliflozin:Sotagliflozin	0	0.060342491	NA	0.060342491	NA	NA	NA	NA
54	Dapagliflozin:Tirzepatide	0	-0.650492383	NA	-0.650492383	NA	NA	NA	NA
55	Dulaglutide:Efpeglenatide	0	0.094389569	NA	0.094389569	NA	NA	NA	NA
56	Dulaglutide:Empagliflozin	0	0.20218649	NA	0.20218649	NA	NA	NA	NA
57	Dulaglutide:Ertugliflozin	0	0.241107524	NA	0.241107524	NA	NA	NA	NA
58	Dulaglutide:Exenatide	0	0.216902279	NA	0.216902279	NA	NA	NA	NA
59	Dulaglutide:Inject_semaglutide	0	0.095912492	NA	0.095912492	NA	NA	NA	NA
60	Dulaglutide:Liraglutide	0	0.209342316	NA	0.209342316	NA	NA	NA	NA
61	Dulaglutide:Lixisenatide	0	0.119601544	NA	0.119601544	NA	NA	NA	NA
62	Dulaglutide:Oral_semaglutide	0	-0.591825571	NA	-0.591825571	NA	NA	NA	NA

63	Dulaglutide:Placebo_or_Control	2	0.071373003	0.071373003	NA	NA	NA	NA	NA
64	Dulaglutide:Sotagliflozin	0	0.390448674	NA	0.390448674	NA	NA	NA	NA
65	Dulaglutide:Tirzepatide	0	-0.3203862	NA	-0.3203862	NA	NA	NA	NA
66	Efpeglenatide:Empagliflozin	0	0.107796921	NA	0.107796921	NA	NA	NA	NA
67	Efpeglenatide:Ertugliflozin	0	0.146717955	NA	0.146717955	NA	NA	NA	NA
68	Efpeglenatide:Exenatide	0	0.12251271	NA	0.12251271	NA	NA	NA	NA
69	Efpeglenatide:Inject_semaglutide	0	0.001522923	NA	0.001522923	NA	NA	NA	NA
70	Efpeglenatide:Liraglutide	0	0.114952747	NA	0.114952747	NA	NA	NA	NA
71	Efpeglenatide:Lixisenatide	0	0.025211975	NA	0.025211975	NA	NA	NA	NA
72	Efpeglenatide:Oral_semaglutide	0	-0.68621514	NA	-0.68621514	NA	NA	NA	NA
73	Efpeglenatide:Placebo_or_Control	1	-0.023016566	-0.023016566	NA	NA	NA	NA	NA
74	Efpeglenatide:Sotagliflozin	0	0.296059105	NA	0.296059105	NA	NA	NA	NA
75	Efpeglenatide:Tirzepatide	0	-0.414775769	NA	-0.414775769	NA	NA	NA	NA
76	Empagliflozin:Ertugliflozin	0	0.038921034	NA	0.038921034	NA	NA	NA	NA
77	Empagliflozin:Exenatide	0	0.014715789	NA	0.014715789	NA	NA	NA	NA
78	Empagliflozin:Inject_semaglutide	0	-0.106273998	NA	-0.106273998	NA	NA	NA	NA
79	Empagliflozin:Liraglutide	0	0.007155826	NA	0.007155826	NA	NA	NA	NA
80	Empagliflozin:Lixisenatide	0	-0.082584945	NA	-0.082584945	NA	NA	NA	NA
81	Empagliflozin:Oral_semaglutide	0	-0.794012061	NA	-0.794012061	NA	NA	NA	NA
82	Empagliflozin:Placebo_or_Control	5	-0.130813487	-0.130813487	NA	NA	NA	NA	NA
83	Empagliflozin:Sotagliflozin	0	0.188262184	NA	0.188262184	NA	NA	NA	NA
84	Empagliflozin:Tirzepatide	0	-0.52257269	NA	-0.52257269	NA	NA	NA	NA

85	Ertugliflozin:Exenatide	0	-0.024205245	NA	-0.024205245	NA	NA	NA	NA
86	Ertugliflozin:Inject_semaglutide	0	-0.145195032	NA	-0.145195032	NA	NA	NA	NA
87	Ertugliflozin:Liraglutide	0	-0.031765208	NA	-0.031765208	NA	NA	NA	NA
88	Ertugliflozin:Lixisenatide	0	-0.121505979	NA	-0.121505979	NA	NA	NA	NA
89	Ertugliflozin:Oral_semaglutide	0	-0.832933095	NA	-0.832933095	NA	NA	NA	NA
90	Ertugliflozin:Placebo_or_Control	2	-0.169734521	-0.169734521	NA	NA	NA	NA	NA
91	Ertugliflozin:Sotagliflozin	0	0.14934115	NA	0.14934115	NA	NA	NA	NA
92	Ertugliflozin:Tirzepatide	0	-0.561493723	NA	-0.561493723	NA	NA	NA	NA
93	Exenatide:Inject_semaglutide	0	-0.120989787	NA	-0.120989787	NA	NA	NA	NA
94	Exenatide:Liraglutide	0	-0.007559963	NA	-0.007559963	NA	NA	NA	NA
95	Exenatide:Lixisenatide	0	-0.097300734	NA	-0.097300734	NA	NA	NA	NA
96	Exenatide:Oral_semaglutide	0	-0.80872785	NA	-0.80872785	NA	NA	NA	NA
97	Exenatide:Placebo_or_Control	1	-0.145529276	-0.145529276	NA	NA	NA	NA	NA
98	Exenatide:Sotagliflozin	0	0.173546395	NA	0.173546395	NA	NA	NA	NA
99	Exenatide:Tirzepatide	0	-0.537288478	NA	-0.537288478	NA	NA	NA	NA
100	Inject_semaglutide:Liraglutide	0	0.113429824	NA	0.113429824	NA	NA	NA	NA
101	Inject_semaglutide:Lixisenatide	0	0.023689053	NA	0.023689053	NA	NA	NA	NA
102	Inject_semaglutide:Oral_semaglutide	0	-0.687738063	NA	-0.687738063	NA	NA	NA	NA
103	Inject_semaglutide:Placebo_or_Control	3	-0.024539489	-0.024539489	NA	NA	NA	NA	NA
104	Inject_semaglutide:Sotagliflozin	0	0.294536182	NA	0.294536182	NA	NA	NA	NA
105	Inject_semaglutide:Tirzepatide	0	-0.416298692	NA	-0.416298692	NA	NA	NA	NA
106	Liraglutide:Lixisenatide	0	-0.089740771	NA	-0.089740771	NA	NA	NA	NA

107	Liraglutide:Oral_semaglutide	0	-0.801167887	NA	-0.801167887	NA	NA	NA	NA
108	Liraglutide:Placebo_or_Control	1	-0.137969313	-0.137969313	NA	NA	NA	NA	NA
109	Liraglutide:Sotagliflozin	0	0.181106358	NA	0.181106358	NA	NA	NA	NA
110	Liraglutide:Tirzepatide	0	-0.529728515	NA	-0.529728515	NA	NA	NA	NA
111	Lixisenatide:Oral_semaglutide	0	-0.711427116	NA	-0.711427116	NA	NA	NA	NA
112	Lixisenatide:Placebo_or_Control	1	-0.048228542	-0.048228542	NA	NA	NA	NA	NA
113	Lixisenatide:Sotagliflozin	0	0.270847129	NA	0.270847129	NA	NA	NA	NA
114	Lixisenatide:Tirzepatide	0	-0.439987744	NA	-0.439987744	NA	NA	NA	NA
115	Oral_semaglutide:Placebo_or_Control	1	0.663198574	0.663198574	NA	NA	NA	NA	NA
116	Oral_semaglutide:Sotagliflozin	0	0.982274245	NA	0.982274245	NA	NA	NA	NA
117	Oral_semaglutide:Tirzepatide	0	0.271439371	NA	0.271439371	NA	NA	NA	NA
118	Sotagliflozin:Placebo_or_Control	2	-0.319075671	-0.319075671	NA	NA	NA	NA	NA
119	Tirzepatide:Placebo_or_Control	2	0.391759203	0.391759203	NA	NA	NA	NA	NA
120	Sotagliflozin:Tirzepatide	0	-0.710834873	NA	-0.710834873	NA	NA	NA	NA

Abbreviation: 95%CIs: 95% confidence intervals; GLP-1 agonist: glucagon-like peptide-1 agonist; NA: not applicable; NMA: network meta-analysis; OR: odds ratio; RCT: randomized controlled trial; SGLT2 inhibitor: sodium–glucose cotransporter 2 inhibitor

Table S8A: GRADE of primary outcome: incidence of hearing loss

	Comparison	No.Studies	Direct		Indirect		NMA	
			Estimate	Rate	Estimate	Rate	Estimate	Rate
1	Albiglutide:Bexagliflozin	0			-2.702488189	⊕⊕○○ Low	-2.702488189	⊕⊕○○ Low
2	Albiglutide:Canagliflozin	0			-1.026627714	⊕⊕○○ Low	-1.026627714	⊕⊕○○ Low
3	Albiglutide:Dapagliflozin	0			-1.829689142	⊕⊕○○ Low	-1.829689142	⊕⊕○○ Low
4	Albiglutide:Dulaglutide	0			-0.965893446	⊕⊕○○ Low	-0.965893446	⊕⊕○○ Low
5	Albiglutide:Efpeglenatide	0			-2.524550194	⊕⊕○○ Low	-2.524550194	⊕⊕○○ Low
6	Albiglutide:Empagliflozin	0			-1.144270447	⊕⊕○○ Low	-1.144270447	⊕⊕○○ Low
7	Albiglutide:Ertugliflozin	0			-2.021519646	⊕⊕○○ Low	-2.021519646	⊕⊕○○ Low
8	Albiglutide:Exenatide	0			-2.01969392	⊕⊕○○ Low	-2.01969392	⊕⊕○○ Low
9	Albiglutide:Inject_semaglutide	0			-1.727878885	⊕⊕○○ Low	-1.727878885	⊕⊕○○ Low
10	Albiglutide:Liraglutide	0			-2.998080272	⊕⊕○○ Low	-2.998080272	⊕⊕○○ Low
11	Albiglutide:Lixisenatide	0			-3.809160922	⊕⊕○○ Low	-3.809160922	⊕⊕○○ Low
12	Albiglutide:Oral_semaglutide	0			-2.125408265	⊕⊕○○ Low	-2.125408265	⊕⊕○○ Low
13	Albiglutide:Placebo_or_Control	1	-1.610286179	⊕⊕⊕⊕ High			-1.610286179	⊕⊕⊕○ Medium
14	Albiglutide:Sotagliflozin	0			0.187479314	⊕⊕○○ Low	0.187479314	⊕⊕○○ Low
15	Albiglutide:Tirzepatide	0			-0.720693839	⊕⊕○○ Low	-0.720693839	⊕⊕○○ Low
16	Bexagliflozin:Canagliflozin	0			1.675860475	⊕⊕○○ Low	1.675860475	⊕⊕○○ Low
17	Bexagliflozin:Dapagliflozin	0			0.872799047	⊕⊕○○ Low	0.872799047	⊕⊕○○ Low
18	Bexagliflozin:Dulaglutide	0			1.736594743	⊕⊕○○ Low	1.736594743	⊕⊕○○ Low

19	Bexagliflozin:Efpeglenatide	0			0.177937995	⊕⊕○○ Low	0.177937995	⊕⊕○○ Low
20	Bexagliflozin:Empagliflozin	0			1.558217742	⊕⊕○○ Low	1.558217742	⊕⊕○○ Low
21	Bexagliflozin:Ertugliflozin	0			0.680968543	⊕⊕○○ Low	0.680968543	⊕⊕○○ Low
22	Bexagliflozin:Exenatide	0			0.682794269	⊕⊕○○ Low	0.682794269	⊕⊕○○ Low
23	Bexagliflozin:Inject_semaglutide	0			0.974609304	⊕⊕○○ Low	0.974609304	⊕⊕○○ Low
24	Bexagliflozin:Liraglutide	0			-0.295592083	⊕⊕○○ Low	-0.295592083	⊕⊕○○ Low
25	Bexagliflozin:Lixisenatide	0			-1.106672732	⊕⊕○○ Low	-1.106672732	⊕⊕○○ Low
26	Bexagliflozin:Oral_semaglutide	0			0.577079925	⊕⊕○○ Low	0.577079925	⊕⊕○○ Low
27	Bexagliflozin:Placebo_or_Control	1	1.09220201	⊕⊕⊕⊕ High			1.09220201	⊕⊕⊕○ Medium
28	Bexagliflozin:Sotagliflozin	0			2.889967504	⊕⊕○○ Low	2.889967504	⊕⊕○○ Low
29	Bexagliflozin:Tirzepatide	0			1.98179435	⊕⊕○○ Low	1.98179435	⊕⊕○○ Low
30	Canagliflozin:Dapagliflozin	0			-0.803061429	⊕⊕○○ Low	-0.803061429	⊕⊕○○ Low
31	Canagliflozin:Dulaglutide	0			0.060734268	⊕⊕○○ Low	0.060734268	⊕⊕○○ Low
32	Canagliflozin:Efpeglenatide	0			-1.49792248	⊕⊕○○ Low	-1.49792248	⊕⊕○○ Low
33	Canagliflozin:Empagliflozin	0			-0.117642734	⊕⊕○○ Low	-0.117642734	⊕⊕○○ Low
34	Canagliflozin:Ertugliflozin	0			-0.994891932	⊕⊕○○ Low	-0.994891932	⊕⊕○○ Low
35	Canagliflozin:Exenatide	0			-0.993066206	⊕⊕○○ Low	-0.993066206	⊕⊕○○ Low
36	Canagliflozin:Inject_semaglutide	0			-0.701251171	⊕⊕○○ Low	-0.701251171	⊕⊕○○ Low
37	Canagliflozin:Liraglutide	0			-1.971452559	⊕⊕○○ Low	-1.971452559	⊕⊕○○ Low
38	Canagliflozin:Lixisenatide	0			-2.782533208	⊕⊕○○ Low	-2.782533208	⊕⊕○○ Low
39	Canagliflozin:Oral_semaglutide	0			-1.098780551	⊕⊕○○ Low	-1.098780551	⊕⊕○○ Low
40	Canagliflozin:Placebo_or_Control	3	-0.583658465	⊕⊕⊕⊕ High			-0.583658465	⊕⊕⊕○ Medium

41	Canagliflozin:Sotagliflozin	0			1.214107028	⊕⊕○○ Low	1.214107028	⊕⊕○○ Low
42	Canagliflozin:Tirzepatide	0			0.305933875	⊕⊕○○ Low	0.305933875	⊕⊕○○ Low
43	Dapagliflozin:Dulaglutide	0			0.863795696	⊕⊕○○ Low	0.863795696	⊕⊕○○ Low
44	Dapagliflozin:Efpeglenatide	0			-0.694861052	⊕⊕○○ Low	-0.694861052	⊕⊕○○ Low
45	Dapagliflozin:Empagliflozin	0			0.685418695	⊕⊕○○ Low	0.685418695	⊕⊕○○ Low
46	Dapagliflozin:Ertugliflozin	0			-0.191830504	⊕⊕○○ Low	-0.191830504	⊕⊕○○ Low
47	Dapagliflozin:Exenatide	0			-0.190004778	⊕⊕○○ Low	-0.190004778	⊕⊕○○ Low
48	Dapagliflozin:Inject_semaglutide	0			0.101810257	⊕⊕○○ Low	0.101810257	⊕⊕○○ Low
49	Dapagliflozin:Liraglutide	0			-1.16839113	⊕⊕○○ Low	-1.16839113	⊕⊕○○ Low
50	Dapagliflozin:Lixisenatide	0			-1.979471779	⊕⊕○○ Low	-1.979471779	⊕⊕○○ Low
51	Dapagliflozin:Oral_semaglutide	0			-0.295719122	⊕⊕○○ Low	-0.295719122	⊕⊕○○ Low
52	Dapagliflozin:Placebo_or_Control	2	0.219402963	⊕⊕⊕⊕ High			0.219402963	⊕⊕⊕○ Medium
53	Dapagliflozin:Sotagliflozin	0			2.017168457	⊕⊕○○ Low	2.017168457	⊕⊕○○ Low
54	Dapagliflozin:Tirzepatide	0			1.108995303	⊕⊕○○ Low	1.108995303	⊕⊕○○ Low
55	Dulaglutide:Efpeglenatide	0			-1.558656748	⊕⊕○○ Low	-1.558656748	⊕⊕○○ Low
56	Dulaglutide:Empagliflozin	0			-0.178377001	⊕⊕○○ Low	-0.178377001	⊕⊕○○ Low
57	Dulaglutide:Ertugliflozin	0			-1.0556262	⊕⊕○○ Low	-1.0556262	⊕⊕○○ Low
58	Dulaglutide:Exenatide	0			-1.053800474	⊕⊕○○ Low	-1.053800474	⊕⊕○○ Low
59	Dulaglutide:Inject_semaglutide	0			-0.761985439	⊕⊕○○ Low	-0.761985439	⊕⊕○○ Low
60	Dulaglutide:Liraglutide	0			-2.032186826	⊕⊕○○ Low	-2.032186826	⊕⊕○○ Low
61	Dulaglutide:Lixisenatide	0			-2.843267475	⊕⊕○○ Low	-2.843267475	⊕⊕○○ Low
62	Dulaglutide:Oral_semaglutide	0			-1.159514819	⊕⊕○○ Low	-1.159514819	⊕⊕○○ Low

63	Dulaglutide:Placebo_or_Control	2	-0.644392733	⊕⊕⊕⊕ High			-0.644392733	⊕⊕⊕○ Medium
64	Dulaglutide:Sotagliflozin	0			1.153372761	⊕⊕○○ Low	1.153372761	⊕⊕○○ Low
65	Dulaglutide:Tirzepatide	0			0.245199607	⊕⊕○○ Low	0.245199607	⊕⊕○○ Low
66	Efpeglenatide:Empagliflozin	0			1.380279747	⊕⊕○○ Low	1.380279747	⊕⊕○○ Low
67	Efpeglenatide:Ertugliflozin	0			0.503030548	⊕⊕○○ Low	0.503030548	⊕⊕○○ Low
68	Efpeglenatide:Exenatide	0			0.504856274	⊕⊕○○ Low	0.504856274	⊕⊕○○ Low
69	Efpeglenatide:Inject_semaglutide	0			0.796671309	⊕⊕○○ Low	0.796671309	⊕⊕○○ Low
70	Efpeglenatide:Liraglutide	0			-0.473530078	⊕⊕○○ Low	-0.473530078	⊕⊕○○ Low
71	Efpeglenatide:Lixisenatide	0			-1.284610727	⊕⊕○○ Low	-1.284610727	⊕⊕○○ Low
72	Efpeglenatide:Oral_semaglutide	0			0.39914193	⊕⊕○○ Low	0.39914193	⊕⊕○○ Low
73	Efpeglenatide:Placebo_or_Control	1	0.914264015	⊕⊕⊕⊕ High			0.914264015	⊕⊕⊕○ Medium
74	Efpeglenatide:Sotagliflozin	0			2.712029509	⊕⊕○○ Low	2.712029509	⊕⊕○○ Low
75	Efpeglenatide:Tirzepatide	0			1.803856355	⊕⊕○○ Low	1.803856355	⊕⊕○○ Low
76	Empagliflozin:Ertugliflozin	0			-0.877249199	⊕⊕○○ Low	-0.877249199	⊕⊕○○ Low
77	Empagliflozin:Exenatide	0			-0.875423473	⊕⊕○○ Low	-0.875423473	⊕⊕○○ Low
78	Empagliflozin:Inject_semaglutide	0			-0.583608438	⊕⊕○○ Low	-0.583608438	⊕⊕○○ Low
79	Empagliflozin:Liraglutide	0			-1.853809825	⊕⊕○○ Low	-1.853809825	⊕⊕○○ Low
80	Empagliflozin:Lixisenatide	0			-2.664890474	⊕⊕○○ Low	-2.664890474	⊕⊕○○ Low
81	Empagliflozin:Oral_semaglutide	0			-0.981137817	⊕⊕○○ Low	-0.981137817	⊕⊕○○ Low
82	Empagliflozin:Placebo_or_Control	6	-0.466015732	⊕⊕⊕⊕ High			-0.466015732	⊕⊕⊕○ Medium
83	Empagliflozin:Sotagliflozin	0			1.331749762	⊕⊕○○ Low	1.331749762	⊕⊕○○ Low
84	Empagliflozin:Tirzepatide	0			0.423576608	⊕⊕○○ Low	0.423576608	⊕⊕○○ Low

85	Ertugliflozin:Exenatide	0			0.001825726	⊕⊕○○ Low	0.001825726	⊕⊕○○ Low
86	Ertugliflozin:Inject_semaglutide	0			0.293640761	⊕⊕○○ Low	0.293640761	⊕⊕○○ Low
87	Ertugliflozin:Liraglutide	0			-0.976560627	⊕⊕○○ Low	-0.976560627	⊕⊕○○ Low
88	Ertugliflozin:Lixisenatide	0			-1.787641276	⊕⊕○○ Low	-1.787641276	⊕⊕○○ Low
89	Ertugliflozin:Oral_semaglutide	0			-0.103888619	⊕⊕○○ Low	-0.103888619	⊕⊕○○ Low
90	Ertugliflozin:Placebo_or_Control	2	0.411233467	⊕⊕⊕⊕ High			0.411233467	⊕⊕⊕○ Medium
91	Ertugliflozin:Sotagliflozin	0			2.20899896	⊕⊕○○ Low	2.20899896	⊕⊕○○ Low
92	Ertugliflozin:Tirzepatide	0			1.300825807	⊕⊕○○ Low	1.300825807	⊕⊕○○ Low
93	Exenatide:Inject_semaglutide	0			0.291815035	⊕⊕○○ Low	0.291815035	⊕⊕○○ Low
94	Exenatide:Liraglutide	0			-0.978386352	⊕⊕○○ Low	-0.978386352	⊕⊕○○ Low
95	Exenatide:Lixisenatide	0			-1.789467002	⊕⊕○○ Low	-1.789467002	⊕⊕○○ Low
96	Exenatide:Oral_semaglutide	0			-0.105714345	⊕⊕○○ Low	-0.105714345	⊕⊕○○ Low
97	Exenatide:Placebo_or_Control	1	0.409407741	⊕⊕⊕⊕ High			0.409407741	⊕⊕⊕○ Medium
98	Exenatide:Sotagliflozin	0			2.207173234	⊕⊕○○ Low	2.207173234	⊕⊕○○ Low
99	Exenatide:Tirzepatide	0			1.299000081	⊕⊕○○ Low	1.299000081	⊕⊕○○ Low
100	Inject_semaglutide:Liraglutide	0			-1.270201388	⊕⊕○○ Low	-1.270201388	⊕⊕○○ Low
101	Inject_semaglutide:Lixisenatide	0			-2.081282037	⊕⊕○○ Low	-2.081282037	⊕⊕○○ Low
102	Inject_semaglutide:Oral_semaglutide	0			-0.39752938	⊕⊕○○ Low	-0.39752938	⊕⊕○○ Low
103	Inject_semaglutide:Placebo_or_Control	3	0.117592706	⊕⊕⊕⊕ High			0.117592706	⊕⊕⊕○ Medium
104	Inject_semaglutide:Sotagliflozin	0			1.915358199	⊕⊕○○ Low	1.915358199	⊕⊕○○ Low
105	Inject_semaglutide:Tirzepatide	0			1.007185046	⊕⊕○○ Low	1.007185046	⊕⊕○○ Low
106	Liraglutide:Lixisenatide	0			-0.811080649	⊕⊕○○ Low	-0.811080649	⊕⊕○○ Low

107	Liraglutide:Oral_semaglutide	0			0.872672008	⊕⊕○○ Low	0.872672008	⊕⊕○○ Low
108	Liraglutide:Placebo_or_Control	1	1.387794094	⊕⊕⊕⊕ High			1.387794094	⊕⊕⊕○ Medium
109	Liraglutide:Sotagliflozin	0			3.185559587	⊕⊕○○ Low	3.185559587	⊕⊕○○ Low
110	Liraglutide:Tirzepatide	0			2.277386433	⊕⊕○○ Low	2.277386433	⊕⊕○○ Low
111	Lixisenatide:Oral_semaglutide	0			1.683752657	⊕⊕○○ Low	1.683752657	⊕⊕○○ Low
112	Lixisenatide:Placebo_or_Control	1	2.198874743	⊕⊕⊕⊕ High			2.198874743	⊕⊕⊕○ Medium
113	Lixisenatide:Sotagliflozin	0			3.996640236	⊕⊕○○ Low	3.996640236	⊕⊕○○ Low
114	Lixisenatide:Tirzepatide	0			3.088467083	⊕⊕○○ Low	3.088467083	⊕⊕○○ Low
115	Oral_semaglutide:Placebo_or_Control	1	0.515122086	⊕⊕⊕⊕ High			0.515122086	⊕⊕⊕○ Medium
116	Oral_semaglutide:Sotagliflozin	0			2.312887579	⊕⊕○○ Low	2.312887579	⊕⊕○○ Low
117	Oral_semaglutide:Tirzepatide	0			1.404714426	⊕⊕○○ Low	1.404714426	⊕⊕○○ Low
118	Sotagliflozin:Placebo_or_Control	1	-1.797765493	⊕⊕⊕⊕ High			-1.797765493	⊕⊕⊕○ Medium
119	Tirzepatide:Placebo_or_Control	2	-0.88959234	⊕⊕⊕⊕ High			-0.88959234	⊕⊕⊕○ Medium
120	Sotagliflozin:Tirzepatide	0			-0.908173153	⊕⊕○○ Low	-0.908173153	⊕⊕○○ Low

Table S8B: GRADE of primary outcome: incidence of hearing loss in subgroup of dosage

	Comparison	No.Studies	Direct		Indirect		NMA	
			Estimate	Rate	Estimate	Rate	Estimate	Rate
1	Albiglutide:Bexagliflozin	0			-2.702488189	⊕⊕○○ Low	-2.702488189	⊕⊕○○ Low
2	Albiglutide:Canagliflozin_high_dosage	0			-1.028101095	⊕⊕○○ Low	-1.028101095	⊕⊕○○ Low
3	Albiglutide:Canagliflozin_low_dosage	0			-1.02515165	⊕⊕○○ Low	-1.02515165	⊕⊕○○ Low
4	Albiglutide:Dapagliflozin	0			-1.829689142	⊕⊕○○ Low	-1.829689142	⊕⊕○○ Low
5	Albiglutide:Dulaglutide_high_dosage	0			-1.10938717	⊕⊕○○ Low	-1.10938717	⊕⊕○○ Low
6	Albiglutide:Dulaglutide_low_dosage	0			-0.010774881	⊕⊕○○ Low	-0.010774881	⊕⊕○○ Low
7	Albiglutide:Efpeglenatide_high_dosage	0			-3.218986628	⊕⊕○○ Low	-3.218986628	⊕⊕○○ Low
8	Albiglutide:Efpeglenatide_low_dosage	0			-1.60660429	⊕⊕○○ Low	-1.60660429	⊕⊕○○ Low
9	Albiglutide:Empagliflozin_high_dosage	0			-1.064754584	⊕⊕○○ Low	-1.064754584	⊕⊕○○ Low
10	Albiglutide:Empagliflozin_low_dosage	0			-1.153206779	⊕⊕○○ Low	-1.153206779	⊕⊕○○ Low
11	Albiglutide:Ertugliflozin_high_dosage	0			-2.149792434	⊕⊕○○ Low	-2.149792434	⊕⊕○○ Low
12	Albiglutide:Ertugliflozin_low_dosage	0			-2.452023508	⊕⊕○○ Low	-2.452023508	⊕⊕○○ Low
13	Albiglutide:Exenatide	0			-2.01969392	⊕⊕○○ Low	-2.01969392	⊕⊕○○ Low
14	Albiglutide:Inject_semaglutide_high_dosage	0			-2.015637644	⊕⊕○○ Low	-2.015637644	⊕⊕○○ Low
15	Albiglutide:Inject_semaglutide_low_dosage	0			-1.068285836	⊕⊕○○ Low	-1.068285836	⊕⊕○○ Low
16	Albiglutide:Inject_semaglutide_medium_dosage	0			-2.025899952	⊕⊕○○ Low	-2.025899952	⊕⊕○○ Low
17	Albiglutide:Liraglutide	0			-2.998080272	⊕⊕○○ Low	-2.998080272	⊕⊕○○ Low
18	Albiglutide:Lixisenatide	0			-3.809160922	⊕⊕○○ Low	-3.809160922	⊕⊕○○ Low

19	Albiglutide:Oral_semaglutide	0			-2.125408265	⊕⊕○○ Low	-2.125408265	⊕⊕○○ Low
20	Albiglutide:Placebo_or_Control	1	-1.610286179	⊕⊕⊕⊕ High			-1.610286179	⊕⊕⊕○ Medium
21	Albiglutide:Sotagliflozin	0			0.187479314	⊕⊕○○ Low	0.187479314	⊕⊕○○ Low
22	Albiglutide:Tirzepatide_high_dosage	0			-1.311296761	⊕⊕○○ Low	-1.311296761	⊕⊕○○ Low
23	Albiglutide:Tirzepatide_low_dosage	0			-0.11150438	⊕⊕○○ Low	-0.11150438	⊕⊕○○ Low
24	Albiglutide:Tirzepatide_medium_dosage	0			-1.097231029	⊕⊕○○ Low	-1.097231029	⊕⊕○○ Low
25	Bexagliflozin:Canagliflozin_high_dosage	0			1.674387094	⊕⊕○○ Low	1.674387094	⊕⊕○○ Low
26	Bexagliflozin:Canagliflozin_low_dosage	0			1.677336539	⊕⊕○○ Low	1.677336539	⊕⊕○○ Low
27	Bexagliflozin:Dapagliflozin	0			0.872799047	⊕⊕○○ Low	0.872799047	⊕⊕○○ Low
28	Bexagliflozin:Dulaglutide_high_dosage	0			1.593101019	⊕⊕○○ Low	1.593101019	⊕⊕○○ Low
29	Bexagliflozin:Dulaglutide_low_dosage	0			2.691713308	⊕⊕○○ Low	2.691713308	⊕⊕○○ Low
30	Bexagliflozin:Efpeglenatide_high_dosage	0			-0.516498439	⊕⊕○○ Low	-0.516498439	⊕⊕○○ Low
31	Bexagliflozin:Efpeglenatide_low_dosage	0			1.0958839	⊕⊕○○ Low	1.0958839	⊕⊕○○ Low
32	Bexagliflozin:Empagliflozin_high_dosage	0			1.637733605	⊕⊕○○ Low	1.637733605	⊕⊕○○ Low
33	Bexagliflozin:Empagliflozin_low_dosage	0			1.54928141	⊕⊕○○ Low	1.54928141	⊕⊕○○ Low
34	Bexagliflozin:Ertugliflozin_high_dosage	0			0.552695755	⊕⊕○○ Low	0.552695755	⊕⊕○○ Low
35	Bexagliflozin:Ertugliflozin_low_dosage	0			0.250464681	⊕⊕○○ Low	0.250464681	⊕⊕○○ Low
36	Bexagliflozin:Exenatide	0			0.682794269	⊕⊕○○ Low	0.682794269	⊕⊕○○ Low
37	Bexagliflozin:Inject_semaglutide_high_dosage	0			0.686850545	⊕⊕○○ Low	0.686850545	⊕⊕○○ Low
38	Bexagliflozin:Inject_semaglutide_low_dosage	0			1.634202353	⊕⊕○○ Low	1.634202353	⊕⊕○○ Low
39	Bexagliflozin:Inject_semaglutide_medium_dosage	0			0.676588237	⊕⊕○○ Low	0.676588237	⊕⊕○○ Low
40	Bexagliflozin:Liraglutide	0			-0.295592083	⊕⊕○○ Low	-0.295592083	⊕⊕○○ Low

41	Bexagliflozin:Lixisenatide	0			-1.106672732	⊕⊕○○ Low	-1.106672732	⊕⊕○○ Low
42	Bexagliflozin:Oral_semaglutide	0			0.577079925	⊕⊕○○ Low	0.577079925	⊕⊕○○ Low
43	Bexagliflozin:Placebo_or_Control	1	1.09220201	⊕⊕⊕⊕ High			1.09220201	⊕⊕⊕○ Medium
44	Bexagliflozin:Sotagliflozin	0			2.889967504	⊕⊕○○ Low	2.889967504	⊕⊕○○ Low
45	Bexagliflozin:Tirzepatide_high_dosage	0			1.391191428	⊕⊕○○ Low	1.391191428	⊕⊕○○ Low
46	Bexagliflozin:Tirzepatide_low_dosage	0			2.590983809	⊕⊕○○ Low	2.590983809	⊕⊕○○ Low
47	Bexagliflozin:Tirzepatide_medium_dosage	0			1.60525716	⊕⊕○○ Low	1.60525716	⊕⊕○○ Low
48	Canagliflozin_high_dosage:Canagliflozin_low_dosage	1	0.002773927	⊕⊕⊕⊕ High	0.003418369	⊕⊕⊕⊕ High	0.002949446	⊕⊕⊕⊕ High
49	Canagliflozin_high_dosage:Dapagliflozin	0			-0.801588047	⊕⊕○○ Low	-0.801588047	⊕⊕○○ Low
50	Canagliflozin_high_dosage:Dulaglutide_high_dosage	0			-0.081286074	⊕⊕○○ Low	-0.081286074	⊕⊕○○ Low
51	Canagliflozin_high_dosage:Dulaglutide_low_dosage	0			1.017326214	⊕⊕○○ Low	1.017326214	⊕⊕○○ Low
52	Canagliflozin_high_dosage:Efpeglenatide_high_dosage	0			-2.190885533	⊕⊕○○ Low	-2.190885533	⊕⊕○○ Low
53	Canagliflozin_high_dosage:Efpeglenatide_low_dosage	0			-0.578503194	⊕⊕○○ Low	-0.578503194	⊕⊕○○ Low
54	Canagliflozin_high_dosage:Empagliflozin_high_dosage	0			-0.036653489	⊕⊕○○ Low	-0.036653489	⊕⊕○○ Low
55	Canagliflozin_high_dosage:Empagliflozin_low_dosage	0			-0.125105684	⊕⊕○○ Low	-0.125105684	⊕⊕○○ Low
56	Canagliflozin_high_dosage:Ertugliflozin_high_dosage	0			-1.121691339	⊕⊕○○ Low	-1.121691339	⊕⊕○○ Low
57	Canagliflozin_high_dosage:Ertugliflozin_low_dosage	0			-1.423922412	⊕⊕○○ Low	-1.423922412	⊕⊕○○ Low
58	Canagliflozin_high_dosage:Exenatide	0			-0.991592825	⊕⊕○○ Low	-0.991592825	⊕⊕○○ Low
59	Canagliflozin_high_dosage:Inject_semaglutide_high_dosage	0			-0.987536549	⊕⊕○○ Low	-0.987536549	⊕⊕○○ Low
60	Canagliflozin_high_dosage:Inject_semaglutide_low_dosage	0			-0.040184741	⊕⊕○○ Low	-0.040184741	⊕⊕○○ Low
61	Canagliflozin_high_dosage:Inject_semaglutide_medium_dosage	0			-0.997798857	⊕⊕○○ Low	-0.997798857	⊕⊕○○ Low
62	Canagliflozin_high_dosage:Liraglutide	0			-1.969979177	⊕⊕○○ Low	-1.969979177	⊕⊕○○ Low

63	Canagliflozin_high_dosage:Lixisenatide	0			-2.781059826	⊕⊕○○ Low	-2.781059826	⊕⊕○○ Low
64	Canagliflozin_high_dosage:Oral_semaglutide	0			-1.097307169	⊕⊕○○ Low	-1.097307169	⊕⊕○○ Low
65	Canagliflozin_high_dosage:Placebo_or_Control	2	-0.471246411	⊕⊕⊕⊕ High	-2.204663782	⊕⊕⊕⊕ High	-0.582185083	⊕⊕⊕⊕ High
66	Canagliflozin_high_dosage:Sotagliflozin	0			1.21558041	⊕⊕○○ Low	1.21558041	⊕⊕○○ Low
67	Canagliflozin_high_dosage:Tirzepatide_high_dosage	0			-0.283195666	⊕⊕○○ Low	-0.283195666	⊕⊕○○ Low
68	Canagliflozin_high_dosage:Tirzepatide_low_dosage	0			0.916596716	⊕⊕○○ Low	0.916596716	⊕⊕○○ Low
69	Canagliflozin_high_dosage:Tirzepatide_medium_dosage	0			-0.069129934	⊕⊕○○ Low	-0.069129934	⊕⊕○○ Low
70	Canagliflozin_low_dosage:Dapagliflozin	0			-0.804537493	⊕⊕○○ Low	-0.804537493	⊕⊕○○ Low
71	Canagliflozin_low_dosage:Dulaglutide_high_dosage	0			-0.08423552	⊕⊕○○ Low	-0.08423552	⊕⊕○○ Low
72	Canagliflozin_low_dosage:Dulaglutide_low_dosage	0			1.014376769	⊕⊕○○ Low	1.014376769	⊕⊕○○ Low
73	Canagliflozin_low_dosage:Efpeglenatide_high_dosage	0			-2.193834978	⊕⊕○○ Low	-2.193834978	⊕⊕○○ Low
74	Canagliflozin_low_dosage:Efpeglenatide_low_dosage	0			-0.58145264	⊕⊕○○ Low	-0.58145264	⊕⊕○○ Low
75	Canagliflozin_low_dosage:Empagliflozin_high_dosage	0			-0.039602934	⊕⊕○○ Low	-0.039602934	⊕⊕○○ Low
76	Canagliflozin_low_dosage:Empagliflozin_low_dosage	0			-0.12805513	⊕⊕○○ Low	-0.12805513	⊕⊕○○ Low
77	Canagliflozin_low_dosage:Ertugliflozin_high_dosage	0			-1.124640784	⊕⊕○○ Low	-1.124640784	⊕⊕○○ Low
78	Canagliflozin_low_dosage:Ertugliflozin_low_dosage	0			-1.426871858	⊕⊕○○ Low	-1.426871858	⊕⊕○○ Low
79	Canagliflozin_low_dosage:Exenatide	0			-0.99454227	⊕⊕○○ Low	-0.99454227	⊕⊕○○ Low
80	Canagliflozin_low_dosage:Inject_semaglutide_high_dosage	0			-0.990485994	⊕⊕○○ Low	-0.990485994	⊕⊕○○ Low
81	Canagliflozin_low_dosage:Inject_semaglutide_low_dosage	0			-0.043134186	⊕⊕○○ Low	-0.043134186	⊕⊕○○ Low
82	Canagliflozin_low_dosage:Inject_semaglutide_medium_dosage	0			-1.000748302	⊕⊕○○ Low	-1.000748302	⊕⊕○○ Low
83	Canagliflozin_low_dosage:Liraglutide	0			-1.972928623	⊕⊕○○ Low	-1.972928623	⊕⊕○○ Low
84	Canagliflozin_low_dosage:Lixisenatide	0			-2.784009272	⊕⊕○○ Low	-2.784009272	⊕⊕○○ Low

85	Canagliflozin_low_dosage:Oral_semaglutide	0			-1.100256615	⊕⊕○○ Low	-1.100256615	⊕⊕○○ Low
86	Canagliflozin_low_dosage:Placebo_or_Control	2	-0.474077806	⊕⊕⊕⊕ High	-2.201374039	⊕⊕⊕⊕ High	-0.585134529	⊕⊕⊕⊕ High
87	Canagliflozin_low_dosage:Sotagliflozin	0			1.212630964	⊕⊕○○ Low	1.212630964	⊕⊕○○ Low
88	Canagliflozin_low_dosage:Tirzepatide_high_dosage	0			-0.286145111	⊕⊕○○ Low	-0.286145111	⊕⊕○○ Low
89	Canagliflozin_low_dosage:Tirzepatide_low_dosage	0			0.91364727	⊕⊕○○ Low	0.91364727	⊕⊕○○ Low
90	Canagliflozin_low_dosage:Tirzepatide_medium_dosage	0			-0.07207938	⊕⊕○○ Low	-0.07207938	⊕⊕○○ Low
91	Dapagliflozin:Dulaglutide_high_dosage	0			0.720301972	⊕⊕○○ Low	0.720301972	⊕⊕○○ Low
92	Dapagliflozin:Dulaglutide_low_dosage	0			1.818914261	⊕⊕○○ Low	1.818914261	⊕⊕○○ Low
93	Dapagliflozin:Efpeglenatide_high_dosage	0			-1.389297486	⊕⊕○○ Low	-1.389297486	⊕⊕○○ Low
94	Dapagliflozin:Efpeglenatide_low_dosage	0			0.223084853	⊕⊕○○ Low	0.223084853	⊕⊕○○ Low
95	Dapagliflozin:Empagliflozin_high_dosage	0			0.764934558	⊕⊕○○ Low	0.764934558	⊕⊕○○ Low
96	Dapagliflozin:Empagliflozin_low_dosage	0			0.676482363	⊕⊕○○ Low	0.676482363	⊕⊕○○ Low
97	Dapagliflozin:Ertugliflozin_high_dosage	0			-0.320103292	⊕⊕○○ Low	-0.320103292	⊕⊕○○ Low
98	Dapagliflozin:Ertugliflozin_low_dosage	0			-0.622334365	⊕⊕○○ Low	-0.622334365	⊕⊕○○ Low
99	Dapagliflozin:Exenatide	0			-0.190004778	⊕⊕○○ Low	-0.190004778	⊕⊕○○ Low
100	Dapagliflozin:Inject_semaglutide_high_dosage	0			-0.185948502	⊕⊕○○ Low	-0.185948502	⊕⊕○○ Low
101	Dapagliflozin:Inject_semaglutide_low_dosage	0			0.761403306	⊕⊕○○ Low	0.761403306	⊕⊕○○ Low
102	Dapagliflozin:Inject_semaglutide_medium_dosage	0			-0.19621081	⊕⊕○○ Low	-0.19621081	⊕⊕○○ Low
103	Dapagliflozin:Liraglutide	0			-1.16839113	⊕⊕○○ Low	-1.16839113	⊕⊕○○ Low
104	Dapagliflozin:Lixisenatide	0			-1.979471779	⊕⊕○○ Low	-1.979471779	⊕⊕○○ Low
105	Dapagliflozin:Oral_semaglutide	0			-0.295719122	⊕⊕○○ Low	-0.295719122	⊕⊕○○ Low
106	Dapagliflozin:Placebo_or_Control	2	0.219402963	⊕⊕⊕⊕ High			0.219402963	⊕⊕⊕○ Medium

107	Dapagliflozin:Sotagliflozin	0			2.017168457	⊕⊕○○ Low	2.017168457	⊕⊕○○ Low
108	Dapagliflozin:Tirzepatide_high_dosage	0			0.518392381	⊕⊕○○ Low	0.518392381	⊕⊕○○ Low
109	Dapagliflozin:Tirzepatide_low_dosage	0			1.718184763	⊕⊕○○ Low	1.718184763	⊕⊕○○ Low
110	Dapagliflozin:Tirzepatide_medium_dosage	0			0.732458113	⊕⊕○○ Low	0.732458113	⊕⊕○○ Low
111	Dulaglutide_high_dosage:Dulaglutide_low_dosage	1	1.098612289	⊕⊕⊕⊕ High			1.098612289	⊕⊕⊕○ Medium
112	Dulaglutide_high_dosage:Efpeglenatide_high_dosage	0			-2.109599458	⊕⊕○○ Low	-2.109599458	⊕⊕○○ Low
113	Dulaglutide_high_dosage:Efpeglenatide_low_dosage	0			-0.49721712	⊕⊕○○ Low	-0.49721712	⊕⊕○○ Low
114	Dulaglutide_high_dosage:Empagliflozin_high_dosage	0			0.044632586	⊕⊕○○ Low	0.044632586	⊕⊕○○ Low
115	Dulaglutide_high_dosage:Empagliflozin_low_dosage	0			-0.043819609	⊕⊕○○ Low	-0.043819609	⊕⊕○○ Low
116	Dulaglutide_high_dosage:Ertugliflozin_high_dosage	0			-1.040405264	⊕⊕○○ Low	-1.040405264	⊕⊕○○ Low
117	Dulaglutide_high_dosage:Ertugliflozin_low_dosage	0			-1.342636338	⊕⊕○○ Low	-1.342636338	⊕⊕○○ Low
118	Dulaglutide_high_dosage:Exenatide	0			-0.91030675	⊕⊕○○ Low	-0.91030675	⊕⊕○○ Low
119	Dulaglutide_high_dosage:Inject_semaglutide_high_dosage	0			-0.906250474	⊕⊕○○ Low	-0.906250474	⊕⊕○○ Low
120	Dulaglutide_high_dosage:Inject_semaglutide_low_dosage	0			0.041101334	⊕⊕○○ Low	0.041101334	⊕⊕○○ Low
121	Dulaglutide_high_dosage:Inject_semaglutide_medium_dosage	0			-0.916512782	⊕⊕○○ Low	-0.916512782	⊕⊕○○ Low
122	Dulaglutide_high_dosage:Liraglutide	0			-1.888693103	⊕⊕○○ Low	-1.888693103	⊕⊕○○ Low
123	Dulaglutide_high_dosage:Lixisenatide	0			-2.699773752	⊕⊕○○ Low	-2.699773752	⊕⊕○○ Low
124	Dulaglutide_high_dosage:Oral_semaglutide	0			-1.016021095	⊕⊕○○ Low	-1.016021095	⊕⊕○○ Low
125	Dulaglutide_high_dosage:Placebo_or_Control	2	-0.500899009	⊕⊕⊕⊕ High			-0.500899009	⊕⊕⊕○ Medium
126	Dulaglutide_high_dosage:Sotagliflozin	0			1.296866484	⊕⊕○○ Low	1.296866484	⊕⊕○○ Low
127	Dulaglutide_high_dosage:Tirzepatide_high_dosage	0			-0.201909591	⊕⊕○○ Low	-0.201909591	⊕⊕○○ Low
128	Dulaglutide_high_dosage:Tirzepatide_low_dosage	0			0.99788279	⊕⊕○○ Low	0.99788279	⊕⊕○○ Low

129	Dulaglutide_high_dosage:Tirzepatide_medium_dosage	0			0.012156141	⊕⊕○○ Low	0.012156141	⊕⊕○○ Low
130	Dulaglutide_low_dosage:Efpeglenatide_high_dosage	0			-3.208211747	⊕⊕○○ Low	-3.208211747	⊕⊕○○ Low
131	Dulaglutide_low_dosage:Efpeglenatide_low_dosage	0			-1.595829408	⊕⊕○○ Low	-1.595829408	⊕⊕○○ Low
132	Dulaglutide_low_dosage:Empagliflozin_high_dosage	0			-1.053979703	⊕⊕○○ Low	-1.053979703	⊕⊕○○ Low
133	Dulaglutide_low_dosage:Empagliflozin_low_dosage	0			-1.142431898	⊕⊕○○ Low	-1.142431898	⊕⊕○○ Low
134	Dulaglutide_low_dosage:Ertugliflozin_high_dosage	0			-2.139017553	⊕⊕○○ Low	-2.139017553	⊕⊕○○ Low
135	Dulaglutide_low_dosage:Ertugliflozin_low_dosage	0			-2.441248627	⊕⊕○○ Low	-2.441248627	⊕⊕○○ Low
136	Dulaglutide_low_dosage:Exenatide	0			-2.008919039	⊕⊕○○ Low	-2.008919039	⊕⊕○○ Low
137	Dulaglutide_low_dosage:Inject_semaglutide_high_dosage	0			-2.004862763	⊕⊕○○ Low	-2.004862763	⊕⊕○○ Low
138	Dulaglutide_low_dosage:Inject_semaglutide_low_dosage	0			-1.057510955	⊕⊕○○ Low	-1.057510955	⊕⊕○○ Low
139	Dulaglutide_low_dosage:Inject_semaglutide_medium_dosage	0			-2.015125071	⊕⊕○○ Low	-2.015125071	⊕⊕○○ Low
140	Dulaglutide_low_dosage:Liraglutide	0			-2.987305391	⊕⊕○○ Low	-2.987305391	⊕⊕○○ Low
141	Dulaglutide_low_dosage:Lixisenatide	0			-3.79838604	⊕⊕○○ Low	-3.79838604	⊕⊕○○ Low
142	Dulaglutide_low_dosage:Oral_semaglutide	0			-2.114633383	⊕⊕○○ Low	-2.114633383	⊕⊕○○ Low
143	Dulaglutide_low_dosage:Placebo_or_Control	0			-1.599511298	⊕⊕○○ Low	-1.599511298	⊕⊕○○ Low
144	Dulaglutide_low_dosage:Sotagliflozin	0			0.198254196	⊕⊕○○ Low	0.198254196	⊕⊕○○ Low
145	Dulaglutide_low_dosage:Tirzepatide_high_dosage	0			-1.30052188	⊕⊕○○ Low	-1.30052188	⊕⊕○○ Low
146	Dulaglutide_low_dosage:Tirzepatide_low_dosage	0			-0.100729499	⊕⊕○○ Low	-0.100729499	⊕⊕○○ Low
147	Dulaglutide_low_dosage:Tirzepatide_medium_dosage	0			-1.086456148	⊕⊕○○ Low	-1.086456148	⊕⊕○○ Low
148	Efpeglenatide_high_dosage:Efpeglenatide_low_dosage	1	1.612382339	⊕⊕⊕⊕ High			1.612382339	⊕⊕⊕○ Medium
149	Efpeglenatide_high_dosage:Empagliflozin_high_dosage	0			2.154232044	⊕⊕○○ Low	2.154232044	⊕⊕○○ Low
150	Efpeglenatide_high_dosage:Empagliflozin_low_dosage	0			2.065779849	⊕⊕○○ Low	2.065779849	⊕⊕○○ Low

151	Efpeglenatide_high_dosage:Ertugliflozin_high_dosage	0			1.069194194	⊕⊕○○ Low	1.069194194	⊕⊕○○ Low
152	Efpeglenatide_high_dosage:Ertugliflozin_low_dosage	0			0.76696312	⊕⊕○○ Low	0.76696312	⊕⊕○○ Low
153	Efpeglenatide_high_dosage:Exenatide	0			1.199292708	⊕⊕○○ Low	1.199292708	⊕⊕○○ Low
154	Efpeglenatide_high_dosage:Inject_semaglutide_high_dosage	0			1.203348984	⊕⊕○○ Low	1.203348984	⊕⊕○○ Low
155	Efpeglenatide_high_dosage:Inject_semaglutide_low_dosage	0			2.150700792	⊕⊕○○ Low	2.150700792	⊕⊕○○ Low
156	Efpeglenatide_high_dosage:Inject_semaglutide_medium_dosage	0			1.193086676	⊕⊕○○ Low	1.193086676	⊕⊕○○ Low
157	Efpeglenatide_high_dosage:Liraglutide	0			0.220906356	⊕⊕○○ Low	0.220906356	⊕⊕○○ Low
158	Efpeglenatide_high_dosage:Lixisenatide	0			-0.590174293	⊕⊕○○ Low	-0.590174293	⊕⊕○○ Low
159	Efpeglenatide_high_dosage:Oral_semaglutide	0			1.093578363	⊕⊕○○ Low	1.093578363	⊕⊕○○ Low
160	Efpeglenatide_high_dosage:Placebo_or_Control	1	1.608700449	⊕⊕⊕⊕ High			1.608700449	⊕⊕⊕○ Medium
161	Efpeglenatide_high_dosage:Sotagliflozin	0			3.406465943	⊕⊕○○ Low	3.406465943	⊕⊕○○ Low
162	Efpeglenatide_high_dosage:Tirzepatide_high_dosage	0			1.907689867	⊕⊕○○ Low	1.907689867	⊕⊕○○ Low
163	Efpeglenatide_high_dosage:Tirzepatide_low_dosage	0			3.107482248	⊕⊕○○ Low	3.107482248	⊕⊕○○ Low
164	Efpeglenatide_high_dosage:Tirzepatide_medium_dosage	0			2.121755599	⊕⊕○○ Low	2.121755599	⊕⊕○○ Low
165	Efpeglenatide_low_dosage:Empagliflozin_high_dosage	0			0.541849705	⊕⊕○○ Low	0.541849705	⊕⊕○○ Low
166	Efpeglenatide_low_dosage:Empagliflozin_low_dosage	0			0.45339751	⊕⊕○○ Low	0.45339751	⊕⊕○○ Low
167	Efpeglenatide_low_dosage:Ertugliflozin_high_dosage	0			-0.543188145	⊕⊕○○ Low	-0.543188145	⊕⊕○○ Low
168	Efpeglenatide_low_dosage:Ertugliflozin_low_dosage	0			-0.845419218	⊕⊕○○ Low	-0.845419218	⊕⊕○○ Low
169	Efpeglenatide_low_dosage:Exenatide	0			-0.41308963	⊕⊕○○ Low	-0.41308963	⊕⊕○○ Low
170	Efpeglenatide_low_dosage:Inject_semaglutide_high_dosage	0			-0.409033355	⊕⊕○○ Low	-0.409033355	⊕⊕○○ Low
171	Efpeglenatide_low_dosage:Inject_semaglutide_low_dosage	0			0.538318454	⊕⊕○○ Low	0.538318454	⊕⊕○○ Low
172	Efpeglenatide_low_dosage:Inject_semaglutide_medium_dosage	0			-0.419295663	⊕⊕○○ Low	-0.419295663	⊕⊕○○ Low

173	Efpeglenatide_low_dosage:Liraglutide	0			-1.391475983	⊕⊕○○ Low	-1.391475983	⊕⊕○○ Low
174	Efpeglenatide_low_dosage:Lixisenatide	0			-2.202556632	⊕⊕○○ Low	-2.202556632	⊕⊕○○ Low
175	Efpeglenatide_low_dosage:Oral_semaglutide	0			-0.518803975	⊕⊕○○ Low	-0.518803975	⊕⊕○○ Low
176	Efpeglenatide_low_dosage:Placebo_or_Control	0			-0.003681889	⊕⊕○○ Low	-0.003681889	⊕⊕○○ Low
177	Efpeglenatide_low_dosage:Sotagliflozin	0			1.794083604	⊕⊕○○ Low	1.794083604	⊕⊕○○ Low
178	Efpeglenatide_low_dosage:Tirzepatide_high_dosage	0			0.295307529	⊕⊕○○ Low	0.295307529	⊕⊕○○ Low
179	Efpeglenatide_low_dosage:Tirzepatide_low_dosage	0			1.49509991	⊕⊕○○ Low	1.49509991	⊕⊕○○ Low
180	Efpeglenatide_low_dosage:Tirzepatide_medium_dosage	0			0.50937326	⊕⊕○○ Low	0.50937326	⊕⊕○○ Low
181	Empagliflozin_high_dosage:Empagliflozin_low_dosage	1	0.001280683	⊕⊕⊕⊕ High	-0.181469751	⊕⊕⊕○ Medium	-0.088452195	⊕⊕⊕⊕ High
182	Empagliflozin_high_dosage:Ertugliflozin_high_dosage	0			-1.08503785	⊕⊕○○ Low	-1.08503785	⊕⊕○○ Low
183	Empagliflozin_high_dosage:Ertugliflozin_low_dosage	0			-1.387268924	⊕⊕○○ Low	-1.387268924	⊕⊕○○ Low
184	Empagliflozin_high_dosage:Exenatide	0			-0.954939336	⊕⊕○○ Low	-0.954939336	⊕⊕○○ Low
185	Empagliflozin_high_dosage:Inject_semaglutide_high_dosage	0			-0.95088306	⊕⊕○○ Low	-0.95088306	⊕⊕○○ Low
186	Empagliflozin_high_dosage:Inject_semaglutide_low_dosage	0			-0.003531252	⊕⊕○○ Low	-0.003531252	⊕⊕○○ Low
187	Empagliflozin_high_dosage:Inject_semaglutide_medium_dosage	0			-0.961145368	⊕⊕○○ Low	-0.961145368	⊕⊕○○ Low
188	Empagliflozin_high_dosage:Liraglutide	0			-1.933325688	⊕⊕○○ Low	-1.933325688	⊕⊕○○ Low
189	Empagliflozin_high_dosage:Lixisenatide	0			-2.744406337	⊕⊕○○ Low	-2.744406337	⊕⊕○○ Low
190	Empagliflozin_high_dosage:Oral_semaglutide	0			-1.06065368	⊕⊕○○ Low	-1.06065368	⊕⊕○○ Low
191	Empagliflozin_high_dosage:Placebo_or_Control	2	-0.47624904	⊕⊕⊕⊕ High	-0.938379859	⊕⊕⊕⊕ High	-0.545531595	⊕⊕⊕⊕ High
192	Empagliflozin_high_dosage:Sotagliflozin	0			1.252233899	⊕⊕○○ Low	1.252233899	⊕⊕○○ Low
193	Empagliflozin_high_dosage:Tirzepatide_high_dosage	0			-0.246542177	⊕⊕○○ Low	-0.246542177	⊕⊕○○ Low
194	Empagliflozin_high_dosage:Tirzepatide_low_dosage	0			0.953250204	⊕⊕○○ Low	0.953250204	⊕⊕○○ Low

195	Empagliflozin_high_dosage:Tirzepatide_medium_dosage	0			-0.032476445	⊕⊕○○ Low	-0.032476445	⊕⊕○○ Low
196	Empagliflozin_low_dosage:Ertugliflozin_high_dosage	0			-0.996585655	⊕⊕○○ Low	-0.996585655	⊕⊕○○ Low
197	Empagliflozin_low_dosage:Ertugliflozin_low_dosage	0			-1.298816728	⊕⊕○○ Low	-1.298816728	⊕⊕○○ Low
198	Empagliflozin_low_dosage:Exenatide	0			-0.866487141	⊕⊕○○ Low	-0.866487141	⊕⊕○○ Low
199	Empagliflozin_low_dosage:Inject_semaglutide_high_dosage	0			-0.862430865	⊕⊕○○ Low	-0.862430865	⊕⊕○○ Low
200	Empagliflozin_low_dosage:Inject_semaglutide_low_dosage	0			0.084920943	⊕⊕○○ Low	0.084920943	⊕⊕○○ Low
201	Empagliflozin_low_dosage:Inject_semaglutide_medium_dosage	0			-0.872693173	⊕⊕○○ Low	-0.872693173	⊕⊕○○ Low
202	Empagliflozin_low_dosage:Liraglutide	0			-1.844873493	⊕⊕○○ Low	-1.844873493	⊕⊕○○ Low
203	Empagliflozin_low_dosage:Lixisenatide	0			-2.655954142	⊕⊕○○ Low	-2.655954142	⊕⊕○○ Low
204	Empagliflozin_low_dosage:Oral_semaglutide	0			-0.972201485	⊕⊕○○ Low	-0.972201485	⊕⊕○○ Low
205	Empagliflozin_low_dosage:Placebo_or_Control	6	-0.444524941	⊕⊕⊕⊕ High	-2.222048597	⊕⊕⊕⊕ High	-0.4570794	⊕⊕⊕⊕ High
206	Empagliflozin_low_dosage:Sotagliflozin	0			1.340686094	⊕⊕○○ Low	1.340686094	⊕⊕○○ Low
207	Empagliflozin_low_dosage:Tirzepatide_high_dosage	0			-0.158089982	⊕⊕○○ Low	-0.158089982	⊕⊕○○ Low
208	Empagliflozin_low_dosage:Tirzepatide_low_dosage	0			1.0417024	⊕⊕○○ Low	1.0417024	⊕⊕○○ Low
209	Empagliflozin_low_dosage:Tirzepatide_medium_dosage	0			0.05597575	⊕⊕○○ Low	0.05597575	⊕⊕○○ Low
210	Ertugliflozin_high_dosage:Ertugliflozin_low_dosage	2	-0.537330523	⊕⊕⊕⊕ High	0.592949082	⊕⊕⊕○ Medium	-0.302231074	⊕⊕⊕⊕ High
211	Ertugliflozin_high_dosage:Exenatide	0			0.130098514	⊕⊕○○ Low	0.130098514	⊕⊕○○ Low
212	Ertugliflozin_high_dosage:Inject_semaglutide_high_dosage	0			0.13415479	⊕⊕○○ Low	0.13415479	⊕⊕○○ Low
213	Ertugliflozin_high_dosage:Inject_semaglutide_low_dosage	0			1.081506598	⊕⊕○○ Low	1.081506598	⊕⊕○○ Low
214	Ertugliflozin_high_dosage:Inject_semaglutide_medium_dosage	0			0.123892482	⊕⊕○○ Low	0.123892482	⊕⊕○○ Low
215	Ertugliflozin_high_dosage:Liraglutide	0			-0.848287838	⊕⊕○○ Low	-0.848287838	⊕⊕○○ Low
216	Ertugliflozin_high_dosage:Lixisenatide	0			-1.659368487	⊕⊕○○ Low	-1.659368487	⊕⊕○○ Low

217	Ertugliflozin_high_dosage:Oral_semaglutide	0			0.02438417	⊕⊕○○ Low	0.02438417	⊕⊕○○ Low
218	Ertugliflozin_high_dosage:Placebo_or_Control	2	0.009673603	⊕⊕⊕⊕ High	2.981390709	⊕⊕⊕⊕ High	0.539506255	⊕⊕⊕⊕ High
219	Ertugliflozin_high_dosage:Sotagliflozin	0			2.337271749	⊕⊕○○ Low	2.337271749	⊕⊕○○ Low
220	Ertugliflozin_high_dosage:Tirzepatide_high_dosage	0			0.838495673	⊕⊕○○ Low	0.838495673	⊕⊕○○ Low
221	Ertugliflozin_high_dosage:Tirzepatide_low_dosage	0			2.038288054	⊕⊕○○ Low	2.038288054	⊕⊕○○ Low
222	Ertugliflozin_high_dosage:Tirzepatide_medium_dosage	0			1.052561405	⊕⊕○○ Low	1.052561405	⊕⊕○○ Low
223	Ertugliflozin_low_dosage:Exenatide	0			0.432329588	⊕⊕○○ Low	0.432329588	⊕⊕○○ Low
224	Ertugliflozin_low_dosage:Inject_semaglutide_high_dosage	0			0.436385864	⊕⊕○○ Low	0.436385864	⊕⊕○○ Low
225	Ertugliflozin_low_dosage:Inject_semaglutide_low_dosage	0			1.383737672	⊕⊕○○ Low	1.383737672	⊕⊕○○ Low
226	Ertugliflozin_low_dosage:Inject_semaglutide_medium_dosage	0			0.426123556	⊕⊕○○ Low	0.426123556	⊕⊕○○ Low
227	Ertugliflozin_low_dosage:Liraglutide	0			-0.546056765	⊕⊕○○ Low	-0.546056765	⊕⊕○○ Low
228	Ertugliflozin_low_dosage:Lixisenatide	0			-1.357137414	⊕⊕○○ Low	-1.357137414	⊕⊕○○ Low
229	Ertugliflozin_low_dosage:Oral_semaglutide	0			0.326615243	⊕⊕○○ Low	0.326615243	⊕⊕○○ Low
230	Ertugliflozin_low_dosage:Placebo_or_Control	1	0.847662292	⊕⊕⊕⊕ High	0.810358217	⊕⊕⊕⊕ High	0.841737329	⊕⊕⊕⊕ High
231	Ertugliflozin_low_dosage:Sotagliflozin	0			2.639502822	⊕⊕○○ Low	2.639502822	⊕⊕○○ Low
232	Ertugliflozin_low_dosage:Tirzepatide_high_dosage	0			1.140726747	⊕⊕○○ Low	1.140726747	⊕⊕○○ Low
233	Ertugliflozin_low_dosage:Tirzepatide_low_dosage	0			2.340519128	⊕⊕○○ Low	2.340519128	⊕⊕○○ Low
234	Ertugliflozin_low_dosage:Tirzepatide_medium_dosage	0			1.354792478	⊕⊕○○ Low	1.354792478	⊕⊕○○ Low
235	Exenatide:Inject_semaglutide_high_dosage	0			0.004056276	⊕⊕○○ Low	0.004056276	⊕⊕○○ Low
236	Exenatide:Inject_semaglutide_low_dosage	0			0.951408084	⊕⊕○○ Low	0.951408084	⊕⊕○○ Low
237	Exenatide:Inject_semaglutide_medium_dosage	0			-0.006206032	⊕⊕○○ Low	-0.006206032	⊕⊕○○ Low
238	Exenatide:Liraglutide	0			-0.978386352	⊕⊕○○ Low	-0.978386352	⊕⊕○○ Low

239	Exenatide:Lixisenatide	0			-1.789467002	⊕⊕○○ Low	-1.789467002	⊕⊕○○ Low
240	Exenatide:Oral_semaglutide	0			-0.105714345	⊕⊕○○ Low	-0.105714345	⊕⊕○○ Low
241	Exenatide:Placebo_or_Control	1	0.409407741	⊕⊕⊕⊕ High			0.409407741	⊕⊕⊕○ Medium
242	Exenatide:Sotagliflozin	0			2.207173234	⊕⊕○○ Low	2.207173234	⊕⊕○○ Low
243	Exenatide:Tirzepatide_high_dosage	0			0.708397159	⊕⊕○○ Low	0.708397159	⊕⊕○○ Low
244	Exenatide:Tirzepatide_low_dosage	0			1.90818954	⊕⊕○○ Low	1.90818954	⊕⊕○○ Low
245	Exenatide:Tirzepatide_medium_dosage	0			0.922462891	⊕⊕○○ Low	0.922462891	⊕⊕○○ Low
246	Inject_semaglutide_high_dosage:Inject_semaglutide_low_dosage	0			0.947351808	⊕⊕○○ Low	0.947351808	⊕⊕○○ Low
247	Inject_semaglutide_high_dosage:Inject_semaglutide_medium_dosage	0			-0.010262308	⊕⊕○○ Low	-0.010262308	⊕⊕○○ Low
248	Inject_semaglutide_high_dosage:Liraglutide	0			-0.982442628	⊕⊕○○ Low	-0.982442628	⊕⊕○○ Low
249	Inject_semaglutide_high_dosage:Lixisenatide	0			-1.793523277	⊕⊕○○ Low	-1.793523277	⊕⊕○○ Low
250	Inject_semaglutide_high_dosage:Oral_semaglutide	0			-0.109770621	⊕⊕○○ Low	-0.109770621	⊕⊕○○ Low
251	Inject_semaglutide_high_dosage:Placebo_or_Control	1	0.405351465	⊕⊕⊕⊕ High			0.405351465	⊕⊕⊕○ Medium
252	Inject_semaglutide_high_dosage:Sotagliflozin	0			2.203116959	⊕⊕○○ Low	2.203116959	⊕⊕○○ Low
253	Inject_semaglutide_high_dosage:Tirzepatide_high_dosage	0			0.704340883	⊕⊕○○ Low	0.704340883	⊕⊕○○ Low
254	Inject_semaglutide_high_dosage:Tirzepatide_low_dosage	0			1.904133264	⊕⊕○○ Low	1.904133264	⊕⊕○○ Low
255	Inject_semaglutide_high_dosage:Tirzepatide_medium_dosage	0			0.918406615	⊕⊕○○ Low	0.918406615	⊕⊕○○ Low
256	Inject_semaglutide_low_dosage:Inject_semaglutide_medium_dosage	1	-1.091679852	⊕⊕⊕⊕ High	-0.75683662	⊕⊕⊕⊕ High	-0.957614116	⊕⊕⊕⊕ High
257	Inject_semaglutide_low_dosage:Liraglutide	0			-1.929794437	⊕⊕○○ Low	-1.929794437	⊕⊕○○ Low
258	Inject_semaglutide_low_dosage:Lixisenatide	0			-2.740875086	⊕⊕○○ Low	-2.740875086	⊕⊕○○ Low
259	Inject_semaglutide_low_dosage:Oral_semaglutide	0			-1.057122429	⊕⊕○○ Low	-1.057122429	⊕⊕○○ Low
260	Inject_semaglutide_low_dosage:Placebo_or_Control	1	-0.408191143	⊕⊕⊕⊕ High	-0.743034375	⊕⊕⊕⊕ High	-0.542000343	⊕⊕⊕⊕ High

261	Inject_semaglutide_low_dosage:Sotagliflozin	0			1.25576515	⊕⊕○○ Low	1.25576515	⊕⊕○○ Low
262	Inject_semaglutide_low_dosage:Tirzepatide_high_dosage	0			-0.243010925	⊕⊕○○ Low	-0.243010925	⊕⊕○○ Low
263	Inject_semaglutide_low_dosage:Tirzepatide_low_dosage	0			0.956781456	⊕⊕○○ Low	0.956781456	⊕⊕○○ Low
264	Inject_semaglutide_low_dosage:Tirzepatide_medium_dosage	0			-0.028945193	⊕⊕○○ Low	-0.028945193	⊕⊕○○ Low
265	Inject_semaglutide_medium_dosage:Liraglutide	0			-0.97218032	⊕⊕○○ Low	-0.97218032	⊕⊕○○ Low
266	Inject_semaglutide_medium_dosage:Lixisenatide	0			-1.783260969	⊕⊕○○ Low	-1.783260969	⊕⊕○○ Low
267	Inject_semaglutide_medium_dosage:Oral_semaglutide	0			-0.099508312	⊕⊕○○ Low	-0.099508312	⊕⊕○○ Low
268	Inject_semaglutide_medium_dosage:Placebo_or_Control	2	0.348645476	⊕⊕⊕⊕ High	0.683488708	⊕⊕⊕⊕ High	0.415613773	⊕⊕⊕⊕ High
269	Inject_semaglutide_medium_dosage:Sotagliflozin	0			2.213379267	⊕⊕○○ Low	2.213379267	⊕⊕○○ Low
270	Inject_semaglutide_medium_dosage:Tirzepatide_high_dosage	0			0.714603191	⊕⊕○○ Low	0.714603191	⊕⊕○○ Low
271	Inject_semaglutide_medium_dosage:Tirzepatide_low_dosage	0			1.914395572	⊕⊕○○ Low	1.914395572	⊕⊕○○ Low
272	Inject_semaglutide_medium_dosage:Tirzepatide_medium_dosage	0			0.928668923	⊕⊕○○ Low	0.928668923	⊕⊕○○ Low
273	Liraglutide:Lixisenatide	0			-0.811080649	⊕⊕○○ Low	-0.811080649	⊕⊕○○ Low
274	Liraglutide:Oral_semaglutide	0			0.872672008	⊕⊕○○ Low	0.872672008	⊕⊕○○ Low
275	Liraglutide:Placebo_or_Control	1	1.387794094	⊕⊕⊕⊕ High			1.387794094	⊕⊕⊕○ Medium
276	Liraglutide:Sotagliflozin	0			3.185559587	⊕⊕○○ Low	3.185559587	⊕⊕○○ Low
277	Liraglutide:Tirzepatide_high_dosage	0			1.686783511	⊕⊕○○ Low	1.686783511	⊕⊕○○ Low
278	Liraglutide:Tirzepatide_low_dosage	0			2.886575893	⊕⊕○○ Low	2.886575893	⊕⊕○○ Low
279	Liraglutide:Tirzepatide_medium_dosage	0			1.900849243	⊕⊕○○ Low	1.900849243	⊕⊕○○ Low
280	Lixisenatide:Oral_semaglutide	0			1.683752657	⊕⊕○○ Low	1.683752657	⊕⊕○○ Low
281	Lixisenatide:Placebo_or_Control	1	2.198874743	⊕⊕⊕⊕ High			2.198874743	⊕⊕⊕○ Medium
282	Lixisenatide:Sotagliflozin	0			3.996640236	⊕⊕○○ Low	3.996640236	⊕⊕○○ Low

283	Lixisenatide:Tirzepatide_high_dosage	0			2.497864161	⊕⊕○○ Low	2.497864161	⊕⊕○○ Low
284	Lixisenatide:Tirzepatide_low_dosage	0			3.697656542	⊕⊕○○ Low	3.697656542	⊕⊕○○ Low
285	Lixisenatide:Tirzepatide_medium_dosage	0			2.711929892	⊕⊕○○ Low	2.711929892	⊕⊕○○ Low
286	Oral_semaglutide:Placebo_or_Control	1	0.515122086	⊕⊕⊕⊕ High			0.515122086	⊕⊕⊕○ Medium
287	Oral_semaglutide:Sotagliflozin	0			2.312887579	⊕⊕○○ Low	2.312887579	⊕⊕○○ Low
288	Oral_semaglutide:Tirzepatide_high_dosage	0			0.814111504	⊕⊕○○ Low	0.814111504	⊕⊕○○ Low
289	Oral_semaglutide:Tirzepatide_low_dosage	0			2.013903885	⊕⊕○○ Low	2.013903885	⊕⊕○○ Low
290	Oral_semaglutide:Tirzepatide_medium_dosage	0			1.028177235	⊕⊕○○ Low	1.028177235	⊕⊕○○ Low
291	Sotagliflozin:Placebo_or_Control	1	-1.797765493	⊕⊕⊕⊕ High			-1.797765493	⊕⊕⊕○ Medium
292	Tirzepatide_high_dosage:Placebo_or_Control	2	-0.013556002	⊕⊕⊕⊕ High	-1.098842812	⊕⊕⊕⊕ High	-0.298989418	⊕⊕⊕⊕ High
293	Tirzepatide_low_dosage:Placebo_or_Control	0			-1.498781799	⊕⊕○○ Low	-1.498781799	⊕⊕○○ Low
294	Tirzepatide_medium_dosage:Placebo_or_Control	1	-1.08509857	⊕⊕⊕⊕ High	0.00018824	⊕⊕⊕○ Medium	-0.51305515	⊕⊕⊕⊕ High
295	Sotagliflozin:Tirzepatide_high_dosage	0			-1.498776075	⊕⊕○○ Low	-1.498776075	⊕⊕○○ Low
296	Sotagliflozin:Tirzepatide_low_dosage	0			-0.298983694	⊕⊕○○ Low	-0.298983694	⊕⊕○○ Low
297	Sotagliflozin:Tirzepatide_medium_dosage	0			-1.284710344	⊕⊕○○ Low	-1.284710344	⊕⊕○○ Low
298	Tirzepatide_high_dosage:Tirzepatide_low_dosage	1	1.07318719	⊕⊕⊕⊕ High	1.373726825	⊕⊕⊕⊕ High	1.199792381	⊕⊕⊕⊕ High
299	Tirzepatide_high_dosage:Tirzepatide_medium_dosage	2	-0.007434402	⊕⊕⊕⊕ High	0.594597416	⊕⊕⊕○ Medium	0.214065732	⊕⊕⊕⊕ High
300	Tirzepatide_low_dosage:Tirzepatide_medium_dosage	1	-1.112171818	⊕⊕⊕⊕ High	-0.811632183	⊕⊕⊕⊕ High	-0.98572665	⊕⊕⊕⊕ High

Table S8C: GRADE of drop-out rate

	Comparison	No.Studies	Direct		Indirect		NMA	
			Estimate	Rate	Estimate	Rate	Estimate	Rate
1	Albiglutide:Bexagliflozin	0			0.505969697	⊕⊕○○ Low	0.505969697	⊕⊕○○ Low
2	Albiglutide:Canagliflozin	0			-0.076095453	⊕⊕○○ Low	-0.076095453	⊕⊕○○ Low
3	Albiglutide:Dapagliflozin	0			-0.077821705	⊕⊕○○ Low	-0.077821705	⊕⊕○○ Low
4	Albiglutide:Dulaglutide	0			-0.407927888	⊕⊕○○ Low	-0.407927888	⊕⊕○○ Low
5	Albiglutide:Efpeglenatide	0			-0.313538318	⊕⊕○○ Low	-0.313538318	⊕⊕○○ Low
6	Albiglutide:Empagliflozin	0			-0.205741398	⊕⊕○○ Low	-0.205741398	⊕⊕○○ Low
7	Albiglutide:Ertugliflozin	0			-0.166820364	⊕⊕○○ Low	-0.166820364	⊕⊕○○ Low
8	Albiglutide:Exenatide	0			-0.191025609	⊕⊕○○ Low	-0.191025609	⊕⊕○○ Low
9	Albiglutide:Inject_semaglutide	0			-0.312015396	⊕⊕○○ Low	-0.312015396	⊕⊕○○ Low
10	Albiglutide:Liraglutide	0			-0.198585572	⊕⊕○○ Low	-0.198585572	⊕⊕○○ Low
11	Albiglutide:Lixisenatide	0			-0.288326343	⊕⊕○○ Low	-0.288326343	⊕⊕○○ Low
12	Albiglutide:Oral_semaglutide	0			-0.999753459	⊕⊕○○ Low	-0.999753459	⊕⊕○○ Low
13	Albiglutide:Placebo_or_Control	1	-0.336554885	⊕⊕⊕⊕ High			-0.336554885	⊕⊕⊕○ Medium
14	Albiglutide:Sotagliflozin	0			-0.017479214	⊕⊕○○ Low	-0.017479214	⊕⊕○○ Low
15	Albiglutide:Tirzepatide	0			-0.728314087	⊕⊕○○ Low	-0.728314087	⊕⊕○○ Low
16	Bexagliflozin:Canagliflozin	0			-0.58206515	⊕⊕○○ Low	-0.58206515	⊕⊕○○ Low
17	Bexagliflozin:Dapagliflozin	0			-0.583791402	⊕⊕○○ Low	-0.583791402	⊕⊕○○ Low
18	Bexagliflozin:Dulaglutide	0			-0.913897585	⊕⊕○○ Low	-0.913897585	⊕⊕○○ Low

19	Bexagliflozin:Efpeglenatide	0			-0.819508015	⊕⊕○○ Low	-0.819508015	⊕⊕○○ Low
20	Bexagliflozin:Empagliflozin	0			-0.711711095	⊕⊕○○ Low	-0.711711095	⊕⊕○○ Low
21	Bexagliflozin:Ertugliflozin	0			-0.672790061	⊕⊕○○ Low	-0.672790061	⊕⊕○○ Low
22	Bexagliflozin:Exenatide	0			-0.696995306	⊕⊕○○ Low	-0.696995306	⊕⊕○○ Low
23	Bexagliflozin:Inject_semaglutide	0			-0.817985093	⊕⊕○○ Low	-0.817985093	⊕⊕○○ Low
24	Bexagliflozin:Liraglutide	0			-0.704555269	⊕⊕○○ Low	-0.704555269	⊕⊕○○ Low
25	Bexagliflozin:Lixisenatide	0			-0.79429604	⊕⊕○○ Low	-0.79429604	⊕⊕○○ Low
26	Bexagliflozin:Oral_semaglutide	0			-1.505723156	⊕⊕○○ Low	-1.505723156	⊕⊕○○ Low
27	Bexagliflozin:Placebo_or_Control	1	-0.842524582	⊕⊕⊕⊕ High			-0.842524582	⊕⊕⊕○ Medium
28	Bexagliflozin:Sotagliflozin	0			-0.523448911	⊕⊕○○ Low	-0.523448911	⊕⊕○○ Low
29	Bexagliflozin:Tirzepatide	0			-1.234283784	⊕⊕○○ Low	-1.234283784	⊕⊕○○ Low
30	Canagliflozin:Dapagliflozin	0			-0.001726252	⊕⊕○○ Low	-0.001726252	⊕⊕○○ Low
31	Canagliflozin:Dulaglutide	0			-0.331832434	⊕⊕○○ Low	-0.331832434	⊕⊕○○ Low
32	Canagliflozin:Efpeglenatide	0			-0.237442865	⊕⊕○○ Low	-0.237442865	⊕⊕○○ Low
33	Canagliflozin:Empagliflozin	0			-0.129645945	⊕⊕○○ Low	-0.129645945	⊕⊕○○ Low
34	Canagliflozin:Ertugliflozin	0			-0.090724911	⊕⊕○○ Low	-0.090724911	⊕⊕○○ Low
35	Canagliflozin:Exenatide	0			-0.114930156	⊕⊕○○ Low	-0.114930156	⊕⊕○○ Low
36	Canagliflozin:Inject_semaglutide	0			-0.235919942	⊕⊕○○ Low	-0.235919942	⊕⊕○○ Low
37	Canagliflozin:Liraglutide	0			-0.122490119	⊕⊕○○ Low	-0.122490119	⊕⊕○○ Low
38	Canagliflozin:Lixisenatide	0			-0.21223089	⊕⊕○○ Low	-0.21223089	⊕⊕○○ Low
39	Canagliflozin:Oral_semaglutide	0			-0.923658005	⊕⊕○○ Low	-0.923658005	⊕⊕○○ Low
40	Canagliflozin:Placebo_or_Control	3	-0.260459432	⊕⊕⊕⊕ High			-0.260459432	⊕⊕⊕○ Medium

41	Canagliflozin:Sotagliflozin	0			0.058616239	⊕⊕○○ Low	0.058616239	⊕⊕○○ Low
42	Canagliflozin:Tirzepatide	0			-0.652218634	⊕⊕○○ Low	-0.652218634	⊕⊕○○ Low
43	Dapagliflozin:Dulaglutide	0			-0.330106183	⊕⊕○○ Low	-0.330106183	⊕⊕○○ Low
44	Dapagliflozin:Efpeglenatide	0			-0.235716614	⊕⊕○○ Low	-0.235716614	⊕⊕○○ Low
45	Dapagliflozin:Empagliflozin	0			-0.127919693	⊕⊕○○ Low	-0.127919693	⊕⊕○○ Low
46	Dapagliflozin:Ertugliflozin	0			-0.088998659	⊕⊕○○ Low	-0.088998659	⊕⊕○○ Low
47	Dapagliflozin:Exenatide	0			-0.113203904	⊕⊕○○ Low	-0.113203904	⊕⊕○○ Low
48	Dapagliflozin:Inject_semaglutide	0			-0.234193691	⊕⊕○○ Low	-0.234193691	⊕⊕○○ Low
49	Dapagliflozin:Liraglutide	0			-0.120763867	⊕⊕○○ Low	-0.120763867	⊕⊕○○ Low
50	Dapagliflozin:Lixisenatide	0			-0.210504638	⊕⊕○○ Low	-0.210504638	⊕⊕○○ Low
51	Dapagliflozin:Oral_semaglutide	0			-0.921931754	⊕⊕○○ Low	-0.921931754	⊕⊕○○ Low
52	Dapagliflozin:Placebo_or_Control	2	-0.25873318	⊕⊕⊕⊕ High			-0.25873318	⊕⊕⊕○ Medium
53	Dapagliflozin:Sotagliflozin	0			0.060342491	⊕⊕○○ Low	0.060342491	⊕⊕○○ Low
54	Dapagliflozin:Tirzepatide	0			-0.650492383	⊕⊕○○ Low	-0.650492383	⊕⊕○○ Low
55	Dulaglutide:Efpeglenatide	0			0.094389569	⊕⊕○○ Low	0.094389569	⊕⊕○○ Low
56	Dulaglutide:Empagliflozin	0			0.20218649	⊕⊕○○ Low	0.20218649	⊕⊕○○ Low
57	Dulaglutide:Ertugliflozin	0			0.241107524	⊕⊕○○ Low	0.241107524	⊕⊕○○ Low
58	Dulaglutide:Exenatide	0			0.216902279	⊕⊕○○ Low	0.216902279	⊕⊕○○ Low
59	Dulaglutide:Inject_semaglutide	0			0.095912492	⊕⊕○○ Low	0.095912492	⊕⊕○○ Low
60	Dulaglutide:Liraglutide	0			0.209342316	⊕⊕○○ Low	0.209342316	⊕⊕○○ Low
61	Dulaglutide:Lixisenatide	0			0.119601544	⊕⊕○○ Low	0.119601544	⊕⊕○○ Low
62	Dulaglutide:Oral_semaglutide	0			-0.591825571	⊕⊕○○ Low	-0.591825571	⊕⊕○○ Low

63	Dulaglutide:Placebo_or_Control	2	0.071373003	⊕⊕⊕⊕ High			0.071373003	⊕⊕⊕○ Medium
64	Dulaglutide:Sotagliflozin	0			0.390448674	⊕⊕○○ Low	0.390448674	⊕⊕○○ Low
65	Dulaglutide:Tirzepatide	0			-0.3203862	⊕⊕○○ Low	-0.3203862	⊕⊕○○ Low
66	Efpeglenatide:Empagliflozin	0			0.107796921	⊕⊕○○ Low	0.107796921	⊕⊕○○ Low
67	Efpeglenatide:Ertugliflozin	0			0.146717955	⊕⊕○○ Low	0.146717955	⊕⊕○○ Low
68	Efpeglenatide:Exenatide	0			0.12251271	⊕⊕○○ Low	0.12251271	⊕⊕○○ Low
69	Efpeglenatide:Inject_semaglutide	0			0.001522923	⊕⊕○○ Low	0.001522923	⊕⊕○○ Low
70	Efpeglenatide:Liraglutide	0			0.114952747	⊕⊕○○ Low	0.114952747	⊕⊕○○ Low
71	Efpeglenatide:Lixisenatide	0			0.025211975	⊕⊕○○ Low	0.025211975	⊕⊕○○ Low
72	Efpeglenatide:Oral_semaglutide	0			-0.68621514	⊕⊕○○ Low	-0.68621514	⊕⊕○○ Low
73	Efpeglenatide:Placebo_or_Control	1	-0.023016566	⊕⊕⊕⊕ High			-0.023016566	⊕⊕⊕○ Medium
74	Efpeglenatide:Sotagliflozin	0			0.296059105	⊕⊕○○ Low	0.296059105	⊕⊕○○ Low
75	Efpeglenatide:Tirzepatide	0			-0.414775769	⊕⊕○○ Low	-0.414775769	⊕⊕○○ Low
76	Empagliflozin:Ertugliflozin	0			0.038921034	⊕⊕○○ Low	0.038921034	⊕⊕○○ Low
77	Empagliflozin:Exenatide	0			0.014715789	⊕⊕○○ Low	0.014715789	⊕⊕○○ Low
78	Empagliflozin:Inject_semaglutide	0			-0.106273998	⊕⊕○○ Low	-0.106273998	⊕⊕○○ Low
79	Empagliflozin:Liraglutide	0			0.007155826	⊕⊕○○ Low	0.007155826	⊕⊕○○ Low
80	Empagliflozin:Lixisenatide	0			-0.082584945	⊕⊕○○ Low	-0.082584945	⊕⊕○○ Low
81	Empagliflozin:Oral_semaglutide	0			-0.794012061	⊕⊕○○ Low	-0.794012061	⊕⊕○○ Low
82	Empagliflozin:Placebo_or_Control	5	-0.130813487	⊕⊕⊕⊕ High			-0.130813487	⊕⊕⊕○ Medium
83	Empagliflozin:Sotagliflozin	0			0.188262184	⊕⊕○○ Low	0.188262184	⊕⊕○○ Low
84	Empagliflozin:Tirzepatide	0			-0.52257269	⊕⊕○○ Low	-0.52257269	⊕⊕○○ Low

85	Ertugliflozin:Exenatide	0			-0.024205245	⊕⊕○○ Low	-0.024205245	⊕⊕○○ Low
86	Ertugliflozin:Inject_semaglutide	0			-0.145195032	⊕⊕○○ Low	-0.145195032	⊕⊕○○ Low
87	Ertugliflozin:Liraglutide	0			-0.031765208	⊕⊕○○ Low	-0.031765208	⊕⊕○○ Low
88	Ertugliflozin:Lixisenatide	0			-0.121505979	⊕⊕○○ Low	-0.121505979	⊕⊕○○ Low
89	Ertugliflozin:Oral_semaglutide	0			-0.832933095	⊕⊕○○ Low	-0.832933095	⊕⊕○○ Low
90	Ertugliflozin:Placebo_or_Control	2	-0.169734521	⊕⊕⊕⊕ High			-0.169734521	⊕⊕⊕○ Medium
91	Ertugliflozin:Sotagliflozin	0			0.14934115	⊕⊕○○ Low	0.14934115	⊕⊕○○ Low
92	Ertugliflozin:Tirzepatide	0			-0.561493723	⊕⊕○○ Low	-0.561493723	⊕⊕○○ Low
93	Exenatide:Inject_semaglutide	0			-0.120989787	⊕⊕○○ Low	-0.120989787	⊕⊕○○ Low
94	Exenatide:Liraglutide	0			-0.007559963	⊕⊕○○ Low	-0.007559963	⊕⊕○○ Low
95	Exenatide:Lixisenatide	0			-0.097300734	⊕⊕○○ Low	-0.097300734	⊕⊕○○ Low
96	Exenatide:Oral_semaglutide	0			-0.80872785	⊕⊕○○ Low	-0.80872785	⊕⊕○○ Low
97	Exenatide:Placebo_or_Control	1	-0.145529276	⊕⊕⊕⊕ High			-0.145529276	⊕⊕⊕○ Medium
98	Exenatide:Sotagliflozin	0			0.173546395	⊕⊕○○ Low	0.173546395	⊕⊕○○ Low
99	Exenatide:Tirzepatide	0			-0.537288478	⊕⊕○○ Low	-0.537288478	⊕⊕○○ Low
100	Inject_semaglutide:Liraglutide	0			0.113429824	⊕⊕○○ Low	0.113429824	⊕⊕○○ Low
101	Inject_semaglutide:Lixisenatide	0			0.023689053	⊕⊕○○ Low	0.023689053	⊕⊕○○ Low
102	Inject_semaglutide:Oral_semaglutide	0			-0.687738063	⊕⊕○○ Low	-0.687738063	⊕⊕○○ Low
103	Inject_semaglutide:Placebo_or_Control	3	-0.024539489	⊕⊕⊕⊕ High			-0.024539489	⊕⊕⊕○ Medium
104	Inject_semaglutide:Sotagliflozin	0			0.294536182	⊕⊕○○ Low	0.294536182	⊕⊕○○ Low
105	Inject_semaglutide:Tirzepatide	0			-0.416298692	⊕⊕○○ Low	-0.416298692	⊕⊕○○ Low
106	Liraglutide:Lixisenatide	0			-0.089740771	⊕⊕○○ Low	-0.089740771	⊕⊕○○ Low

107	Liraglutide:Oral_semaglutide	0			-0.801167887	⊕⊕○○ Low	-0.801167887	⊕⊕○○ Low
108	Liraglutide:Placebo_or_Control	1	-0.137969313	⊕⊕⊕⊕ High			-0.137969313	⊕⊕⊕○ Medium
109	Liraglutide:Sotagliflozin	0			0.181106358	⊕⊕○○ Low	0.181106358	⊕⊕○○ Low
110	Liraglutide:Tirzepatide	0			-0.529728515	⊕⊕○○ Low	-0.529728515	⊕⊕○○ Low
111	Lixisenatide:Oral_semaglutide	0			-0.711427116	⊕⊕○○ Low	-0.711427116	⊕⊕○○ Low
112	Lixisenatide:Placebo_or_Control	1	-0.048228542	⊕⊕⊕⊕ High			-0.048228542	⊕⊕⊕○ Medium
113	Lixisenatide:Sotagliflozin	0			0.270847129	⊕⊕○○ Low	0.270847129	⊕⊕○○ Low
114	Lixisenatide:Tirzepatide	0			-0.439987744	⊕⊕○○ Low	-0.439987744	⊕⊕○○ Low
115	Oral_semaglutide:Placebo_or_Control	1	0.663198574	⊕⊕⊕⊕ High			0.663198574	⊕⊕⊕○ Medium
116	Oral_semaglutide:Sotagliflozin	0			0.982274245	⊕⊕○○ Low	0.982274245	⊕⊕○○ Low
117	Oral_semaglutide:Tirzepatide	0			0.271439371	⊕⊕○○ Low	0.271439371	⊕⊕○○ Low
118	Sotagliflozin:Placebo_or_Control	2	-0.319075671	⊕⊕⊕⊕ High			-0.319075671	⊕⊕⊕○ Medium
119	Tirzepatide:Placebo_or_Control	2	0.391759203	⊕⊕⊕⊕ High			0.391759203	⊕⊕⊕○ Medium
120	Sotagliflozin:Tirzepatide	0			-0.710834873	⊕⊕○○ Low	-0.710834873	⊕⊕○○ Low

Reference list of supplement materials:

1. Page, M.J.; McKenzie, J.E.; Bossuyt, P.M.; Boutron, I.; Hoffmann, T.C.; Mulrow, C.D.; Shamseer, L.; Tetzlaff, J.M.; Akl, E.A.; Brennan, S.E.; et al. The PRISMA 2020 statement: an updated guideline for reporting systematic reviews. *Bmj* **2021**, *372*, n71, doi:10.1136/bmj.n71.
2. Jagomae, T.; Seppa, K.; Reimets, R.; Pastak, M.; Plaas, M.; Hickey, M.A.; Kukker, K.G.; Moons, L.; De Groef, L.; Vasar, E.; et al. Early Intervention and Lifelong Treatment with GLP1 Receptor Agonist Liraglutide in a Wolfram Syndrome Rat Model with an Emphasis on Visual Neurodegeneration, Sensorineural Hearing Loss and Diabetic Phenotype. *Cells* **2021**, *10*, doi:10.3390/cells10113193.
3. Scully, K.J.; Wolfsdorf, J.I. Efficacy of GLP-1 Agonist Therapy in Autosomal Dominant WFS1-Related Disorder: A Case Report. *Horm Res Paediatr* **2020**, *93*, 409-414, doi:10.1159/000510852.
4. Koychev, I.; Reid, G.; Nguyen, M.; Mentz, R.J.; Joyce, D.; Shah, S.H.; Holman, R.R. Inflammatory proteins associated with Alzheimer's disease reduced by a GLP1 receptor agonist: a post hoc analysis of the EXSCEL randomized placebo controlled trial. *Alzheimers Res Ther* **2024**, *16*, 212, doi:10.1186/s13195-024-01573-x.
5. Vardeny, O.; Desai, A.S.; Jhund, P.S.; Fang, J.C.; Claggett, B.; de Boer, R.A.; Hernandez, A.F.; Inzucchi, S.E.; Kosiborod, M.N.; Lam, C.S.P.; et al. Dapagliflozin and Mode of Death in Heart Failure With Improved Ejection Fraction: A Post Hoc Analysis of the DELIVER Trial. *JAMA cardiology* **2024**, *9*, 283-289, doi:10.1001/jamacardio.2023.5318.
6. Gerstein, H.C.; Lee, S.F.; Pare, G.; Bethel, M.A.; Colhoun, H.M.; Hoover, A.; Lakshmanan, M.; Lin, Y.; Pirro, V.; Qian, H.R.; et al. Biomarker Changes Associated With Both Dulaglutide and Cardiovascular Events in the REWIND Randomized Controlled Trial: A Nested Case-Control Post Hoc Analysis. *Diabetes Care* **2023**, *46*, 1046-1051, doi:10.2337/dc22-2397.
7. Schechter, M.; Wiviott, S.D.; Raz, I.; Goodrich, E.L.; Rozenberg, A.; Yanuv, I.; Murphy, S.A.; Zelniker, T.A.; Fredriksson, M.; Johansson, P.A.; et al. Effects of dapagliflozin on hospitalisations in people with type 2 diabetes: post-hoc analyses of the DECLARE-TIMI 58 trial. *Lancet Diabetes Endocrinol* **2023**, *11*, 233-241, doi:10.1016/S2213-8587(23)00009-8.

8. Heller, S.R.; Geybels, M.S.; Iqbal, A.; Liu, L.; Wagner, L.; Chow, E. A higher non-severe hypoglycaemia rate is associated with an increased risk of subsequent severe hypoglycaemia and major adverse cardiovascular events in individuals with type 2 diabetes in the LEADER study. *Diabetologia* **2022**, *65*, 55-64, doi:10.1007/s00125-021-05556-7.
9. Leiter, L.A.; Cefalu, W.T.; de Bruin, T.W.; Xu, J.; Parikh, S.; Johnsson, E.; Gause-Nilsson, I. Long-term maintenance of efficacy of dapagliflozin in patients with type 2 diabetes mellitus and cardiovascular disease. *Diabetes Obes Metab* **2016**, *18*, 766-774, doi:10.1111/dom.12666.
10. Rosenstock, J.; Raccah, D.; Koranyi, L.; Maffei, L.; Boka, G.; Miossec, P.; Gerich, J.E. Efficacy and safety of lixisenatide once daily versus exenatide twice daily in type 2 diabetes inadequately controlled on metformin: a 24-week, randomized, open-label, active-controlled study (GetGoal-X). *Diabetes Care* **2013**, *36*, 2945-2951, doi:10.2337/dc12-2709.
11. Charbonnel, B.; Steinberg, H.; Eymard, E.; Xu, L.; Thakkar, P.; Prabhu, V.; Davies, M.J.; Engel, S.S. Efficacy and safety over 26 weeks of an oral treatment strategy including sitagliptin compared with an injectable treatment strategy with liraglutide in patients with type 2 diabetes mellitus inadequately controlled on metformin: a randomised clinical trial. *Diabetologia* **2013**, *56*, 1503-1511, doi:10.1007/s00125-013-2905-1.
12. Schernthaner, G.; Gross, J.L.; Rosenstock, J.; Guarisco, M.; Fu, M.; Yee, J.; Kawaguchi, M.; Canovatchel, W.; Meininger, G. Canagliflozin compared with sitagliptin for patients with type 2 diabetes who do not have adequate glycemic control with metformin plus sulfonylurea: a 52-week randomized trial. *Diabetes Care* **2013**, *36*, 2508-2515, doi:10.2337/dc12-2491.
13. Gallwitz, B.; Guzman, J.; Dotta, F.; Guerci, B.; Simo, R.; Basson, B.R.; Festa, A.; Kiljanski, J.; Sapin, H.; Trautmann, M.; et al. Exenatide twice daily versus glimepiride for prevention of glycaemic deterioration in patients with type 2 diabetes with metformin failure (EUREXA): an open-label, randomised controlled trial. *Lancet* **2012**, *379*, 2270-2278, doi:10.1016/S0140-6736(12)60479-6.
14. Wilding, J.P.; Woo, V.; Soler, N.G.; Pahor, A.; Sugg, J.; Rohwedder, K.; Parikh, S.; Dapagliflozin 006 Study, G. Long-term efficacy of dapagliflozin in patients with type 2 diabetes mellitus receiving high doses of insulin: a randomized trial. *Ann Intern Med* **2012**, *156*, 405-415, doi:10.7326/0003-4819-156-6-201203200-00003.

15. Gallwitz, B.; Bohmer, M.; Segiet, T.; Molle, A.; Milek, K.; Becker, B.; Helsberg, K.; Petto, H.; Peters, N.; Bachmann, O. Exenatide twice daily versus premixed insulin aspart 70/30 in metformin-treated patients with type 2 diabetes: a randomized 26-week study on glycemic control and hypoglycemia. *Diabetes Care* **2011**, *34*, 604-606, doi:10.2337/dc10-1900.
16. Bailey, C.J.; Gross, J.L.; Pieters, A.; Bastien, A.; List, J.F. Effect of dapagliflozin in patients with type 2 diabetes who have inadequate glycaemic control with metformin: a randomised, double-blind, placebo-controlled trial. *Lancet* **2010**, *375*, 2223-2233, doi:10.1016/S0140-6736(10)60407-2.
17. Buse, J.B.; Rosenstock, J.; Sesti, G.; Schmidt, W.E.; Montanya, E.; Brett, J.H.; Zychma, M.; Blonde, L.; Group, L.-S. Liraglutide once a day versus exenatide twice a day for type 2 diabetes: a 26-week randomised, parallel-group, multinational, open-label trial (LEAD-6). *Lancet* **2009**, *374*, 39-47, doi:10.1016/S0140-6736(09)60659-0.
18. Nauck, M.; Frid, A.; Hermansen, K.; Shah, N.S.; Tankova, T.; Mitha, I.H.; Zdravkovic, M.; During, M.; Matthews, D.R.; Group, L.-S. Efficacy and safety comparison of liraglutide, glimepiride, and placebo, all in combination with metformin, in type 2 diabetes: the LEAD (liraglutide effect and action in diabetes)-2 study. *Diabetes Care* **2009**, *32*, 84-90, doi:10.2337/dc08-1355.
19. Garber, A.; Henry, R.; Ratner, R.; Garcia-Hernandez, P.A.; Rodriguez-Pattzi, H.; Olvera-Alvarez, I.; Hale, P.M.; Zdravkovic, M.; Bode, B.; Group, L.-S. Liraglutide versus glimepiride monotherapy for type 2 diabetes (LEAD-3 Mono): a randomised, 52-week, phase III, double-blind, parallel-treatment trial. *Lancet* **2009**, *373*, 473-481, doi:10.1016/S0140-6736(08)61246-5.
20. Rosenstock, J.; Fonseca, V.A.; Gross, J.L.; Ratner, R.E.; Ahren, B.; Chow, F.C.; Yang, F.; Miller, D.; Johnson, S.L.; Stewart, M.W.; et al. Advancing basal insulin replacement in type 2 diabetes inadequately controlled with insulin glargine plus oral agents: a comparison of adding albiglutide, a weekly GLP-1 receptor agonist, versus thrice-daily prandial insulin lispro. *Diabetes Care* **2014**, *37*, 2317-2325, doi:10.2337/dc14-0001.
21. Wysham, C.; Blevins, T.; Arakaki, R.; Colon, G.; Garcia, P.; Atisso, C.; Kuhstoss, D.; Lakshmanan, M. Efficacy and safety of dulaglutide added onto pioglitazone and metformin versus exenatide in type 2 diabetes in a randomized controlled trial (AWARD-1). *Diabetes Care* **2014**, *37*, 2159-2167, doi:10.2337/dc13-2760.

22. Umpierrez, G.; Tofe Povedano, S.; Perez Manghi, F.; Shurzinske, L.; Pechtner, V. Efficacy and safety of dulaglutide monotherapy versus metformin in type 2 diabetes in a randomized controlled trial (AWARD-3). *Diabetes Care* **2014**, *37*, 2168-2176, doi:10.2337/dc13-2759.
23. Barnett, A.H.; Mithal, A.; Manassie, J.; Jones, R.; Rattunde, H.; Woerle, H.J.; Broedl, U.C.; investigators, E.-R.R.t. Efficacy and safety of empagliflozin added to existing antidiabetes treatment in patients with type 2 diabetes and chronic kidney disease: a randomised, double-blind, placebo-controlled trial. *Lancet Diabetes Endocrinol* **2014**, *2*, 369-384, doi:10.1016/S2213-8587(13)70208-0.
24. Pratley, R.E.; Nauck, M.A.; Barnett, A.H.; Feinglos, M.N.; Ovalle, F.; Harman-Boehm, I.; Ye, J.; Scott, R.; Johnson, S.; Stewart, M.; et al. Once-weekly albiglutide versus once-daily liraglutide in patients with type 2 diabetes inadequately controlled on oral drugs (HARMONY 7): a randomised, open-label, multicentre, non-inferiority phase 3 study. *Lancet Diabetes Endocrinol* **2014**, *2*, 289-297, doi:10.1016/S2213-8587(13)70214-6.
25. Roden, M.; Weng, J.; Eilbracht, J.; Delafont, B.; Kim, G.; Woerle, H.J.; Broedl, U.C.; investigators, E.-R.M.t. Empagliflozin monotherapy with sitagliptin as an active comparator in patients with type 2 diabetes: a randomised, double-blind, placebo-controlled, phase 3 trial. *Lancet Diabetes Endocrinol* **2013**, *1*, 208-219, doi:10.1016/S2213-8587(13)70084-6.
26. Polidori, D.; Mari, A.; Ferrannini, E. Canagliflozin, a sodium glucose co-transporter 2 inhibitor, improves model-based indices of beta cell function in patients with type 2 diabetes. *Diabetologia* **2014**, *57*, 891-901, doi:10.1007/s00125-014-3196-x.
27. Ferrannini, E.; Berk, A.; Hantel, S.; Pinnetti, S.; Hach, T.; Woerle, H.J.; Broedl, U.C. Long-term safety and efficacy of empagliflozin, sitagliptin, and metformin: an active-controlled, parallel-group, randomized, 78-week open-label extension study in patients with type 2 diabetes. *Diabetes Care* **2013**, *36*, 4015-4021, doi:10.2337/dc13-0663.
28. Stenlof, K.; Cefalu, W.T.; Kim, K.A.; Jodar, E.; Alba, M.; Edwards, R.; Tong, C.; Canovatchel, W.; Meininger, G. Long-term efficacy and safety of canagliflozin monotherapy in patients with type 2 diabetes inadequately controlled with diet and exercise: findings from the 52-week CANTATA-M study. *Curr Med Res Opin* **2014**, *30*, 163-175, doi:10.1185/03007995.2013.850066.

29. Lavalle-Gonzalez, F.J.; Januszewicz, A.; Davidson, J.; Tong, C.; Qiu, R.; Canovatchel, W.; Meininger, G. Efficacy and safety of canagliflozin compared with placebo and sitagliptin in patients with type 2 diabetes on background metformin monotherapy: a randomised trial. *Diabetologia* **2013**, *56*, 2582-2592, doi:10.1007/s00125-013-3039-1.
30. Mathieu, C.; Ranetti, A.E.; Li, D.; Ekholm, E.; Cook, W.; Hirshberg, B.; Chen, H.; Hansen, L.; Iqbal, N. Randomized, Double-Blind, Phase 3 Trial of Triple Therapy With Dapagliflozin Add-on to Saxagliptin Plus Metformin in Type 2 Diabetes. *Diabetes Care* **2015**, *38*, 2009-2017, doi:10.2337/dc15-0779.
31. Kovacs, C.S.; Seshiah, V.; Merker, L.; Christiansen, A.V.; Roux, F.; Salsali, A.; Kim, G.; Stella, P.; Woerle, H.J.; Broedl, U.C.; et al. Empagliflozin as Add-on Therapy to Pioglitazone With or Without Metformin in Patients With Type 2 Diabetes Mellitus. *Clin Ther* **2015**, *37*, 1773-1788 e1771, doi:10.1016/j.clinthera.2015.05.511.
32. Pi-Sunyer, X.; Astrup, A.; Fujioka, K.; Greenway, F.; Halpern, A.; Krempf, M.; Lau, D.C.; le Roux, C.W.; Violante Ortiz, R.; Jensen, C.B.; et al. A Randomized, Controlled Trial of 3.0 mg of Liraglutide in Weight Management. *N Engl J Med* **2015**, *373*, 11-22, doi:10.1056/NEJMoa1411892.
33. Giorgino, F.; Benroubi, M.; Sun, J.H.; Zimmermann, A.G.; Pechtner, V. Efficacy and Safety of Once-Weekly Dulaglutide Versus Insulin Glargine in Patients With Type 2 Diabetes on Metformin and Glimepiride (AWARD-2). *Diabetes Care* **2015**, *38*, 2241-2249, doi:10.2337/dc14-1625.
34. Blonde, L.; Jendle, J.; Gross, J.; Woo, V.; Jiang, H.; Fahrbach, J.L.; Milicevic, Z. Once-weekly dulaglutide versus bedtime insulin glargine, both in combination with prandial insulin lispro, in patients with type 2 diabetes (AWARD-4): a randomised, open-label, phase 3, non-inferiority study. *Lancet* **2015**, *385*, 2057-2066, doi:10.1016/S0140-6736(15)60936-9.
35. Weinstock, R.S.; Guerci, B.; Umpierrez, G.; Nauck, M.A.; Skrivanek, Z.; Milicevic, Z. Safety and efficacy of once-weekly dulaglutide versus sitagliptin after 2 years in metformin-treated patients with type 2 diabetes (AWARD-5): a randomized, phase III study. *Diabetes Obes Metab* **2015**, *17*, 849-858, doi:10.1111/dom.12479.
36. Cefalu, W.T.; Leiter, L.A.; de Bruin, T.W.; Gause-Nilsson, I.; Sugg, J.; Parikh, S.J. Dapagliflozin's Effects on Glycemia and Cardiovascular Risk Factors in High-Risk Patients With Type 2 Diabetes: A 24-Week, Multicenter, Randomized, Double-

Blind, Placebo-Controlled Study With a 28-Week Extension. *Diabetes Care* **2015**, *38*, 1218-1227, doi:10.2337/dc14-0315.
37. Weissman, P.N.; Carr, M.C.; Ye, J.; Cirkel, D.T.; Stewart, M.; Perry, C.; Pratley, R. HARMONY 4: randomised clinical trial comparing once-weekly albiglutide and insulin glargine in patients with type 2 diabetes inadequately controlled with metformin with or without sulfonylurea. *Diabetologia* **2014**, *57*, 2475-2484, doi:10.1007/s00125-014-3360-3.
38. Dungan, K.M.; Povedano, S.T.; Forst, T.; Gonzalez, J.G.; Atisso, C.; Sealls, W.; Fahrbach, J.L. Once-weekly dulaglutide versus once-daily liraglutide in metformin-treated patients with type 2 diabetes (AWARD-6): a randomised, open-label, phase 3, non-inferiority trial. *Lancet* **2014**, *384*, 1349-1357, doi:10.1016/S0140-6736(14)60976-4.
39. Ridderstrale, M.; Andersen, K.R.; Zeller, C.; Kim, G.; Woerle, H.J.; Broedl, U.C.; investigators, E.-R.H.H.S.t. Comparison of empagliflozin and glimepiride as add-on to metformin in patients with type 2 diabetes: a 104-week randomised, active-controlled, double-blind, phase 3 trial. *Lancet Diabetes Endocrinol* **2014**, *2*, 691-700, doi:10.1016/S2213-8587(14)70120-2.
40. Meneilly, G.S.; Roy-Duval, C.; Alawi, H.; Dailey, G.; Bellido, D.; Trescoli, C.; Manrique Hurtado, H.; Guo, H.; Pilorget, V.; Perfetti, R.; et al. Lixisenatide Therapy in Older Patients With Type 2 Diabetes Inadequately Controlled on Their Current Antidiabetic Treatment: The GetGoal-O Randomized Trial. *Diabetes Care* **2017**, *40*, 485-493, doi:10.2337/dc16-2143.
41. Mellander, A.; Billger, M.; Johnsson, E.; Traff, A.K.; Yoshida, S.; Johnsson, K. Hypersensitivity Events, Including Potentially Hypersensitivity-Related Skin Events, with Dapagliflozin in Patients with Type 2 Diabetes Mellitus: A Pooled Analysis. *Clinical drug investigation* **2016**, *36*, 925-933, doi:10.1007/s40261-016-0438-3.
42. Hadjadj, S.; Rosenstock, J.; Meinicke, T.; Woerle, H.J.; Broedl, U.C. Initial Combination of Empagliflozin and Metformin in Patients With Type 2 Diabetes. *Diabetes Care* **2016**, *39*, 1718-1728, doi:10.2337/dc16-0522.
43. Nauck, M.; Rizzo, M.; Johnson, A.; Bosch-Traberg, H.; Madsen, J.; Cariou, B. Once-Daily Liraglutide Versus Lixisenatide as Add-on to Metformin in Type 2 Diabetes: A 26-Week Randomized Controlled Clinical Trial. *Diabetes Care* **2016**, *39*, 1501-1509, doi:10.2337/dc15-2479.
44. Investigators, F.-S.T. Glucose Variability in a 26-Week Randomized Comparison of Mealtime Treatment With Rapid-Acting Insulin Versus GLP-1 Agonist in Participants With Type 2 Diabetes at High Cardiovascular Risk. *Diabetes Care* **2016**, *39*, 973-

981, doi:10.2337/dc15-2782.
45. Dungan, K.M.; Weitgasser, R.; Perez Manghi, F.; Pintilei, E.; Fahrbach, J.L.; Jiang, H.H.; Shell, J.; Robertson, K.E. A 24-week study to evaluate the efficacy and safety of once-weekly dulaglutide added on to glimepiride in type 2 diabetes (AWARD-8). *Diabetes Obes Metab* **2016**, *18*, 475-482, doi:10.1111/dom.12634.
46. Davies, M.J.; Bain, S.C.; Atkin, S.L.; Rossing, P.; Scott, D.; Shamkhalova, M.S.; Bosch-Traberg, H.; Syren, A.; Umpierrez, G.E. Efficacy and Safety of Liraglutide Versus Placebo as Add-on to Glucose-Lowering Therapy in Patients With Type 2 Diabetes and Moderate Renal Impairment (LIRA-RENAL): A Randomized Clinical Trial. *Diabetes Care* **2016**, *39*, 222-230, doi:10.2337/dc14-2883.
47. Nauck, M.A.; Stewart, M.W.; Perkins, C.; Jones-Leone, A.; Yang, F.; Perry, C.; Reinhardt, R.R.; Rendell, M. Efficacy and safety of once-weekly GLP-1 receptor agonist albiglutide (HARMONY 2): 52 week primary endpoint results from a randomised, placebo-controlled trial in patients with type 2 diabetes mellitus inadequately controlled with diet and exercise. *Diabetologia* **2016**, *59*, 266-274, doi:10.1007/s00125-015-3795-1.
48. Davies, M.J.; Bergenstal, R.; Bode, B.; Kushner, R.F.; Lewin, A.; Skjoth, T.V.; Andreasen, A.H.; Jensen, C.B.; DeFronzo, R.A.; Group, N.N.S. Efficacy of Liraglutide for Weight Loss Among Patients With Type 2 Diabetes: The SCALE Diabetes Randomized Clinical Trial. *Jama* **2015**, *314*, 687-699, doi:10.1001/jama.2015.9676.
49. Aronson, R.; Frias, J.; Goldman, A.; Darekar, A.; Lauring, B.; Terra, S.G. Long-term efficacy and safety of ertugliflozin monotherapy in patients with inadequately controlled T2DM despite diet and exercise: VERTIS MONO extension study. *Diabetes Obes Metab* **2018**, *20*, 1453-1460, doi:10.1111/dom.13251.
50. Pratley, R.E.; Aroda, V.R.; Lingvay, I.; Ludemann, J.; Andreassen, C.; Navarria, A.; Viljoen, A.; investigators, S. Semaglutide versus dulaglutide once weekly in patients with type 2 diabetes (SUSTAIN 7): a randomised, open-label, phase 3b trial. *Lancet Diabetes Endocrinol* **2018**, *6*, 275-286, doi:10.1016/S2213-8587(18)30024-X.
51. Kaku, K.; Yamada, Y.; Watada, H.; Abiko, A.; Nishida, T.; Zacho, J.; Kiyosue, A. Safety and efficacy of once-weekly semaglutide vs additional oral antidiabetic drugs in Japanese people with inadequately controlled type 2 diabetes: A

randomized trial. *Diabetes Obes Metab* **2018**, *20*, 1202-1212, doi:10.1111/dom.13218.
52. Ahmann, A.J.; Capehorn, M.; Charpentier, G.; Dotta, F.; Henkel, E.; Lingvay, I.; Holst, A.G.; Annett, M.P.; Aroda, V.R. Efficacy and Safety of Once-Weekly Semaglutide Versus Exenatide ER in Subjects With Type 2 Diabetes (SUSTAIN 3): A 56-Week, Open-Label, Randomized Clinical Trial. *Diabetes Care* **2018**, *41*, 258-266, doi:10.2337/dc17-0417.
53. Grunberger, G.; Camp, S.; Johnson, J.; Huyck, S.; Terra, S.G.; Mancuso, J.P.; Jiang, Z.W.; Golm, G.; Engel, S.S.; Lauring, B. Ertugliflozin in Patients with Stage 3 Chronic Kidney Disease and Type 2 Diabetes Mellitus: The VERTIS RENAL Randomized Study. *Diabetes Ther* **2018**, *9*, 49-66, doi:10.1007/s13300-017-0337-5.
54. Yu, M.; Brunt, K.V.; Milicevic, Z.; Varnado, O.; Boye, K.S. Patient-reported Outcomes in Patients with Type 2 Diabetes Treated with Dulaglutide Added to Titrated Insulin Glargine (AWARD-9). *Clin Ther* **2017**, *39*, 2284-2295, doi:10.1016/j.clinthera.2017.10.002.
55. Home, P.D.; Ahren, B.; Reusch, J.E.B.; Rendell, M.; Weissman, P.N.; Cirkel, D.T.; Miller, D.; Ambery, P.; Carr, M.C.; Nauck, M.A. Three-year data from 5 HARMONY phase 3 clinical trials of albiglutide in type 2 diabetes mellitus: Long-term efficacy with or without rescue therapy. *Diabetes Res Clin Pract* **2017**, *131*, 49-60, doi:10.1016/j.diabres.2017.06.013.
56. Januzzi, J.L., Jr.; Butler, J.; Jarolim, P.; Sattar, N.; Vijapurkar, U.; Desai, M.; Davies, M.J. Effects of Canagliflozin on Cardiovascular Biomarkers in Older Adults With Type 2 Diabetes. *J Am Coll Cardiol* **2017**, *70*, 704-712, doi:10.1016/j.jacc.2017.06.016.
57. Ahren, B.; Masmiquel, L.; Kumar, H.; Sargin, M.; Karsbol, J.D.; Jacobsen, S.H.; Chow, F. Efficacy and safety of once-weekly semaglutide versus once-daily sitagliptin as an add-on to metformin, thiazolidinediones, or both, in patients with type 2 diabetes (SUSTAIN 2): a 56-week, double-blind, phase 3a, randomised trial. *Lancet Diabetes Endocrinol* **2017**, *5*, 341-354, doi:10.1016/S2213-8587(17)30092-X.
58. Aroda, V.R.; Bain, S.C.; Cariou, B.; Piletic, M.; Rose, L.; Axelsen, M.; Rowe, E.; DeVries, J.H. Efficacy and safety of once-weekly semaglutide versus once-daily insulin glargine as add-on to metformin (with or without sulfonylureas) in insulin-naive patients with type 2 diabetes (SUSTAIN 4): a randomised, open-label, parallel-group, multicentre, multinational, phase

3a trial. *Lancet Diabetes Endocrinol* **2017**, *5*, 355-366, doi:10.1016/S2213-8587(17)30085-2.
59. Pratley, R.; Amod, A.; Hoff, S.T.; Kadowaki, T.; Lingvay, I.; Nauck, M.; Pedersen, K.B.; Saugstrup, T.; Meier, J.J.; investigators, P. Oral semaglutide versus subcutaneous liraglutide and placebo in type 2 diabetes (PIONEER 4): a randomised, double-blind, phase 3a trial. *Lancet* **2019**, *394*, 39-50, doi:10.1016/S0140-6736(19)31271-1.
60. Husain, M.; Birkenfeld, A.L.; Donsmark, M.; Dungan, K.; Eliaschewitz, F.G.; Franco, D.R.; Jeppesen, O.K.; Lingvay, I.; Mosenzon, O.; Pedersen, S.D.; et al. Oral Semaglutide and Cardiovascular Outcomes in Patients with Type 2 Diabetes. *N Engl J Med* **2019**, *381*, 841-851, doi:10.1056/NEJMoa1901118.
61. Perkovic, V.; Jardine, M.J.; Neal, B.; Bompoint, S.; Heerspink, H.J.L.; Charytan, D.M.; Edwards, R.; Agarwal, R.; Bakris, G.; Bull, S.; et al. Canagliflozin and Renal Outcomes in Type 2 Diabetes and Nephropathy. *N Engl J Med* **2019**, *380*, 2295-2306, doi:10.1056/NEJMoa1811744.
62. Coskun, T.; Sloop, K.W.; Loghin, C.; Alsina-Fernandez, J.; Urva, S.; Bokvist, K.B.; Cui, X.; Briere, D.A.; Cabrera, O.; Roell, W.C.; et al. LY3298176, a novel dual GIP and GLP-1 receptor agonist for the treatment of type 2 diabetes mellitus: From discovery to clinical proof of concept. *Mol Metab* **2018**, *18*, 3-14, doi:10.1016/j.molmet.2018.09.009.
63. Frias, J.P.; Nauck, M.A.; Van, J.; Kutner, M.E.; Cui, X.; Benson, C.; Urva, S.; Gimeno, R.E.; Milicevic, Z.; Robins, D.; et al. Efficacy and safety of LY3298176, a novel dual GIP and GLP-1 receptor agonist, in patients with type 2 diabetes: a randomised, placebo-controlled and active comparator-controlled phase 2 trial. *Lancet* **2018**, *392*, 2180-2193, doi:10.1016/S0140-6736(18)32260-8.
64. O'Neil, P.M.; Birkenfeld, A.L.; McGowan, B.; Mosenzon, O.; Pedersen, S.D.; Wharton, S.; Carson, C.G.; Jepsen, C.H.; Kabisch, M.; Wilding, J.P.H. Efficacy and safety of semaglutide compared with liraglutide and placebo for weight loss in patients with obesity: a randomised, double-blind, placebo and active controlled, dose-ranging, phase 2 trial. *Lancet* **2018**, *392*, 637-649, doi:10.1016/S0140-6736(18)31773-2.
65. Danne, T.; Cariou, B.; Banks, P.; Brandle, M.; Brath, H.; Franek, E.; Kushner, J.A.; Lapuerta, P.; McGuire, D.K.; Peters, A.L.; et al. HbA(1c) and Hypoglycemia Reductions at 24 and 52 Weeks With Sotagliflozin in Combination With Insulin in Adults

With Type 1 Diabetes: The European inTandem2 Study. *Diabetes Care* **2018**, *41*, 1981-1990, doi:10.2337/dc18-0342.
66. Buse, J.B.; Garg, S.K.; Rosenstock, J.; Bailey, T.S.; Banks, P.; Bode, B.W.; Danne, T.; Kushner, J.A.; Lane, W.S.; Lapuerta, P.; et al. Sotagliflozin in Combination With Optimized Insulin Therapy in Adults With Type 1 Diabetes: The North American inTandem1 Study. *Diabetes Care* **2018**, *41*, 1970-1980, doi:10.2337/dc18-0343.
67. Tuttle, K.R.; Lakshmanan, M.C.; Rayner, B.; Busch, R.S.; Zimmermann, A.G.; Woodward, D.B.; Botros, F.T. Dulaglutide versus insulin glargine in patients with type 2 diabetes and moderate-to-severe chronic kidney disease (AWARD-7): a multicentre, open-label, randomised trial. *Lancet Diabetes Endocrinol* **2018**, *6*, 605-617, doi:10.1016/S2213-8587(18)30104-9.
68. Ludvik, B.; Frias, J.P.; Tinahones, F.J.; Wainstein, J.; Jiang, H.; Robertson, K.E.; Garcia-Perez, L.E.; Woodward, D.B.; Milicevic, Z. Dulaglutide as add-on therapy to SGLT2 inhibitors in patients with inadequately controlled type 2 diabetes (AWARD-10): a 24-week, randomised, double-blind, placebo-controlled trial. *Lancet Diabetes Endocrinol* **2018**, *6*, 370-381, doi:10.1016/S2213-8587(18)30023-8.
69. Bhatt, D.L.; Szarek, M.; Pitt, B.; Cannon, C.P.; Leiter, L.A.; McGuire, D.K.; Lewis, J.B.; Riddle, M.C.; Inzucchi, S.E.; Kosiborod, M.N.; et al. Sotagliflozin in Patients with Diabetes and Chronic Kidney Disease. *N Engl J Med* **2021**, *384*, 129-139, doi:10.1056/NEJMoa2030186.
70. Stack, A.G.; Han, D.; Goldwater, R.; Johansson, S.; Dronamraju, N.; Oscarsson, J.; Johnsson, E.; Parkinson, J.; Erlandsson, F. Dapagliflozin Added to Verinurad Plus Febuxostat Further Reduces Serum Uric Acid in Hyperuricemia: The QUARTZ Study. *J Clin Endocrinol Metab* **2021**, *106*, e2347-e2356, doi:10.1210/clinem/dgaa748.
71. Heerspink, H.J.L.; Stefansson, B.V.; Correa-Rotter, R.; Chertow, G.M.; Greene, T.; Hou, F.F.; Mann, J.F.E.; McMurray, J.J.V.; Lindberg, M.; Rossing, P.; et al. Dapagliflozin in Patients with Chronic Kidney Disease. *N Engl J Med* **2020**, *383*, 1436-1446, doi:10.1056/NEJMoa2024816.
72. Lingvay, I.; Catarig, A.M.; Frias, J.P.; Kumar, H.; Lausvig, N.L.; le Roux, C.W.; Thielke, D.; Viljoen, A.; McCrimmon, R.J. Efficacy and safety of once-weekly semaglutide versus daily canagliflozin as add-on to metformin in patients with type 2 diabetes (SUSTAIN 8): a double-blind, phase 3b, randomised controlled trial. *Lancet Diabetes Endocrinol* **2019**, *7*, 834-844,

doi:10.1016/S2213-8587(19)30311-0.
73. McMurray, J.J.V.; Solomon, S.D.; Inzucchi, S.E.; Kober, L.; Kosiborod, M.N.; Martinez, F.A.; Ponikowski, P.; Sabatine, M.S.; Anand, I.S.; Belohlavek, J.; et al. Dapagliflozin in Patients with Heart Failure and Reduced Ejection Fraction. *N Engl J Med* **2019**, *381*, 1995-2008, doi:10.1056/NEJMoa1911303.
74. Rodbard, H.W.; Rosenstock, J.; Canani, L.H.; Deerochanawong, C.; Gumprecht, J.; Lindberg, S.O.; Lingvay, I.; Sondergaard, A.L.; Treppendahl, M.B.; Montanya, E.; et al. Oral Semaglutide Versus Empagliflozin in Patients With Type 2 Diabetes Uncontrolled on Metformin: The PIONEER 2 Trial. *Diabetes Care* **2019**, *42*, 2272-2281, doi:10.2337/dc19-0883.
75. Mullins, R.J.; Mustapic, M.; Chia, C.W.; Carlson, O.; Gulyani, S.; Tran, J.; Li, Y.; Mattson, M.P.; Resnick, S.; Egan, J.M.; et al. A Pilot Study of Exenatide Actions in Alzheimer's Disease. *Curr Alzheimer Res* **2019**, *16*, 741-752, doi:10.2174/1567205016666190913155950.
76. Pieber, T.R.; Bode, B.; Mertens, A.; Cho, Y.M.; Christiansen, E.; Hertz, C.L.; Wallenstein, S.O.R.; Buse, J.B.; investigators, P. Efficacy and safety of oral semaglutide with flexible dose adjustment versus sitagliptin in type 2 diabetes (PIONEER 7): a multicentre, open-label, randomised, phase 3a trial. *Lancet Diabetes Endocrinol* **2019**, *7*, 528-539, doi:10.1016/S2213-8587(19)30194-9.
77. Mosenzon, O.; Blicher, T.M.; Rosenlund, S.; Eriksson, J.W.; Heller, S.; Hels, O.H.; Pratley, R.; Sathyapalan, T.; Desouza, C.; Investigators, P. Efficacy and safety of oral semaglutide in patients with type 2 diabetes and moderate renal impairment (PIONEER 5): a placebo-controlled, randomised, phase 3a trial. *Lancet Diabetes Endocrinol* **2019**, *7*, 515-527, doi:10.1016/S2213-8587(19)30192-5.
78. Aroda, V.R.; Rosenstock, J.; Terauchi, Y.; Altuntas, Y.; Lalic, N.M.; Morales Villegas, E.C.; Jeppesen, O.K.; Christiansen, E.; Hertz, C.L.; Haluzik, M.; et al. PIONEER 1: Randomized Clinical Trial of the Efficacy and Safety of Oral Semaglutide Monotherapy in Comparison With Placebo in Patients With Type 2 Diabetes. *Diabetes Care* **2019**, *42*, 1724-1732, doi:10.2337/dc19-0749.
79. Del Prato, S.; Kahn, S.E.; Pavo, I.; Weerakkody, G.J.; Yang, Z.; Doupis, J.; Aizenberg, D.; Wynne, A.G.; Riesmeyer, J.S.; Heine,

R.J.; et al. Tirzepatide versus insulin glargine in type 2 diabetes and increased cardiovascular risk (SURPASS-4): a randomised, open-label, parallel-group, multicentre, phase 3 trial. *Lancet* **2021**, *398*, 1811-1824, doi:10.1016/S0140-6736(21)02188-7.

80. Ludvik, B.; Giorgino, F.; Jodar, E.; Frias, J.P.; Fernandez Lando, L.; Brown, K.; Bray, R.; Rodriguez, A. Once-weekly tirzepatide versus once-daily insulin degludec as add-on to metformin with or without SGLT2 inhibitors in patients with type 2 diabetes (SURPASS-3): a randomised, open-label, parallel-group, phase 3 trial. *Lancet* **2021**, *398*, 583-598, doi:10.1016/S0140-6736(21)01443-4.
81. Kosiborod, M.N.; Esterline, R.; Furtado, R.H.M.; Oscarsson, J.; Gasparyan, S.B.; Koch, G.G.; Martinez, F.; Mukhtar, O.; Verma, S.; Chopra, V.; et al. Dapagliflozin in patients with cardiometabolic risk factors hospitalised with COVID-19 (DARE-19): a randomised, double-blind, placebo-controlled, phase 3 trial. *Lancet Diabetes Endocrinol* **2021**, *9*, 586-594, doi:10.1016/S2213-8587(21)00180-7.
82. Rosenstock, J.; Wysham, C.; Frias, J.P.; Kaneko, S.; Lee, C.J.; Fernandez Lando, L.; Mao, H.; Cui, X.; Karanikas, C.A.; Thieu, V.T. Efficacy and safety of a novel dual GIP and GLP-1 receptor agonist tirzepatide in patients with type 2 diabetes (SURPASS-1): a double-blind, randomised, phase 3 trial. *Lancet* **2021**, *398*, 143-155, doi:10.1016/S0140-6736(21)01324-6.
83. Frias, J.P.; Davies, M.J.; Rosenstock, J.; Perez Manghi, F.C.; Fernandez Lando, L.; Bergman, B.K.; Liu, B.; Cui, X.; Brown, K.; Investigators, S.-. Tirzepatide versus Semaglutide Once Weekly in Patients with Type 2 Diabetes. *N Engl J Med* **2021**, *385*, 503-515, doi:10.1056/NEJMoa2107519.
84. Rubino, D.; Abrahamsson, N.; Davies, M.; Hesse, D.; Greenway, F.L.; Jensen, C.; Lingvay, I.; Mosenzon, O.; Rosenstock, J.; Rubio, M.A.; et al. Effect of Continued Weekly Subcutaneous Semaglutide vs Placebo on Weight Loss Maintenance in Adults With Overweight or Obesity: The STEP 4 Randomized Clinical Trial. *Jama* **2021**, *325*, 1414-1425, doi:10.1001/jama.2021.3224.
85. Davies, M.; Faerch, L.; Jeppesen, O.K.; Pakseresht, A.; Pedersen, S.D.; Perreault, L.; Rosenstock, J.; Shimomura, I.; Viljoen, A.; Wadden, T.A.; et al. Semaglutide 2.4 mg once a week in adults with overweight or obesity, and type 2 diabetes (STEP 2): a randomised, double-blind, double-dummy, placebo-controlled, phase 3 trial. *Lancet* **2021**, *397*, 971-984, doi:10.1016/S0140-

6736(21)00213-0.
86. Wadden, T.A.; Bailey, T.S.; Billings, L.K.; Davies, M.; Frias, J.P.; Koroleva, A.; Lingvay, I.; O'Neil, P.M.; Rubino, D.M.; Skovgaard, D.; et al. Effect of Subcutaneous Semaglutide vs Placebo as an Adjunct to Intensive Behavioral Therapy on Body Weight in Adults With Overweight or Obesity: The STEP 3 Randomized Clinical Trial. *Jama* **2021**, *325*, 1403-1413, doi:10.1001/jama.2021.1831.
87. Wilding, J.P.H.; Batterham, R.L.; Calanna, S.; Davies, M.; Van Gaal, L.F.; Lingvay, I.; McGowan, B.M.; Rosenstock, J.; Tran, M.T.D.; Wadden, T.A.; et al. Once-Weekly Semaglutide in Adults with Overweight or Obesity. *N Engl J Med* **2021**, *384*, 989-1002, doi:10.1056/NEJMoa2032183.
88. Bhatt, D.L.; Szarek, M.; Steg, P.G.; Cannon, C.P.; Leiter, L.A.; McGuire, D.K.; Lewis, J.B.; Riddle, M.C.; Voors, A.A.; Metra, M.; et al. Sotagliflozin in Patients with Diabetes and Recent Worsening Heart Failure. *N Engl J Med* **2021**, *384*, 117-128, doi:10.1056/NEJMoa2030183.
89. Frias, J.P.; Choi, J.; Rosenstock, J.; Popescu, L.; Niemoeller, E.; Muehlen-Bartmer, I.; Baek, S. Efficacy and Safety of Once-Weekly Efpeglenatide Monotherapy Versus Placebo in Type 2 Diabetes: The AMPLITUDE-M Randomized Controlled Trial. *Diabetes Care* **2022**, *45*, 1592-1600, doi:10.2337/dc21-2656.
90. Jastreboff, A.M.; Aronne, L.J.; Ahmad, N.N.; Wharton, S.; Connery, L.; Alves, B.; Kiyosue, A.; Zhang, S.; Liu, B.; Bunck, M.C.; et al. Tirzepatide Once Weekly for the Treatment of Obesity. *N Engl J Med* **2022**, *387*, 205-216, doi:10.1056/NEJMoa2206038.
91. Kellerer, M.; Kaltoft, M.S.; Lawson, J.; Nielsen, L.L.; Strojek, K.; Tabak, O.; Jacob, S. Effect of once-weekly semaglutide versus thrice-daily insulin aspart, both as add-on to metformin and optimized insulin glargine treatment in participants with type 2 diabetes (SUSTAIN 11): A randomized, open-label, multinational, phase 3b trial. *Diabetes Obes Metab* **2022**, *24*, 1788-1799, doi:10.1111/dom.14765.
92. Tuttle, K.R.; Levin, A.; Nangaku, M.; Kadowaki, T.; Agarwal, R.; Hauske, S.J.; Elsasser, A.; Ritter, I.; Steubl, D.; Wanner, C.; et al. Safety of Empagliflozin in Patients With Type 2 Diabetes and Chronic Kidney Disease: Pooled Analysis of Placebo-Controlled Clinical Trials. *Diabetes Care* **2022**, *45*, 1445-1452, doi:10.2337/dc21-2034.

93. Heise, T.; Mari, A.; DeVries, J.H.; Urva, S.; Li, J.; Pratt, E.J.; Coskun, T.; Thomas, M.K.; Mather, K.J.; Haupt, A.; et al. Effects of subcutaneous tirzepatide versus placebo or semaglutide on pancreatic islet function and insulin sensitivity in adults with type 2 diabetes: a multicentre, randomised, double-blind, parallel-arm, phase 1 clinical trial. *Lancet Diabetes Endocrinol* **2022**, *10*, 418-429, doi:10.1016/S2213-8587(22)00085-7.
94. Fox, C.K.; Clark, J.M.; Rudser, K.D.; Ryder, J.R.; Gross, A.C.; Nathan, B.M.; Sunni, M.; Dengel, D.R.; Billington, C.J.; Bensignor, M.O.; et al. Exenatide for weight-loss maintenance in adolescents with severe obesity: A randomized, placebo-controlled trial. *Obesity (Silver Spring)* **2022**, *30*, 1105-1115, doi:10.1002/oby.23395.
95. Spertus, J.A.; Birmingham, M.C.; Nassif, M.; Damaraju, C.V.; Abbate, A.; Butler, J.; Lanfear, D.E.; Lingvay, I.; Kosiborod, M.N.; Januzzi, J.L. The SGLT2 inhibitor canagliflozin in heart failure: the CHIEF-HF remote, patient-centered randomized trial. *Nat Med* **2022**, *28*, 809-813, doi:10.1038/s41591-022-01703-8.
96. Kadowaki, T.; Isendahl, J.; Khalid, U.; Lee, S.Y.; Nishida, T.; Ogawa, W.; Tobe, K.; Yamauchi, T.; Lim, S.; investigators, S. Semaglutide once a week in adults with overweight or obesity, with or without type 2 diabetes in an east Asian population (STEP 6): a randomised, double-blind, double-dummy, placebo-controlled, phase 3a trial. *Lancet Diabetes Endocrinol* **2022**, *10*, 193-206, doi:10.1016/S2213-8587(22)00008-0.
97. Rubino, D.M.; Greenway, F.L.; Khalid, U.; O'Neil, P.M.; Rosenstock, J.; Sorrig, R.; Wadden, T.A.; Wizert, A.; Garvey, W.T.; Investigators, S. Effect of Weekly Subcutaneous Semaglutide vs Daily Liraglutide on Body Weight in Adults With Overweight or Obesity Without Diabetes: The STEP 8 Randomized Clinical Trial. *Jama* **2022**, *327*, 138-150, doi:10.1001/jama.2021.23619.
98. Rodgers, M.; Migdal, A.L.; Rodriguez, T.G.; Chen, Z.Z.; Nath, A.K.; Gerszten, R.E.; Kasid, N.; Toschi, E.; Tripaldi, J.; Heineman, B.; et al. Weight Loss Outcomes Among Early High Responders to Exenatide Treatment: A Randomized, Placebo Controlled Study in Overweight and Obese Women. *Front Endocrinol (Lausanne)* **2021**, *12*, 742873, doi:10.3389/fendo.2021.742873.
99. Rosenstock, J.; Frias, J.P.; Rodbard, H.W.; Tofe, S.; Sears, E.; Huh, R.; Fernandez Lando, L.; Patel, H. Tirzepatide vs Insulin

Lispro Added to Basal Insulin in Type 2 Diabetes: The SURPASS-6 Randomized Clinical Trial. *Jama* **2023**, *330*, 1631-1640, doi:10.1001/jama.2023.20294.
100. Garvey, W.T.; Frias, J.P.; Jastreboff, A.M.; le Roux, C.W.; Sattar, N.; Aizenberg, D.; Mao, H.; Zhang, S.; Ahmad, N.N.; Bunck, M.C.; et al. Tirzepatide once weekly for the treatment of obesity in people with type 2 diabetes (SURMOUNT-2): a double-blind, randomised, multicentre, placebo-controlled, phase 3 trial. *Lancet* **2023**, *402*, 613-626, doi:10.1016/S0140-6736(23)01200-X.
101. Frias, J.P.; Hsia, S.; Eyde, S.; Liu, R.; Ma, X.; Konig, M.; Kazda, C.; Mather, K.J.; Haupt, A.; Pratt, E.; et al. Efficacy and safety of oral orforglipron in patients with type 2 diabetes: a multicentre, randomised, dose-response, phase 2 study. *Lancet* **2023**, *402*, 472-483, doi:10.1016/S0140-6736(23)01302-8.
102. Feng, P.; Sheng, X.; Ji, Y.; Urva, S.; Wang, F.; Miller, S.; Qian, C.; An, Z.; Cui, Y. A Phase 1 Multiple Dose Study of Tirzepatide in Chinese Patients with Type 2 Diabetes. *Adv Ther* **2023**, *40*, 3434-3445, doi:10.1007/s12325-023-02536-8.
103. Gao, L.; Lee, B.W.; Chawla, M.; Kim, J.; Huo, L.; Du, L.; Huang, Y.; Ji, L. Tirzepatide versus insulin glargine as second-line or third-line therapy in type 2 diabetes in the Asia-Pacific region: the SURPASS-AP-Combo trial. *Nat Med* **2023**, *29*, 1500-1510, doi:10.1038/s41591-023-02344-1.
104. Buse, J.B.; Nordahl Christensen, H.; Harty, B.J.; Mitchell, J.; Soule, B.P.; Zacherle, E.; Cziraky, M.; Willey, V.J. Study design and baseline profile for adults with type 2 diabetes in the once-weekly subcutaneous SEmaglutide randomized PRAgmatic (SEPRA) trial. *BMJ Open Diabetes Res Care* **2023**, *11*, doi:10.1136/bmjdrc-2022-003206.
105. Aroda, V.R.; Frias, J.P.; Ji, L.; Niemoeller, E.; Nguyen-Pascal, M.L.; Denkel, K.; Espinasse, M.; Guo, H.; Baek, S.; Choi, J.; et al. Efficacy and safety of once-weekly efpeglenatide in people with suboptimally controlled type 2 diabetes: The AMPLITUDE-D, AMPLITUDE-L and AMPLITUDE-S randomized controlled trials. *Diabetes Obes Metab* **2023**, *25*, 2084-2095, doi:10.1111/dom.15079.
106. Cherney, D.Z.I.; Ferrannini, E.; Umpierrez, G.E.; Peters, A.L.; Rosenstock, J.; Powell, D.R.; Davies, M.J.; Banks, P.; Agarwal, R. Efficacy and safety of sotagliflozin in patients with type 2 diabetes and stage 3 chronic kidney disease. *Diabetes Obes Metab*

2023, *25*, 1646-1657, doi:10.1111/dom.15019.

107. Garvey, W.T.; Batterham, R.L.; Bhatta, M.; Buscemi, S.; Christensen, L.N.; Frias, J.P.; Jodar, E.; Kandler, K.; Rigas, G.; Wadden, T.A.; et al. Two-year effects of semaglutide in adults with overweight or obesity: the STEP 5 trial. *Nat Med* **2022**, *28*, 2083-2091, doi:10.1038/s41591-022-02026-4.
108. Inagaki, N.; Takeuchi, M.; Oura, T.; Imaoka, T.; Seino, Y. Efficacy and safety of tirzepatide monotherapy compared with dulaglutide in Japanese patients with type 2 diabetes (SURPASS J-mono): a double-blind, multicentre, randomised, phase 3 trial. *Lancet Diabetes Endocrinol* **2022**, *10*, 623-633, doi:10.1016/S2213-8587(22)00188-7.
109. Bliddal, H.; Bays, H.; Czernichow, S.; Udden Hemmingsson, J.; Hjelmesaeth, J.; Hoffmann Morville, T.; Koroleva, A.; Skov Neergaard, J.; Velez Sanchez, P.; Wharton, S.; et al. Once-Weekly Semaglutide in Persons with Obesity and Knee Osteoarthritis. *N Engl J Med* **2024**, *391*, 1573-1583, doi:10.1056/NEJMoa2403664.
110. Zhao, L.; Cheng, Z.; Lu, Y.; Liu, M.; Chen, H.; Zhang, M.; Wang, R.; Yuan, Y.; Li, X. Tirzepatide for Weight Reduction in Chinese Adults With Obesity: The SURMOUNT-CN Randomized Clinical Trial. *Jama* **2024**, *332*, 551-560, doi:10.1001/jama.2024.9217.
111. Dei Cas, A.; Micheli, M.M.; Aldigeri, R.; Gardini, S.; Ferrari-Pellegrini, F.; Perini, M.; Messa, G.; Antonini, M.; Spigoni, V.; Cinquegrani, G.; et al. Long-acting exenatide does not prevent cognitive decline in mild cognitive impairment: a proof-of-concept clinical trial. *Journal of endocrinological investigation* **2024**, *47*, 2339-2349, doi:10.1007/s40618-024-02320-7.
112. Mu, Y.; Bao, X.; Eliaschewitz, F.G.; Hansen, M.R.; Kim, B.T.; Koroleva, A.; Ma, R.C.W.; Yang, T.; Zu, N.; Liu, M.; et al. Efficacy and safety of once weekly semaglutide 2.4 mg for weight management in a predominantly east Asian population with overweight or obesity (STEP 7): a double-blind, multicentre, randomised controlled trial. *Lancet Diabetes Endocrinol* **2024**, *12*, 184-195, doi:10.1016/S2213-8587(23)00388-1.
113. Tuttle, K.R.; Hauske, S.J.; Canziani, M.E.; Caramori, M.L.; Cherney, D.; Cronin, L.; Heerspink, H.J.L.; Hugo, C.; Nangaku, M.; Rotter, R.C.; et al. Efficacy and safety of aldosterone synthase inhibition with and without empagliflozin for chronic kidney disease: a randomised, controlled, phase 2 trial. *Lancet* **2024**, *403*, 379-390, doi:10.1016/S0140-6736(23)02408-X.

114. Aronne, L.J.; Sattar, N.; Horn, D.B.; Bays, H.E.; Wharton, S.; Lin, W.Y.; Ahmad, N.N.; Zhang, S.; Liao, R.; Bunck, M.C.; et al. Continued Treatment With Tirzepatide for Maintenance of Weight Reduction in Adults With Obesity: The SURMOUNT-4 Randomized Clinical Trial. *Jama* **2024**, *331*, 38-48, doi:10.1001/jama.2023.24945.
115. Wadden, T.A.; Chao, A.M.; Machineni, S.; Kushner, R.; Ard, J.; Srivastava, G.; Halpern, B.; Zhang, S.; Chen, J.; Bunck, M.C.; et al. Tirzepatide after intensive lifestyle intervention in adults with overweight or obesity: the SURMOUNT-3 phase 3 trial. *Nat Med* **2023**, *29*, 2909-2918, doi:10.1038/s41591-023-02597-w.
116. Wason, S. Efficacy and Safety of Sotagliflozin Versus Placebo in Participants With Type 2 Diabetes Mellitus Who Have Inadequate Glycemic Control While Taking Insulin Alone or With Other Oral Antidiabetic Agents (SOTA-INS). Available online: https://clinicaltrials.gov/study/NCT03285594?cond=NCT03285594&rank=1 (accessed on 2024/10/28).
117. Lock, J.P. Bexagliflozin Efficacy and Safety Trial (BEST). Available online: https://clinicaltrials.gov/study/NCT02558296?cond=NCT02558296&rank=1 (accessed on 2024/10/28).
118. Panfili, E.; Frontino, G.; Pallotta, M.T. GLP-1 receptor agonists as promising disease-modifying agents in WFS1 spectrum disorder. *Front Clin Diabetes Healthc* **2023**, *4*, 1171091, doi:10.3389/fcdhc.2023.1171091.
119. Samocha-Bonet, D.; Wu, B.; Ryugo, D.K. Diabetes mellitus and hearing loss: A review. *Ageing Res Rev* **2021**, *71*, 101423, doi:10.1016/j.arr.2021.101423.
120. Lee, B.W.; Cho, Y.M.; Kim, S.G.; Ko, S.H.; Lim, S.; Dahaoui, A.; Jeong, J.S.; Lim, H.J.; Yu, J.M. Efficacy and Safety of Once-Weekly Semaglutide Versus Once-Daily Sitagliptin as Metformin Add-on in a Korean Population with Type 2 Diabetes. *Diabetes Ther* **2024**, *15*, 547-563, doi:10.1007/s13300-023-01515-0.
121. Natale, P.; Tunnicliffe, D.J.; Toyama, T.; Palmer, S.C.; Saglimbene, V.M.; Ruospo, M.; Gargano, L.; Stallone, G.; Gesualdo, L.; Strippoli, G.F. Sodium-glucose co-transporter protein 2 (SGLT2) inhibitors for people with chronic kidney disease and diabetes. *The Cochrane database of systematic reviews* **2024**, *5*, CD015588, doi:10.1002/14651858.CD015588.pub2.
122. SURMOUNT-J. A Study of Tirzepatide (LY3298176) in Participants With Obesity Disease (SURMOUNT-J). Available online: https://clinicaltrials.gov/study/NCT04844918?cond=NCT04844918&rank=1 (accessed on 2024/10/28).

123. The, E.-K.C.G.; Herrington, W.G.; Staplin, N.; Wanner, C.; Green, J.B.; Hauske, S.J.; Emberson, J.R.; Preiss, D.; Judge, P.; Mayne, K.J.; et al. Empagliflozin in Patients with Chronic Kidney Disease. *N Engl J Med* **2023**, *388*, 117-127, doi:10.1056/NEJMoa2204233.
124. Ji, L.; Lu, Y.; Li, Q.; Fu, L.; Luo, Y.; Lei, T.; Li, L.; Ye, S.; Shi, B.; Li, X.; et al. Efficacy and safety of empagliflozin in combination with insulin in Chinese patients with type 2 diabetes and insufficient glycaemic control: A phase III, randomized, double-blind, placebo-controlled, parallel study. *Diabetes Obes Metab* **2023**, *25*, 1839-1848, doi:10.1111/dom.15041.
125. Lincoff, A.M.; Brown-Frandsen, K.; Colhoun, H.M.; Deanfield, J.; Emerson, S.S.; Esbjerg, S.; Hardt-Lindberg, S.; Hovingh, G.K.; Kahn, S.E.; Kushner, R.F.; et al. Semaglutide and Cardiovascular Outcomes in Obesity without Diabetes. *N Engl J Med* **2023**, *389*, 2221-2232, doi:10.1056/NEJMoa2307563.
126. Dahl, D.; Onishi, Y.; Norwood, P.; Huh, R.; Bray, R.; Patel, H.; Rodriguez, A. Effect of Subcutaneous Tirzepatide vs Placebo Added to Titrated Insulin Glargine on Glycemic Control in Patients With Type 2 Diabetes: The SURPASS-5 Randomized Clinical Trial. *Jama* **2022**, *327*, 534-545, doi:10.1001/jama.2022.0078.
127. Kadowaki, T.; Chin, R.; Ozeki, A.; Imaoka, T.; Ogawa, Y. Safety and efficacy of tirzepatide as an add-on to single oral antihyperglycaemic medication in patients with type 2 diabetes in Japan (SURPASS J-combo): a multicentre, randomised, open-label, parallel-group, phase 3 trial. *Lancet Diabetes Endocrinol* **2022**, *10*, 634-644, doi:10.1016/S2213-8587(22)00187-5.
128. Solomon, S.D.; McMurray, J.J.V.; Claggett, B.; de Boer, R.A.; DeMets, D.; Hernandez, A.F.; Inzucchi, S.E.; Kosiborod, M.N.; Lam, C.S.P.; Martinez, F.; et al. Dapagliflozin in Heart Failure with Mildly Reduced or Preserved Ejection Fraction. *N Engl J Med* **2022**, *387*, 1089-1098, doi:10.1056/NEJMoa2206286.
129. Voors, A.A.; Angermann, C.E.; Teerlink, J.R.; Collins, S.P.; Kosiborod, M.; Biegus, J.; Ferreira, J.P.; Nassif, M.E.; Psotka, M.A.; Tromp, J.; et al. The SGLT2 inhibitor empagliflozin in patients hospitalized for acute heart failure: a multinational randomized trial. *Nat Med* **2022**, *28*, 568-574, doi:10.1038/s41591-021-01659-1.
130. Wada, T.; Mori-Anai, K.; Takahashi, A.; Matsui, T.; Inagaki, M.; Iida, M.; Maruyama, K.; Tsuda, H. Effect of canagliflozin on the decline of estimated glomerular filtration rate in chronic kidney disease patients with type 2 diabetes mellitus: A

multicenter, randomized, double-blind, placebo-controlled, parallel-group, phase III study in Japan. *J Diabetes Investig* **2022**, *13*, 1981-1989, doi:10.1111/jdi.13888.
131. Anker, S.D.; Butler, J.; Filippatos, G.; Ferreira, J.P.; Bocchi, E.; Bohm, M.; Brunner-La Rocca, H.P.; Choi, D.J.; Chopra, V.; Chuquiure-Valenzuela, E.; et al. Empagliflozin in Heart Failure with a Preserved Ejection Fraction. *N Engl J Med* **2021**, *385*, 1451-1461, doi:10.1056/NEJMoa2107038.
132. Gerstein, H.C.; Sattar, N.; Rosenstock, J.; Ramasundarahettige, C.; Pratley, R.; Lopes, R.D.; Lam, C.S.P.; Khurmi, N.S.; Heenan, L.; Del Prato, S.; et al. Cardiovascular and Renal Outcomes with Efpeglenatide in Type 2 Diabetes. *N Engl J Med* **2021**, *385*, 896-907, doi:10.1056/NEJMoa2108269.
133. Wason, S. Efficacy and Bone Safety of Sotagliflozin 400 and 200 mg Versus Placebo in Participants With Type 2 Diabetes Mellitus Who Have Inadequate Glycemic Control (SOTA-BONE). Available online: https://clinicaltrials.gov/study/NCT03386344?cond=NCT03386344&rank=1 (accessed on 2024/10/28).
134. Cannon, C.P.; Pratley, R.; Dagogo-Jack, S.; Mancuso, J.; Huyck, S.; Masiukiewicz, U.; Charbonnel, B.; Frederich, R.; Gallo, S.; Cosentino, F.; et al. Cardiovascular Outcomes with Ertugliflozin in Type 2 Diabetes. *N Engl J Med* **2020**, *383*, 1425-1435, doi:10.1056/NEJMoa2004967.
135. Packer, M.; Anker, S.D.; Butler, J.; Filippatos, G.; Pocock, S.J.; Carson, P.; Januzzi, J.; Verma, S.; Tsutsui, H.; Brueckmann, M.; et al. Cardiovascular and Renal Outcomes with Empagliflozin in Heart Failure. *N Engl J Med* **2020**, *383*, 1413-1424, doi:10.1056/NEJMoa2022190.
136. Gallo, S.; Charbonnel, B.; Goldman, A.; Shi, H.; Huyck, S.; Darekar, A.; Lauring, B.; Terra, S.G. Long-term efficacy and safety of ertugliflozin in patients with type 2 diabetes mellitus inadequately controlled with metformin monotherapy: 104-week VERTIS MET trial. *Diabetes Obes Metab* **2019**, *21*, 1027-1036, doi:10.1111/dom.13631.
137. Gerstein, H.C.; Colhoun, H.M.; Dagenais, G.R.; Diaz, R.; Lakshmanan, M.; Pais, P.; Probstfield, J.; Riesmeyer, J.S.; Riddle, M.C.; Ryden, L.; et al. Dulaglutide and cardiovascular outcomes in type 2 diabetes (REWIND): a double-blind, randomised placebo-controlled trial. *Lancet* **2019**, *394*, 121-130, doi:10.1016/S0140-6736(19)31149-3.

138. Rosenstock, J.; Allison, D.; Birkenfeld, A.L.; Blicher, T.M.; Deenadayalan, S.; Jacobsen, J.B.; Serusclat, P.; Violante, R.; Watada, H.; Davies, M.; et al. Effect of Additional Oral Semaglutide vs Sitagliptin on Glycated Hemoglobin in Adults With Type 2 Diabetes Uncontrolled With Metformin Alone or With Sulfonylurea: The PIONEER 3 Randomized Clinical Trial. *Jama* **2019**, *321*, 1466-1480, doi:10.1001/jama.2019.2942.
139. Wang, J.; Li, H.Q.; Xu, X.H.; Kong, X.C.; Sun, R.; Jing, T.; Ye, L.; Su, X.F.; Ma, J.H. The Effects of Once-Weekly Dulaglutide and Insulin Glargine on Glucose Fluctuation in Poorly Oral-Antidiabetic Controlled Patients with Type 2 Diabetes Mellitus. *Biomed Res Int* **2019**, *2019*, 2682657, doi:10.1155/2019/2682657.
140. Wiviott, S.D.; Raz, I.; Bonaca, M.P.; Mosenzon, O.; Kato, E.T.; Cahn, A.; Silverman, M.G.; Zelniker, T.A.; Kuder, J.F.; Murphy, S.A.; et al. Dapagliflozin and Cardiovascular Outcomes in Type 2 Diabetes. *N Engl J Med* **2019**, *380*, 347-357, doi:10.1056/NEJMoa1812389.
141. Hernandez, A.F.; Green, J.B.; Janmohamed, S.; D'Agostino, R.B., Sr.; Granger, C.B.; Jones, N.P.; Leiter, L.A.; Rosenberg, A.E.; Sigmon, K.N.; Somerville, M.C.; et al. Albiglutide and cardiovascular outcomes in patients with type 2 diabetes and cardiovascular disease (Harmony Outcomes): a double-blind, randomised placebo-controlled trial. *Lancet* **2018**, *392*, 1519-1529, doi:10.1016/S0140-6736(18)32261-X.
142. Holman, R.R.; Bethel, M.A.; Mentz, R.J.; Thompson, V.P.; Lokhnygina, Y.; Buse, J.B.; Chan, J.C.; Choi, J.; Gustavson, S.M.; Iqbal, N.; et al. Effects of Once-Weekly Exenatide on Cardiovascular Outcomes in Type 2 Diabetes. *N Engl J Med* **2017**, *377*, 1228-1239, doi:10.1056/NEJMoa1612917.
143. Neal, B.; Perkovic, V.; Mahaffey, K.W.; de Zeeuw, D.; Fulcher, G.; Erondu, N.; Shaw, W.; Law, G.; Desai, M.; Matthews, D.R.; et al. Canagliflozin and Cardiovascular and Renal Events in Type 2 Diabetes. *N Engl J Med* **2017**, *377*, 644-657, doi:10.1056/NEJMoa1611925.
144. Marso, S.P.; Daniels, G.H.; Brown-Frandsen, K.; Kristensen, P.; Mann, J.F.; Nauck, M.A.; Nissen, S.E.; Pocock, S.; Poulter, N.R.; Ravn, L.S.; et al. Liraglutide and Cardiovascular Outcomes in Type 2 Diabetes. *N Engl J Med* **2016**, *375*, 311-322, doi:10.1056/NEJMoa1603827.

145. Marso, S.P.; Bain, S.C.; Consoli, A.; Eliaschewitz, F.G.; Jodar, E.; Leiter, L.A.; Lingvay, I.; Rosenstock, J.; Seufert, J.; Warren, M.L.; et al. Semaglutide and Cardiovascular Outcomes in Patients with Type 2 Diabetes. *N Engl J Med* **2016**, *375*, 1834-1844, doi:10.1056/NEJMoa1607141.
146. Pfeffer, M.A.; Claggett, B.; Diaz, R.; Dickstein, K.; Gerstein, H.C.; Kober, L.V.; Lawson, F.C.; Ping, L.; Wei, X.; Lewis, E.F.; et al. Lixisenatide in Patients with Type 2 Diabetes and Acute Coronary Syndrome. *N Engl J Med* **2015**, *373*, 2247-2257, doi:10.1056/NEJMoa1509225.
147. Zinman, B.; Wanner, C.; Lachin, J.M.; Fitchett, D.; Bluhmki, E.; Hantel, S.; Mattheus, M.; Devins, T.; Johansen, O.E.; Woerle, H.J.; et al. Empagliflozin, Cardiovascular Outcomes, and Mortality in Type 2 Diabetes. *N Engl J Med* **2015**, *373*, 2117-2128, doi:10.1056/NEJMoa1504720.
